# Regioselective Synthesis of 5-Substituted 3-(β-d-Glycopyranosyl)isoxazoles and -isoxazolines by 1,3-Dipolar Cycloaddition as Potential Anticancer Agents and Glycogen Phosphorylase Inhibitors

**DOI:** 10.3390/ijms26178167

**Published:** 2025-08-22

**Authors:** Tímea Kaszás, Bence Szakács, Márta Bertalan, Tekla Blága, Faria Hameed, Ákos Lengyel, Samreen Saifi, Éva Juhász-Tóth, Luca A. Varga, Tibor Docsa, Adrienn Sipos, Péter Bai, Anita Ábrahám, Attila Kiss-Szikszai, Sándor Kun, György Attila Kiss, János József, László Juhász, Marietta Tóth

**Affiliations:** 1 Department of Organic Chemistry, University of Debrecen, P.O. Box 400, H-4002 Debrecen, Hungary; kaszas.timea@science.unideb.hu (T.K.); szakacs.bence@science.unideb.hu (B.S.); bertalanmarta02@gmail.com (M.B.); tekla.tblaga@gmail.com (T.B.); fariahameed555@gmail.com (F.H.); polszka76@mailbox.unideb.hu (Á.L.); saifi.samreen@science.unideb.hu (S.S.); toth.eva@science.unideb.hu (É.J.-T.); dulryc@unideb.hu (A.Á.); kiss.attila@science.unideb.hu (A.K.-S.); kun.sandor@science.unideb.hu (S.K.); kiss.gyorgy@science.unideb.hu (G.A.K.); jozsef.janos@science.unideb.hu (J.J.); juhasz.laszlo@science.unideb.hu (L.J.); 2 Doctoral School of Chemistry, University of Debrecen, P.O. Box 400, H-4002 Debrecen, Hungary; 3 Department of Medical Chemistry, Faculty of Medicine, University of Debrecen, P.O. Box 400, H-4002 Debrecen, Hungary; varga.luca@med.unideb.hu (L.A.V.); tdocsa@med.unideb.hu (T.D.); siposadri@med.unideb.hu (A.S.); baip@med.unideb.hu (P.B.); 4 HUN-REN Cell Biology and Signaling Research Group, H-4032 Debrecen, Hungary; 5 MTA-DE Lendület Laboratory of Cellular Metabolism, H-4032 Debrecen, Hungary; 6 Research Center for Molecular Medicine, Faculty of Medicine, University of Debrecen, H-4032 Debrecen, Hungary

**Keywords:** 1,3-dipolar cycloaddition, nitrile oxides, anhydro-aldose oxime, 3-(β-d-glycopyranosyl)isoxazole, 3-(β-d-glycopyranosyl)isoxazoline, anticancer activity, glycogen phosphorylase inhibition

## Abstract

Anhydro-aldose oximes were employed to generate in situ nitrile oxides via a halogenation/base-induced elimination sequence in the presence of NCS and Et_3_N, which were then used in 1,3-dipolar cycloadditions with alkenes and alkynes to afford 5-substituted 3-(β-d-glycopyranosyl)isoxazole and -isoxazoline derivatives exclusively. These newly synthesized glycomimetics were evaluated for their potential to act as antagonists of A2780 ovarian cancer cells and as inhibitors of glycogen phosphorylase; however, they exhibited no significant activity.

## 1. Introduction

Carbohydrates have been shown to play pivotal roles in many biological processes, including bacterial and viral infections, cell adhesion, contact inhibition of cell division, immune responses, inflammation, and metastasis.

Therefore, the pharmaceutical potential of carbohydrates is considerable [1,2,3]. Unfortunately, the low hydrolytic stability of natural *O*-glycosidic bonds limits their use as drug candidates [4]. However, more hydrolytically stable moieties can be synthesized by replacing the glycosidic oxygen with different atoms—most frequently S, N, and C—providing a variety of methods to produce glycomimetic compounds [5]. Multi-atomic replacement of the glycosidic oxygen is also well-known (e.g., S-S, S-Se, SO_2_-N, N-CO-N) [6]. These compounds offer several advantages, including simpler synthesis, resistance to hydrolysis and metabolic processes, a wide range of derivatization possibilities, and potential utility as glycobiological tools and leads in drug design [7].

**Scheme 1:**
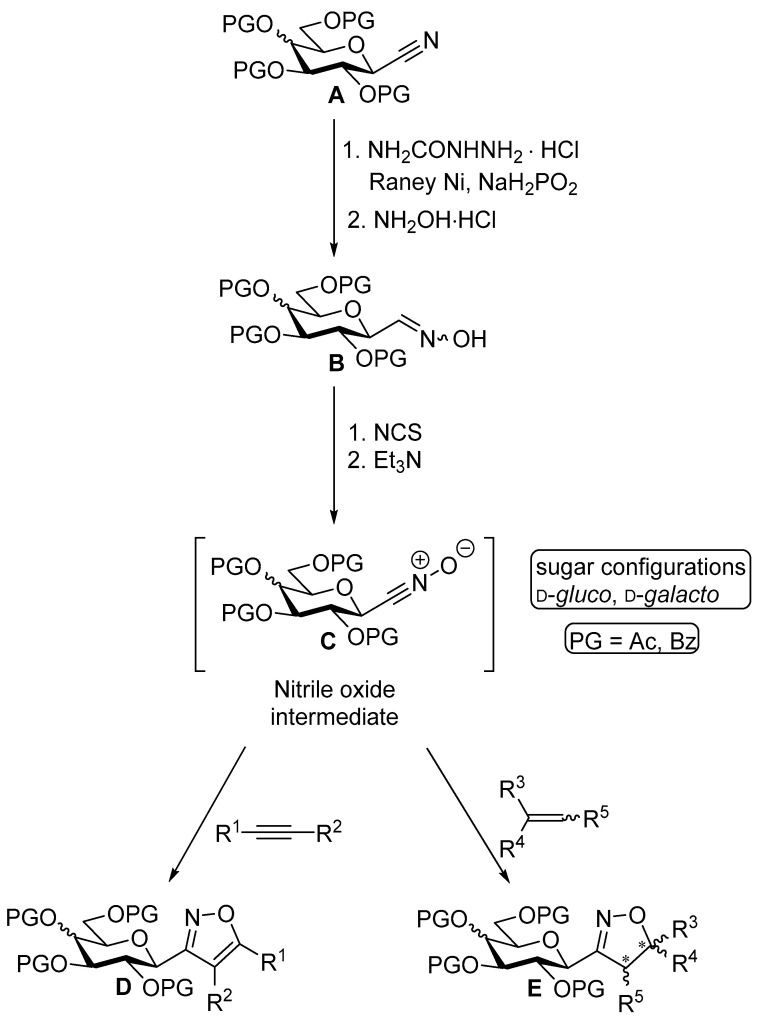
Present study to synthesize isoxazoles **D** and isoxazolines **E**.

Only a few examples of the synthesis of substituted 3-(*C*-glycopyranosyl)isoxazoles and -isoxazolines have been reported in the literature. In these papers, glycopyranosyl nitrile oxides were reacted with alkynes (dimethyl acetylenedicarboxylate [8,9], ethyl propiolate [8], propargyl glycosides [10,11], 6-*O*-propargyl cyclomaltoheptaose derivative [10], ethynyl α-amino-acid derivatives [12], tri- and tetrapropargylated compounds [10,11]) and alkenes (styrene, allyl alcohol, 4-*C*-vinyl furanose derivative, methylenecyclohexene [8], norbornene [8,13], norbornadiene [13]) in 1,3-dipolar cycloaddition reactions. The glycopyranosyl nitrile oxides were generated in situ from the corresponding oximes either by base-induced dehydrohalogenation of the derived hydroximoyl chloride (Cl_2_, Et_3_N) [8,9,12,14] or bromide (NBS, Et_3_N) [8,9,12,14] or by oxidation with aq. hypochlorite (NaOCl, Et_3_N) [8,14]. Direct dehydration of nitromethanes to nitrile oxides by phenyl isocyanate or TDI (toluene-2,4-diisocyanate) in the presence of a catalytic amount of trimethylamine was also reported [10,11,13,14]. In the absence of dipolarophiles, dimerization of carbohydrate nitrile oxides leads to the formation of 3,4-bis-glycopyranosyl-1,2,5-oxadiazole *N*-oxides (bis-glycopyranosylfuroxans) in high yields [8,9,14,15]. These compounds may also appear as by-products in the above 1,3-dipolar cycloadditions. Another possibility to obtain 3-(2′,3′,4′,6′-tetra-*O*-benzoyl-β-d-glucopyranosyl)-5-phenylisoxazole involved the cyclization of the corresponding *C*-glucopyranosyl phenylethynyl ketone with hydroxylamine [15,16]. Anhydro-aldose oximes (*C*-glycosyl formaldoximes) can easily be prepared either from anhydro-aldoses (*C*-glycosyl formaldehydes) in a condensation reaction with hydroxylamine [9] or from *C*-glycosyl nitromethanes by reduction with the complex [Et_3_NH][(PhS)_3_Sn] generated from SnCl_2_/PhSH/Et_3_N [8,14]. Our group has developed a simple synthetic method for the preparation of anhydro-aldoximes **B** by the reduction in the readily accessible *C*-glycosyl cyanides **A** and the in situ trapping of the imine intermediate by semicarbazide, followed by a transimination reaction in the presence of hydroxylamine hydrochloride (Scheme 1) [15,17].

Herein, we report our findings on the synthesis of novel (3-*C*-glycosyl)isoxazoles **D** and -isoxazolines **E** via 1,3-dipolar cycloaddition reactions of nitrile oxides **C**. We also describe the biological activity of galactose-derived products as antagonists of the A2780 ovarian cancer cell line and of glucose analogues as glycogen phosphorylase inhibitors.

## 2. Results and Discussion

### 2.1. Syntheses

We started our investigations with the 1,3-dipolar cycloaddition reactions of *C*-(2,3,4,6-tetra-*O*-benzoyl-β-d-glucopyranosyl)formaldehyde oxime (**1a**) and styrene (**2a**) (Table 1). First, the literature conditions [17] were applied using aqueous sodium hypochlorite in THF [18] at room temperature under inert (N_2_) atmosphere to give 3-(2′,3′,4′,6′-tetra-*O*-benzoyl-β-d-glucopyranosyl)-5-phenylisoxazoline (**3a**) in 60% yield as inseparable mixtures of two diastereoisomers with a 1.1:1 diastereomeric ratio (entry 1). Using KI/oxone (2 KHSO_5_•KHSO_4_•K_2_SO_4_) as the oxidizing agent in a mixture of methanol and water [19] resulted in a complex reaction mixture (entry 2). Next, base-induced dehydrohalogenation of the derived hydroximoyl bromide [9] was used. In this reaction NBS (1.1 equiv.) was added to a stirred solution of oxime **1a** and 5-fold excess of styrene (**2a**) in DMF followed by dropwise administration of a solution of Et_3_N in DMF to get **3a** in 58% yield (entry 3).

Replacing DMF with CH_2_Cl_2_ (entry 4) and/or using NCS instead of NBS slightly increased the yield of **3a** to 64% and 65%, respectively.

The dimerization of *C*-(2,3,4,6-tetra-*O*-benzoyl-β-d-glucopyranosyl)formaldehyde nitrile oxides resulted in diglucopyranosylfuroxan **4a**, which was detected after the work-up in the ^1^H NMR spectra (entries 1, 3–5) (see Supplementary Materials). In these cycloadditions, two regioisomers, 5- and 4-substituted isoxazolines, are normally formed, but in our case, only 3-(*C*-glucosyl)-5-phenylisoxazoline **3a** was formed.

Under optimized reaction conditions, digalactopyranosyl furoxan (**4b**) [14] was obtained as the main product in 61% yield from the reaction of phenylacetylene (**5a**) with *C*-(2,3,4,6-tetra-*O*-acetyl-β-d-galactopyranosyl)formaldehyde oxime (**1b**), whereas the expected 5-phenylisoxazole (**6a**) was isolated in only 29% yield (Table 2, entry 1). Therefore, further optimization reactions were needed. Using 1 equivalent of oxime **1b** and 1.5 equivalents of NCS and adding 1.5 equivalents of Et_3_N in CH_2_Cl_2_ dropwise in 2 h, the conversion was very low (38%) even after 3 days. Phenylisoxazole **6a** was isolated in good (52%) yield as a sole 3,5-regioisomer and furoxan **4b** in 45% yield (yields were corrected with the conversion) (entry 2). Increasing the dosage time of the Et_3_N to 4 h resulted in 68% conversion after 1 day, which provided the corresponding product **6a** in low (37%) yield and the furoxan **4b** in higher yield (entry 3). Reducing the amount of *N*-chlorosuccinimide to a 1.1-fold excess and increasing the amount of triethylamine to 2.2 equivalents resulted in a lower conversion of 60% and a yield of 40% for **6a** (entry 4). Using a syringe pump to increase the dosage time for the Et_3_N-dichloromethane solution to 16 h resulted in better conversion (70%), with a higher yield of compound **6a** and a lower yield of 28% of furoxan **4b** (entry 5). Next, the reaction was carried out with 1.5 equivalents of NCS to achieve isoxazole **6a** in moderate yield with 76% conversion (entry 6). Increasing the equivalents of phenylacetylene (**5a**) from 1 to 1.5 did not significantly improve the conversion and the yield of the product **6a** (entry 7). Performing the reaction in the presence of 2-fold excess of *N*-chlorosuccinimide resulted in the corresponding isoxazole **6a** in good (61%) yield with 85% conversion (entry 8). Using NBS instead of NCS resulted in complete conversion but lower yield of **6a** (entry 9). Increasing the amount of dipolarophile **5a** to 2 equivalents in the presence of NCS decreased both the conversion and the yield (entry 10). The best result, 68% of **6a**, was achieved using 2 equivalents of phenylacetylene (**5a**) in the presence of 3-fold excess of NCS and 3.3-fold excess of triethylamine in dichloromethane (entry 11). The 3-(*C*-glycosyl)-4-phenylisoxazole regioisomer was not detected in the reaction mixtures.

With the resulting optimized conditions, the scope of the 1,3-dipolar cycloaddition reaction was investigated in the reaction of *C*-(2,3,4,6-tetra-*O*-acetyl-β-d-galactopyranosyl)formaldehyde oxime **1b** with a variety of alkyne dipolarophiles **5a**–**i** (Table 3). 5-(naphth-2-yl)isoxazole **6b** and 5-(naphth-1-yl)isoxazole **6c** were obtained in good yields (56% and 57%, respectively) with 2- (**5b**) and 1-ethynylnaphthalene (**5c**) (entries 2 and 3). 1,3-dipolar cycloaddition with 2-ethynylpyridine (**5d**) gave the isoxazole **6d** in 63% yield (entry 4). The reaction with 1,4-diethynylbenzene (**5e**) resulted in compound **6e** with moderate yield and the disaccharide product was not observed (entry 5). The 5-substituted 3-(galactopyranosyl)isoxazoles **6f** and **6g** were obtained in moderate yields with propargyl alcohol (**5f**) and propargyl acetate (**5g**) (entries 6 and 7). Only furoxan **4b** was obtained in the case of 3,7-anhydro-4,5,6,8-tetra-*O*-benzyl-1,2-dideoxy-d-*glycero*-d-*gulo*-oct-1-ynitol (**5h**) [20] (entry 8). The reaction with diethyl acetylenedicarboxylate (**5i**) resulted in the corresponding product **6i** in moderate yield (entry 9).

Extending the 1,3-dipolar cycloaddition reactions to *C*-(2,3,4,6-tetra-*O*-benzoyl-β-d-glucopyranosyl)formaldehyde oxime **1a**, the corresponding 5-substituted 3-(glucopyranosyl)isoxazoles **7a**,**d**–**I** were isolated in low to good yields (Table 4). The transformation with phenylacetylene (**5a**), 2-ethynylpyridine (**5d**), and 1,4-diethynylbenzene (**5e**) furnished the isoxazoles **7a**,**d** and **7e** in low to moderate yields (entries 1–3) but the yield of **7a** (57%) was better then in the literature process (49%) [16] (entry 1). The cycloaddition reactions with propargyl alcohol (**5f**) and propargyl acetate (**5g**) gave compounds **7f** and **7g** in good (**7f**: 67%) and moderate (**7g**: 47%) yields (entries 4 and 5). When carrying out the reaction with the sugar derivative (**5h**) [20], 3-(glucopyranosyl)-5-(glucopyranosyl)isoxazole **7h** was isolated in low (16%) yield beside diglucosylfuroxan **4a** (entry 6). The 3,4,5-trisubstituted isoxazole **7i** was obtained in moderate (50%) yield (entry 7).

The 1,3-dipolar cycloaddition reactions of *C*-(2,3,4,6-tetra-*O*-acetyl-β-d-galactopyranosyl)formaldehyde oxime **1b** with alkene dipolarophiles **2a**–**g** were carried out using our optimized conditions (Table 2, entry 11) and the results are summarized in Table 5. The reactions with styrene (**2a**) and 2-vinylnaphthalene (**2b**) produced isoxazolines **8a** and **8b** with good yields with 1.1:1 diastereomeric ratios (entries 1 and 2). The isoxazoline derivative **8c** was obtained in the presence of vinyl acetate (**2c**) in a moderate yield of 56% with a diastereomeric ratio of 1.6:1 (entry 3). However, the transformation with *cis*-stilbene (**2d**) furnished only furoxan **4b** (entry 4). Cycloaddition with *trans*-stilbene (**2e**) gave **8e** as the sole isomer, albeit in low yield, and furoxan **4b** as the major product (entry 5). Neither 2-methoxyprop-1-ene (**2f**) nor cyclohexene (**2g**) gave the expected products, only furoxan **4b** was formed in the transformations (entries 6 and 7).

Cycloaddition reactions of *C*-(2,3,4,6-tetra-*O*-benzoyl-β-d-glucopyranosyl)formaldehyde oxime **1a** with alkene dipolarophiles **2a**–**c, f** were also performed (see Table 6). Using the optimized conditions (Table 2, entry 11), the corresponding products **3a**–**c** were isolated in good to excellent yields with diastereomeric ratios of 1.1–1.3:1, respectively (entries 1–3). A complex reaction mixture was obtained with 2-methoxyprop-1-ene (**2f**) (entry 4).

**Scheme 2:**
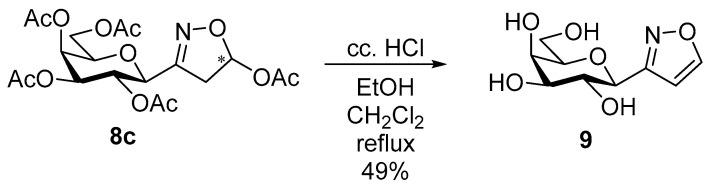
Synthesis of 3-(β-d-galactopyranosyl)isoxazole **9** from [3-(2′,3′,4′,6′-tetra-*O*-acetyl-β-d-galactopyranosyl)isoxazol-5-yl] acetates **8c**.

To obtain (glycopyranosyl)isoxazoles **6** and **7** from (glycopyranosyl)isoxazolines **3** and **8**, the literature methods were tested. Bromination of the isoxazoline **8a** followed by elimination of hydrogen bromide [21] or oxidation with manganese(IV) oxide (MnO_2_) [22] with **8b** in dry toluene at reflux temperature using a Dean–Stark apparatus failed. Finally, acetic acid was eliminated from **8c** using a literature method [23] to obtain isoxazole **9** in a moderate yield of 49% (Scheme 2). However, a complex reaction mixture formed in the case of the benzoylated **3c**.

**Scheme 3:**
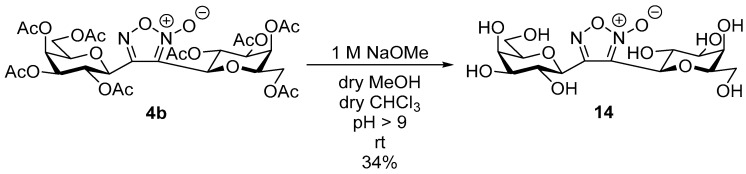
Deprotection of 3,4-di(β-d-galactopyranosyl)-1,2,5-oxadiazole-2-oxide **4b**.

Finally, the deprotection of 5-substituted 3-(2,3,4,6-tetra-*O*-acyl-β-d-glycopyranosyl)isoxazoles **6a**–**d** and **7a**,**d**,**f**, -isoxazolines **8a**,**b** and **3a**,**b**, and furoxan **4b** was achieved by the Zemplén-method using a catalytic amount of NaOMe in dry MeOH at room temperature (Table 7 and Table 8, Scheme 3). The isoxazole **10a**–**d** and **11a**,**d**,**f** and isoxazoline **12a**,**b** and **13a**,**b** and furoxan **14** derivatives were isolated in low to excellent yields.

### 2.2. Glycogen Phosphorylase Assays

The inhibition of hepatic glycogen phosphorylase (GP), the rate-determining enzyme for the degradation of the storage polysaccharide glycogen, can reduce the hepatic glucose output and may directly influence blood glucose levels [24]. Furthermore, glycogen phosphorylase inhibitors can support the proliferation and function of pancreatic beta cells [25,26]. Thus, GP inhibition has become a validated target in finding new therapeutic possibilities for type 2 diabetes [27,28,29,30]. The most populated class of inhibitors of GP is that of the glucose analogues, which primarily bind to the active site of the enzyme.

Phenyl isoxazole **11a** was tested as an inhibitor of GP and showed no significant binding [16]. However, the 5-(pyridin-2-yl)isoxazole **11d** and isoxazolylmethanol **11f** derivatives and the isoxazolines **13a**,**b** were not investigated in GP inhibition.

Some of the newly synthesized glucose derivatives were assayed against rabbit muscle glycogen phosphorylase *b* (RMGP*b*) according to previously described protocols [31]. Compared to previous molecules, isoxazoles **11d**,**f** and isoxazolines **13a**,**b** did not inhibit the GP enzyme.

### 2.3. Cell Proliferation Assays

**Figure 1:**
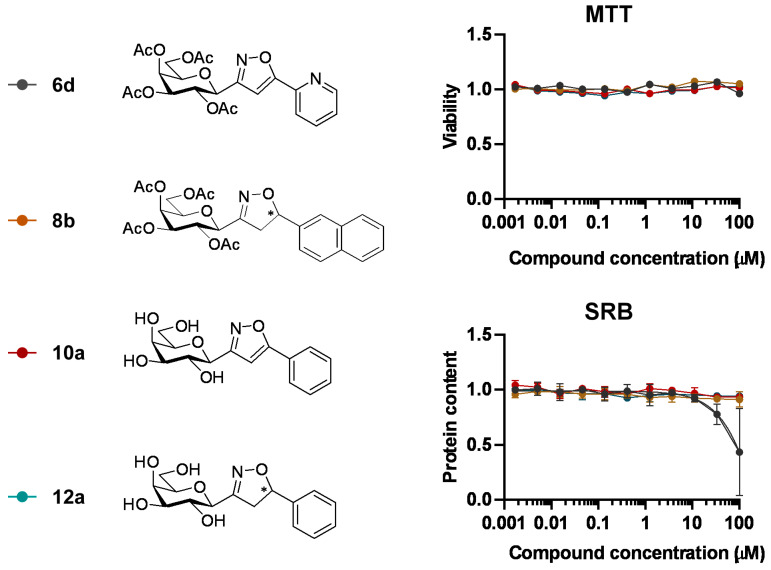
Cytostatic properties of compounds **6d**, **8b**, **10a**, and **12a** on A2780 cells.

Toxicity or the inhibition of cell proliferation are pre-requisites for application as cancer chemotherapy agents. A subset of the compounds (**6d**, **8b**, **10a**, **12a**) was assessed for rapid toxicity (MTT assay) and for the inhibition of cell proliferation (SRB assay), as described previously on human ovarian adenocarcinoma cell model (A2780) [32]. Compounds **8b**, **10a**, and **12a** did not induce rapid toxicity or cytostasis (Figure 1). However, **6d** induced cytostasis at 100 µM with a ~50% maximal inhibition without inducing rapid toxicity (Figure 1).

For MTT assays 4 × 10^3^ and for SRB assays 2 × 10^3^, A2780 cells were plated to 96-well plates. The cells were treated with the compounds in the concentrations indicated for either 4 h for an MTT assay or for 48 h for an SRB assay. Data is represented as average ± SD, from three biological replicates, and individual assays were performed in duplicates. Values were normalized for vehicle-treated cells; absorbance for vehicle-treated cells equals to 1. Normality was assessed using the Shapiro–Wilk test. Statistical significance was assessed using one-way ANOVA or Kruskal–Wallis test as a function of normality followed by Holm–Sidak’s, Dunett’s, or Dunn’s post hoc test. For better visibility normality, statistical tests and *p* values are presented in an excel sheet at https://figshare.com/s/2261964aba80a88bd606, accessed on 9 July 2025. Nonlinear regression was performed on the datasets.

## 3. Materials and Methods

### 3.1. Synthesis

#### 3.1.1. General Methods

Optical rotations were determined with a Jasco P-2000 (Easton, MD, USA) polarimeter at room temperature. NMR spectra were recorded with Bruker AM Avance DRX 360 MHz (360/90 MHz for ^1^H/^13^C) or Bruker AM Avance I 400 MHz (400/100 MHz for ^1^H/^13^C) or Bruker AM Avance II 500 MHz (500/125 MHz for ^1^H/^13^C) or Bruker Avance Neo 700 MHz (700/175 MHz for ^1^H/^13^C) spectrometers (Bruker, Karlsruhe, Germany). Chemical shifts are referenced to TMS as the internal reference (^1^H), or to the residual solvent signals (^1^H and ^13^C). The assignments of the ^1^H and ^13^C NMR signals of compounds **3**, **4**, **6**, **7**, **8**, **9**, **10**, **11**, **12**, **13**, and **14** were performed by their COSY (**3a**–**c**, **4a**,**b**, **6a**,**d**,**g**,**i**, **7a**,**d**–**i**, **8a**–**c**,**e**, **9**, **10b**, **11a**,**d**,**f**, **12a**,**b**, **13a**,**b**, and **14**), HSQC (**3a**–**c**, **4a**,**b**, **6a**,**d**,**g**,**i**, **7a**,**d**–**i**, **8a**–**c**,**e**, **9**, **10b**, **11a**,**d**,**f**, **12a**,**b**, **13a**,**b**, and **14**) and HMBC (**3a**–**c**, **4a**,**b**, **6a**,**d**,**g**,**i**, **7a**,**d**–**i**, **8a**–**c**,**e**, **9**, **10b**, **11a**,**d**,**f**, **12a**,**b**, **13a**,**b**, and **14**) spectra. Mass spectra were recorded with maXis II UHR ESI-QTOF MS (Bruker Daltonik, Bremen, Germany) instruments in positive ion mode with the electrospray ionization technique or Thermo LTQ XL (Thermo Electron Corp., San Jose, CA, USA) mass spectrometers operated in a full scan positive and negative ion ESI mode. TLC was performed on DCAlurolle Kieselgel 60 F254 (Merck & Co., Inc., Rahway, NJ, USA). TLC plates were visualized under UV light and by gentle heating (generally no spray reagent was used but, if more intense charring was necessary, the plate was sprayed with the following solution: abs. EtOH (95 mL), cc. H_2_SO_4_ (5 mL), anisaldehyde (1 mL)). For column chromatography, Kieselgel 60 (Merck & Co., Inc., Rahway, NJ, USA, particle size (0.063–0.200 mm) was applied.

#### 3.1.2. General Procedure I for Synthesis of 5-Substituted and 4,5-Disubstituted 3-(2′,3′,4′,6′-Tetra-*O*-acyl-β-d-glycopyranosyl)isoxazoles and -isoxazolines (**3**, **6**, **7**, **8**)

A *C*-(2,3,4,6-tetra-*O*-acyl-β-d-glycopyranosyl)formaldehyde oxime (3,4,5,7-tetra-*O*-acyl-2,6-anhydro-heptose oxime) (**1a** and **1b** 1 mmol), *N*-chlorosuccinimide (3 mmol), and dipolarophile (alkene or alkyne (2 mmol)) were added to dry dichloromethane (17 mL). The suspension was stirred for 30 min at room temperature, and then a solution of dry trimethylamine (3.3 mmol) in dry dichloromethane (40 mL) was added dropwise with a syringe pump in 16 h. When TLC (1:2 EtOAc–hexane for **1a**, 1:1 EtOAc–hexane for **1b**) indicated complete consumption of the starting compound (~16 h), the solvent was removed under reduced pressure, and the residue was purified by silica gel column chromatography with eluents indicated for the particular compounds to give 5-substituted and 4,5-disubstituted 3-(2′,3′,4′,6′-tetra-*O*-acyl-β-d-glycopyranosyl)isoxazoles **6** and **7** and -isoxazolines **3** and **8**.

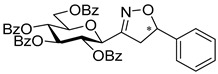



##### 3-(2′,3′,4′,6′-Tetra-*O*-benzoyl-β-d-glucopyranosyl)-5-phenylisoxazolines (**3a**)

Prepared from oxime **1a** (0.10 g, 0.16 mmol) and ethenylbenzene **2a** (2 equiv., 36.7 µL, 0.03 g, 0.32 mmol) according to the General Procedure I. Purified by column chromatography (1:3 EtOAc–hexane) to yield 89 mg (77%) of (diastereomeric ratio: 1.3:1) as a white amorphous product. R_f_: 0.26 (1:2 EtOAc–hexane). **3a-I** ^1^H NMR (500 MHz, CDCl_3_) δ (ppm) 8.14–7.75 (8H, m, Ar), 7.64–7.14 (17H, m, Ar), 6.03 (1H, pseudo t, *J*_3′,4′_ 9.5 Hz, H-3′), 5.72 (1H, pseudo t, *J*_4′,5′_ 9.4 Hz, H-4′), 5.63–5.52 (2H, m, H-5, H-2′), 4.78 (1H, d, *J*_1′,2′_ 9.9 Hz, H-1′), 4.61 (1H, dd, *J*_6a′,6b′_ 12.3 Hz, H-6_a_′), 4.47 (1H, dd, H-6_b_′), 4.23 (1H, ddd, *J*_5′,6a′_ 2.3, *J*_5′,6b′_ 5.2 Hz, H-5′), 3.68 (1H, dd, *J*_4a,4b_ 17.0, *J*_4a,5_ 10.9 Hz, H-4_a_), 3.13 (1H, dd, *J*_4b,5_ 9.2 Hz, H-4_b_). ^13^C NMR (125 MHz, CDCl_3_) δ (ppm) 166.2, 165.9, 165.8, 165.4 (4 × CO), 155.2 (C-3), 140.4–125.9 (Ar), 83.1 (C-5), 76.6 (C-5′), 74.6 (C-1′), 73.7 (C-3′), 70.1 (C-2′), 69.5 (C-4′), 63.2 (C-6′), 40.7 (C-4). **3a-II** ^1^H NMR (500 MHz, CDCl_3_) δ (ppm) 8.14–7.75 (8H, m, Ar), 7.64–7.14 (17H, m, Ar), 6.00 (1H, pseudo t, *J*_3′,4′_ 9.5 Hz, H-3′), 5.70 (1H, pseudo t, *J*_4′,5′_ 9.4 Hz, H-4′), 5.63–5.52 (1H, m, H-5), 5.55 (1H, pseudo t, *J*_2′,3′_ 9.7 Hz, H-2′), 4.82 (1H, d, *J*_1′,2′_ 10.0 Hz, H-1′), 4.65 (1H, dd, *J*_6a′,6b′_ 12.3 Hz, H-6_a_′), 4.47 (1H, dd, H-6_b_′), 4.23 (1H, ddd, *J*_5′,6a′_ 1.9, *J*_5′,6b′_ 5.2 Hz, H-5′), 3.55 (1H, dd, *J*_4a,4b_ 17.2, *J*_4a,5_ 11.3 Hz, H-4_a_), 3.26 (1H, dd, *J*_4b,5_ 9.2 Hz, H-4_b_). ^13^C NMR (125 MHz, CDCl_3_) δ (ppm) 166.2, 165.9, 165.8, 165.4 (4 × CO), 154.8 (C-3), 140.4–125.9 (Ar), 83.1 (C-5), 76.7 (C-5′), 74.6 (C-1′), 73.9 (C-3′), 69.6 (C-2′), 69.4 (C-4′), 63.2 (C-6′), 40.7 (C-4). HR-ESI-MS positive mode (*m*/*z*): calcd. for C_43_H_35_NO_10_ (725.23) [M + Na]^+^ = 748.2153, found: [M + Na]^+^ = 748.2152.

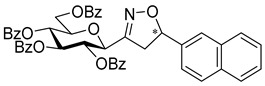



##### 3-(2′,3′,4′,6′-Tetra-*O*-benzoyl-β-d-glucopyranosyl)-5-(naphth-2-yl)isoxazolines (**3b**)

Prepared from oxime **1a** (0.10 g, 0.16 mmol) and 2-ethenylnaphthalene **2b** (2 equiv., 0.05 g, 0.32 mmol) according to the General Procedure I. Purified by column chromatography (1:3 EtOAc–hexane) to yield 116 mg (93%) of **3b** (diastereomeric ratio: 1:1) as a yellow amorphous product. R_f_: 0.23 (1:2 EtOAc–hexane). **3b-I** ^1^H NMR (500 MHz, CDCl_3_) δ (ppm) 8.15–7.63 (12H, m, Ar), 7.62–7.16 (15H, m, Ar), 6.05 (1H, pseudo t, *J*_3′,4′_ 9.6 Hz, H-3′), 5.81–5.73 (1H, m, H-5), 5.73 (1H, pseudo t, *J*_4′,5′_ 10.0 Hz, H-4′), 5.62 (1H, pseudo t, *J*_2′,3′_ 9.9 Hz, H-2′), 4.81 (1H, d, *J*_1′,2′_ 9.9 Hz, H-1′), 4.61 (1H, dd, *J*_6a′,6b′_ 12.3 Hz, H-6_a_′), 4.48 (1H, dd, H-6_b_′), 4.25 (1H, ddd, *J*_5′,6a′_ 2.4, *J*_5′,6b′_ 4.9 Hz, H-5′), 3.75 (1H, dd, *J*_4a,4b_ 17.1, *J*_4a,5_ 11.0 Hz, H-4_a_), 3.21 (1H, dd, *J*_4b,5_ 9.2 Hz, H-4_b_). ^13^C NMR (125 MHz, CDCl_3_) δ (ppm) 166.2, 165.9, 165.8, 165.4 (4 × CO), 155.3 (C-3), 137.6–123.7 (Ar), 83.2 (C-5), 76.6 (C-5′), 74.6 (C-1′), 73.7 (C-3′), 70.2 (C-2′), 69.5 (C-4′), 63.1 (C-6′), 40.8 (C-4). **3b-II** ^1^H NMR (500 MHz, CDCl_3_) δ (ppm) 8.15–7.63 (12H, m, Ar), 7.62–7.16 (15H, m, Ar), 6.01 (1H, pseudo t, *J*_3′,4′_ 9.6 Hz, H-3′), 5.81–5.73 (1H, m, H-5), 5.71 (1H, pseudo t, *J*_4′,5′_ 10.0 Hz, H-4′), 5.57 (1H, pseudo t, *J*_2′,3′_ 9.7 Hz, H-2′), 4.85 (1H, d, *J*_1′,2′_ 10.0 Hz, H-1′), 4.66 (1H, dd, *J*_6a′,6b′_ 12.2 Hz, H-6_a_′), 4.46 (1H, dd, H-6_b_′), 4.25 (1H, ddd, *J*_5′,6a′_ 2.1, *J*_5′,6b′_ 4.9 Hz, H-5′), 3.63 (1H, dd, *J*_4a,4b_ 17.1, *J*_4a,5_ 11.2 Hz, H-4_a_), 3.37 (1H, dd, *J*_4b,5_ 9.0 Hz, H-4_b_). ^13^C NMR (125 MHz, CDCl_3_) δ (ppm) 166.3, 165.9, 165.4, 165.3 (4 × CO), 154.9 (C-3), 137.6–123.7 (Ar), 83.1 (C-5), 76.7 (C-5′), 74.6 (C-1′), 73.8 (C-3′), 69.6 (C-2′), 69.4 (C-4′), 63.1 (C-6′), 40.8 (C-4). HR-ESI-MS positive mode (*m*/*z*): calcd. for C_47_H_37_NO_10_ (775.24) [M + Na]^+^ = 798.2310, found: [M + Na]^+^ = 798.2310.

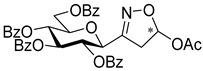



##### [3-(2′,3′,4′,6′-Tetra-*O*-benzoyl-β-d-glucopyranosyl)isoxazol-5-yl] acetates (**3c**)

Prepared from oxime **1a** (0.10 g, 0.16 mmol) and ethenyl acetate **2c** (2 equiv., 29.6 µL, 0.03 g, 0.32 mmol) according to the General Procedure I. Purified by column chromatography (1:3 EtOAc–hexane) to yield 67 mg (59%) of **3c** (diastereomeric ratio: 1.1:1) as a white amorphous product. R_f_: 0.36 (1:1 EtOAc–hexane). **3c-I** ^1^H NMR (500 MHz, CDCl_3_) δ (ppm) 8.08–7.74 (8H, m, Ar), 7.62–7.12 (12H, m, Ar), 6.72 (1H, d, H-5), 6.03 (1H, pseudo t, *J*_3′,4′_ 9.6 Hz, H-3′), 5.76 (1H, pseudo t, *J*_4′,5′_ 9.9 Hz, H-4′), 5.49 (1H, pseudo t, *J*_2′,3′_ 9.7 Hz, H-2′), 4.81 (1H, d, *J*_1′,2′_ 9.9 Hz, H-1′), 4.66 (1H, dd, H-6_a_′), 4.51 (1H, dd, *J*_6a′,6b′_ 12.3 Hz, H-6_b_′), 4.25 (1H, ddd, *J*_5′,6a′_ 2.4, *J*_5′,6b′_ 4.8 Hz, H-5′), 3.56 (1H, dd, *J*_4a,4b_ 18.3, *J*_4a,5_ 7.2 Hz, H-4_a_), 3.13 (1H, dd, *J*_4b,5_ < 1.0 Hz, H-4_b_), 2.03 (3H, s, CH_3_). ^13^C NMR (125 MHz, CDCl_3_) δ (ppm) 169.7 (O*CO*CH_3_), 166.2, 165.8, 165.3 (4 × CO), 156.3 (C-3), 134.1–128.1 (Ar), 95.8 (C-5), 76.7 (C-5′), 74.2 (C-1′), 73.5 (C-3′), 69.9 (C-2′), 69.3 (C-4′), 63.0 (C-6′), 39.3 (C-4), 21.1 (CH_3_). **3c-II** ^1^H NMR (500 MHz, CDCl_3_) δ (ppm) 8.08–7.74 (8H, m, Ar), 7.62–7.12 (12H, m, Ar), 6.63 (1H, d, H-5), 5.99 (1H, pseudo t, *J*_3′,4′_ 9.6 Hz, H-3′), 5.74 (1H, pseudo t, *J*_4′,5′_ 9.9 Hz, H-4′), 5.67 (1H, pseudo t, *J*_2′,3′_ 9.7 Hz, H-2′), 4.87 (1H, d, *J*_1′,2′_ 10.0 Hz, H-1′), 4.66 (1H, dd, H-6_a_′), 4.46 (1H, dd, *J*_6a′,6b′_ 12.3 Hz, H-6_b_′), 4.25 (1H, ddd, *J*_5′,6a′_ 2.4, *J*_5′,6b′_ 5.2 Hz, H-5′), 3.35 (1H, dd, *J*_4a,4b_ 18.2, *J*_4a,5_ 6.7 Hz, H-4_a_), 3.20 (1H, dd, *J*_4b,5_ < 1.0 Hz, H-4_b_), 1.82 (3H, s, CH_3_). ^13^C NMR (125 MHz, CDCl_3_) δ (ppm) 169.7 (O*CO*CH_3_), 166.2, 165.8, 165.3 (4 × CO), 155.7 (C-3), 134.1–128.1 (Ar), 95.7 (C-5), 76.9 (C-5′), 74.0 (C-1′), 73.8 (C-3′), 69.7 (C-2′), 69.4 (C-4′), 63.0 (C-6′), 39.3 (C-4), 20.7 (CH_3_). HR-ESI-MS positive mode (*m*/*z*): calcd. for C_39_H_33_NO_12_ (707.20) [M + Na]^+^ = 730.1895, found: [M + Na]^+^ = 730.1895.

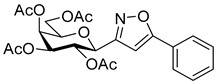



##### 3-(2′,3′,4′,6′-Tetra-*O*-acetyl-β-d-galactopyranosyl)-5-phenylisoxazole (**6a**)

Prepared from oxime **1b** (0.10 g, 0.27 mmol) and ethynylbenzene **5a** (2 equiv., 59.7 µL, 0.06 g, 0.53 mmol) according to the General Procedure I. Purified by column chromatography (1:2 EtOAc–hexane) to yield 86 mg (68%) of **6a** as a yellow amorphous product. R_f_: 0.59 (1:1 EtOAc–hexane); [α]_D_−16 (*c* 0.18, CH_2_Cl_2_). ^1^H NMR (500 MHz, CDCl_3_) δ (ppm) 7.86–7.73 (2H, m, Ar), 7.52–7.39 (3H, m, Ar), 6.68 (1H, s, H-4), 5.55 (1H, dd, *J*_4′,5′_ 0.9 Hz, H-4′), 5.47 (1H, pseudo t, *J*_2′,3′_ 10.1 Hz, H-2′), 5.21 (1H, dd, *J*_3′,4′_ 3.4 Hz, H-3′), 4.69 (1H, d, *J*_1′,2′_ 9.9 Hz, H-1′), 4.24–4.14 (2H, m, H-6_a_′, H-6_b_′), 4.12 (1H, ddd, *J*_5′,6a′_ 6.1, *J*_5′,6b′_ 6.7 Hz, H-5′), 2.21, 2.05, 2.01, 1.97 (12H, 4s, 4 × CH_3_). ^13^C NMR (90 MHz, CDCl_3_) δ (ppm) 170.7, 170.3, 170.2, 169.6 (4 × CO), 170.6 (C-5), 161.5 (C-3), 130.9–125.6 (Ar), 97.9 (C-4), 75.1 (C-5′), 73.5 (C-1′), 71.9 (C-3′), 67.8 (C-2′), 67.7 (C-4′), 61.8 (C-6′), 20.82, 20.78, 20.72, 20.68 (4 × CH_3_). HR-ESI-MS positive mode (*m*/*z*): calcd. for C_23_H_25_NO_10_ (475.15) [M + Na]^+^ = 498.1371, found: [M + Na]^+^ = 498.1370.

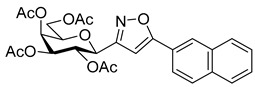



##### 3-(2′,3′,4′,6′-Tetra-*O*-acetyl-β-d-galactopyranosyl)-5-(naphth-2-yl)isoxazole (**6b**)

Prepared from oxime **1b** (0.10 g, 0.27 mmol) and 2-ethynylnaphthalene **5b** (2 equiv., 0.08 g, 0.53 mmol) according to the General Procedure I. Purified by column chromatography (1:1 EtOAc–hexane) to yield 78 mg (56%) of **6b** as a yellow amorphous product. R_f_: 0.35 (1:1 EtOAc–hexane); [α]_D_−23 (*c* 0.23, CH_2_Cl_2_). ^1^H NMR (500 MHz, CDCl_3_) δ (ppm) 8.31 (1H, bs, Ar), 7.91 (2H, d, *J* 8.1 Hz, Ar), 7.88–7.79 (2H, m, Ar), 7.57–7.51 (2H, m, Ar), 6.80 (1H, s, H-4), 5.57 (1H, dd, *J*_4′,5′_ 0.5 Hz, H-4′), 5.50 (1H, pseudo t, *J*_2′,3′_ 10.1 Hz, H-2′), 5.23 (1H, dd, *J*_3′,4′_ 3.3 Hz, H-3′), 4.72 (1H, d, *J*_1′,2′_ 9.9 Hz, H-1′), 4.25–4.07 (3H, m, H-5′, H-6_a_′, H-6_b_′), 2.23, 2.06, 2.01, 1.99 (12H, 4s, 4 × CH_3_). ^13^C NMR (125 MHz, CDCl_3_) δ (ppm) 170.7, 170.5, 170.3, 170.2, 169.6 (4 × CO, C-5), 161.6 (C-3), 134.2–122.8 (Ar), 98.3 (C-4), 75.1 (C-5′), 73.5 (C-1′), 71.9 (C-3′), 67.8 (C-2′), 67.7 (C-4′), 61.8 (C-6′), 20.82, 20.81, 20.80, 20.71 (4 × CH_3_). HR-ESI-MS positive mode (*m*/*z*): calcd. for C_27_H_27_NO_10_ (525.16) [M + H]^+^ = 526.1708, found: [M + H]^+^ = 526.1702.

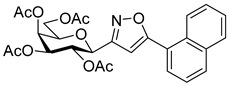



##### 3-(2′,3′,4′,6′-Tetra-*O*-acetyl-β-d-galactopyranosyl)-5-(naphth-1-yl)isoxazole (**6c**)

Prepared from oxime **1b** (0.10 g, 0.27 mmol) and 2-ethynylnaphthalene **5c** (2 equiv., 0.08 g, 0.53 mmol) according to the General Procedure I. Purified by column chromatography (1:2 EtOAc–hexane) to yield 80 mg (57%) of **6c** as a pale yellow amorphous product. R_f_: 0.41 (1:1 EtOAc–hexane); [α]_D_−17 (*c* 0.11, CH_2_Cl_2_). ^1^H NMR (700 MHz, CDCl_3_) δ (ppm) 8.28 (1H, d, *J* 8.4 Hz, Ar), 7.97 (1H, d, *J* 8.2 Hz, Ar), 7.92 (1H, d, *J* 8.1 Hz, Ar), 7.81 (1H, dd, *J* 1.0, 7.1 Hz, Ar), 7.61 (1H, ddd, *J* 1.2, 6.8, 8.3 Hz, Ar), 7.59–7.48 (2H, m, Ar), 6.77 (1H, s, H-4), 5.57 (1H, dd, *J*_4′,5′_ 0.8 Hz, H-4′), 5.55 (1H, pseudo t, *J*_2′,3′_ 10.1 Hz, H-2′), 5.25 (1H, dd, *J*_3′,4′_ 3.5 Hz, H-3′), 4.76 (1H, d, *J*_1′,2′_ 9.9 Hz, H-1′), 4.23–4.17 (2H, m, H-6_a_′, H-6_b_′), 4.17–4.13 (1H, m, H-5′), 2.21, 2.06, 2.02, 2.01 (12H, 4s, 4 × CH_3_). ^13^C NMR (175 MHz, CDCl_3_) δ (ppm) 170.7, 170.6, 170.4, 170.2, 169.7 (4 × CO, C-5), 161.1 (C-3), 134.0–124.9 (Ar), 102.1 (C-4), 75.2 (C-5′), 73.6 (C-1′), 71.9 (C-3′), 67.9 (C-2′), 67.7 (C-4′), 61.8 (C-6′), 20.87, 20.84, 20.83, 20.75 (4 × CH_3_). HR-ESI-MS positive mode (*m*/*z*): calcd. for C_27_H_27_NO_10_ (525.16) [M + H]^+^ = 526.1708, found: [M + H]^+^ = 526.1713.

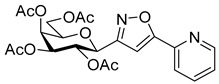



##### 3-(2′,3′,4′,6′-Tetra-*O*-acetyl-β-d-galactopyranosyl)-5-(pyridin-2-yl)isoxazole (**6d**)

Prepared from oxime **1b** (0.10 g, 0.27 mmol) and 2-ethynylpyridine **5d** (2 equiv., 56.1 µL, 0.05 g, 0.53 mmol) according to the General Procedure I. Purified by column chromatography (1:1 EtOAc–hexane) to yield 80 mg (63%) of **6d** as a pale orange amorphous product. R_f_: 0.57 (1:1 EtOAc–hexane); [α]_D_−21 (*c* 0.19, CH_2_Cl_2_). ^1^H NMR (400 MHz, CDCl_3_) δ (ppm) 8.75–8.64 (1H, m, Ar), 7.93–7.77 (2H, m, Ar), 7.41–7.30 (1H, m, Ar), 7.09 (1H, s, H-4), 5.54 (1H, dd, *J*_4′,5′_ 0.9 Hz, H-4′), 5.46 (1H, pseudo t, *J*_2′,3′_ 10.1 Hz, H-2′), 5.21 (1H, dd, *J*_3′,4′_ 3.3 Hz, H-3′), 4.71 (1H, d, *J*_1′,2′_ 9.9 Hz, H-1′), 4.21–4.07 (3H, m, H-5′, H-6_a_′, H-6_b_′), 2.20, 2.05, 2.01, 1.98 (12H, 4s, 4 × CH_3_). ^13^C NMR (100 MHz, CDCl_3_) δ (ppm) 170.6, 170.4, 170.2, 169.5 (4 × CO), 169.7 (C-5), 162.0 (C-3), 177.9–120.6 (Ar), 101.0 (C-4), 74.9 (C-5′), 73.2 (C-1′), 71.8 (C-3′), 67.8 (C-2′), 67.6 (C-4′), 61.9 (C-6′), 20.77, 20.76, 20.73, 20.69 (4 × CH_3_). HR-ESI-MS positive mode (*m*/*z*): calcd. for C_22_H_24_N_2_O_10_ (476.14) [M + H]^+^ = 477.1504, found: [M + H]^+^ = 477.1504.

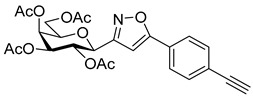



##### 3-(2′,3′,4′,6′-Tetra-*O*-acetyl-β-d-galactopyranosyl)-5-(4-ethynylphenyl)isoxazole (**6e**)

Prepared from oxime **1b** (0.10 g, 0.27 mmol) and 1,4-diethynylbenzene **5e** (2 equiv., 0.07 g, 0.53 mmol) according to the General Procedure I. Purified by column chromatography (1.2:1 EtOAc–hexane) to yield 55 mg (41%) of **6e** as a yellow amorphous product. R_f_: 0.58 (1:1 EtOAc–hexane); [α]_D_−37 (*c* 0.18, CH_2_Cl_2_). ^1^H NMR (500 MHz, CDCl_3_) δ (ppm) 7.75 (2H, d, *J* 8.3 Hz, Ar), 7.58 (2H, d, *J* 8.4 Hz, Ar), 6.70 (1H, s, H-4), 5.55 (1H, dd, *J*_4′,5′_ 0.9 Hz, H-4′), 5.45 (1H, pseudo t, *J*_2′,3′_ 10.1 Hz, H-2′), 5.20 (1H, dd, *J*_3′,4′_ 3.4 Hz, H-3′), 4.68 (1H, d, *J*_1′,2′_ 10.0 Hz, H-1′), 4.21–4.15 (2H, m, H-6_a_′, H-6_b_′), 4.11 (1H, ddd, *J*_5′,6a′_ 6.7, *J*_5′,6b′_ 6.6 Hz, H-5′), 3.21 (1H, s, CC*H*), 2.21, 2.05, 2.01, 1.98 (12H, 4s, 4 × CH_3_). ^13^C NMR (125 MHz, CDCl_3_) δ (ppm) 170.6, 170.3, 170.2, 169.7 (4 × CO), 169.7 (C-5), 161.7 (C-3), 133.0–124.2 (Ar), 98.7 (C-4), 83.0 (*C*CH), 79.6 (C*C*H), 75.1 (C-5′), 73.5 (C-1′), 71.8 (C-3′), 67.7 (C-2′, C-4′), 61.8 (C-6′), 20.85 (2), 20.81, 20.75 (4 × CH_3_). HR-ESI-MS positive mode (*m*/*z*): calcd. for C_25_H_25_NO_10_ (499.47). [M + H]^+^ = 500.1551, found: [M + H]^+^ = 500.1552.

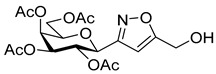



##### [3-(2′,3′,4′,6′-Tetra-*O*-acetyl-β-d-galactopyranosyl)isoxazol-5-yl]methanol (**6f**)

Prepared from oxime **1b** (0.10 g, 0.27 mmol) and prop-2-yn-1-ol **5f** (2 equiv., 28.8 µL, 0.03 g, 0.53 mmol) according to the General Procedure I. Purified by column chromatography (from 1:3 to 1:1 EtOAc–hexane) to yield 54 mg (47%) of **6f** as a pale orange amorphous product. R_f_: 0.15 (1:1 EtOAc–hexane); [α]_D_−0.1 (*c* 0.15, CH_2_Cl_2_). ^1^H NMR (500 MHz, CDCl_3_) δ (ppm) 6.42 (1H, s, H-4), 5.52 (1H, dd, *J*_4′,5′_ 0.6 Hz, H-4′), 5.38 (1H, pseudo t, *J*_2′,3′_ 10.2 Hz, H-2′), 5.18 (1H, dd, *J*_3′,4′_ 2.8 Hz, H-3′), 4.76 (2H, s, C*H*_2_OH), 4.63 (1H, d, *J*_1′,2′_ 9.9 Hz, H-1′), 4.18–4.12 (2H, m, H-6_a_′, H-6_b_′), 4.08 (1H, ddd, *J*_5′,6a′_ 6.1, *J*_5′,6b′_ 6.2 Hz, H-5′), 2.38 (1H, bs, CH_2_O*H*), 2.19, 2.05, 2.00, 1.96 (12H, 4s, 4 × CH_3_). ^13^C NMR (125 MHz, CDCl_3_) δ (ppm) 172.1 (C-5), 170.6, 170.3, 170.2, 169.8 (4 × CO), 161.0 (C-3), 100.5 (C-4), 75.0 (C-5′), 73.3 (C-1′), 71.8 (C-3′), 67.8 (C-2′), 67.6 (C-4′), 61.8 (C-6′), 56.7 (CH_2_OH), 20.83, 20.81, 20.79, 20.73 (4 × CH_3_). HR-ESI-MS positive mode (*m*/*z*): calcd. for C_18_H_23_NO_11_ (429.13) [M + H]^+^ = 430.1344, found: [M + H]^+^ = 430.1343.

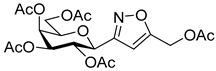



##### [3-(2′,3′,4′,6′-Tetra-*O*-acetyl-β-d-galactopyranosyl)isoxazol-5-yl]methyl acetate (**6g**)

Prepared from oxime **1b** (0.10 g, 0.27 mmol) and prop-2-yn-1-yl acetate **5g** (2 equiv., 52.9 µL, 0.05 g, 0.53 mmol) according to the General Procedure I. Purified by column chromatography (from 1:2 to 1:0 EtOAc–hexane) to yield 58 mg (46%) of **6g** as a pale yellow amorphous product. R_f_: 0.55 (2:1 EtOAc–hexane); [α]_D_−5 (*c* 0.22, CH_2_Cl_2_). ^1^H NMR (500 MHz, CDCl_3_) δ (ppm) 6.46 (1H, s, H-4), 5.52 (1H, dd, *J*_4′,5′_ 0.4 Hz, H-4′), 5.37 (1H, pseudo t, *J*_2′,3′_ 10.1 Hz, H-2′), 5.19 (1H, d, *J*_CHa,CHb_ 13.3 Hz, C*H_a_*OAc), 5.18 (1H, dd, *J*_3′,4′_ 3.2 Hz, H-3′), 5.15 (1H, d, C*H_b_*OAc), 4.64 (1H, d, *J*_1′,2′_ 9.9 Hz, H-1′), 4.19–4.11 (2H, m, H-6_a_′, H-6_b_′), 4.08 (1H, ddd, *J*_5′,6a′_ 6.1, *J*_5′,6b′_ 6.2 Hz, H-5′), 2.14 (3H, s, CH_2_OCOC*H*_3_), 2.19, 2.05, 2.00, 1.96 (12H, 4s, 4 × CH_3_). ^13^C NMR (125 MHz, CDCl_3_) δ (ppm) 170.3 (CH_2_O*C*OCH_3_), 170.5, 170.2, 170.1, 169.9 (4 × CO), 167.4 (C-5), 161.1 (C-3), 102.6 (C-4), 75.0 (C-5′), 73.2 (C-1′), 71.7 (C-3′), 67.7 (C-2′), 67.5 (C-4′), 61.7 (C-6′), 56.4 (*C*H_2_OAc), 20.78, 20.77, 20.72, 20.69, 20.68 (5×CH_3_). HR-ESI-MS positive mode (*m*/*z*): calcd. for C_20_H_25_NO_12_ (471.42) [M + Na]^+^ = 494.1269, found: [M + Na]^+^ = 494.1268.

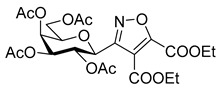



##### Diethyl 3-(2′,3′,4′,6′-Tetra-*O*-acetyl-β-d-galactopyranosyl)isoxazole-4,5-dicarboxylate (**6i**)

Prepared from oxime **1b** (0.10 g, 0.27 mmol) and diethyl but-2-ynedioate **5i** (2 equiv., 85.3 µL, 0.09 g, 0.53 mmol) according to the General Procedure I. Purified by column chromatography (from 1:2 to 2:1 EtOAc–hexane) to yield 64 mg (44%) of **6i** as a pale yellow amorphous product. R_f_: 0.30 (1:1 EtOAc–hexane); [α]_D_+2 (*c* 0.33, CH_2_Cl_2_). ^1^H NMR (500 MHz, CDCl_3_) δ (ppm) 5.77 (1H, pseudo t, *J*_2′,3′_ 10.2 Hz, H-2′), 5.50 (1H, dd, *J*_4′,5′_ 0.6 Hz, H-4′), 5.17 (1H, dd, *J*_3′,4′_ 3.3 Hz, H-3′), 4.88 (1H, d, *J*_1′,2′_ 10.0 Hz, H-1′), 4.49–4.32 (4H, m, 2 × C*H*_2_CH_3_) 4.15–4.04 (3H, m, H-5′, H-6_a_′, H-6_b_′), 2.19, 2.04, 2.01, 1.96 (12H, 4s, 4 × CH_3_), 1.40 (6H, 2 × t, *J* 7.1 Hz, 2 × CH_2_*CH*_3_). ^13^C NMR (125 MHz, CDCl_3_) δ (ppm) 170.5, 170.4, 170.3, 169.2 (4 × CO), 160.1 (C-5, COOEt), 159.0 (C-3), 156.0 (COOEt), 115.7 (C-4), 75.2 (C-5′), 72.7 (C-1′), 72.0 (C-3′), 67.5 (C-4′), 67.2 (C-2′), 63.1, 62.2 (2 × *C*H_2_CH_3_), 61.6 (C-6′), 20.78, 20.75, 20.74 (2) (4 × CH_3_), 14.2, 14.1 (2 × CH_2_*CH*_3_). HR-ESI-MS positive mode (*m*/*z*): calcd. for C_23_H_29_NO_14_ (543.16) [M + H]^+^ = 544.1661, found: [M + H]^+^ = 544.1664.

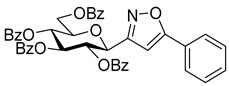



##### 3-(2′,3′,4′,6′-Tetra-*O*-benzoyl-β-d-glucopyranosyl)-5-phenylisoxazole (**7a**)

Prepared from oxime **1a** (0.10 g, 0.16 mmol) and ethynylbenzene **5a** (2 equiv., 35.2 µL, 0.03 g, 0.32 mmol) according to the General Procedure I. Purified by column chromatography (from 1:3 to 1:2 EtOAc–hexane) to yield 67 mg (57%) of **7a** as a yellow amorphous product. R_f_: 0.34 (1:2 EtOAc–hexane). ^1^H NMR (500 MHz, CDCl_3_) δ (ppm) 8.14–7.68 (10H, m, Ar), 7.64–7.20 (15H, m, Ar), 6.72 (1H, s, H-4), 6.05 (1H, pseudo t, *J*_3′,4′_ 9.5 Hz, H-3′), 5.84 (1H, pseudo t, *J*_4′,5′_ 9.5 Hz, H-4′), 5.82 (1H, pseudo t, *J*_2′,3′_ 9.4 Hz, H-2′), 5.10 (1H, d, *J*_1′,2′_ 10.0 Hz, H-1′), 4.69 (1H, dd, *J*_6a′,6b′_ 12.3 Hz, H-6_a_′), 4.53 (1H, dd, H-6_b_′), 4.35 (1H, ddd, *J*_5′,6a′_ 2.6, *J*_5′,6b′_ 5.1 Hz, H-5′). ^13^C NMR (125 MHz, CDCl_3_) δ (ppm) 170.8 (C-5), 166.3, 165.9, 165.4, 165.2 (4 × CO), 161.2 (C-3), 134.9–125.6 (Ar), 97.8 (C-4), 76.9 (C-5′), 74.3 (C-3′), 73.5 (C-1′), 71.3 (C-2′), 69.6 (C-4′), 63.3 (C-6′). C_43_H_33_NO_10_ (723.21). NMR spectra are identical with those reported [16].

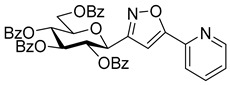



##### 3-(2′,3′,4′,6′-Tetra-*O*-benzoyl-β-d-glucopyranosyl)-5-(pyridin-2-yl)isoxazole (**7d**)

Prepared from oxime **1a** (0.10 g, 0.16 mmol) and 2-ethynylpyridine **5d** (2 equiv., 32.4 µL, 0.03 g, 0.32 mmol) according to the General Procedure I. Purified by column chromatography (1:3 EtOAc–hexane) to yield 46 mg (39%) of **7d** as a pale orange amorphous product. R_f_: 0.16 (1:2 EtOAc–hexane); [α]_D_−57 (*c* 0.22, CH_2_Cl_2_). ^1^H NMR (500 MHz, CDCl_3_) δ (ppm) 8.67 (1H, d, *J* 4.8 Hz, Ar), 8.08–7.71 (10H, m, Ar), 7.59–7.22 (13H, m, Ar), 7.12 (1H, s, H-4), 6.05 (1H, pseudo t, *J*_3′,4′_ 9.5 Hz, H-3′), 5.84 (1H, pseudo t, *J*_4′,5′_ 9.4 Hz, H-4′), 5.82 (1H, pseudo t, *J*_2′,3′_ 9.6 Hz, H-2′), 5.12 (1H, d, *J*_1′,2′_ 10.0 Hz, H-1′), 4.67 (1H, dd, *J*_6a′,6b′_ 12.3 Hz, H-6_a_′), 4.53 (1H, dd, H-6_b_′), 4.35 (1H, ddd, *J*_5′,6a′_ 2.5, *J*_5′,6b′_ 5.1 Hz, H-5′). ^13^C NMR (125 MHz, CDCl_3_) δ (ppm) 170.0 (C-5), 166.3, 166.0, 165.4, 165.1 (4 × CO), 161.6 (C-3), 150.3–120.7 (Ar), 100.8 (C-4), 76.9 (C-5′), 74.3 (C-3′), 73.5 (C-1′), 71.3 (C-2′), 69.6 (C-4′), 63.4 (C-6′). HR-ESI-MS positive mode (*m*/*z*): calcd. for C_42_H_32_N_2_O_10_ (724.21) [M + Na]^+^ = 747.1949, found: [M + Na]^+^ = 747.1946.

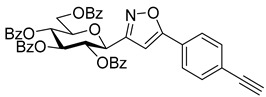



##### 3-(2′,3′,4′,6′-Tetra-*O*-benzoyl-β-d-glucopyranosyl)-5-(4-ethynylphenyl)isoxazole (**7e**)

Prepared from oxime **1a** (0.10 g, 0.16 mmol) and 1,4-diethynylbenzene **5e** (2 equiv., 0.04 g, 0.32 mmol) according to the General Procedure I. Purified by column chromatography (1:3 EtOAc–hexane) to yield 29 mg (24%) of **7e** as a yellow amorphous product. R_f_: 0.36 (1:2 EtOAc–hexane); [α]_D_−96 (*c* 0.13, CH_2_Cl_2_). ^1^H NMR (500 MHz, CDCl_3_) δ (ppm) 8.08–7.78 (8H, m, Ar), 7.68 (1H, d, *J* 8.3 Hz, Ar), 7.60–7.24 (14H, m, Ar), 6.73 (1H, s, H-4), 6.04 (1H, pseudo t, *J*_3′,4′_ 9.7 Hz, H-3′), 5.83 (1H, pseudo t, *J*_4′,5′_ 9.6 Hz, H-4′), 5.80 (1H, pseudo t, *J*_2′,3′_ 9.7 Hz, H-2′), 5.09 (1H, d, *J*_1′,2′_ 10.0 Hz, H-1′), 4.69 (1H, dd, *J*_6a′,6b′_ 12.3 Hz, H-6_a_′), 4.52 (1H, dd, H-6_b_′), 4.34 (1H, ddd, *J*_5′,6a′_ 2.8, *J*_5′,6b′_ 5.1 Hz, H-5′), 3.19 (1H, s, CC*H*). ^13^C NMR (125 MHz, CDCl_3_) δ (ppm) 169.8 (C-5), 166.3, 165.9, 165.4, 165.2 (4 × CO), 161.3 (C-3), 135.5–124.0 (Ar), 98.5 (C-4), 83.0 (*C*CH), 79.5 (C*C*H), 77.0 (C-5′), 74.2 (C-3′), 73.4 (C-1′), 71.2 (C-2′), 69.6 (C-4′), 63.3 (C-6′). HR-ESI-MS positive mode (*m*/*z*): calcd. for C_42_H_32_N_2_O_10_ (747.21) [M + Na]^+^ = 770.1997, found: [M + Na]^+^ = 770.1998.

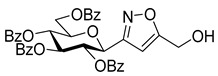



##### [3-(2′,3′,4′,6′-Tetra-*O*-benzoyl-β-d-glucopyranosyl)isoxazol-5-yl]methanol (**7f**)

Prepared from oxime **1a** (0.10 g, 0.16 mmol) and prop-2-yn-1-ol **5f** (2 equiv., 18.5 µL, 0.02 g, 0.32 mmol) according to the General Procedure I. Purified by column chromatography (1:2 EtOAc–hexane) to yield 73 mg (67%) of **7f** as a white amorphous product. R_f_: 0.18 (1:2 EtOAc–hexane); [α]_D_−3 (*c* 0.34, CH_2_Cl_2_). ^1^H NMR (500 MHz, CDCl_3_) δ (ppm) 8.10–7.77 (8H, m, Ar), 7.59–7.22 (12H, m, Ar), 6.45 (1H, s, H-4), 6.03 (1H, pseudo t, *J*_3′,4′_ 9.5 Hz, H-3′), 5.81 (1H, pseudo t, *J*_4′,5′_ 9.8 Hz, H-4′), 5.74 (1H, pseudo t, *J*_2′,3′_ 9.6 Hz, H-2′), 5.05 (1H, d, *J*_1′,2′_ 10.0 Hz, H-1′), 4.67 (2H, s, C*H*_2_OH), 4.66 (1H, dd, *J*_6a′,6b′_ 12.3 Hz, H-6_a_′), 4.50 (1H, dd, H-6_b_′), 4.32 (1H, ddd, *J*_5′,6a′_ 2.7, *J*_5′,6b′_ 5.1 Hz, H-5′), 2.57 (1H, bs, CH_2_O*H*). ^13^C NMR (125 MHz, CDCl_3_) δ (ppm) 172.4 (C-5), 166.3, 165.9, 165.4, 165.3 (4 × CO), 160.6 (C-3), 133.9–128.0 (Ar), 100.3 (C-4), 76.9 (C-5′), 74.2 (C-3′), 73.3 (C-1′), 71.3 (C-2′), 69.5 (C-4′), 63.2 (C-6′), 56.6 (CH_2_OH). HR-ESI-MS positive mode (*m*/*z*): calcd. for C_38_H_31_NO_11_ (677.19) [M + Na]^+^ = 700.1789, found: [M + Na]^+^ = 700.1789.

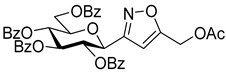



##### [3-(2′,3′,4′,6′-Tetra-*O*-benzoyl-β-d-glucopyranosyl)isoxazol-5-yl]methyl acetate (**7g**)

Prepared from oxime **1a** (0.10 g, 0.16 mmol) and prop-2-yn-1-yl acetate **5g** (2 equiv., 31.8 µL, 0.03 g, 0.32 mmol) according to the General Procedure I. Purified by column chromatography (1:3 EtOAc–hexane) to yield 54 mg (47%) of **7g** as a white amorphous product. R_f_: 0.18 (1:2 EtOAc–hexane); [α]_D_−11 (*c* 0.20, CH_2_Cl_2_). ^1^H NMR (500 MHz, CDCl_3_) δ (ppm) 8.10–7.74 (8H, m, Ar), 7.60–7.13 (12H, m, Ar), 6.51 (1H, s, H-4), 6.02 (1H, pseudo t, *J*_3′,4′_ 9.5 Hz, H-3′), 5.80 (1H, pseudo t, *J*_4′,5′_ 9.8 Hz, H-4′), 5.73 (1H, pseudo t, *J*_2′,3′_ 9.6 Hz, H-2′), 5.12 (2H, s, C*H*_2_OAc), 5.05 (1H, d, *J*_1′,2′_ 10.0 Hz, H-1′), 4.66 (1H, dd, *J*_6a′,6b′_ 12.3 Hz, H-6_a_′), 4.50 (1H, dd, H-6_b_′), 4.32 (1H, ddd, *J*_5′,6a′_ 2.8, *J*_5′,6b′_ 5.1 Hz, H-5′), 2.08 (3H, s, CH_3_). ^13^C NMR (125 MHz, CDCl_3_) δ (ppm) 170.2 (CH_2_O*C*OCH_3_), 167.6 (C-5), 166.3, 165.9, 165.3, 165.1 (4 × CO), 160.8 (C-3), 134.0–128.0 (Ar), 102.4 (C-4), 76.9 (C-5′), 74.2 (C-3′), 73.3 (C-1′), 71.2 (C-2′), 69.5 (C-4′), 63.2 (C-6′), 56.4 (CH_2_OAc), 20.7 (CH_3_). HR-ESI-MS positive mode (*m*/*z*): calcd. for C_40_H_33_NO_12_ (719.20) [M + Na]^+^ = 742.1895, found: [M + Na]^+^ = 742.1892.

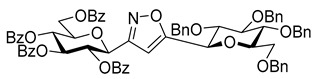



##### 3-(2′,3′,4′,6′-Tetra-*O*-benzoyl-β-d-glucopyranosyl)-5-(2″,3″,4″,6″-tetra-*O*-benzyl-β-d-glucopyranosyl)isoxazole (**7h**)

Prepared from oxime **1a** (0.05 g, 0.08 mmol) and 2-*C*-(2′,3′,4′,6′-tetra-*O*-benzyl-β-d-glucopyranosyl)ethyne (3,7-anhydro-4,5,6,8-tetra-*O*-benzyl-1,2-dideoxy-d-*glycero*-d-*gulo*-oct-1-ynitol) **5h** [20] (2 equiv., 0.09 g, 0.16 mmol) according to the General Procedure I. Purified by column chromatography (from 1:4 to 1:2 EtOAc–hexane) to yield 15 mg (16%) of **7h** as a white amorphous product. R_f_: 0.35 (1:2 EtOAc–hexane); [α]_D_−5 (*c* 0.10, CH_2_Cl_2_). ^1^H NMR (500 MHz, CDCl_3_) δ (ppm) 8.09–7.69 (8H, m, Ar), 7.61–6.99 (32H, m, Ar), 6.57 (1H, s, H-4), 6.02 (1H, pseudo t, *J*_3′,4′_ 9.6 Hz, H-3′), 5.79 (1H, pseudo t, *J*_4′,5′_ 9.7 Hz, H-4′), 5.73 (1H, pseudo t, *J*_2′,3′_ 9.7 Hz, H-2′), 5.07 (1H, d, *J*_1′,2′_ 10.0 Hz, H-1′), 4.87–4.77 (3H, m, CH_2_), 4.65 (1H, dd, *J*_6a′,6b′_ 12.3 Hz, H-6_a_′), 4.61–4.47 (3H, m, CH_2_), 4.51 (1H, dd, H-6_b_′), 4.44 (1H, d, *J*_1″,2″_ 8.9 Hz, H-1″), 4.32 (1H, ddd, *J*_5′,6a′_ 2.7, *J*_5′,6b′_ 4.6 Hz, H-5′), 4.31 (1H, d, *J* 10.5 Hz, CH_2_), 4.14 (1H, d, *J* 10.5 Hz, CH_2_), 3.79–3.60 (5H, m, H-2″, H-3″, H-4″, H-6_a_″, H-6_b_″), 3.54 (1H, ddd, *J*_4′,5′_ 9.6, *J*_5″,6a″_ 2.0, *J*_5″,6b″_ 3.6 Hz, H-5″). ^13^C NMR (125 MHz, CDCl_3_) δ (ppm) 170.3 (C-5), 166.3, 165.9, 165.4, 165.0 (4 × CO), 160.7 (C-3), 138.7–127.4 (Ar), 102.1 (C-4), 86.4 (C-3″), 81.2 (C-2″), 79.9 (C-5″), 77.8 (C-4″), 76.7 (C-5′), 75.7, 75.3, 75.0 (3×CH_2_), 74.3 (C-3′), 73.7 (C-1″, CH_2_), 73.5 (C-1′), 71.3 (C-2′), 69.6 (C-4′), 68.9 (C-6″), 63.4 (C-6′). HR-ESI-MS positive mode (*m*/*z*): calcd. for C_71_H_63_NO_15_ (1169.42) [M + Na]^+^ = 1192.4090, found: [M + Na]^+^ = 1192.4093.

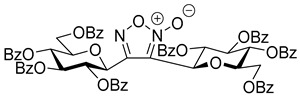



##### 3,4-Di(2,3,4,6-Tetra-*O*-benzoyl-β-d-glucopyranosyl)-1,2,5-oxadiazole-2-oxide (**4a**)

Prepared from the previous reaction mixture from oxime **1a** (0.05 g, 0.08 mmol) and 2-*C*-(2′,3′,4′,6′-tetra-*O*-benzyl-β-d-glucopyranosyl)ethyne (3,7-anhydro-4,5,6,8-tetra-*O*-benzyl-1,2-dideoxy-d-*glycero*-d-*gulo*-oct-1-ynitol) **5h** [20] (2 equiv., 0.09 g, 0.16 mmol) according to the General Procedure I. Purified by column chromatography (from 1:4 to 1:2 EtOAc–hexane) to yield 15 mg (31%) of **4a** as a white amorphous product. R_f_: 0.29 (1:2 EtOAc–hexane); [α]_D_−18 (*c* 0.14, CH_2_Cl_2_). ^1^H NMR (500 MHz, CDCl_3_) δ (ppm) 8.12–7.89 (8H, m, Ar), 7.87–7.70 (8H, m, Ar), 7.59–7.09 (24H, m, Ar), 6.18–6.09 (3H, m, H-2′, H-3′, H-3″), 6.07 (1H, pseudo t, *J*_2″,3″_ 9.5 Hz, H-2″), 5.79 (1H, pseudo t, *J*_3′,4′_ 9.7, *J*_4′,5′_ 10.2 Hz, H-4′), 5.77 (1H, pseudo t, *J*_3″,4″_ 9.7, *J*_4″,5″_ 9.9 Hz, H-4″), 5.26 (1H, d, *J*_1′,2′_ 10.1 Hz, H-1′), 5.17 (1H, d, *J*_1″,2″_ 9.4 Hz, H-1″), 4.87–4.81 (2H, m, H-6_a_′, H-6_a_″), 4.81 (1H, dd, H-6_b_′), 4.75 (1H, dd, *J*_6a″,6b″_ 12.8 Hz, H-6_b_″), 4.47 (1H, ddd, *J*_5′,6a′_ 2.8, *J*_5′,6b′_ 6.9 Hz, H-5′), 4.43 (1H, ddd, *J*_5″,6a″_ 2.7, *J*_5″,6b″_ 7.4 Hz, H-5″). ^13^C NMR (125 MHz, CDCl_3_) δ (ppm) 166.2, 165.9, 165.8, 165.4, 165.0, 164.9 (8×CO), 153.7 (C-4), 134.0–128.1 (Ar), 112.9 (C-3), 77.6 (C-5′, C-5″), 74.4 (C-1″), 73.7 (C-3′), 73.5 (C-3″), 72.0 (C-1′), 71.2 (C-2′), 71.0 (C-2″), 70.0 (C-4′), 69.9 (C-4″), 63.8 (C-6′, C-6″). HR-ESI-MS positive mode (*m*/*z*): calcd. for C_70_H_54_N_2_O_20_ (1242.33). [M + Na]^+^ = 1265.3162, found: [M + Na]^+^ = 1265.3161.

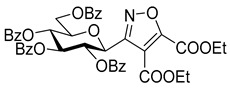



##### Diethyl 3-(2′,3′,4′,6′-Tetra-*O*-benzoyl-β-d-glucopyranosyl)isoxazole-4,5-dicarboxylate (**7i**)

Prepared from oxime **1a** (0.10 g, 0.16 mmol) and diethyl but-2-ynedioate **5i** (2 equiv., 51.3 µL, 0.05 g, 0.32 mmol) according to the General Procedure I. Purified by column chromatography (1:2 EtOAc–hexane) to yield 64 mg (50%) of **7i** as a pale yellow amorphous product. R_f_: 0.42 (1:1 EtOAc–hexane); [α]_D_−3 (*c* 0.58, CH_2_Cl_2_). ^1^H NMR (500 MHz, CDCl_3_) δ (ppm) 8.11–7.74 (8H, m, Ar), 7.58–7.16 (12H, m, Ar), 6.22 (1H, pseudo t, *J*_2′,3′_ 9.7 Hz, H-2′), 6.02 (1H, pseudo t, *J*_3′,4′_ 9.5 Hz, H-3′), 5.82 (1H, pseudo t, *J*_4′,5′_ 9.7 Hz, H-4′), 5.27 (1H, d, *J*_1′,2′_ 10.0 Hz, H-1′), 4.62 (1H, dd, *J*_6a′,6b′_ 12.4 Hz, H-6_a_′), 4.45 (1H, dd, H-6_b_′), 4.41 (2H, q, *J* 7.2 Hz, C*H*_2_CH_3_), 4.30 (1H, ddd, *J*_5′,6a′_ 2.5, *J*_5′,6b′_ 5.1 Hz, H-5′), 4.29–4.18 (2H, m, C*H*_2_CH_3_), 1.37, 1.30 (6H, 2 × t, *J* 7.1 Hz, 2 × CH_2_C*H*_3_). ^13^C NMR (125 MHz, CDCl_3_) δ (ppm) 166.3, 166.0, 165.3, 164.9 (4 × CO), 160.8 (C-5), 160.2 (COOEt), 159.0 (C-3), 156.1 (COOEt), 133.7–128.4 (Ar), 115.1 (C-4), 77.0 (C-5′), 74.3 (C-3′), 72.9 (C-1′), 70.5 (C-2′), 69.2 (C-4′), 63.1 (C-6′), 63.1, 62.3 (2 × *C*H_2_CH_3_), 14.1 (2 × CH_2_*C*H_3_). HR-ESI-MS positive mode (*m*/*z*): calcd. for C_47_H_37_NO_14_ (791.22) [M + H]^+^ = 814.2106, found: [M + H]^+^ = 814.2098.

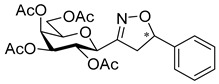



##### 3-(2′,3′,4′,6′-Tetra-*O*-acetyl-β-d-galactopyranosyl)-5-phenylisoxazolines (**8a**)

Prepared from oxime **1b** (0.10 g, 0.27 mmol) and ethenylbenzene **2a** (2 equiv., 61.1 µL, 0.06 g, 0.53 mmol) according to the General Procedure I. Purified by column chromatography (1:1 EtOAc–hexane) to yield 106 mg (83%) of **8a** (diastereomeric ratio: 1.1:1) as a yellow amorphous product. R_f_: 0.44 (1:1 EtOAc–hexane). **8a-I** ^1^H NMR (400 MHz, CDCl_3_) δ (ppm) 7.43–7.25 (5H, m, Ar), 5.59 (1H, dd, H-5), 5.49 (1H, dd, *J*_4′,5′_ 0.5 Hz, H-4′), 5.27 (1H, pseudo t, *J*_2′,3′_ 10.7 Hz, H-2′), 5.17 (1H, dd, *J*_3′,4′_ 3.3 Hz, H-3′), 4.39 (1H, d, *J*_1′,2′_ 9.6 Hz, H-1′), 4.17–4.06 (2H, m, H-6_a_′, H-6_b_′), 4.05–3.96 (1H, m, H-5′), 3.57 (1H, dd, *J*_4a,4b_ 17.2, *J*_4a,5_ 11.0 Hz, H-4_a_), 3.08 (1H, dd, *J*_4b,5_ 9.3 Hz, H-4_b_), 2.15, 2.08, 2.03, 2.01 (12H, 4s, 4 × CH_3_). ^13^C NMR (90 MHz, CDCl_3_) δ (ppm) 170.4, 170.1, 170.0 (4 × CO), 155.4 (C-3), 140.6–125.6 (Ar), 82.7 (C-5), 74.6 (C-5′), 74.4 (C-1′), 71.2 (C-3′), 67.4 (C-4′), 66.4 (C-2′), 61.5 (C-6′), 40.9 (C-4), 20.77, 20.68, 20.64 (2) (4 × CH_3_). **8a-II** ^1^H NMR (400 MHz, CDCl_3_) δ (ppm) 7.43–7.25 (5H, m, Ar), 5.57 (1H, dd, H-5), 5.48 (1H, dd, *J*_4′,5′_ 0.5 Hz, H-4′), 5.22 (1H, pseudo t, *J*_2′,3′_ 10.5 Hz, H-2′), 5.14 (1H, dd, *J*_3′,4′_ 3.3 Hz, H-3′), 4.41 (1H, d, *J*_1′,2′_ 9.3 Hz, H-1′), 4.17–4.06 (2H, m, H-6_a_′, H-6_b_′), 4.05–3.96 (1H, m, H-5′), 3.49 (1H, dd, *J*_4a,4b_ 17.3, *J*_4a,5_ 11.2 Hz, H-4_a_), 3.16 (1H, dd, *J*_4b,5_ 10.2 Hz, H-4_b_), 2.13, 2.06, 2.01, 1.98 (12H, 4s, 4 × CH_3_). ^13^C NMR (90 MHz, CDCl_3_) δ (ppm) 170.4, 170.1, 170.0, 169.9 (4 × CO), 155.4 (C-3), 140.6–125.6 (Ar), 83.0 (C-5), 74.6 (C-5′), 74.5 (C-1′), 71.4 (C-3′), 67.5 (C-4′), 66.0 (C-2′), 61.7 (C-6′), 40.9 (C-4), 20.73, 20.64 (2), 20.59 (4 × CH_3_). HR-ESI-MS positive mode (*m*/*z*): calcd. for C_23_H_27_NO_10_ (477.16) [M + H]^+^ = 478.1708, found: [M + H]^+^ = 478.1710.

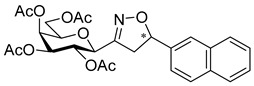



##### 3-(2′,3′,4′,6′-Tetra-*O*-acetyl-β-d-galactopyranosyl)-5-(naphth-2-yl)isoxazolines (**8b**)

Prepared from oxime **1b** (0.10 g, 0.27 mmol) and 2-ethenylnaphthalene **2b** (2 equiv., 0.08 g, 0.53 mmol) according to the General Procedure I. Purified by column chromatography (1:1 EtOAc–hexane) to yield 106 mg (83%) of **8b** (diastereomeric ratio: 1.1:1) as a pale yellow amorphous product. R_f_: 0.38 (1:1 EtOAc–hexane). **8b-I** ^1^H NMR (400 MHz, CDCl_3_) δ (ppm) 7.92–7.76 (4H, m, Ar), 7.54–7.38 (3H, m, Ar), 5.74 (1H, dd, H-5), 5.49 (1H, dd, *J*_4′,5′_ 0.5 Hz, H-4′), 5.30 (1H, pseudo t, *J*_2′,3′_ 10.5 Hz, H-2′), 5.17 (1H, dd, *J*_3′,4′_ 3.5 Hz, H-3′), 4.41 (1H, d, *J*_1′,2′_ 9.4 Hz, H-1′), 4.17–4.05 (2H, m, H-6_a_′, H-6_b_′), 4.04–3.96 (1H, m, H-5′), 3.64 (1H, dd, *J*_4a,4b_ 17.2, *J*_4a,5_ 10.9 Hz, H-4_a_), 3.16 (1H, dd, *J*_4b,5_ 9.2 Hz, H-4_b_), 2.15, 2.10, 2.01, 1.97 (12H, 4s, 4 × CH_3_). ^13^C NMR (90 MHz, CDCl_3_) δ (ppm) 170.5, 170.2, 170.0 (4 × CO), 155.6 (C-3), 137.8–123.3 (Ar), 82.9 (C-5), 74.6 (C-5′), 74.5 (C-1′), 71.3 (C-3′), 67.5 (C-4′), 66.5 (C-2′), 61.5 (C-6′), 41.0 (C-4), 20.83, 20.70, 20.67, 20.64 (4 × CH_3_). **8b-II** ^1^H NMR (400 MHz, CDCl_3_) δ (ppm) 7.92–7.76 (4H, m, Ar), 7.54–7.38 (3H, m, Ar), 5.77 (1H, dd, H-5), 5.48 (1H, dd, *J*_4′,5′_ 0.5 Hz, H-4′), 5.24 (1H, pseudo t, *J*_2′,3′_ 10.3 Hz, H-2′), 5.14 (1H, dd, *J*_3′,4′_ 3.4 Hz, H-3′), 4.44 (1H, d, *J*_1′,2′_ 8.9 Hz, H-1′), 4.17–4.05 (2H, m, H-6_a_′, H-6_b_′), 4.04–3.96 (1H, m, H-5′), 3.56 (1H, dd, *J*_4a,4b_ 17.4, *J*_4a,5_ 11.2 Hz, H-4_a_), 3.26 (1H, dd, *J*_4b,5_ 9.8 Hz, H-4_b_), 2.12, 2.06, 2.01, 1.97 (12H, 4s, 4 × CH_3_). ^13^C NMR (90 MHz, CDCl_3_) δ (ppm) 170.5, 170.4, 170.0, 169.9 (4 × CO), 155.5 (C-3), 137.8–123.3 (Ar), 83.1 (C-5), 74.7 (C-5′), 74.6 (C-1′), 71.5 (C-3′), 67.5 (C-4′), 66.1 (C-2′), 61.7 (C-6′), 40.9 (C-4), 20.77, 20.70, 20.67, 20.61 (4 × CH_3_). HR-ESI-MS positive mode (*m*/*z*): calcd. for C_27_H_29_NO_10_ (527.18) [M + H]^+^ = 528.1684, found: [M + H]^+^ = 528.1682.

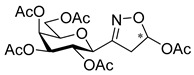



##### [3-(2′,3′,4′,6′-Tetra-*O*-acetyl-β-d-galactopyranosyl)isoxazol-5-yl] acetates (**8c**)

Prepared from oxime **1b** (0.10 g, 0.27 mmol) and ethenyl acetate **2c** (2 equiv., 49.1 µL, 0.05 g, 0.53 mmol) according to the General Procedure I. Purified by column chromatography (from 1:2 to 2:1 EtOAc–hexane) to yield 65 mg (53%) of **8c** (diastereomeric ratio: 1.6:1) as a yellow amorphous product. R_f_: 0.42 (2:1 EtOAc–hexane). **8c-I** ^1^H NMR (500 MHz, CDCl_3_) δ (ppm) 6.70 (1H, dd, H-5), 5.52–5.48 (1H, m, H-4′), 5.20–5.12 (2H, m, H-2′, H-3′), 4.45–4.39 (1H, m, H-1′), 4.19–4.08 (2H, m, H-6_a_′, H-6_b_′), 4.04 (1H, ddd, *J*_5′,6a′_ 6.1, *J*_5′,6b′_ 6.3 Hz, H-5′), 3.43 (1H, dd, *J*_4a,4b_ 18.3, *J*_4a,5_ 7.1 Hz, H-4_a_), 3.09 (1H, dd, *J*_4b,5_ < 1.0 Hz, H-4_b_), 2.08 (3H, s, CH_2_OCOC*H*_3_), 2.17, 2.06, 2.02, 2.00 (12H, 4s, 4 × CH_3_). ^13^C NMR (125 MHz, CDCl_3_) δ (ppm) 170.5, 170.3, 170.1, 170.0 (4 × CO), 169.7 (O*CO*CH_3_), 156.6 (C-3), 95.8 (C-5), 74.7 (C-5′), 74.0 (C-1′), 71.1 (C-3′), 67.3 (C-4′), 66.2 (C-2′), 61.4 (C-6′), 39.5 (C-4), 21.07, 20.96, 20.74, 20.69, 20.62 (5 × CH_3_). **8c-II** ^1^H NMR (500 MHz, CDCl_3_) δ (ppm) 6.65 (1H, dd, H-5), 5.52–5.48 (1H, m, H-4′), 5.32 (1H, pseudo t, *J*_2′,3′_ 10.1 Hz, H-2′), 5.15 (1H, dd, *J*_3′,4′_ 3.3 Hz, H-3′), 4.46 (1H, d, *J*_1′,2′_ 9.8 Hz, H-1′), 4.19–4.08 (2H, m, H-6_a_′, H-6_b_′), 4.03 (1H, ddd, *J*_5′,6a′_ 6.2, *J*_5′,6b′_ 6.0 Hz, H-5′), 3.32 (1H, dd, *J*_4a,4b_ 18.5, *J*_4a,5_ 6.9 Hz, H-4_a_), 3.13 (1H, dd, *J*_4b,5_ < 1.0 Hz, H-4_b_), 2.08 (3H, s, CH_2_OCOC*H*_3_), 2.18, 2.05, 2.04, 2.00 (12H, 4s, 4 × CH_3_). ^13^C NMR (125 MHz, CDCl_3_) δ (ppm) 170.5, 170.1, 170.0, 169.5 (4 × CO), 169.8 (O*CO*CH_3_), 155.9 (C-3), 95.9 (C-5), 74.9 (C-5′), 73.9 (C-1′), 71.3 (C-3′), 67.5 (C-4′), 66.1 (C-2′), 61.6 (C-6′), 39.8 (C-4), 21.07, 20.96, 20.74, 20.69, 20.62 (5×CH_3_). HR-ESI-MS positive mode (*m*/*z*): calcd. for C_19_H_25_NO_12_ (459.40) [M + Na]^+^ = 482.1269, found: [M + Na]^+^ = 482.1269.

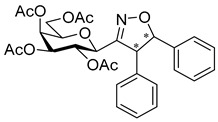



##### 3-(2′,3′,4′,6′-Tetra-*O*-acetyl-β-d-galactopyranosyl)-4,5-diphenylisoxazoline (**8e**)

Prepared from oxime **1b** (0.10 g, 0.27 mmol) and (*E*)-1,2-diphenylethene **2e** (2 equiv., 0.10 g, 0.53 mmol) according to the General Procedure I. Purified by column chromatography (from 1:3 to 1:1 EtOAc–hexane) to yield 33 mg (23%) of **8e** as a colourless amorphous product. R_f_: 0.60 (2:1 EtOAc–hexane); [α]_D_+76 (*c* 0.59, CH_2_Cl_2_). ^1^H NMR (500 MHz, CDCl_3_) δ (ppm) 7.39–7.29 (6H, m, Ar), 7.29–7.23 (4H, m, Ar), 5.54 (1H, d, H-5), 5.42 (1H, pseudo t, *J*_2′,3′_ 10.0 Hz, H-2′), 5.29 (1H, dd, *J*_4′,5′_ 0.4 Hz, H-4′), 4.97 (1H, dd, *J*_3′,4′_ 3.3 Hz, H-3′), 4.46 (1H, d, *J*_4,5_ 7.9 Hz, H-4), 4.27 (1H, d, *J*_1′,2′_ 10.0 Hz, H-1′), 3.76–3.67 (3H, m, H-5′, H-6_a_′, H-6_b_′), 2.05, 2.01, 1.98, 1.95 (12H, 4s, 4 × CH_3_). ^13^C NMR (125 MHz, CDCl_3_) δ (ppm) 170.4, 170.2, 169.6 (4 × CO), 155.9 (C-3), 139.9–125.5 (Ar), 91.7 (C-5), 74.2 (C-5′), 73.6 (C-1′), 72.0 (C-3′), 67.4 (C-4′), 66.1 (C-2′), 62.4 (C-4), 61.4 (C-6′), 20.86, 20.78, 20.73, 20.72 (4 × CH_3_). HR-ESI-MS positive mode (*m*/*z*): calcd. for C_29_H_31_NO_10_ (553.56) [M + Na]^+^ = 576.1840, found: [M + Na]^+^ = 576.1841.

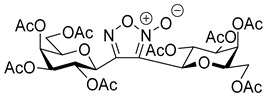



##### 3,4-Di(2,3,4,6-Tetra-*O*-acetyl-β-d-galactopyranosyl)-1,2,5-oxadiazole-2-oxide (**4b**)

Prepared from the previous reaction mixture from oxime **1b** (0.10 g, 0.27 mmol) and (*E*)-1,2-diphenylethene **2e** (2 equiv., 0.10 g, 0.53 mmol) according to the General Procedure I. Purified by column chromatography (from 1:3 to 1:1 EtOAc–hexane) to yield 76 mg (76%) of **4b** as a yellow amorphous product. R_f_: 0.47 (2:1 EtOAc–hexane). ^1^H NMR (500 MHz, CDCl_3_) δ (ppm) 5.65 (1H, pseudo t, *J*_2′,3′_ 10.0 Hz, H-2′), 5.58 (1H, pseudo t, *J*_2″,3″_ 10.0 Hz, H-2″), 5.55 (1H, dd, *J*_4′,5′_ 0.7 Hz, H-4′), 5.51 (1H, dd, *J*_4″,5″_ 0.7 Hz, H-4″), 5.19 (1H, dd, *J*_3′,4′_ 3.3 Hz, H-3′), 5.17 (1H, dd, *J*_3″,4″_ 3.3 Hz, H-3″), 4.84 (1H, d, *J*_1′,2′_ 10.1 Hz, H-1′), 4.82 (1H, d, *J*_1″,2″_ 10.1 Hz, H-1″), 4.31–4.01 (6H, m, H-5′, H-5″, H-6_a_′, H-6_a_″, H-6_b_′, H-6_b_″), 2.24, 2.06, 2.05, 2.02, 2.00, 1.99 (24H, 8s, 8 × CH_3_). ^13^C NMR (125 MHz, CDCl_3_) δ (ppm) 170.4, 170.2, 170.1, 170.0, 169.5, 169.2 (8 × CO), 153.3 (C-4), 111.7 (C-3), 75.4 (C-5″), 75.2 (C-5′), 72.9 (C-1″), 71.7 (C-3′, C-3″), 70.8 (C-1′), 67.3 (C-4′, C-4″), 66.9 (C-2″), 65.9 (C-2′), 61.4 (C-6′, C-6″), 20.77, 20.74, 20.70 (2), 20.61 (2), 20.59, 20.49 (8 × CH_3_). NMR spectra are identical with those reported [14].

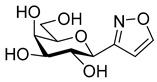



#### 3.1.3. Synthesis of 3-(β-d-Galactopyranosyl)isoxazole (**9**)

[3-(2′,3′,4′,6′-Tetra-*O*-acetyl-β-d-galactopyranosyl)isoxazol-5-yl] acetates **8c** (0.04 g, 0.09 mmol) were dissolved in dichloromethane (0.5 mL) and ethanol (0.5 mL). Then cc. HCl (36 µL) was added to the mixture. The solution was stirred and heated to reflux temperature. When TLC (5:1 CHCl_3_–MeOH) indicated complete consumption of the starting compound (~3 h), the mixture was cooled and the solvent was removed under reduced pressure, and the residue was purified by column chromatography (5:1 CHCl_3_–MeOH) to yield 10 mg (49%) of **9** as a colourless amorphous product. R_f_: 0.17 (4:1 CHCl_3_–MeOH); [α]_D_+42 (*c* 0.05, MeOH). ^1^H NMR (500 MHz, CD_3_OD) δ (ppm) 8.62 (1H, s, H-5), 6.67 (1H, s, H-4), 4.36 (1H, d, *J*_1′,2′_ 9.7 Hz, H-1′), 3.96 (1H, bs, H-4′), 3.81 (1H, pseudo t, *J*_2′,3′_ 9.5 Hz, H-2′), 3.77 (1H, dd, *J*_5′,6b′_ 7.3, *J*_6a′,6b′_ 11.1 Hz, H-6_a_′), 3.73–3.64 (2H, m, H-5′, H-6_b_′), 3.59 (1H, dd, *J*_3′,4′_ 3.4 Hz, H-3′). ^13^C NMR (125 MHz, CD_3_OD) δ (ppm) 163.3 (C-3), 160.3 (C-5), 104.4 (C-4), 81.1 (C-5′), 76.3 (C-1′), 76.1 (C-3′), 71.5 (C-2′), 70.8 (C-4′), 62.8 (C-6′). ESI-MS negative mode (*m*/*z*): calcd. for C_9_H_13_NO_6_ (231.07) [M − H]^−^ = 230.0670, found: [M − H]^−^ = 229.99.

#### 3.1.4. General Procedure II for Removal of *O*-Acyl Protecting Groups (**10**, **11**, **12**, **13**)

5-Substituted 3-(2′,3′,4′,6′-tetra-*O*-acyl-β-d-glycopyranosyl)isoxazole or -isoxazolines **3**, **6**, **7**, **8** (100 mg) were dissolved in dry MeOH (5 mL) and dry chloroform (3 mL) then a solution of NaOMe (1 M in MeOH) was added to the solution in a catalytic amount. The reaction mixture was stirred at room temperature. When the reaction was complete (TLC, 7:3 CHCl_3_–MeOH) (1–3 h), the solution was neutralized with a cation exchange resin Amberlyst 15 (H^+^ form). The resin was filtered off with suction and the filtrate was evaporated under reduced pressure. The crude product was purified by silica gel column chromatography with eluents indicated for the particular compounds to give 5-substituted 3-(β-d-glycopyranosyl)isoxazoles **10** and **11** and -isoxazolines **12** and **13**.

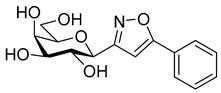



##### 3-(β-d-Galactopyranosyl)-5-phenylisoxazole (**10a**)

Prepared from isoxazole **6a** (0.06 g, 0.12 mmol) according to the General Procedure II. Purified by column chromatography (4:1 CHCl_3_–MeOH) to yield 18 mg (49%) of **10a** as a pale yellow amorphous product. R_f_: 0.56 (7:3 CHCl_3_–MeOH); [α]_D_+18 (*c* 0.12, MeOH). ^1^H NMR (700 MHz, CD_3_OD) δ (ppm) 7.83 (2H, d, *J* 7.2 Hz, Ar), 7.53–7.45 (3H, m, Ar), 6.96 (1H, s, H-4), 4.35 (1H, d, *J*_1′,2′_ 9.7 Hz, H-1′), 3.99 (1H, dd, *J*_4′,5′_ 0.6 Hz, H-4′), 3.87 (1H, pseudo t, *J*_2′,3′_ 9.4 Hz, H-2′), 3.80 (1H, dd, *J*_6a′,6b′_ 11.3 Hz, H-6_a_′), 3.73 (1H, dd, H-6_b_′), 3.70 (1H, ddd, *J*_5′,6b′_ 4.9, *J*_5′,6a′_ 6.9 Hz, H-5′), 3.62 (1H, dd, *J*_3′,4′_ 3.3 Hz, H-3′). ^13^C NMR (90 MHz, CD_3_OD) δ (ppm) 171.2 (C-5), 165.1 (C-3), 131.5–126.7 (Ar), 99.7 (C-4), 81.1 (C-5′), 76.5 (C-1′), 76.1 (C-3′), 71.5 (C-2′), 70.8 (C-4′), 62.9 (C-6′). HR-ESI-MS positive mode (*m*/*z*): calcd. for C_15_H_17_NO_6_ (307.11) [M + H]^+^ = 308.1129, found: [M + H]^+^ = 308.1129.

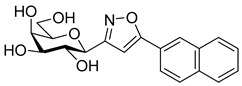



##### 3-(β-d-Galactopyranosyl)-5-(naphth-2-yl)isoxazole (**10b**)

Prepared from isoxazole **6b** (0.06 g, 0.12 mmol) according to the General Procedure II. Purified by column chromatography (6:1 CHCl_3_–MeOH) to yield 19 mg (43%) of **10b** as a white amorphous product. R_f_: 0.43 (1:1 CHCl_3_–MeOH); [α]_D_+32 (*c* 0.10, MeOH). ^1^H NMR (700 MHz, CD_3_OD) δ (ppm) 8.38 (1H, bs, Ar), 8.01–7.96 (1H, m, Ar), 7.99 (1H, d, *J* 8.4 Hz, Ar), 7.93–7.88 (1H, m, Ar), 7.90 (1H, dd, *J* 1.7, 8.5 Hz, Ar), 7.60–7.54 (2H, m, Ar), 7.08 (1H, s, H-4), 4.38 (1H, d, *J*_1′,2′_ 9.7 Hz, H-1′), 4.00 (1H, dd, *J*_4′,5′_ 0.4 Hz, H-4′), 3.90 (1H, pseudo t, *J*_2′,3′_ 9.4 Hz, H-2′), 3.81 (1H, dd, *J*_6a′,6b′_ 11.3 Hz, H-6_a_′), 3.74 (1H, dd, H-6_b_′), 3.72 (1H, ddd, *J*_5′,6a′_ 4.9, *J*_5′,6b′_ 6.8 Hz, H-5′), 3.63 (1H, dd, *J*_3′,4′_ 3.3 Hz, H-3′). ^13^C NMR (175 MHz, CD_3_OD) δ (ppm) 171.3 (C-5), 165.2 (C-3), 135.5–123.8 (Ar), 100.2 (C-4), 81.2 (C-5′), 76.6 (C-1′), 76.1 (C-3′), 71.6 (C-2′), 70.9 (C-4′), 62.9 (C-6′). HR-ESI-MS positive mode (*m*/*z*): calcd. for C_19_H_19_NO_6_ (357.12) [M + Na]^+^ = 380.1105, found: [M + Na]^+^ = 380.1106.

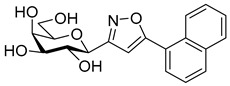



##### 3-(β-d-Galactopyranosyl)-5-(naphth-1-yl)isoxazole (**10c**)

Prepared from isoxazole **6c** (0.05 g, 0.10 mmol) according to the General Procedure II. Purified by column chromatography (3.5:1 CHCl_3_–MeOH) to yield 26 mg (75%) of **10c** as a white amorphous product. R_f_: 0.46 (7:3 CHCl_3_–MeOH); [α]_D_+21 (*c* 0.10, MeOH). ^1^H NMR (500 MHz, CD_3_OD) δ (ppm) 8.31 (1H, d, *J* 8.1 Hz, Ar), 8.02 (1H, d, *J* 8.2 Hz, Ar), 7.97 (1H, dd, *J* 1.9, 7.5 Hz, Ar), 7.83 (1H, dd, *J* 1.1, 7.2 Hz, Ar), 7.63–7.54 (3H, m, Ar), 7.02 (1H, s, H-4), 4.44 (1H, d, *J*_1′,2′_ 9.7 Hz, H-1′), 4.01 (1H, dd, *J*_4′,5′_ 0.5 Hz, H-4′), 3.95 (1H, pseudo t, *J*_2′,3′_ 9.4 Hz, H-2′), 3.83 (1H, dd, *J*_5′,6a′_ 8.3, *J*_6a′,6b′_ 12.6 Hz, H-6_a_′), 3.79–3.71 (2H, m, H-5′, H-6_b_′), 3.66 (1H, dd, *J*_3′,4′_ 3.3 Hz, H-3′). ^13^C NMR (125 MHz, CD_3_OD) δ (ppm) 171.2 (C-5), 164.8 (C-3), 135.5–125.7 (Ar), 103.8 (C-4), 81.2 (C-5′), 76.6 (C-1′), 76.1 (C-3′), 71.6 (C-2′), 70.9 (C-4′), 62.9 (C-6′). HR-ESI-MS positive mode (*m*/*z*): calcd. for C_19_H_19_NO_6_ (357.12) [M + Na]^+^ = 380.1105, found: [M + Na]^+^ = 380.1104.

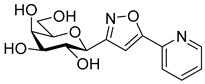



##### 3-(β-d-Galactopyranosyl)-5-(pyridin-2-yl)isoxazole (**10d**)

Prepared from isoxazole **6d** (0.11 g, 0.23 mmol) according to the General Procedure II. Purified by column chromatography (7:3 CHCl_3_–MeOH) to yield 45 mg (63%) of **10d** as a white amorphous product. R_f_: 0.36 (4:1 CHCl_3_–MeOH); [α]_D_+28 (*c* 0.20, MeOH). ^1^H NMR (700 MHz, CD_3_OD) δ (ppm) 8.68–8.65 (1H, m, Ar), 8.00–7.95 (2H, m, Ar), 7.49 (1H, ddd, *J* 1.6, 4.9, 6.7 Hz, Ar), 7.19 (1H, s, H-4), 4.39 (1H, d, *J*_1′,2′_ 9.7 Hz, H-1′), 3.99 (1H, dd, *J*_4′,5′_ 0.6 Hz, H-4′), 3.89 (1H, pseudo t, *J*_2′,3′_ 9.4 Hz, H-2′), 3.80 (1H, dd, *J*_6a′,6b′_ 11.1 Hz, H-6_a_′), 3.72 (1H, dd, H-6_b_′), 3.70 (1H, ddd, *J*_5′,6b′_ 4.9, *J*_5′,6a′_ 6.6 Hz, H-5′), 3.62 (1H, dd, *J*_3′,4′_ 3.3 Hz, H-3′). ^13^C NMR (100 MHz, CD_3_OD) δ (ppm) 170.0 (C-5), 165.2 (C-3), 151.6–121.5 (Ar), 102.6 (d, *J* 15.7 Hz, C-4), 81.2 (C-5′), 76.5 (d, *J* 10.1 Hz, C-1′), 76.1 (C-3′), 71.5 (C-2′), 70.8 (d, *J* 6.2 Hz, C-4′), 62.9 (C-6′). HR-ESI-MS positive mode (*m*/*z*): calcd. for C_14_H_16_N_2_O_6_ (308.10) [M + H]^+^ = 309.1081, found: [M + H]^+^ = 309.1081.

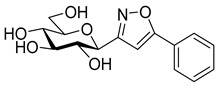



##### 3-(β-d-Glucopyranosyl)-5-phenylisoxazole (**11a**)

Prepared from isoxazole **7a** (0.06 g, 0.08 mmol) according to the General Procedure II. Purified by column chromatography (4:1 CHCl_3_–MeOH) to yield 19 mg (80%) of **11a** as a pale orange amorphous product. R_f_: 0.44 (4:1 CHCl_3_–MeOH). ^1^H NMR (500 MHz, CD_3_OD) δ (ppm) 7.83 (2H, d, *J* 7.7 Hz, Ar), 7.50 (2H, d, *J* 7.9 Hz, Ar), 7.54–7.42 (1H, m, Ar), 6.91 (1H, s, H-4), 4.42 (1H, d, *J*_1′,2′_ 8.9 Hz, H-1′), 3.90 (1H, dd, *J*_6a′,6b′_ 12.1 Hz, H-6_a_′), 3.72 (1H, dd, H-6_b_′), 3.54 (1H, pseudo t, *J*_2′,3′_ 9.0 Hz, H-2′), 3.51 (1H, pseudo t, *J*_3′,4′_ 8.7 Hz, H-3′), 3.45 (1H, pseudo t, *J*_4′,5′_ 8.7 Hz, H-4′), 3.45 (1H, ddd, *J*_5′,6a′_ < 1.0, *J*_5′,6b′_ 2.9 Hz, H-5′). ^13^C NMR (125 MHz, CD_3_OD) δ (ppm) 171.2 (C-5), 165.0 (C-3), 131.6–126.5 (Ar), 99.8 (C-4), 82.4 (C-5′), 79.5 (C-3′), 76.1 (C-1′), 74.7 (C-2′), 71.4 (C-4′), 62.9 (C-6′). C_15_H_17_NO_6_ (307.11). NMR spectra are identical with those reported [16].

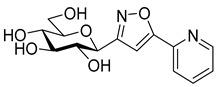



##### 3-(β-d-Glucopyranosyl)-5-(pyridin-2-yl)isoxazole (**11d**)

Prepared from isoxazole **7d** (0.03 g, 0.04 mmol) according to the General Procedure II. Purified by column chromatography (4:1 CHCl_3_–MeOH) to yield 9 mg (67%) of **11d** as a colourless amorphous product. R_f_: 0.32 (4:1 CHCl_3_–MeOH); [α]_D_+21 (*c* 0.15, MeOH). ^1^H NMR (500 MHz, CD_3_OD) δ (ppm) 8.66 (1H, dt, *J* 4.9, 1.2 Hz, Ar), 8.02–7.95 (2H, m, Ar), 7.49 (1H, ddd, *J* 6.9, 4.9, 2.3 Hz, Ar), 7.13 (1H, s, H-4), 4.46 (1H, d, *J*_1′,2′_ 9.3 Hz, H-1′), 3.90 (1H, dd, *J*_6a′,6b′_ 12.2 Hz, H-6_a_′), 3.72 (1H, strongly coupled, H-6_b_′), 3.54 (1H, pseudo t, *J*_2′,3′_ 8.9 Hz, H-2′), 3.51 (1H, pseudo t, *J*_3′,4′_ 8.9 Hz, H-3′), 3.45 (1H, pseudo t, *J*_4′,5′_ 9.5 Hz, H-4′), 3.45 (1H, ddd, *J*_5′,6a′_ 1.4, *J*_5′,6b′_ 5.1 Hz, H-5′). ^13^C NMR (125 MHz, CD_3_OD) δ (ppm) 170.0 (C-5), 165.2 (C-3), 151.3–122.2 (Ar), 102.5 (C-4), 82.5 (C-5′), 79.5 (C-3′), 76.0 (C-1′), 74.7 (C-2′), 71.5 (C-4′), 62.9 (C-6′). HR-ESI-MS positive mode (*m*/*z*): calcd. for C_14_H_16_N_2_O_6_ (308.10) [M + Na]^+^ = 331.0901, found: [M + Na]^+^ = 331.0900.

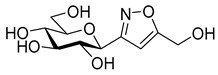



##### 3-(β-d-Glucopyranosylisoxazol-5-yl)methanol (**11f**)

Prepared from isoxazole **7f** (0.05 g, 0.08 mmol) according to the General Procedure II. Purified by column chromatography (4:1 CHCl_3_–MeOH) to yield 21 mg (99%) of **11f** as a colourless amorphous product. R_f_: 0.07 (4:1 CHCl_3_–MeOH); [α]_D_+17 (*c* 0.26, MeOH). ^1^H NMR (500 MHz, CD_3_OD) δ (ppm) 6.45 (1H, s, H-4), 4.66 (2H, s, C*H*_2_OH), 4.36 (1H, d, *J*_1′,2′_ 9.3 Hz, H-1′), 3.87 (1H, dd, *J*_6a′,6b′_ 12.2 Hz, H-6_a_′), 3.69 (1H, strongly coupled, H-6_b_′), 3.50–3.43 (2H, m, H-2′, H-3′), 3.41 (1H, pseudo t, *J*_4′,5′_ 9.4 Hz, H-4′), 3.40 (1H, ddd, *J*_5′,6a′_ 1.2, *J*_5′,6b′_ 5.2 Hz, H-5′). ^13^C NMR (125 MHz, CD_3_OD) δ (ppm) 173.8 (C-5), 164.2 (C-3), 101.7 (C-4), 82.4 (C-5′), 79.5 (C-3′), 76.0 (C-1′), 74.7 (C-2′), 71.4 (C-4′), 62.8 (C-6′), 56.4 (CH_2_OH). HR-ESI-MS positive mode (*m*/*z*): calcd. for C_10_H_15_NO_7_ (261.08) [M + Na]^+^ = 284.0741, found: [M + Na]^+^ = 284.0738.

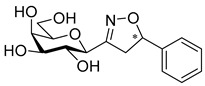



##### 3-(β-d-Galactopyranosyl)-5-phenylisoxazolines (**12a**)

Prepared from isoxazolines **8a** (0.09 g, 0.18 mmol) according to the General Procedure II. Purified by column chromatography (4:1 CHCl_3_–MeOH) to yield 18 mg (76%) of **12a** (diastereomeric ratio: 1.1:1) as a white amorphous product. R_f_: 0.52 (7:3 CHCl_3_–MeOH). **12a-I** ^1^H NMR (400 MHz, CD_3_OD) δ (ppm) 7.41–7.27 (5H, m, Ar), 5.61 (1H, dd, *J*_4a,5_ 11.0, *J*_4b,5_ 8.4 Hz, H-5), 4.08 (1H, dd, *J*_1′,2′_ 9.7 Hz, H-1′), 3.92 (1H, dd, *J*_3′,4′_ 3.3, *J*_4′,5′_ 0.6 Hz, H-4′), 3.81–3.51 (6H, m, H-2′, H-3′, H-5′, H-6_a_′, H-6_b_′, H-4_a_), 3.15 (1H, dd, *J*_4a,4b_ 17.6 Hz, H-4_b_). ^13^C NMR (90 MHz, CD_3_OD) δ (ppm) 158.8 (C-3), 142.6–126.7 (Ar), 83.4 (C-5), 80.9 (C-5′), 77.1 (C-1′), 75.9 (C-3′), 70.8 (C-4′), 69.8 (C-2′), 62.8 (C-6′), 42.5 (C-4). **12a-II** ^1^H NMR (400 MHz, CD_3_OD) δ (ppm) 7.41–7.27 (5H, m, Ar), 5.57 (1H, dd, *J*_4a,5_ 10.9, *J*_4b,5_ 9.3 Hz, H-5), 4.09 (1H, dd, *J*_1′,2′_ 9.7 Hz, H-1′), 3.92 (1H, dd, *J*_3′,4′_ 3.3, *J*_4′,5′_ 0.6 Hz, H-4′), 3.81–3.51 (6H, m, H-2′, H-3′, H-5′, H-6_a_′, H-6_b_′, H-4_a_), 3.09 (1H, dd, *J*_4a,4b_ 17.5 Hz, H-4_b_). ^13^C NMR (90 MHz, CD_3_OD) δ (ppm) 159.1 (C-3), 142.6–126.7 (Ar), 83.7 (C-5), 80.9 (C-5′), 77.1 (C-1′), 76.1 (C-3′), 70.8 (C-4′), 69.8 (C-2′), 62.8 (C-6′), 42.6 (C-4). HR-ESI-MS positive mode (*m*/*z*): calcd. for C_15_H_19_NO_6_ (309.12) [M + H]^+^ = 310.1285, found: [M + H]^+^ = 310.1286.

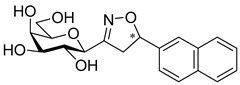



##### 3-(β-d-Galactopyranosyl)-5-(naphth-2-yl)isoxazolines (**12b**)

Prepared from isoxazolines **8b** (0.06 g, 0.10 mmol) according to the General Procedure II. Purified by column chromatography (4:1 CHCl_3_–MeOH) to yield 26 mg (70%) of **12b** (diastereomeric ratio: 1.1:1) as a brownish amorphous product. R_f_: 0.59 (7:3 CHCl_3_–MeOH). **12b-I** ^1^H NMR (400 MHz, CD_3_OD) δ (ppm) 7.91–7.80 (4H, m, Ar), 7.56–7.42 (3H, m, Ar), 5.78 (1H, dd, *J*_4a,5_ 10.9, *J*_4b,5_ 8.3 Hz, H-5), 4.12 (1H, dd, *J*_1′,2′_ 9.7 Hz, H-1′), 3.92 (1H, dd, *J*_3′,4′_ 3.2, *J*_4′,5′_ 0.9 Hz, H-4′), 3.81 (1H, dd, *J*_2′,3′_ 9.5 Hz, H-2′), 3.80–3.59 (4H, m, H-5′, H-6_a_′, H-6_b_′, H-4_a_), 3.56 (1H, dd, H-3′), 3.26 (1H, dd, *J*_4a,4b_ 17.6 Hz, H-4_b_). ^13^C NMR (90 MHz, CD_3_OD) δ (ppm) 158.9 (C-3), 139.9–124.7 (Ar), 83.5 (C-5), 80.9 (C-5′), 77.2 (C-1′), 76.1 (C-3′), 70.8 (C-4′), 69.8 (C-2′), 62.8 (C-6′), 42.5 (C-4). **12b-II** ^1^H NMR (400 MHz, CD_3_OD) δ (ppm) 7.91–7.80 (4H, m, Ar), 7.56–7.42 (3H, m, Ar), 5.73 (1H, dd, *J*_4a,5_ 10.8, *J*_4b,5_ 9.1 Hz, H-5), 4.13 (1H, dd, *J*_1′,2′_ 9.7 Hz, H-1′), 3.92 (1H, dd, *J*_3′,4′_ 3.2, *J*_4′,5′_ 0.9 Hz, H-4′), 3.80–3.59 (5H, m, H-2′, H-5′, H-6_a_′, H-6_b_′, H-4_a_), 3.55 (1H, dd, H-3′), 3.19 (1H, dd, *J*_4a,4b_ 17.4 Hz, H-4_b_). ^13^C NMR (90 MHz, CD_3_OD) δ (ppm) 159.1 (C-3), 139.9–124.7 (Ar), 83.8 (C-5), 80.9 (C-5′), 77.1 (C-1′), 75.9 (C-3′), 70.8 (C-4′), 69.8 (C-2′), 62.8 (C-6′), 42.6 (C-4). HR-ESI-MS positive mode (*m*/*z*): calcd. for C_19_H_21_NO_6_ (359.14) [M + H]^+^ = 360.1442, found: [M + H]^+^ = 360.1444.

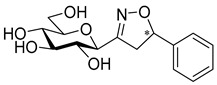



##### 3-(β-d-Glucopyranosyl)-5-phenylisoxazolines (**13a**)

Prepared from isoxazolines **3a** (0.07 g, 0.10 mmol) according to the General Procedure II. Purified by column chromatography (4:1 CHCl_3_–MeOH) to yield 24 mg (80%) of **13a** (diastereomeric ratio: 1.3:1) as a pale orange amorphous product. R_f_: 0.44 (4:1 CHCl_3_–MeOH). **13a-I** ^1^H NMR (500 MHz, CD_3_OD) δ (ppm) 7.42–7.26 (5H, m, Ar), 5.61 (1H, dd, *J*_4a,5_ 11.1, *J*_4b,5_ 8.6 Hz, H-5), 4.14 (1H, dd, *J*_1′,2′_ 9.2 Hz, H-1′), 3.85 (1H, dd, *J*_5′,6a′_ 1.3, *J*_6a′,6b′_ 11.7 Hz, H-6_a_′), 3.65 (1H, dd, *J*_5′,6b′_ 4.9 Hz, H-6_b_′), 3.54 (1H, dd, *J*_4a,4b_ 17.5, *J*_4a,5_ 11.4 Hz, H-4_a_), 3.47–3.27 (3H, m, H-3′, H-4′, H-5′), 3.41 (1H, pseudo t, *J*_2′,3′_ 8.7 Hz, H-2′), 3.15 (1H, dd, *J*_4b,5_ 8.5 Hz, H-4_b_). ^13^C NMR (125 MHz, CD_3_OD) δ (ppm) 158.8 (C-3), 142.5–126.9 (Ar), 83.4 (C-5), 82.3 (C-5′), 79.3 (C-3′), 76.7 (C-1′), 73.0 (C-2′), 71.4 (C-4′), 62.7 (C-6′), 42.6 (C-4). **13a-II** ^1^H NMR (500 MHz, CD_3_OD) δ (ppm) 7.42–7.26 (5H, m, Ar), 5.57 (1H, dd, *J*_4a,5_ 10.8, *J*_4b,5_ 10.0 Hz, H-5), 4.13 (1H, dd, *J*_1′,2′_ 9.3 Hz, H-1′), 3.85 (1H, dd, *J*_5′,6a′_ 1.3, *J*_6a′,6b′_ 11.7 Hz, H-6_a_′), 3.67 (1H, dd, *J*_5′,6b′_ 5.4 Hz, H-6_b_′), 3.60 (1H, dd, *J*_4a,4b_ 17.4, *J*_4a,5_ 11.1 Hz, H-4_a_), 3.47–3.27 (3H, m, H-3′, H-4′, H-5′), 3.41 (1H, pseudo t, *J*_2′,3′_ 8.7 Hz, H-2′), 3.06 (1H, dd, *J*_4b,5_ 9.2 Hz, H-4_b_). ^13^C NMR (125 MHz, CD_3_OD) δ (ppm) 159.0 (C-3), 142.5–126.9 (Ar), 83.7 (C-5), 82.3 (C-5′), 79.5 (C-3′), 76.6 (C-1′), 73.1 (C-2′), 71.4 (C-4′), 62.8 (C-6′), 42.7 (C-4). HR-ESI-MS positive mode (*m*/*z*): calcd. for C_15_H_19_NO_6_ (309.12) [M + Na]^+^ = 332.1105, found: [M + Na]^+^ = 332.1104.

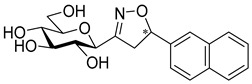



##### 3-(β-d-Glucopyranosyl)-5-(naphth-2-yl)isoxazolines (**13b**)

Prepared from isoxazolines **3b** (0.08 g, 0.10 mmol) according to the General Procedure II. Purified by column chromatography (4:1 CHCl_3_–MeOH) to yield 16 mg (46%) of **13b** (diastereomeric ratio: 1:1) as a white amorphous product. R_f_: 0.40 (4:1 CHCl_3_–MeOH). **13b-I** ^1^H NMR (500 MHz, CD_3_OD) δ (ppm) 8.11–7.77 (4H, m, Ar), 7.65–7.39 (3H, m, Ar), 5.79 (1H, dd, *J*_4a,5_ 11.2, *J*_4b,5_ 8.7 Hz, H-5), 4.16 (1H, dd, *J*_1′,2′_ 9.1 Hz, H-1′), 3.84 (1H, dd, *J*_5′,6a′_ 1.6, *J*_6a′,6b′_ 12.0 Hz, H-6_a_′), 3.71–3.54 (2H, m, H-4_a_, H-6_b_′), 3.50–3.41 (2H, m, H-2′, H-3′), 3.41–3.29 (2H, m, H-4′, H-5′), 3.25 (1H, dd, *J*_4a,4b_ 17.5, *J*_4b,5_ 8.4 Hz, H-4_b_). ^13^C NMR (125 MHz, CD_3_OD) δ 158.9 (C-3), 139.8–124.7 (Ar), 83.6 (C-5), 82.3 (C-5′), 79.4 (C-3′), 76.7 (C-1′), 73.1 (C-2′), 71.4 (C-4′), 62.7 (C-6′), 42.6 (C-4). **13b-II** ^1^H NMR (500 MHz, CD_3_OD) δ (ppm) 8.11–7.77 (4H, m, Ar), 7.65–7.39 (3H, m, Ar), 5.75 (1H, dd, *J*_4a,5_ 11.3, *J*_4b,5_ 9.7 Hz, H-5), 4.17 (1H, dd, *J*_1′,2′_ 9.3 Hz, H-1′), 3.88 (1H, dd, *J*_5′,6a′_ 2.0, *J*_6a′,6b′_ 12.1 Hz, H-6_a_′), 3.71–3.54 (2H, m, H-4_a_, H-6_b_′), 3.50–3.41 (2H, m, H-2′, H-3′), 3.41–3.29 (2H, m, H-4′, H-5′), 3.17 (1H, dd, *J*_4a,4b_ 17.4, *J*_4b,5_ 9.2 Hz, H-4_b_). ^13^C NMR (125 MHz, CD_3_OD) δ (ppm) 159.1 (C-3), 139.8–124.7 (Ar), 83.9 (C-5), 82.3 (C-5′), 79.5 (C-3′), 76.7 (C-1′), 73.1 (C-2′), 71.4 (C-4′), 62.8 (C-6′), 42.6 (C-4). HR-ESI-MS positive mode (*m*/*z*): calcd. for C_19_H_21_NO_6_ (359.14) [M + Na]^+^ = 382.1261, found: [M + Na]^+^ = 382.1258.

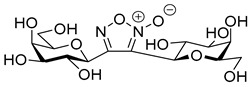



##### 3,4-Di(β-d-Galactopyranosyl)-1,2,5-oxadiazole-2-oxide (**14**)

Prepared from isoxazole **4b** (0.10 g, 0.13 mmol) according to the General Procedure II. Purified by column chromatography (4:1 CHCl_3_–MeOH) to yield 19 mg (34%) of **14** as a white amorphous product. R_f_: 0.16 (1:1 CHCl_3_–MeOH); [α]_D_+82 (*c* 0.08, DMSO). ^1^H NMR (500 MHz, CD_3_OD) δ (ppm) 4.44 (1H, d, *J*_1′,2′_ 9.9 Hz, H-1′), 4.42 (1H, d, *J*_1″,2″_ 9.8 Hz, H-1″), 4.31 (1H, pseudo t, *J*_2′,3′_ 9.6 Hz, H-2′), 4.29 (1H, pseudo t, *J*_2″,3″_ 9.6 Hz, H-2″), 3.92 (1H, dd, *J*_4″,5″_ 0.5 Hz, H-4″), 3.89 (1H, dd, *J*_4′,5′_ 0.5 Hz, H-4′), 3.91–3.83 (2H, m, H-6_a_′, H-6_a_″), 3.75–3.66 (4H, m, H-5′, H-5″, H-6_b_′, H-6_b_″), 3.56 (1H, dd, *J*_3″,4″_ 3.3 Hz, H-3″), 3.55 (1H, dd, *J*_3′,4′_ 3.4 Hz, H-3′). ^13^C NMR (125 MHz, CD_3_OD) δ (ppm) 157.2 (C-4), 115.6 (C-3), 82.2 (C-5′), 81.8 (C-5″), 77.9 (C-1″), 76.2 (C-3″), 76.1 (C-3′), 75.3 (C-1′), 71.0 (C-4′, C-4″), 70.2 (C-2′), 69.9 (C-2″), 62.7 (C-6″), 62.6 (C-6′). HR-ESI-MS positive mode (*m*/*z*): calcd. for C_14_H_22_N_2_O_11_ (410.33) [M + H]^+^ = 411.1246, found: [M + H]^+^ = 411.1246.

### 3.2. Chemicals for Cell Proliferation Experiments

All chemicals used in the cell biology and biochemistry assays were obtained from Sigma-Aldrich (St. Louis, MO, USA) unless otherwise stated. The compounds were dissolved in dimethyl-sulfoxide for biology experiments, and 0.1% dimethylsulfoxide was used as a vehicle control.

### 3.3. Cell Culture

The cells were cultured under standard cell culture conditions: 37 °C, 5% CO_2_, humidified atmosphere.

*A2780* cells were cultured in RMPI 1640 medium, supplemented with 10% fetal calf serum, 2 mM glutamine, and 1% penicillin-streptomycin.

### 3.4. Methylthiazolyldiphenyl-Tetrazolium Bromide (MTT) Reduction Assay

An MTT reduction assay measures the activity of mitochondrial complex I and can be used to detect toxicity [33]. The assay was performed in a manner similar to that described in [32]. Briefly, the cells were plated in 96-well plates the day before the assay. The cells were treated with the compounds for 4 h; then, MTT was added to a 0.5 mg/mL final concentration, and the cells were incubated at 37 °C in a cell incubator for 40–60 min as a function of the cell line being assessed. Culture medium was removed, the reduced MTT dye was dissolved in dimethyl-sulfoxide, and plates were measured in a plate photometer (Thermo Scientific Multiscan GO spectrophotometer, Waltham, MA, USA) at 540 nm. On each plate, the wells were designed to contain vehicle-treated cells. In calculations, the readings for these wells were considered to be 1, and all readings were expressed relative to these values.

### 3.5. Sulforhodamine B (SRB) Binding Assay

An SRB assay measures the protein content of cells in correlation with the cell number in an assay well and can therefore be used to assess cell proliferation or long-term cytostasis [34]. The cells were seeded in 96-well plates the day before the treatment for the assay. The cells were treated with the compounds for 48 h. Then the medium was removed and the cells were fixed with 10% trichloroacetic acid. The fixed cells were washed in distilled water 3 times, followed by staining with SRB (0.4 m/V% dissolved in 1% acetic acid) for 10 min. The stained cells were washed in 1% acetic acid 5 times before the acetic acid was removed and the cells were left to dry. Protein-bound SRB was released by adding 100 µL of 10 mM Tris base. The plates were measured in a plate photometer (Thermo Scientific Multiscan GO spectrophotometer, Waltham, MA, USA) at 540 nm. On each plate, the wells were designed to contain vehicle-treated cells. In calculations, the readings for these wells were considered to be 1, and all readings were expressed relative to these values.

## 4. Conclusions

Nitrile oxides were generated in situ from anhydro-aldose oximes in a two-step halogenation–elimination sequence using NCS/Et_3_N/dry CH_2_Cl_2_ conditions. The resulting nitrile oxides were then used in 1,3-dipolar cycloaddition reactions with alkynes and alkenes. These cycloadditions resulted exclusively in 5-substituted-3-(*C*-glycopyranosyl)isoxazoles **6a**–**g**,**i** and -isoxazolines **3a**–**c** in low to good yields. Debenzoylated 3-(β-d-glucopyranosyl)isoxazoles **11d**,**f** and -isoxazolines **13a**,**b** gave no significant inhibition against rabbit muscle glycogen phosphorylase b, and neither the protected nor unprotected galactopyranosyl derivatives **6d**, **8b**, **10a**, and **12a** exhibited significant activity against A2780 ovarian cancer cells.

## Figures and Tables

**Table 1:** Optimization reactions of 1,3-dipolar cycloaddition reactions of *C*-(2,3,4,6-tetra-*O*-benzoyl-β-d-glucopyranosyl)formaldehyde oxime **1a** with styrene **2a**.

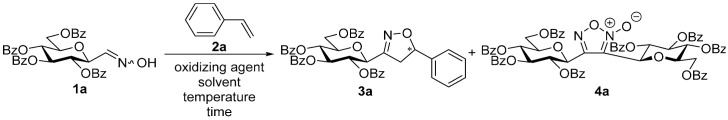									
Entry	Reaction Conditions							Yield (%)	
	Styrene (2a) (Equiv.)	Reagent (Equiv.)	Et_3_N (Equiv.)	Solvent	Temp. (°C)	Time (h)	N_2_ Atm.	3a	4a
1	3	NaOCl (0.14)	-	THF	rt	24	+	60 ^a^	+ ^b^
2	5	KI (1.1) oxone (1.5)	-	MeOH:H_2_O = 20:1	25–40	72	-	Complex reaction mixture	
3	5	NBS (1.1)	1.1	DMF	rt	4	-	58 ^a^	+ ^b^
4	5	NBS (1.1)	1.1	CH_2_Cl_2_	rt	4	-	64 ^a^	+ ^b^
5	5	NCS (1.1)	1.1	CH_2_Cl_2_	rt	4	-	65 ^a^	+ ^b^

^a^ Diastereomeric ratio: 1.1:1. ^b^ Compound **4a** was detected after the work-up in the ^1^H NMR spectra.

**Table 2:** Optimization reactions of 1,3-dipolar cycloaddition reactions of *C*-(2,3,4,6-tetra-*O*-acetyl-β-d-galactopyranosyl)formaldehyde oxime **1b** with phenylacetylene **5a**.

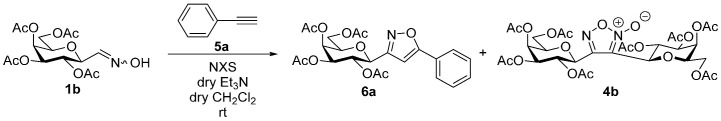								
Entry	Reaction Conditions					Conversion (%)	Isolated Yield Corr. with Conv. (%)	
	Phenylacetylene 5a (Equiv.)	NXS (Equiv.)	Dry Et_3_N (Equiv.)	Time (h)	Dosage Time (h)		6a	4b
1	5	NCS (1.1)	1.1	24.5	0.5	100	29	61
2	1	NCS (1.5)	1.5	72	2	38	52	45
3	1	NCS (1.5)	1.5	24	4	68	37	57
4	1	NCS (1.1)	2.2	24	4	60	40	- ^a^
5	1	NCS (1.1)	2.2	16.5	16 ^b^	70	50	28
6	1	NCS (1.5)	2.2	16.5	16 ^b^	76	47	- ^a^
7	1.5	NCS (1.5)	2.2	16.5	16 ^b^	77	51	- ^a^
8	1.5	NCS (2)	2.2	16.5	16 ^b^	85	61	- ^a^
9	1.5	NBS (2)	2.2	16.5	16 ^b^	100	36	- ^a^
10	2	NCS (2)	2.2	16.5	16 ^b^	83	56	- ^a^
**11 ^c^**	**2**	**NCS (3)**	**3.3**	**16.5**	**16 ^b^**	**100**	**68**	**- ^a^**

^a^ Compound **4b** was not detected after work-up. ^b^ Syringe pump was applied. ^c^ Optimized reaction conditions.

**Table 3:** 1,3-Dipolar cycloaddition reactions of *C*-(2,3,4,6-tetra-*O*-acetyl-β-d-galactopyranosyl)formaldehyde oxime **1b** with alkyne dipolarophiles **5a**–**i**.

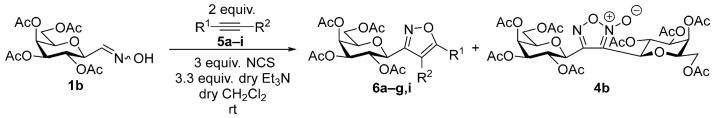					
Entry		R^1^	R^2^	Isolated Yield (%)	
				6	4b
1	**a**	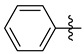	H	68	- ^a^
2	**b**	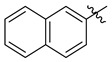	H	56	- ^a^
3	**c**	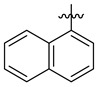	H	57	- ^a^
4	**d**	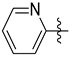	H	63	+ ^b^
5	**e**	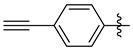	H	41	- ^a^
6	**f**	CH_2_OH	H	47	- ^a^
7	**g**	CH_2_OAc	H	46	+ ^b^
8	**h**	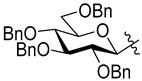	H	- ^a^	99
9	**i**	COOEt	COOEt	44	- ^a^

^a^ Compounds were not detected after work-up. ^b^ Compound **4b** was detected after work-up in ^1^H NMR spectra.

**Table 4:** 1,3-Dipolar cycloaddition reactions of *C*-(2,3,4,6-tetra-*O*-benzoyl-β-d-glucopyranosyl)formaldehyde oxime **1a** with alkyne dipolarophiles **5a**,**d**–**i**.

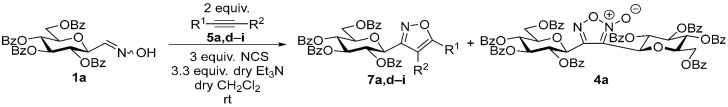					
Entry		R^1^	R^2^	Isolated Yield (%)	
				7	4a
1	**a**	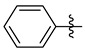	H	57	- ^a^
2	**d**	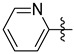	H	39	+ ^b^
3	**e**	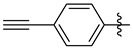	H	24	- ^a^
4	**f**	CH_2_OH	H	67	- ^a^
5	**g**	CH_2_OAc	H	47	4
6	**h**	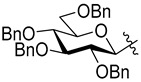	H	16	31
7	**i**	COOEt	COOEt	50	+ ^b^

^a^ Compound **4a** was not detected after work-up. ^b^ Compound **4a** was detected after work-up in ^1^H NMR spectra.

**Table 5:** 1,3-Dipolar cycloaddition reactions of *C*-(2,3,4,6-tetra-*O*-acetyl-β-d-galactopyranosyl)formaldehyde oxime **1b** with alkene dipolarophiles **2a**–**g**.

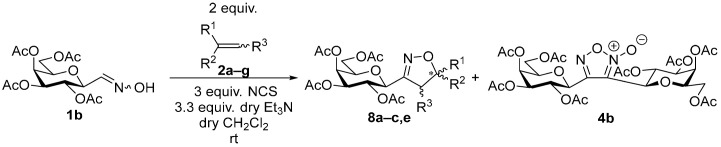							
Entry		R^1^	R^2^	R^3^	Diastereomeric Ratio 8	Isolated Yield (%)	
						8	4b
1	**a**	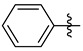	H	H	1.1:1	83	- ^a^
2	**b**	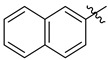	H	H	1.1:1	71	+ ^b^
3	**c**	OAc	H	H	1.6:1	53	+ ^b^
4	**d**	H (cis)	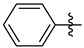	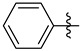	-	- ^a^	99
5	**e**	H (trans)	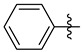	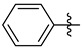	-	23	76
6	**f**	OMe	CH_3_	H	-	- ^a^	23
7	**g**	H	(CH_2_)_4_		-	- ^a^	99

^a^ Compounds were not detected after work-up. ^b^ Compound **4b** was detected after work-up in ^1^H NMR spectra.

**Table 6:** 1,3-Dipolar cycloaddition reactions of *C*-(2,3,4,6-tetra-*O*-benzoyl-β-d-glucopyranosyl)formaldehyde oxime **1a** with alkene dipolarophiles **2a**–**c**,**f**.

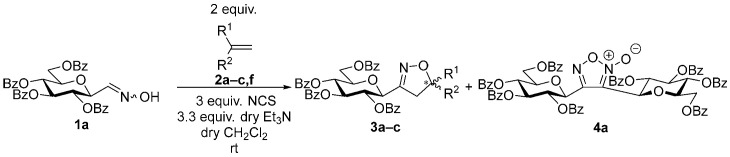						
Entry		R^1^	R^2^	Diastereomeric Ratio 3	Isolated Yield (%)	
					3	4a
1	**a**	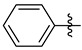	H	1.3:1	77	- ^a^
2	**b**	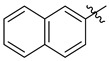	H	1:1	93	- ^a^
3	**c**	OAc	H	1.1:1	59	+ ^b^
4	**f**	OMe	CH_3_	Complex reaction mixture		

^a^ Compounds **4a** was not detected after work-up. ^b^ Compound **4a** was detected after work-up in ^1^H NMR spectrum.

**Table 7:** Deacylation of 5-substituted 3-(2,3,4,6-tetra-*O*-acyl-β-d-glycopyranosyl)isoxazoles **6a**–**d** and **7a**,**d**,**f**.

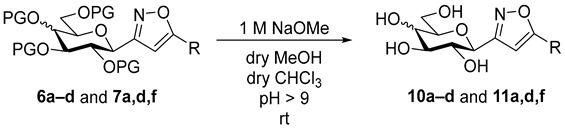						
Entry	Isoxazole	Sugar Configuration	Protecting Group	R	Isoxazole	Isolated Yield (%)
1	**6a**	Gal	Ac	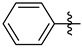	**10a**	49
2	**6b**	Gal	Ac	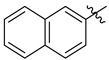	**10b**	43
3	**6c**	Gal	Ac	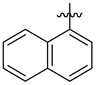	**10c**	75
4	**6d**	Gal	Ac	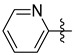	**10d**	63
5	**7a**	Glc	Bz	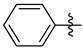	**11a**	80
6	**7d**	Glc	Bz	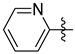	**11d**	67
7	**7f**	Glc	Bz	CH_2_OH	**11f**	99

**Table 8:** Deacylation 5-substituted 3-(2,3,4,6-tetra-*O*-acyl-β-d-glycopyranosyl)isoxazolines **8a**,**b** and **3a**,**b**.

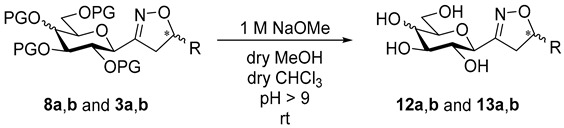							
Entry	Isoxazoline	Sugar Configuration	Protecting Group	R	Diastereomeric Ratio 12 and 13	Isoxazoline	Isolated Yield (%)
1	**8a**	Gal	Ac	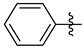	1.1:1	**12a**	76
2	**8b**	Gal	Ac	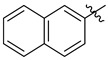	1.1:1	**12b**	70
3	**3a**	Glc	Bz	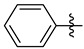	1.3:1	**13a**	80
4	**3b**	Glc	Bz	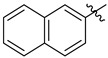	1:1	**13b**	46

## Data Availability

Primary data for cell proliferation experiments is available at https://figshare.com/s/2261964aba80a88bd606, accessed on 9 July, 2025 (DOI: 10.6084/m9.figshare.29524970).

## Supplementary Materials

The following supporting information can be downloaded at: https://www.mdpi.com/article/10.3390/ijms26178167/s1.
