# Supplementary material for: Regioselective Synthesis of 5-Substituted 3-(β-d-Glycopyranosyl)isoxazoles and -isoxazolines by 1,3-Dipolar Cycloaddition as Potential Anticancer Agents and Glycogen Phosphorylase Inhibitors
Source: Int J Mol Sci. 2025 Aug 22;26(17):8167. doi: 10.3390/ijms26178167 (PMC12427784; doi:10.3390/ijms26178167)
Supplement: Supplementary file 1 [file ijms-26-08167-s001.zip › ijms-3815418-supplementary.pdf]

Regioselective Synthesis of 5-Substituted 3-(β-D-Glycopyranosyl)isoxazoles and -isoxazolines by 1,3-Dipolar Cycloaddition as Potential Anticancer Agents and Glycogen Phosphorylase Inhibitors

Tímea Kaszás [1], Bence Szakács [1,2], Márta Bertalan [1], Tekla Blága [1], Faria Hameed [1], Ákos Lengyel [1], Samreen Saifi [1,2], Éva Juhász-Tóth [1], Luca A. Varga [3], Tibor Docsa [3], Adrienn Sipos [3], Péter Bai [3,4,5,6], Anita Ábrahám [1], Attila Kiss-Szikszai [1], Sándor Kun [1], György Attila Kiss [1], János József [1], László Juhász [1] and Marietta Tóth [1,*]

[1] Department of Organic Chemistry, University of Debrecen,
PO Box 400, H-4002 Debrecen, Hungary;
kaszas.timea@science.unideb.hu (T.K.); szakacs.bence@science.unideb.hu (B.Sz.);
bertalanmarta02@gmail.com (M.B.); tekla.tblaga@gmail.com (T.B.);
fariahameed555@gmail.com (F.H.); polszka76@mailbox.unideb.hu (Á.L.);
saifi.samreen@science.unideb.hu (S.S.); toth.eva@science.unideb.hu (É.J.-T.);
dulryc@unideb.hu (A.Á.); kiss.attila@science.unideb.hu (A.K.-Sz.);
kun.sandor@science.unideb.hu (S.K.); kiss.gyorgy@science.unideb.hu (Gy.A.K.);
jozsef.janos@science.unideb.hu (J.J.); juhasz.laszlo@science.unideb.hu (L.J.)

[2] Doctoral School of Chemistry, University of Debrecen,
PO Box 400, H-4002 Debrecen, Hungary

[3] Department of Medical Chemistry, Faculty of Medicine, University of Debrecen,
PO Box 400, H-4002 Debrecen, Hungary;
varga.luca@med.unideb.hu (L.A.V.); tdocsa@med.unideb.hu (T.D.);
siposadri@med.unideb.hu (A.S.); baip@med.unideb.hu (P.B.)

[4] HUN-REN Cell Biology and Signaling Research Group, H-4032, Debrecen, Hungary

[5] MTA-DE Lendület Laboratory of Cellular Metabolism, H-4032, Debrecen, Hungary

[6] Research Center for Molecular Medicine, Faculty of Medicine, University of Debrecen,
H-4032, Debrecen, Hungary

* Correspondence: toth.marietta@science.unideb.hu

CONTENTS

Copies of the NMR spectra S2–S95

Copies of the MS spectra S96–S100

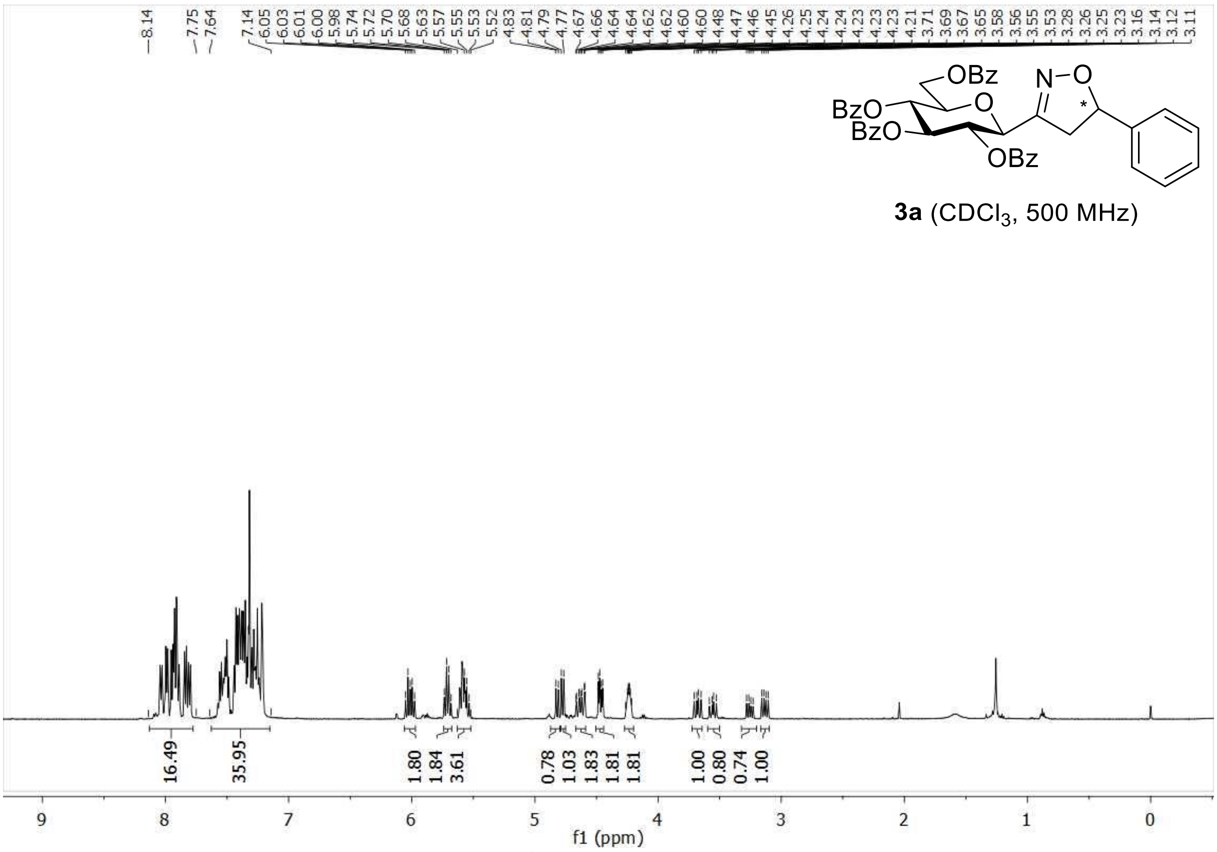

Figure S1. ^{1}H NMR spectrum of **3a**

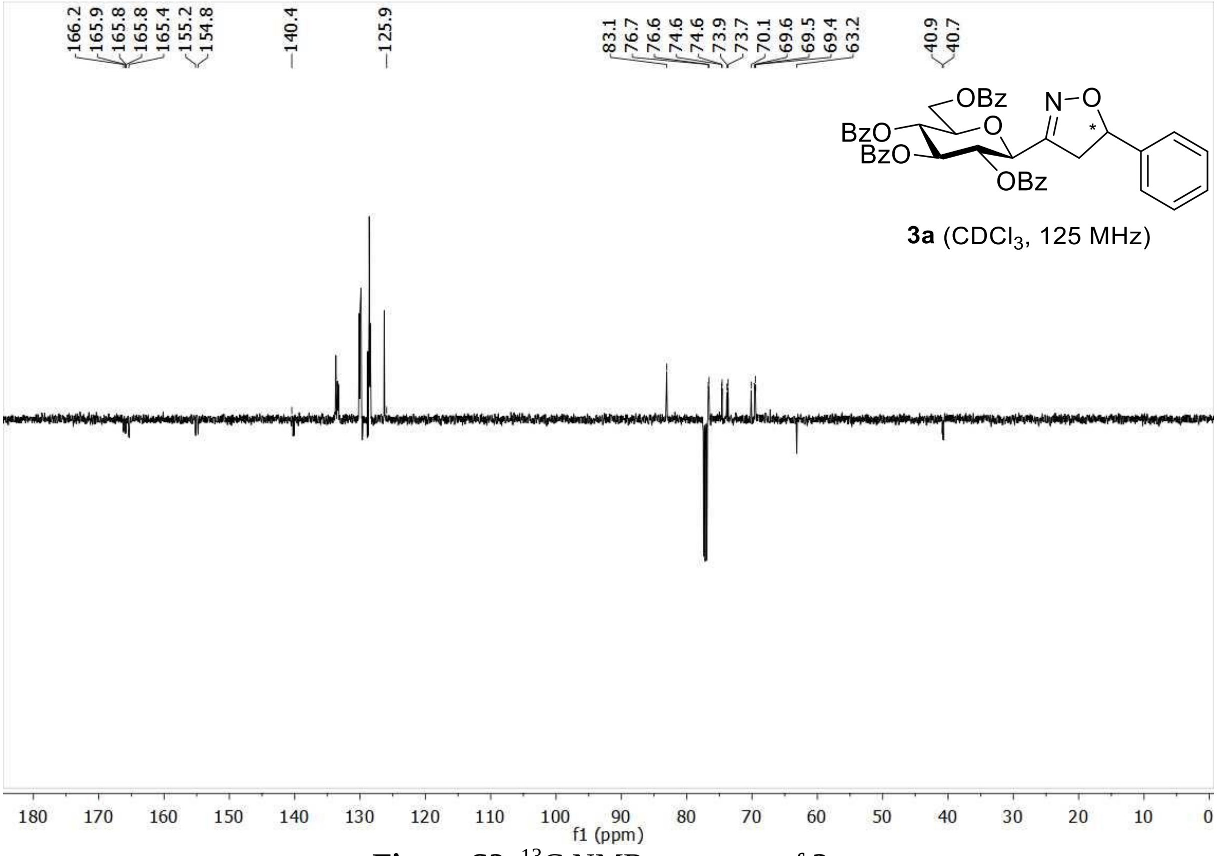

Figure S2. ^{13}C NMR spectrum of **3a**

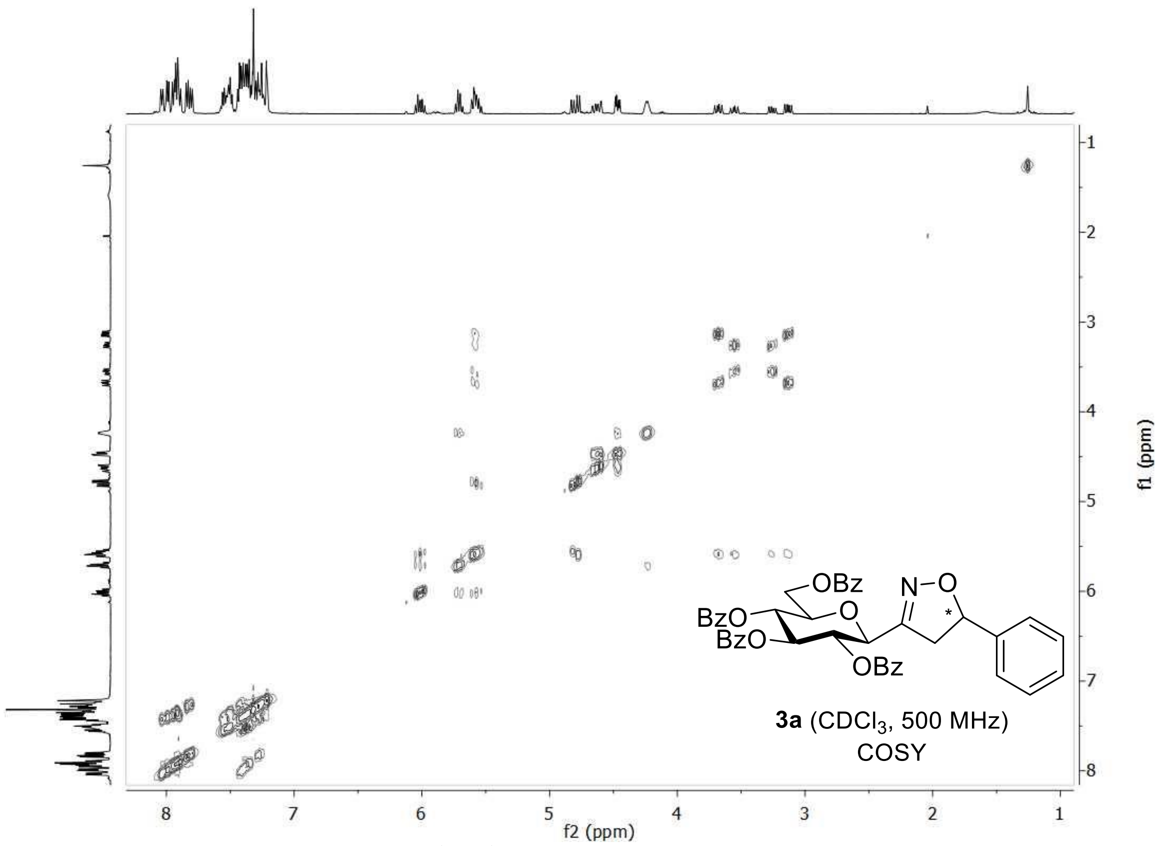

Figure S3. ^{1}H–^{1}H COSY spectrum of **3a**

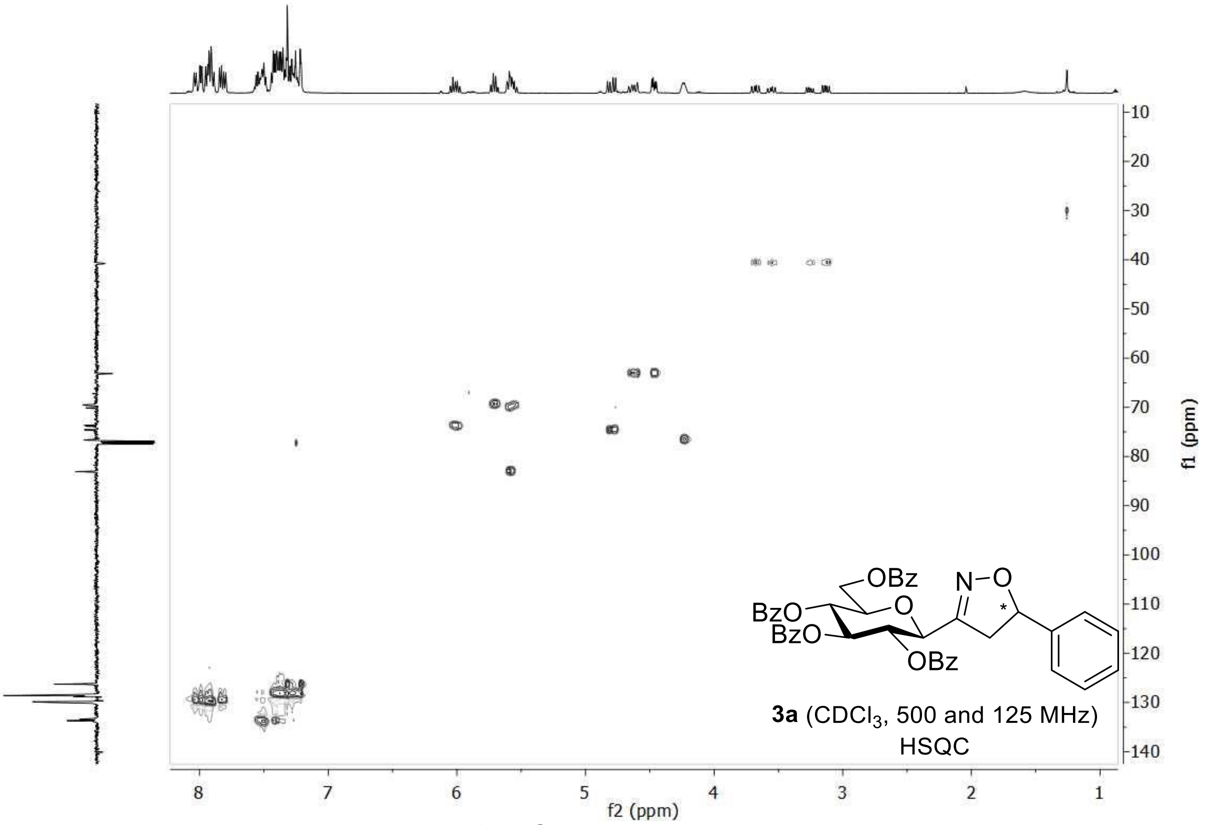

Figure S4. ^{1}H–^{13}C HSQC spectrum of **3a**

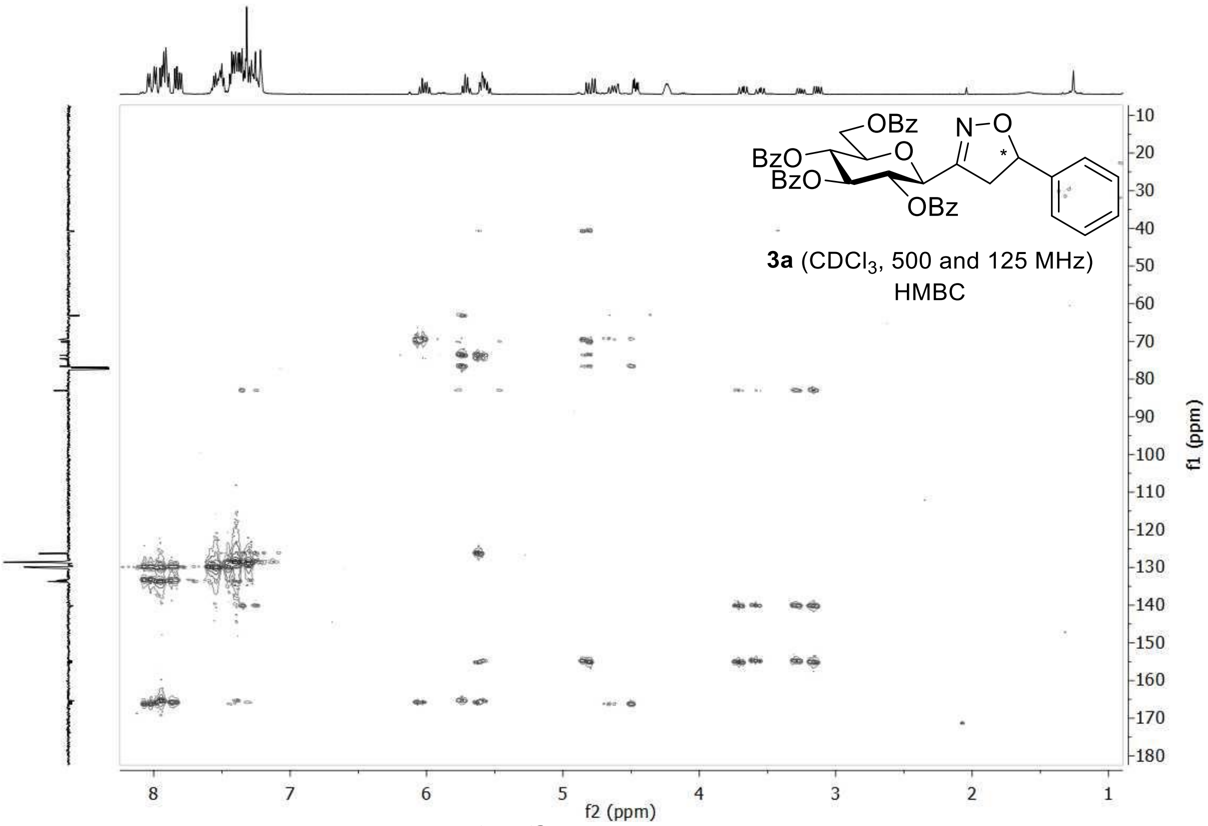

Figure S5. ^{1}H–^{13}C HMBC spectrum of **3a**

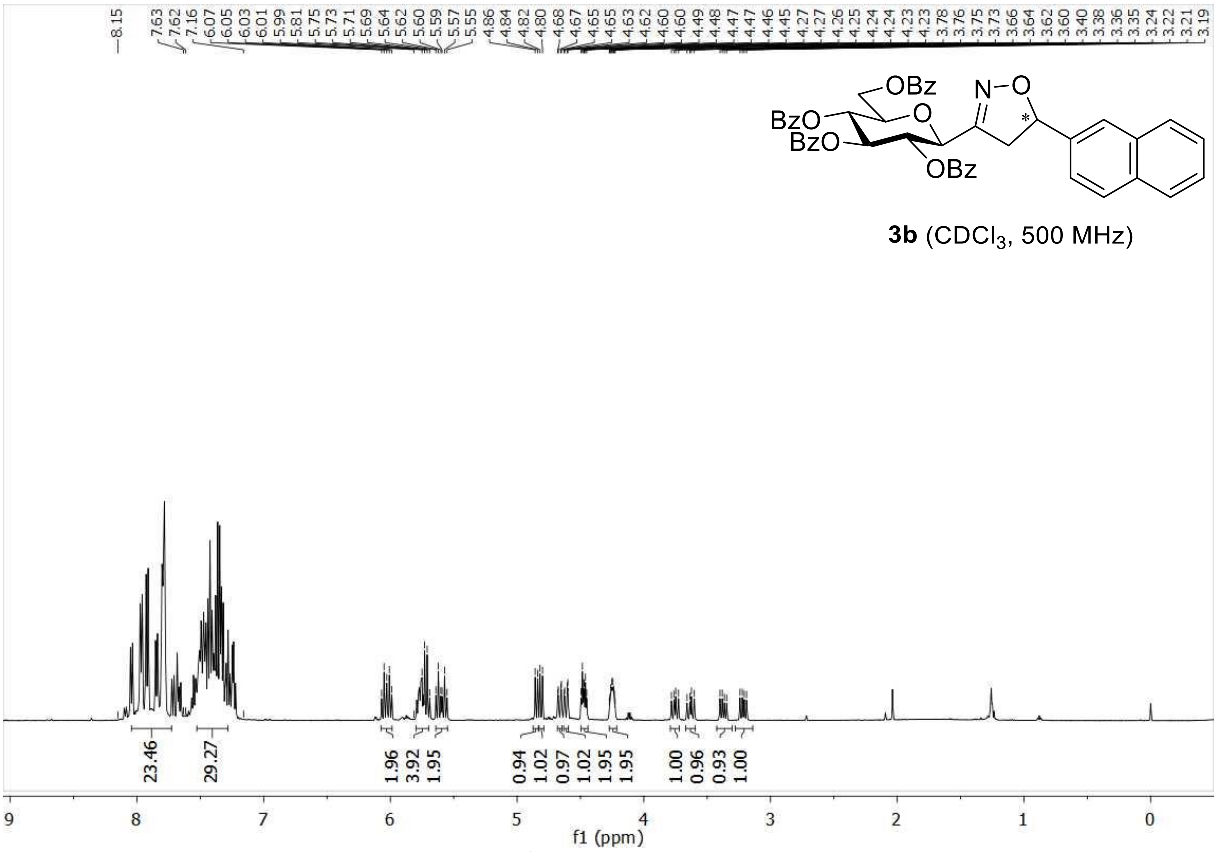

Figure S6. ^{1}H NMR spectrum of **3b**

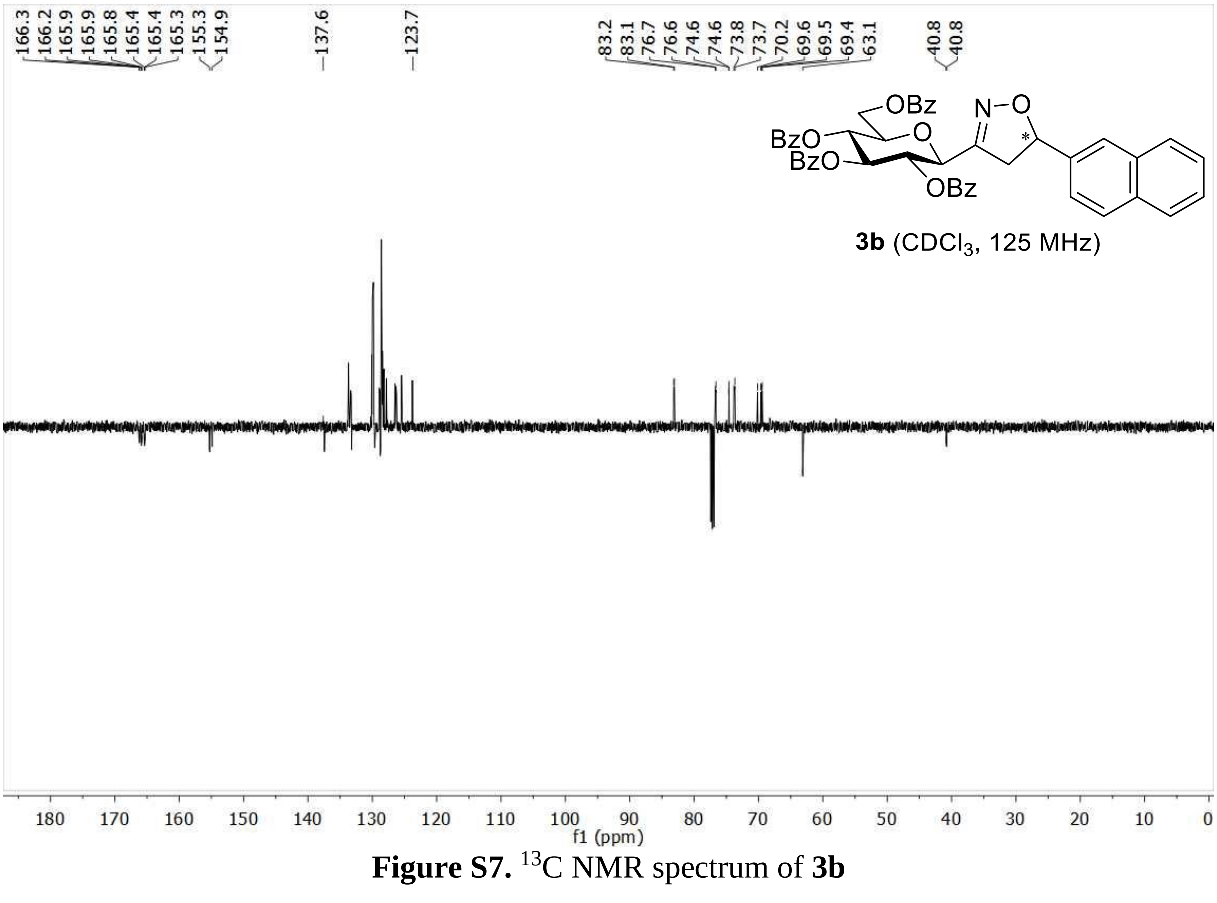

Figure S7. ^{13}C NMR spectrum of **3b**

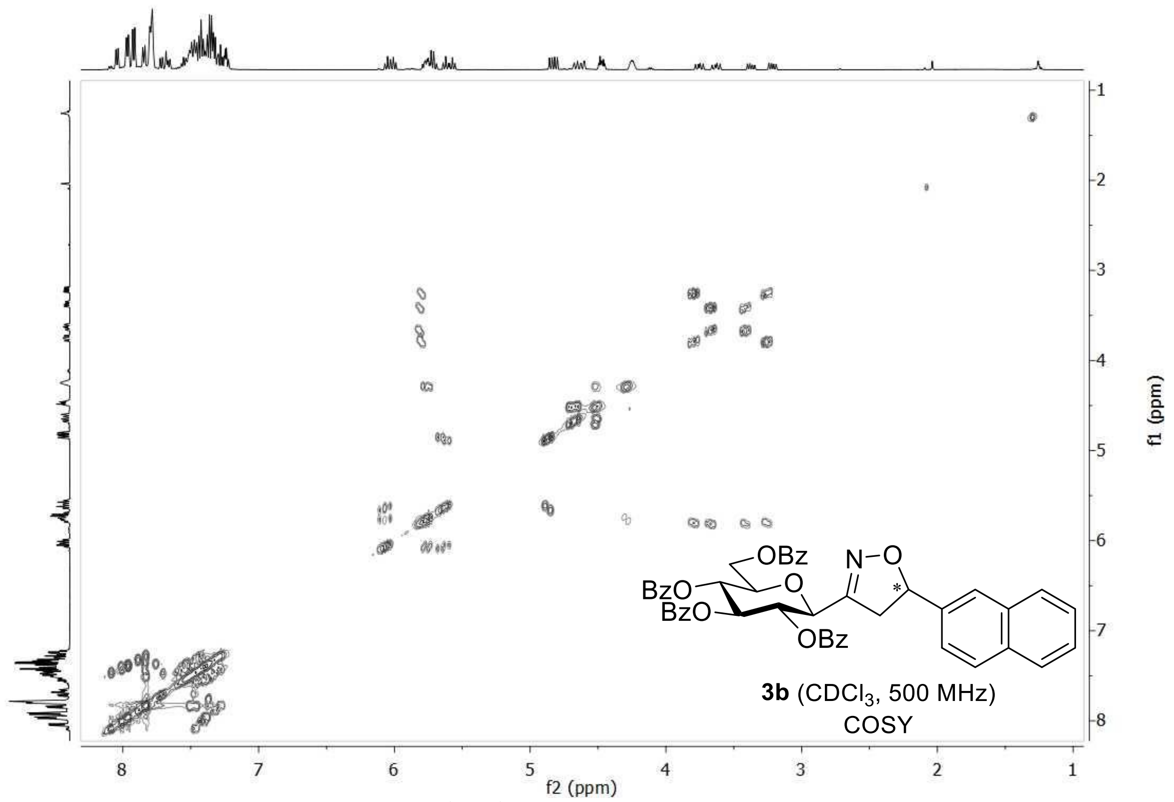

Figure S8. ^{1}H–^{1}H COSY spectrum of **3b**

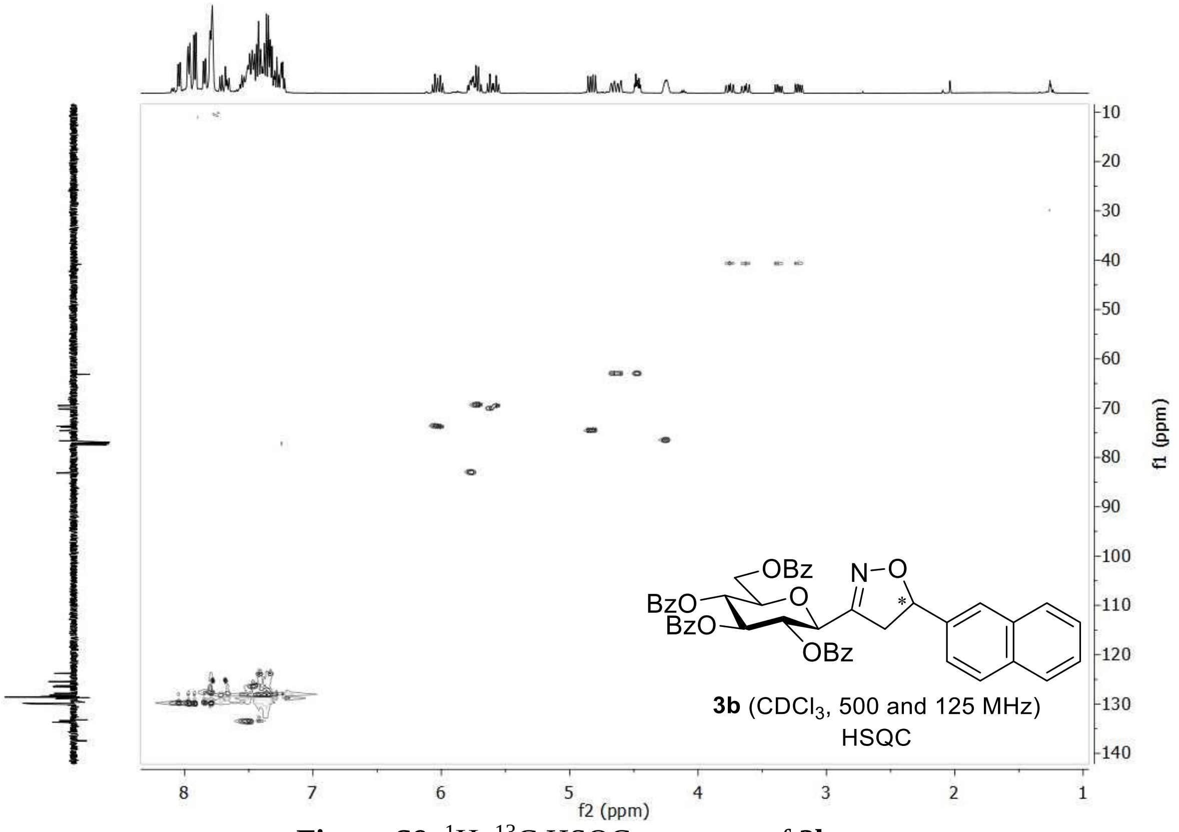

Figure S9. ^{1}H–^{13}C HSQC spectrum of **3b**

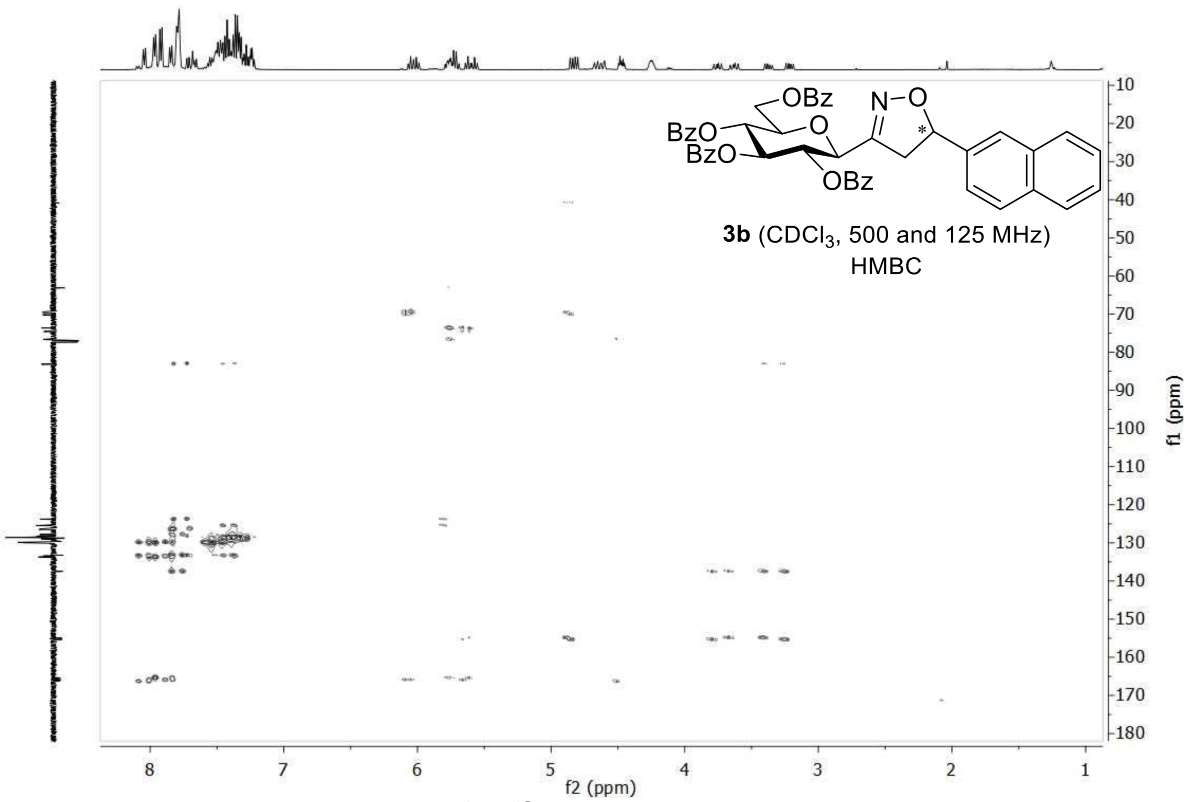

Figure S10. ^{1}H–^{13}C HMBC spectrum of **3b**

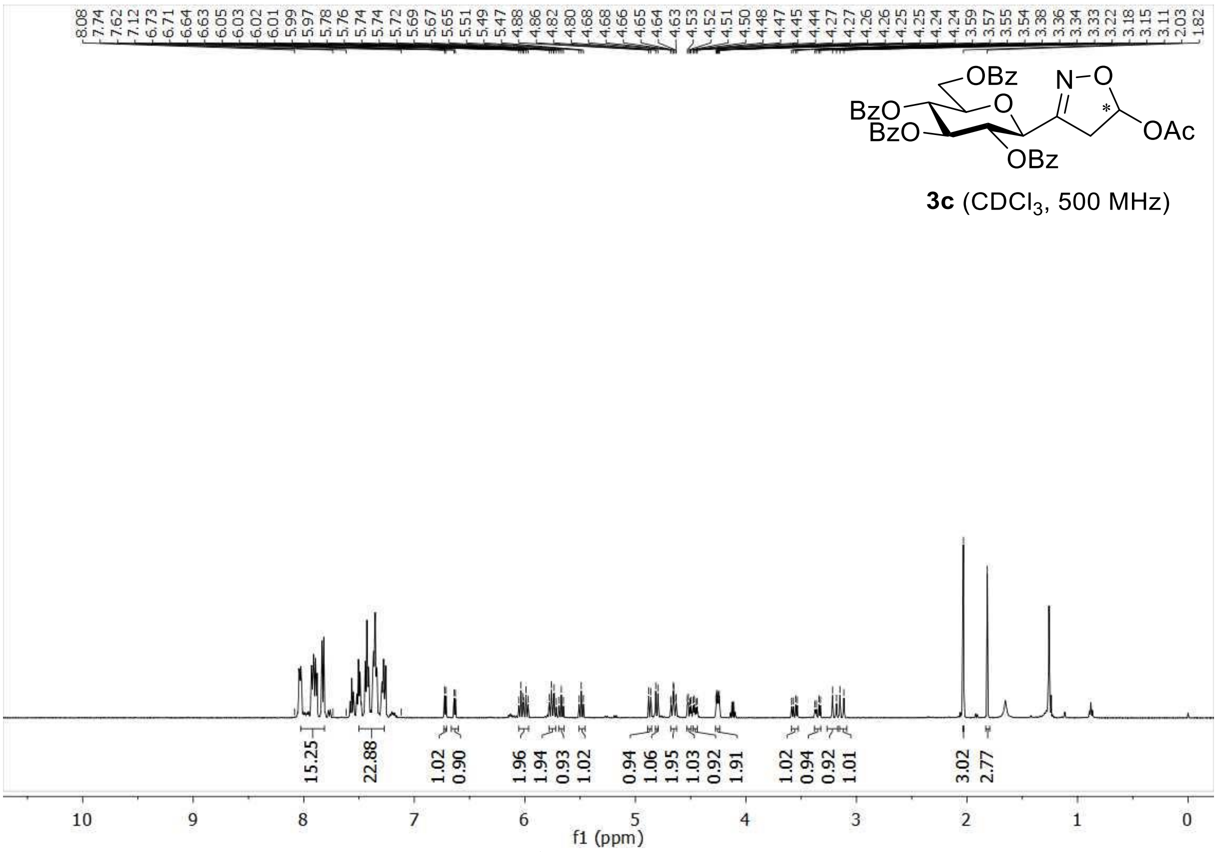

Figure S11. ^{1}H NMR spectrum of **3c**

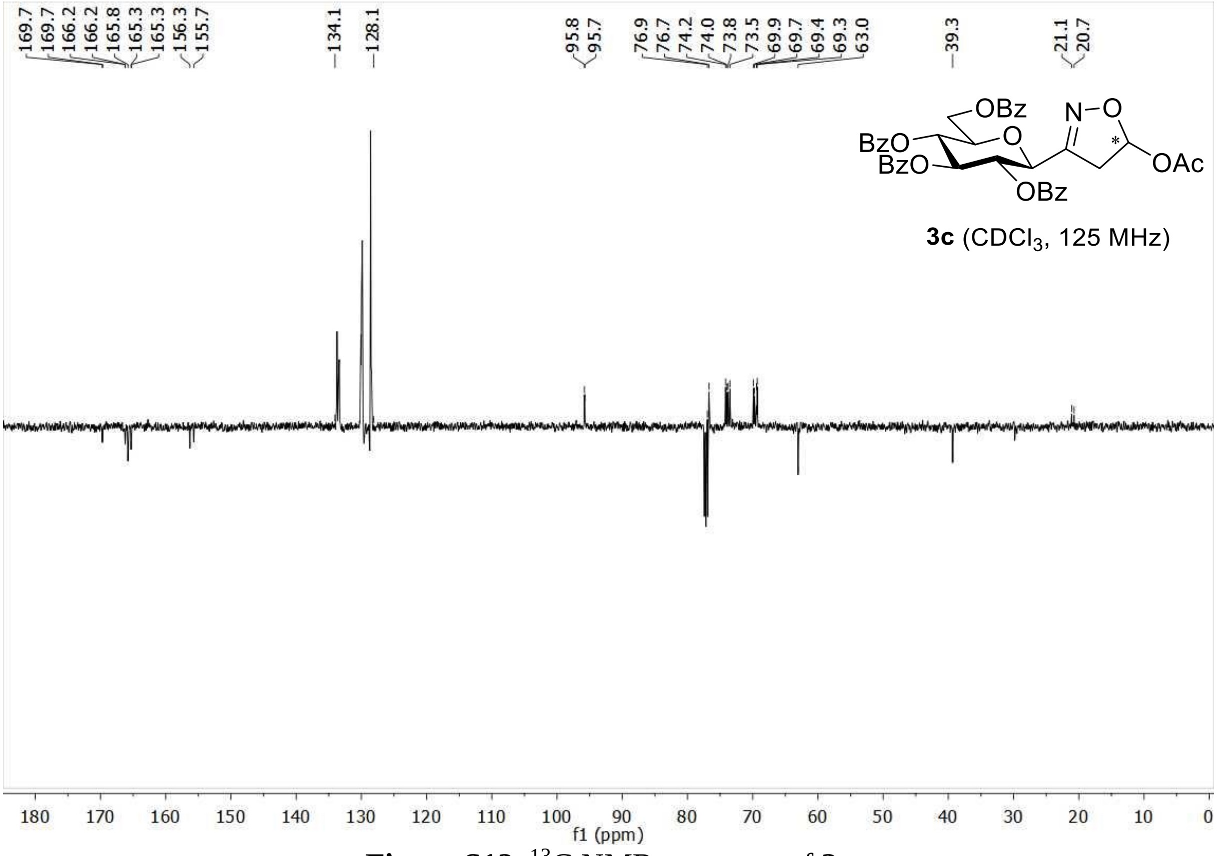

Figure S12. ^{13}C NMR spectrum of **3c**

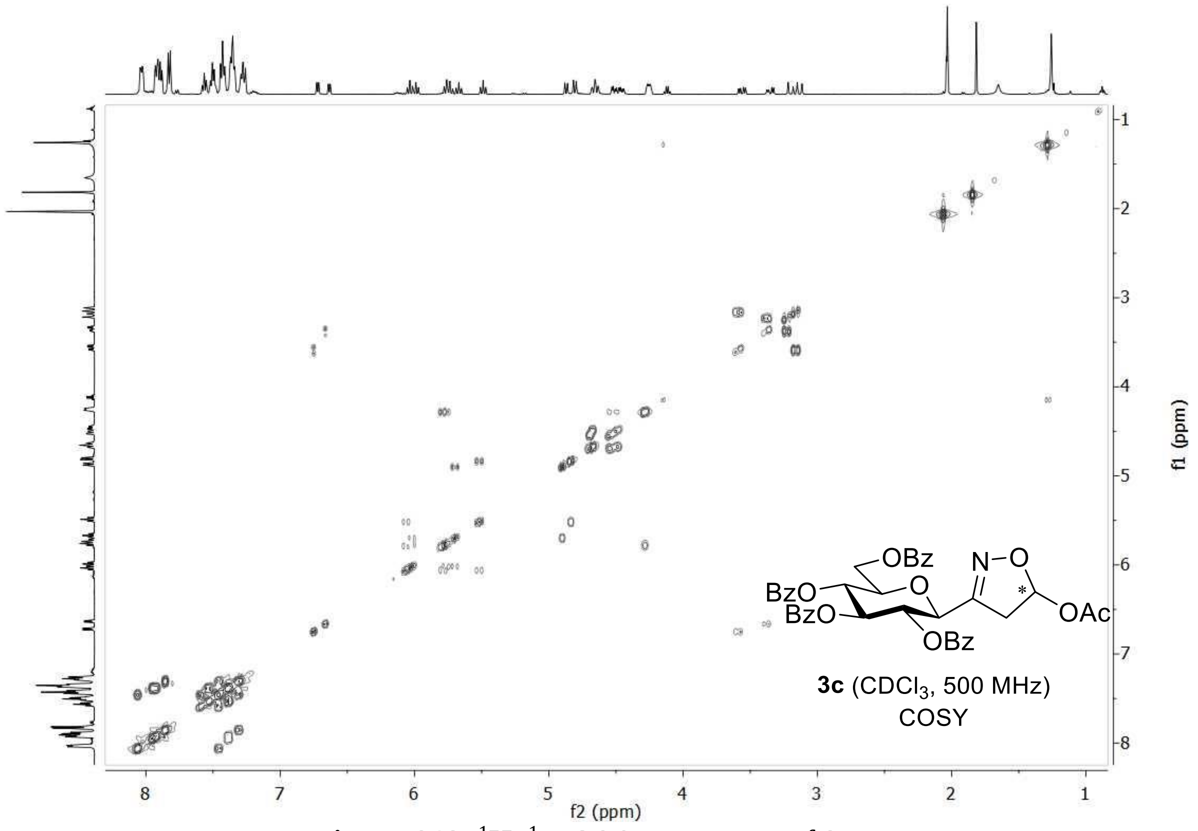

Figure S13. ^{1}H–^{1}H COSY spectrum of **3c**

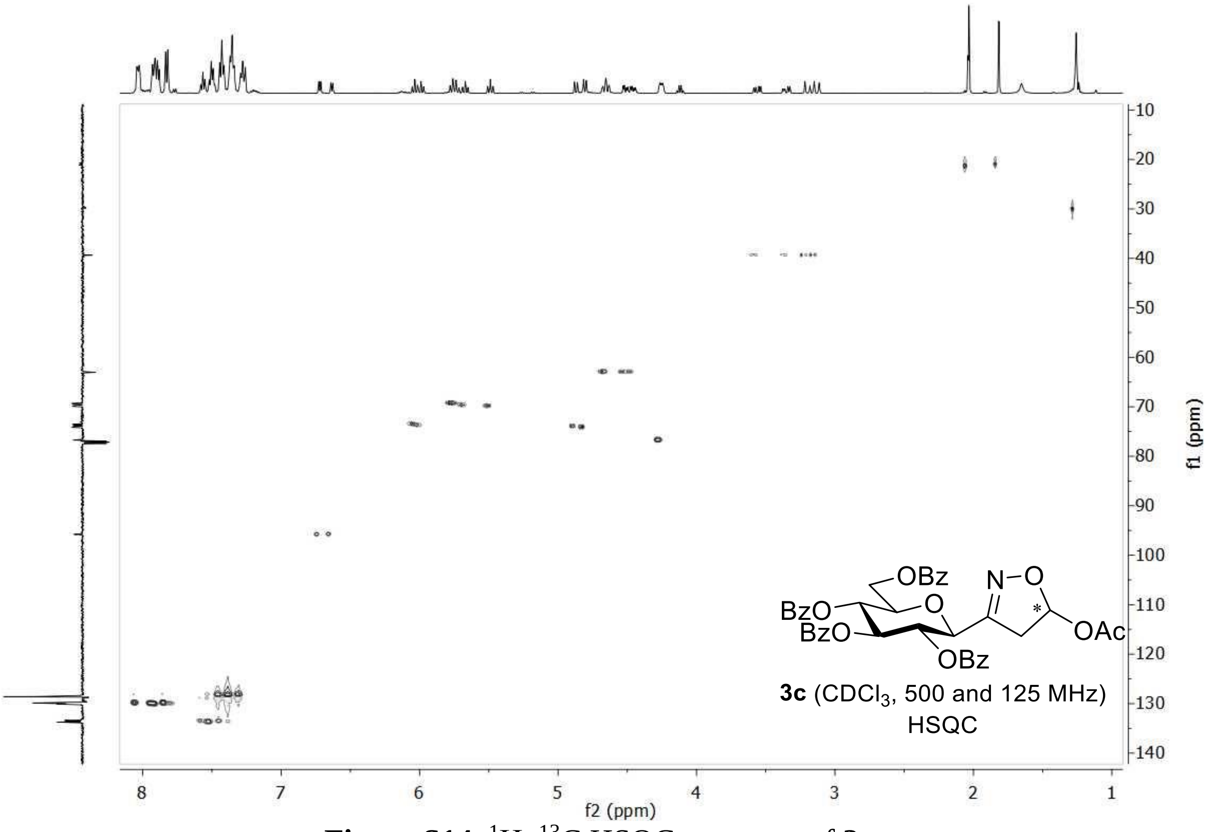

Figure S14. ^{1}H–^{13}C HSQC spectrum of **3c**

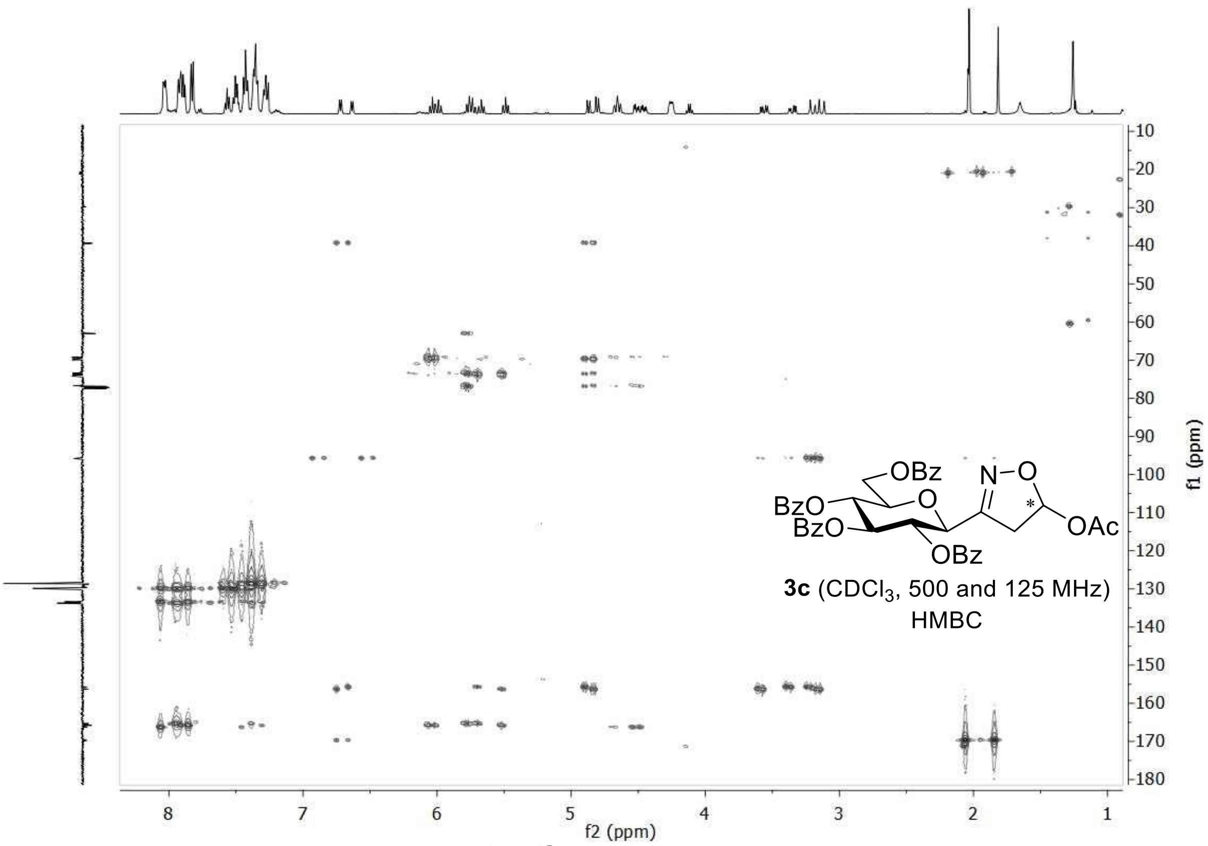

Figure S15. ^{1}H–^{13}C HMBC spectrum of **3c**

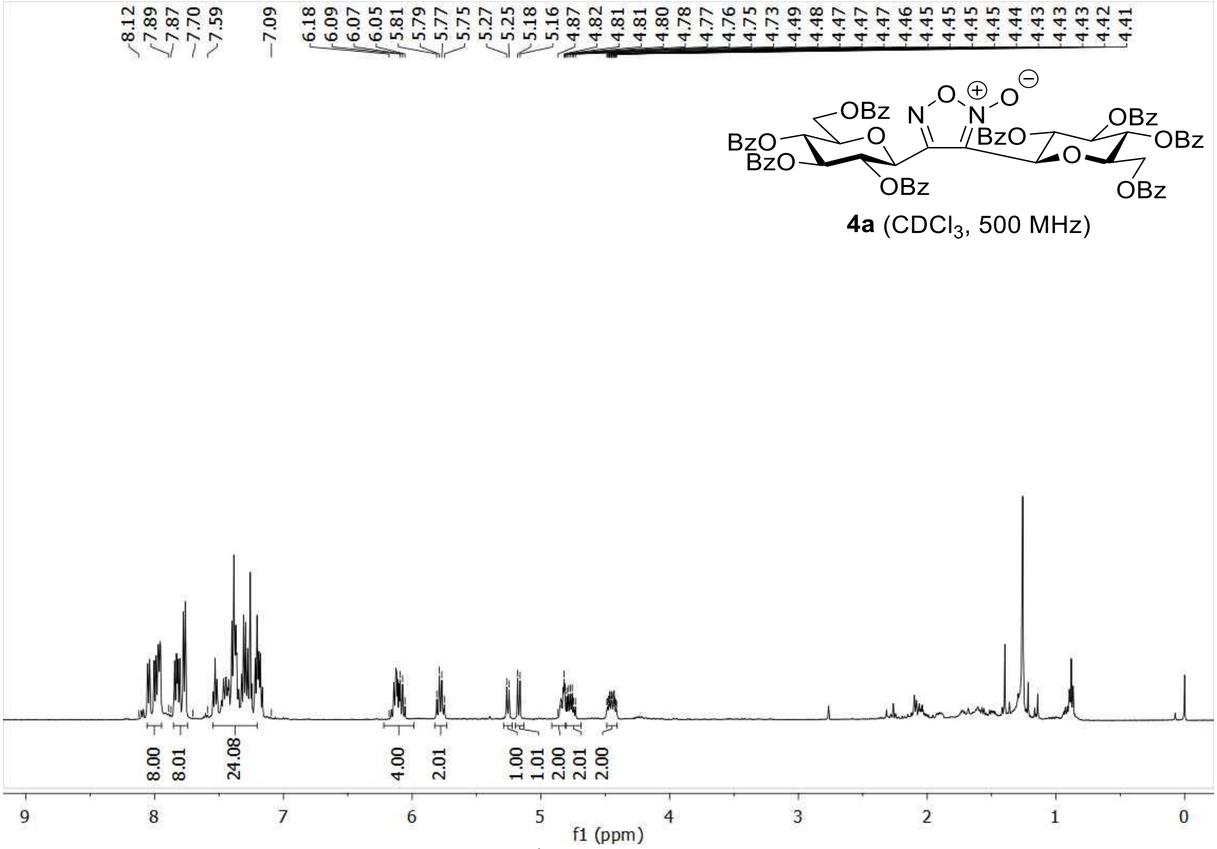

Figure S16. ^{1}H NMR spectrum of **4a**

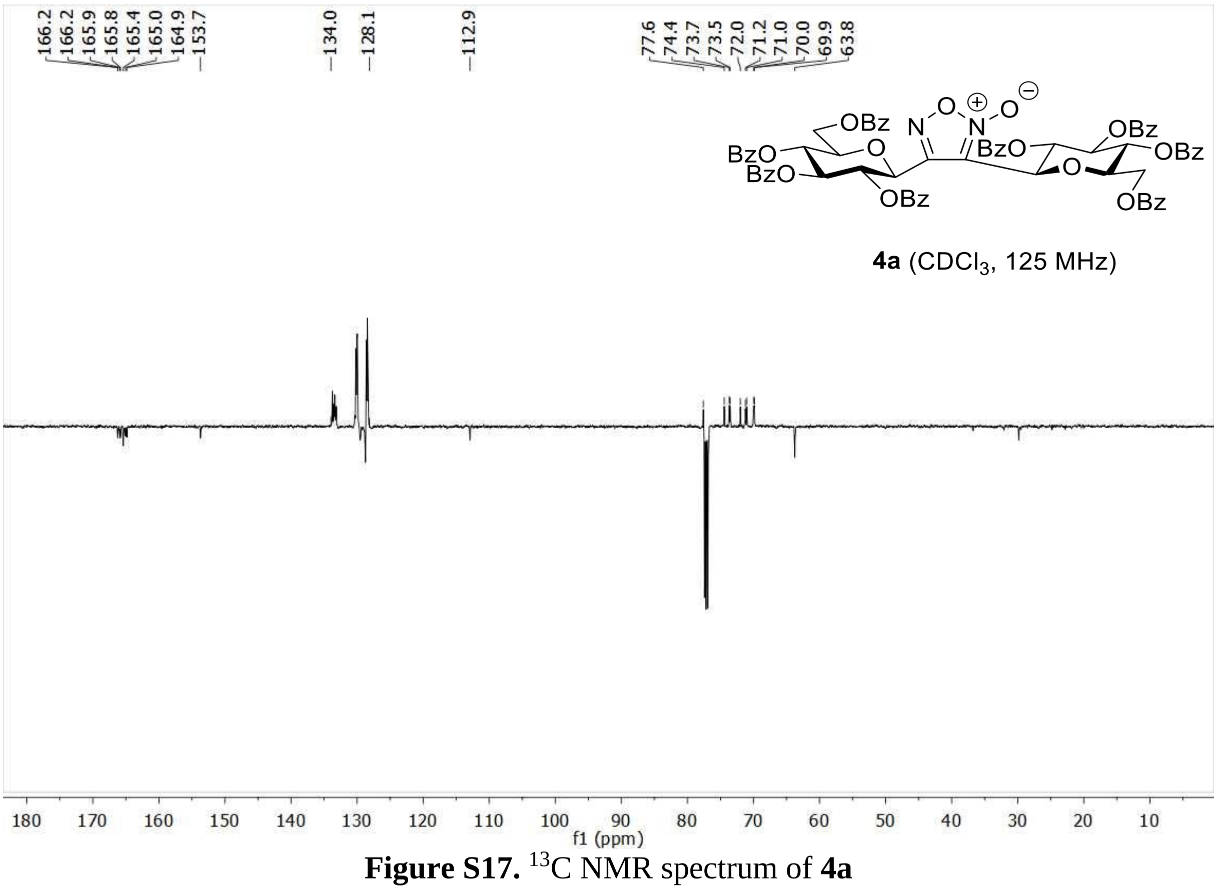

Figure S17. ^{13}C NMR spectrum of **4a**

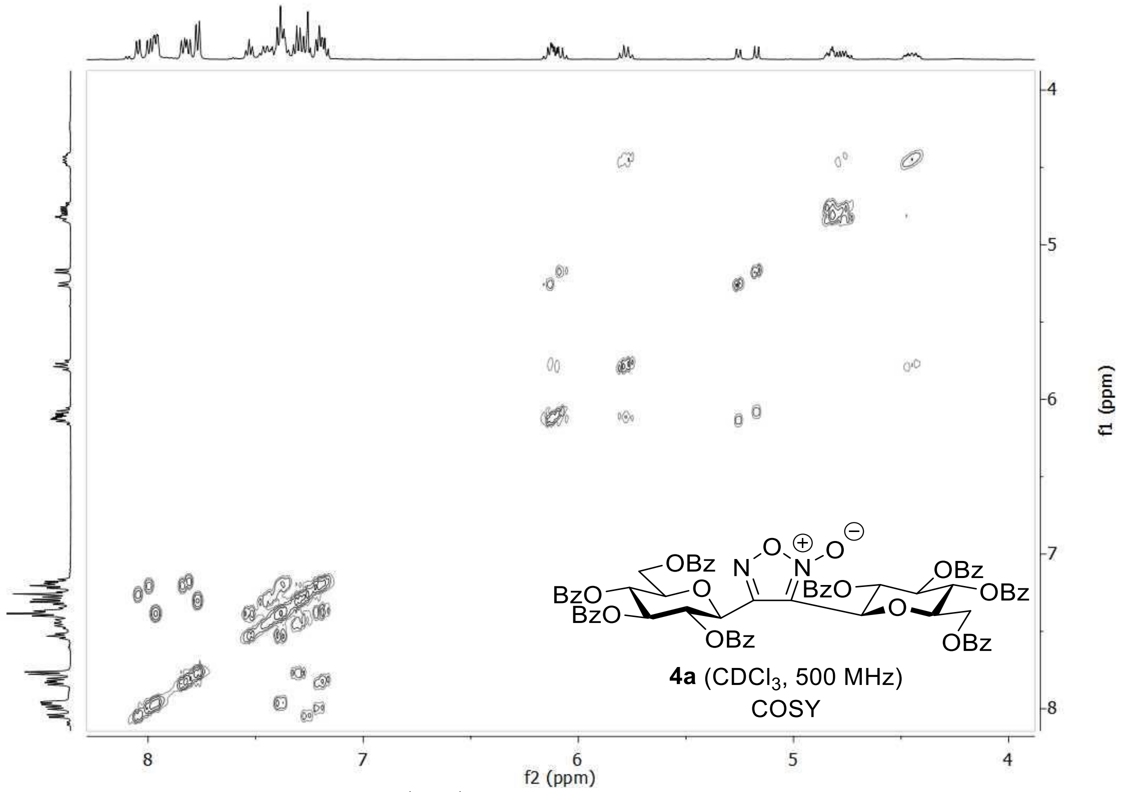

Figure S18. ^{1}H–^{1}H COSY spectrum of **4a**

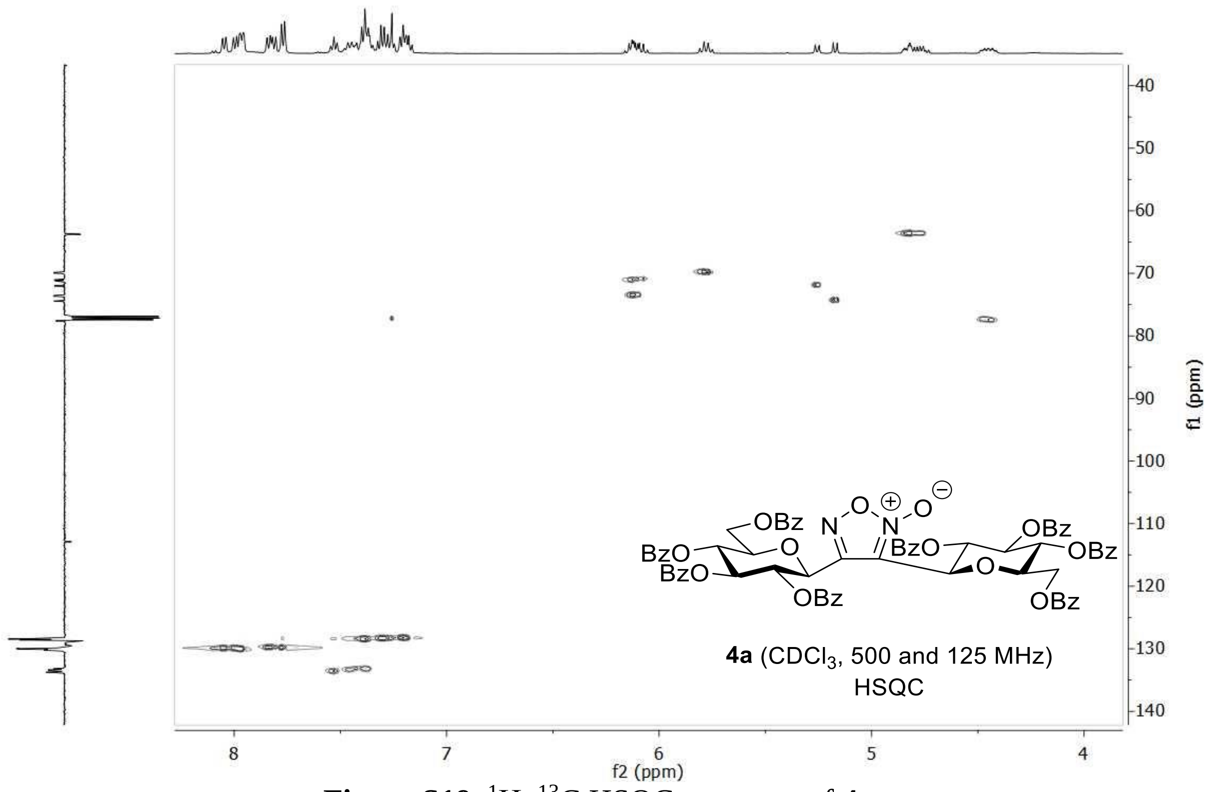

Figure S19. ^{1}H–^{13}C HSQC spectrum of **4a**

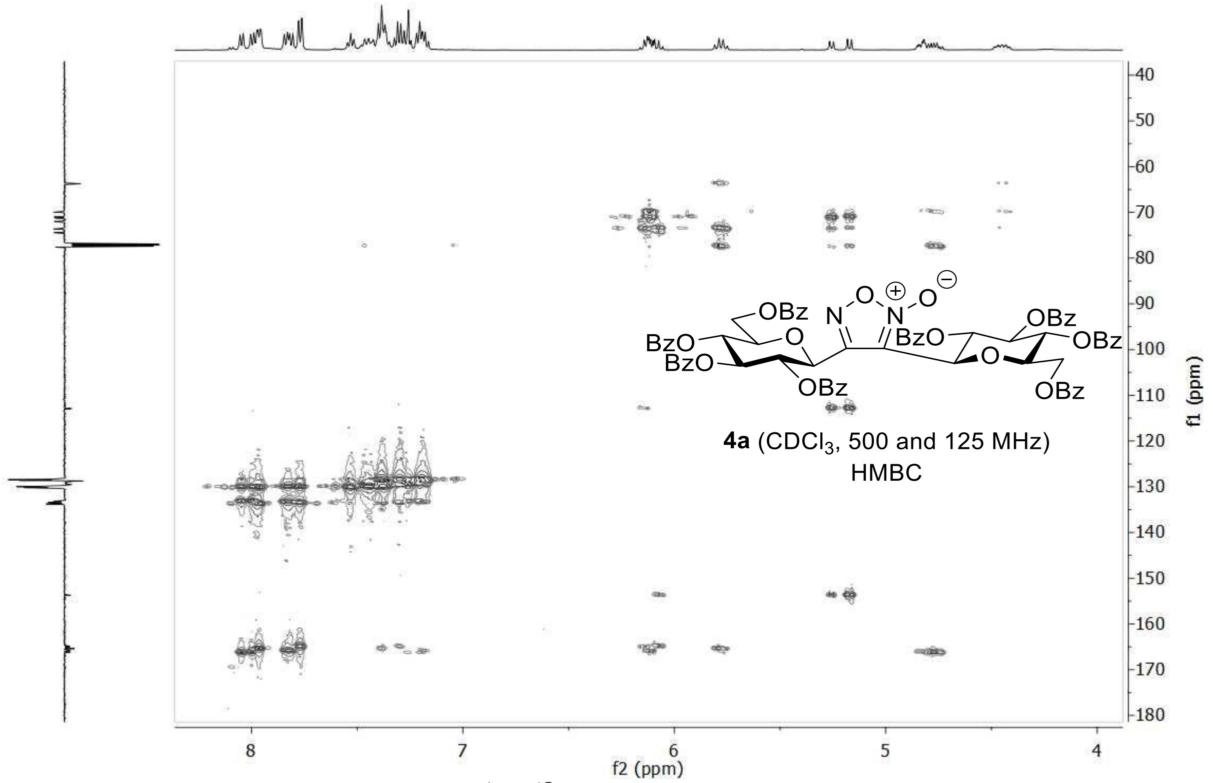

Figure S20. ^{1}H–^{13}C HMBC spectrum of **4a**

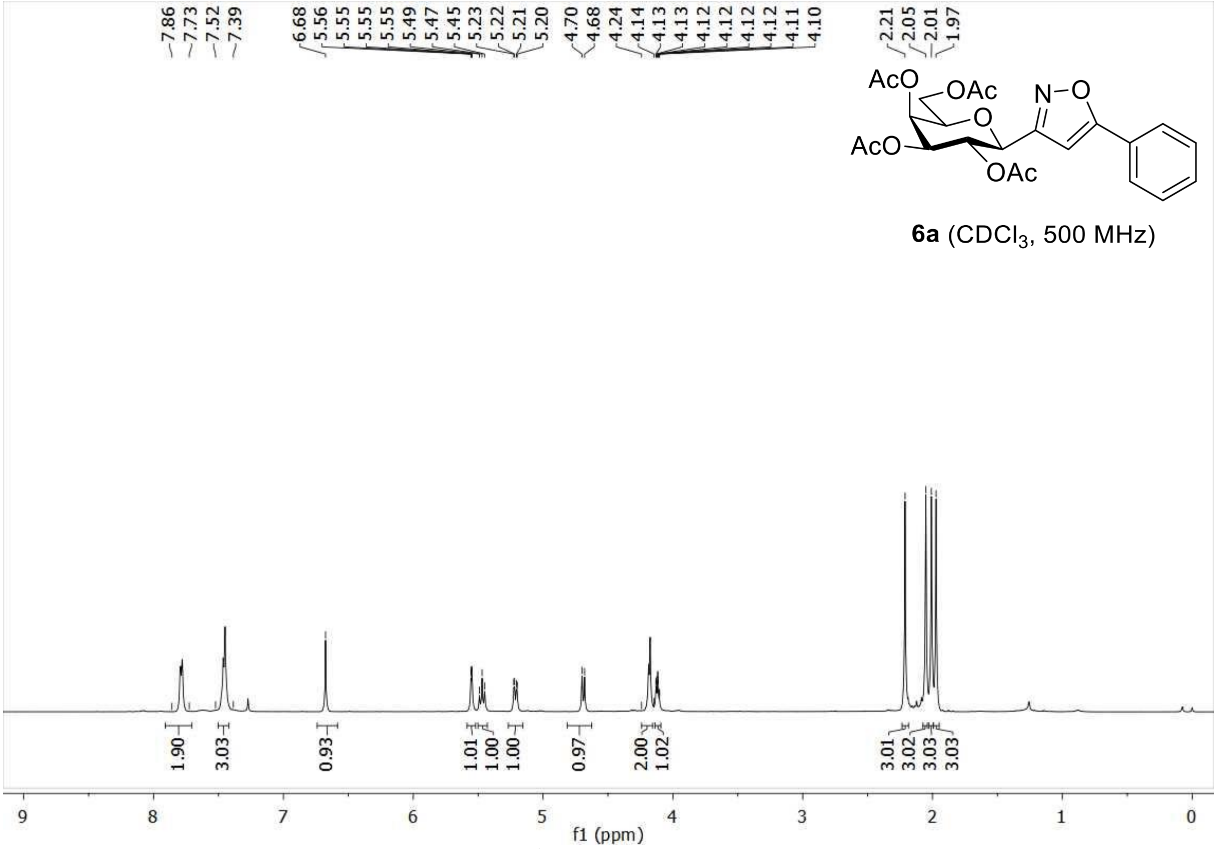

Figure S21. ^{1}H NMR spectrum of **6a**

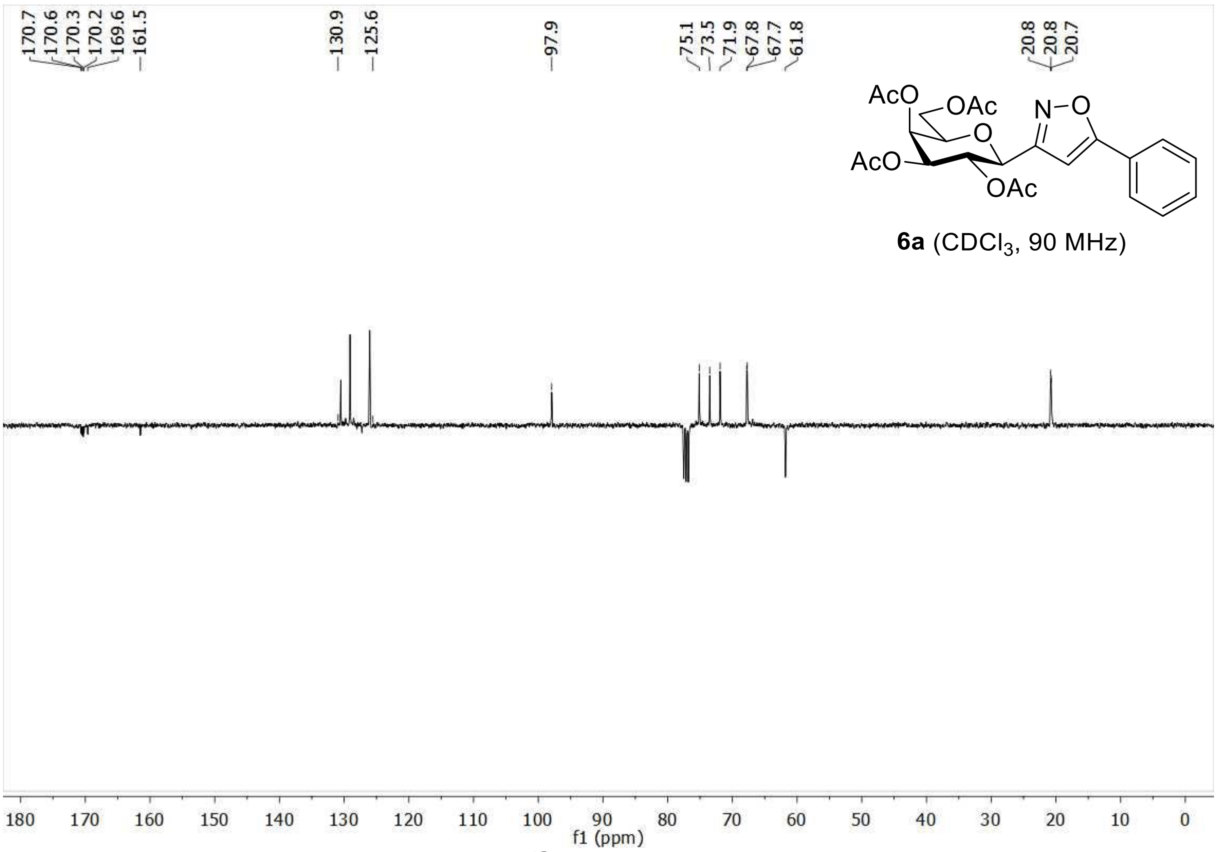

Figure S22. ^{13}C NMR spectrum of **6a**

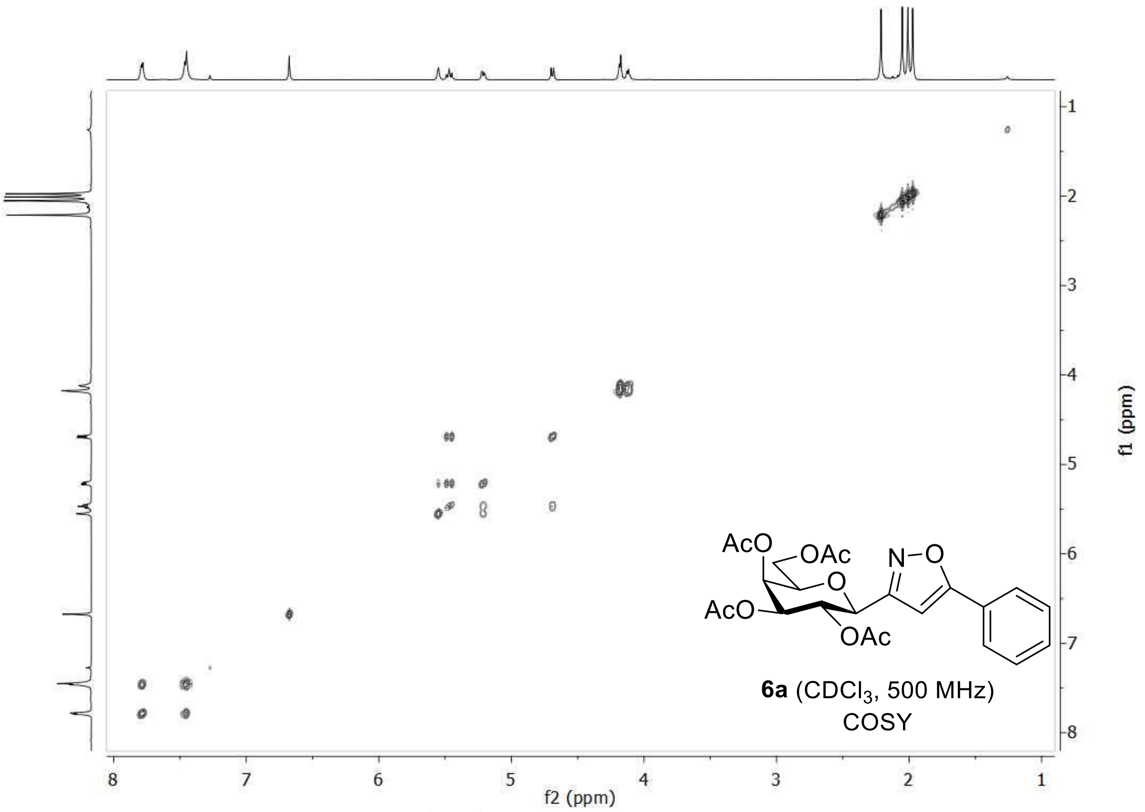

Figure S23. ^{1}H–^{1}H COSY spectrum of **6a**

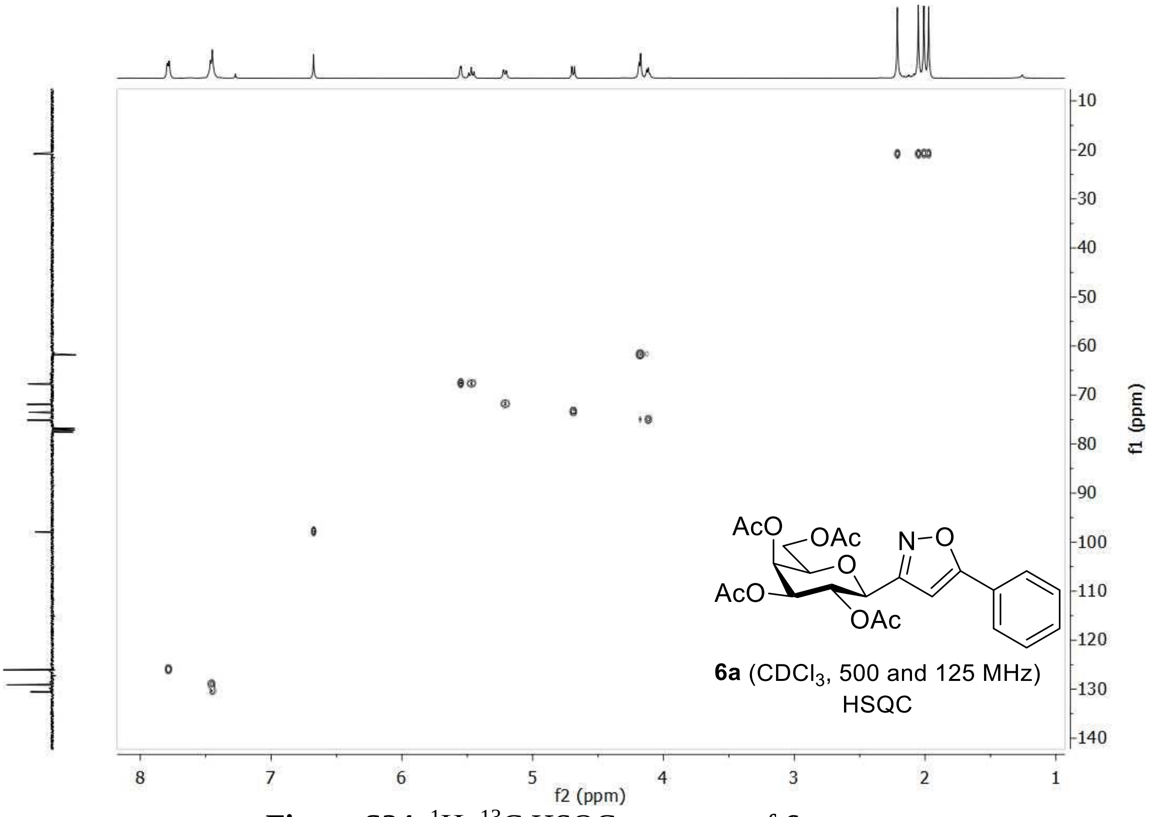

Figure S24. ^{1}H–^{13}C HSQC spectrum of **6a**

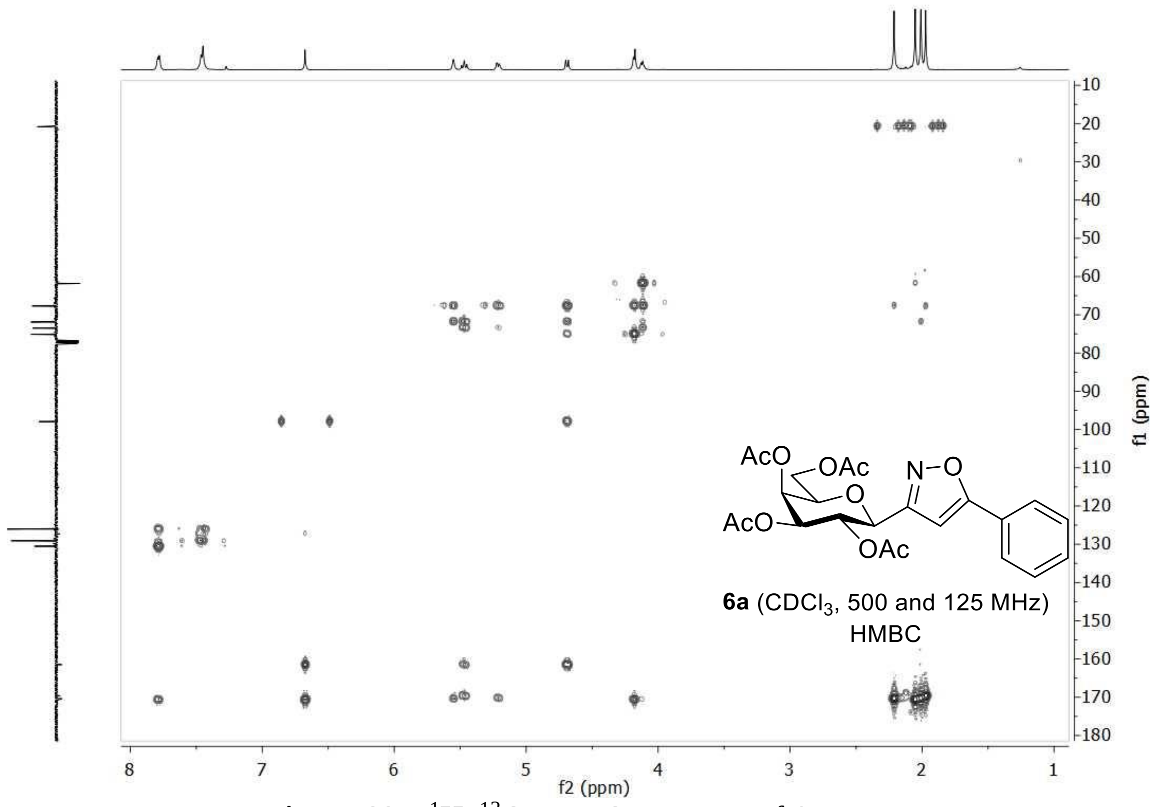

Figure S25. ^{1}H–^{13}C HMBC spectrum of **6a**

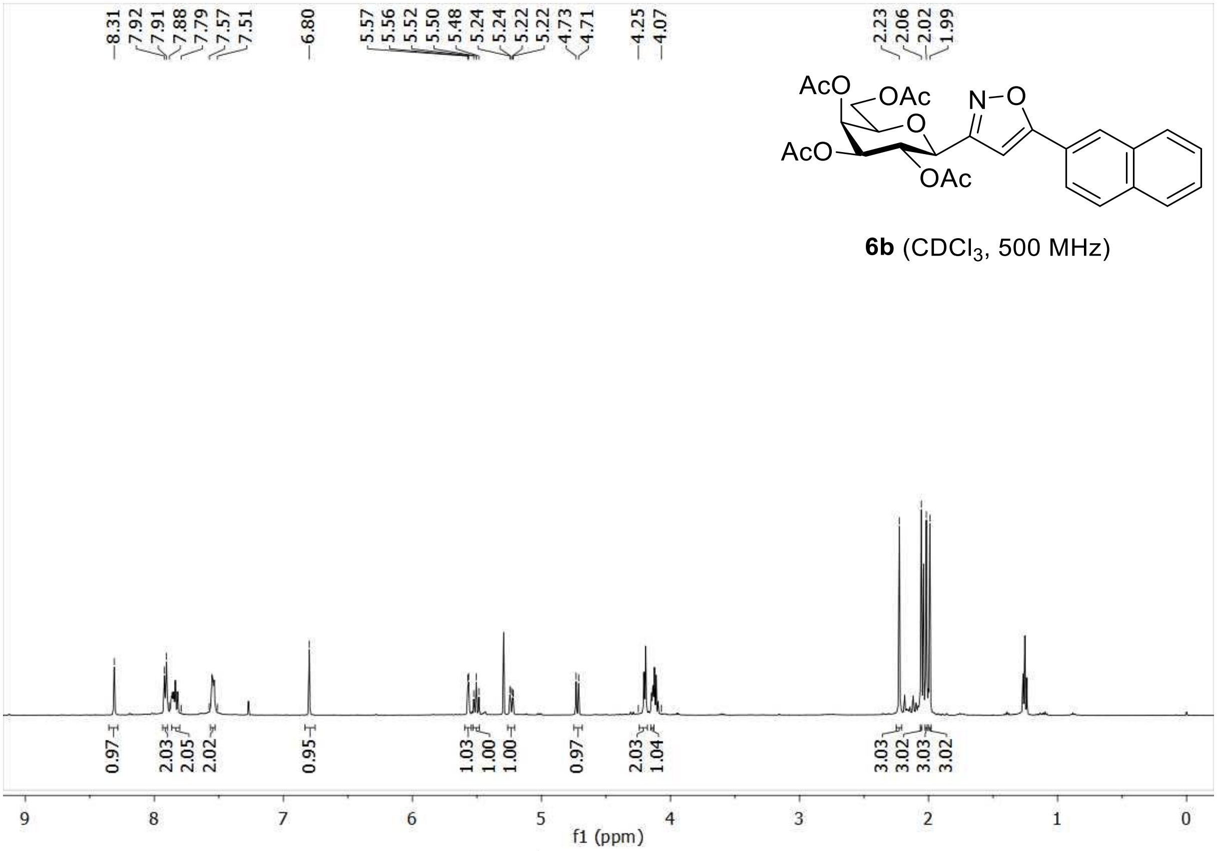

Figure S26. ^{1}H NMR spectrum of **6b**

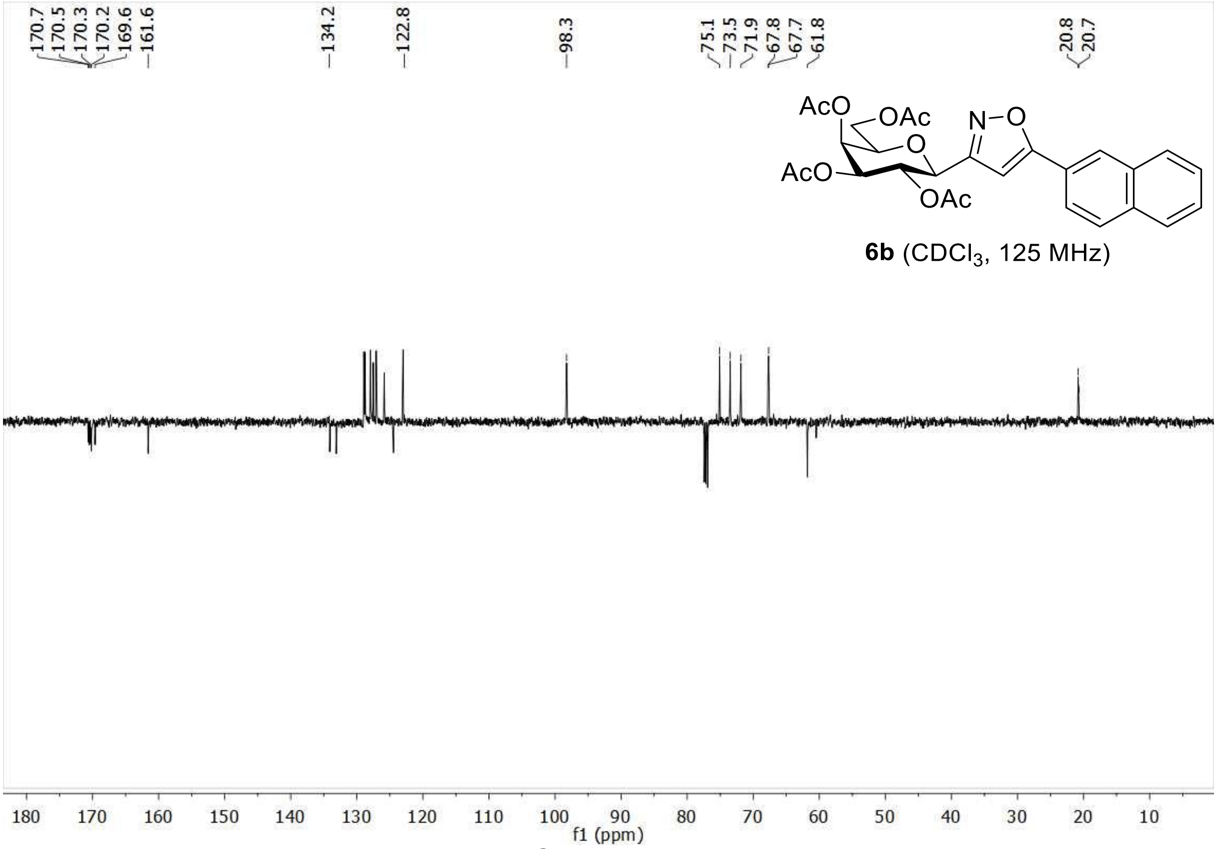

Figure S27. ^{13}C NMR spectrum of **6b**

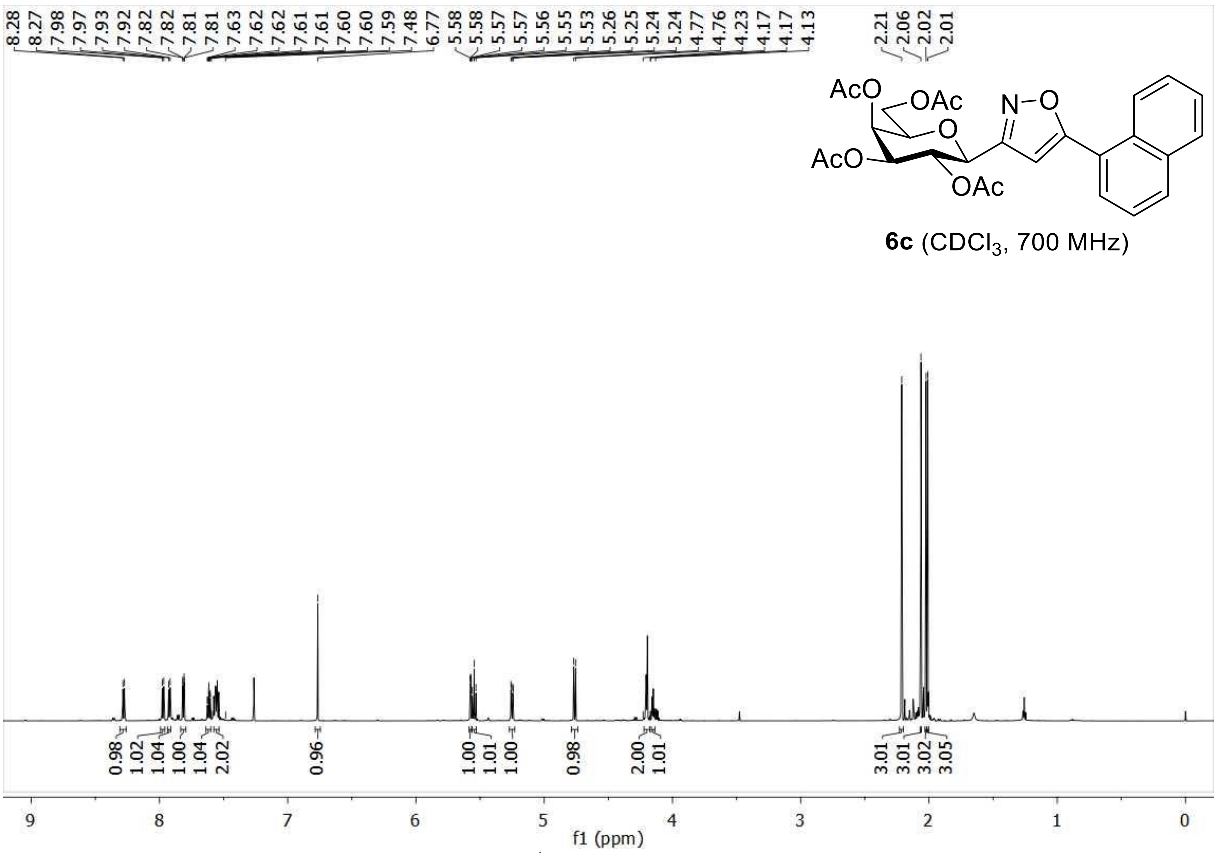

Figure S28. ^{1}H NMR spectrum of **6c**

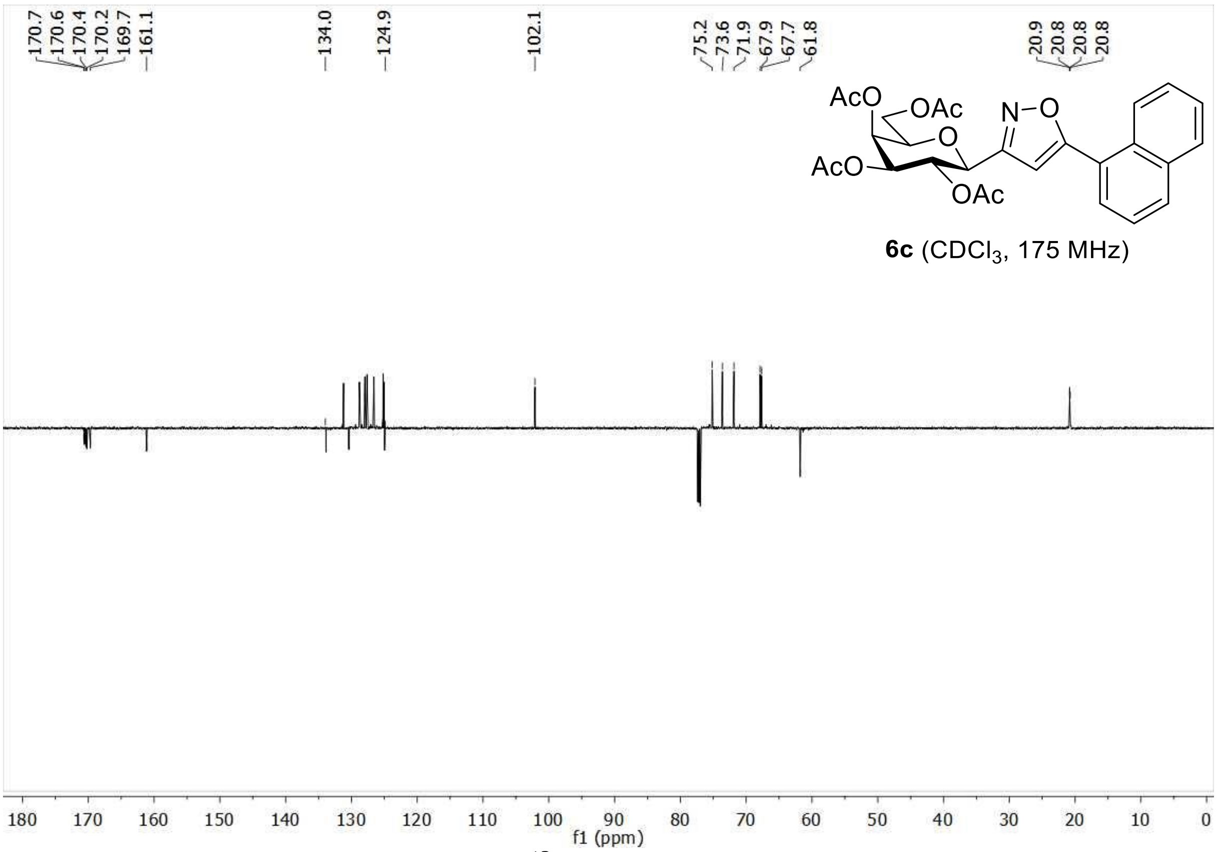

Figure S29. ^{13}C NMR spectrum of **6c**

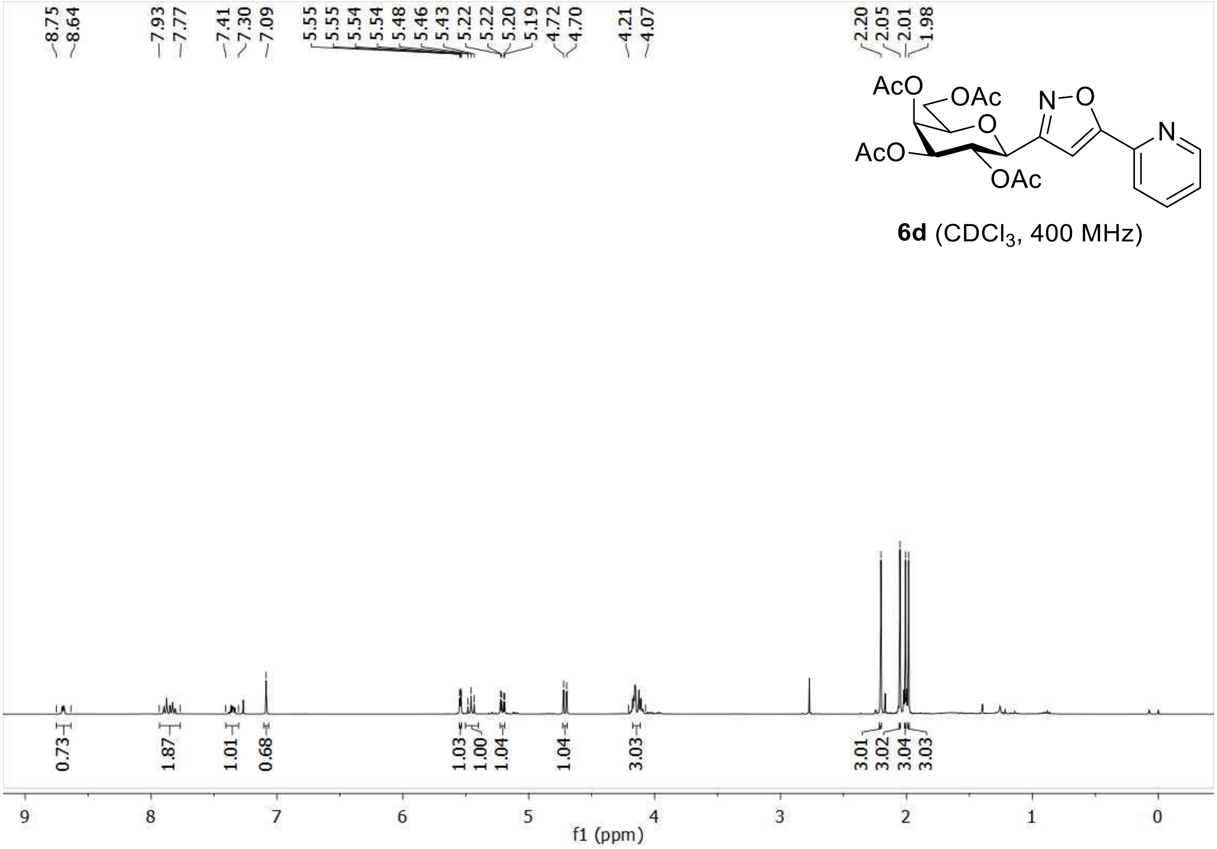

Figure S30. ^{1}H NMR spectrum of **6d**

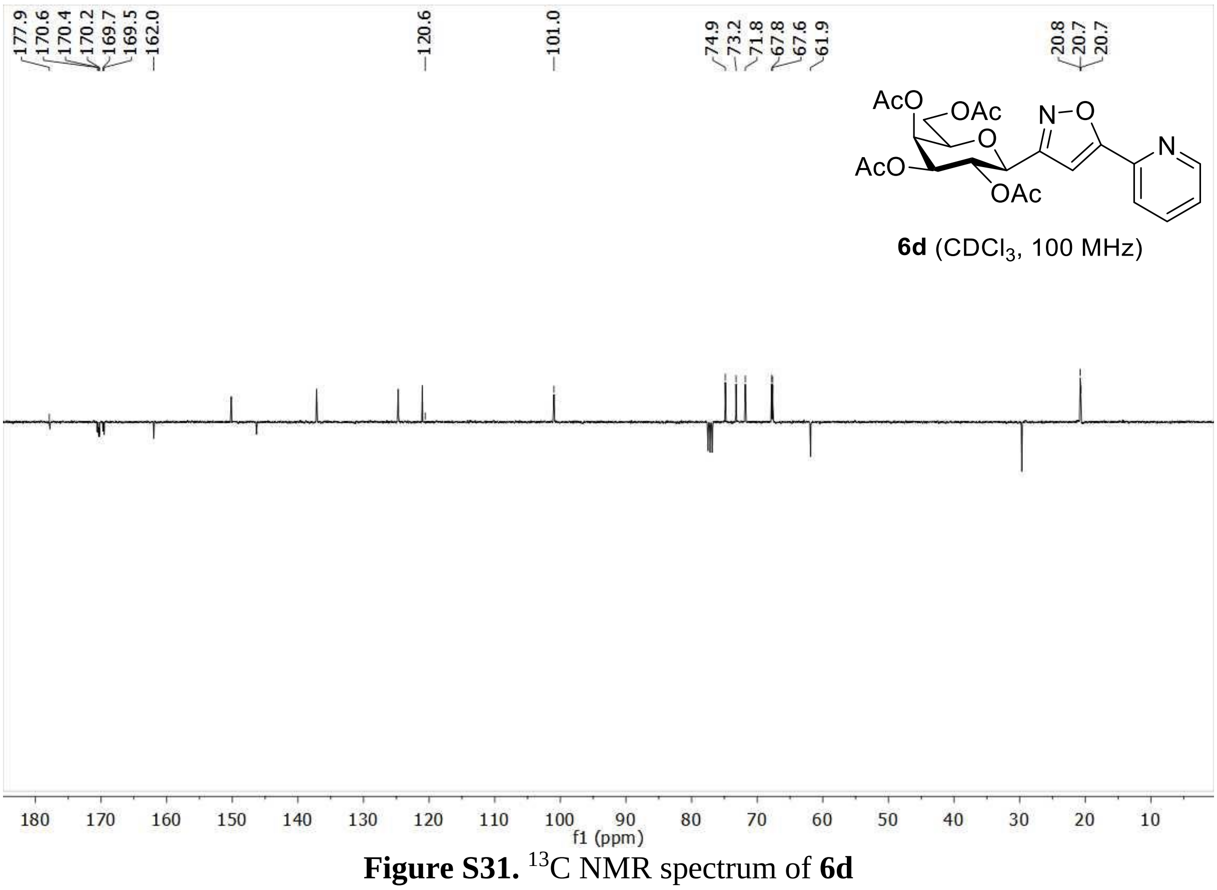

Figure S31. ^{13}C NMR spectrum of **6d**

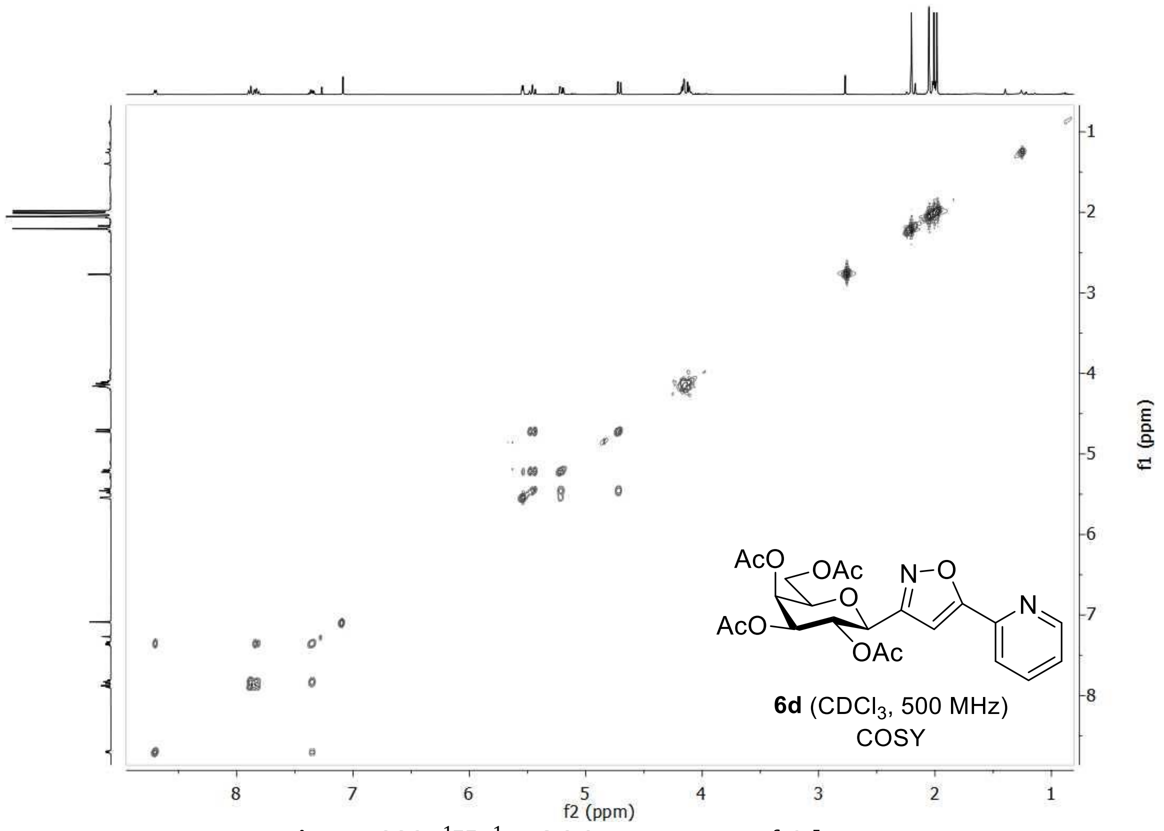

Figure S32. ^{1}H–^{1}H COSY spectrum of **6d**

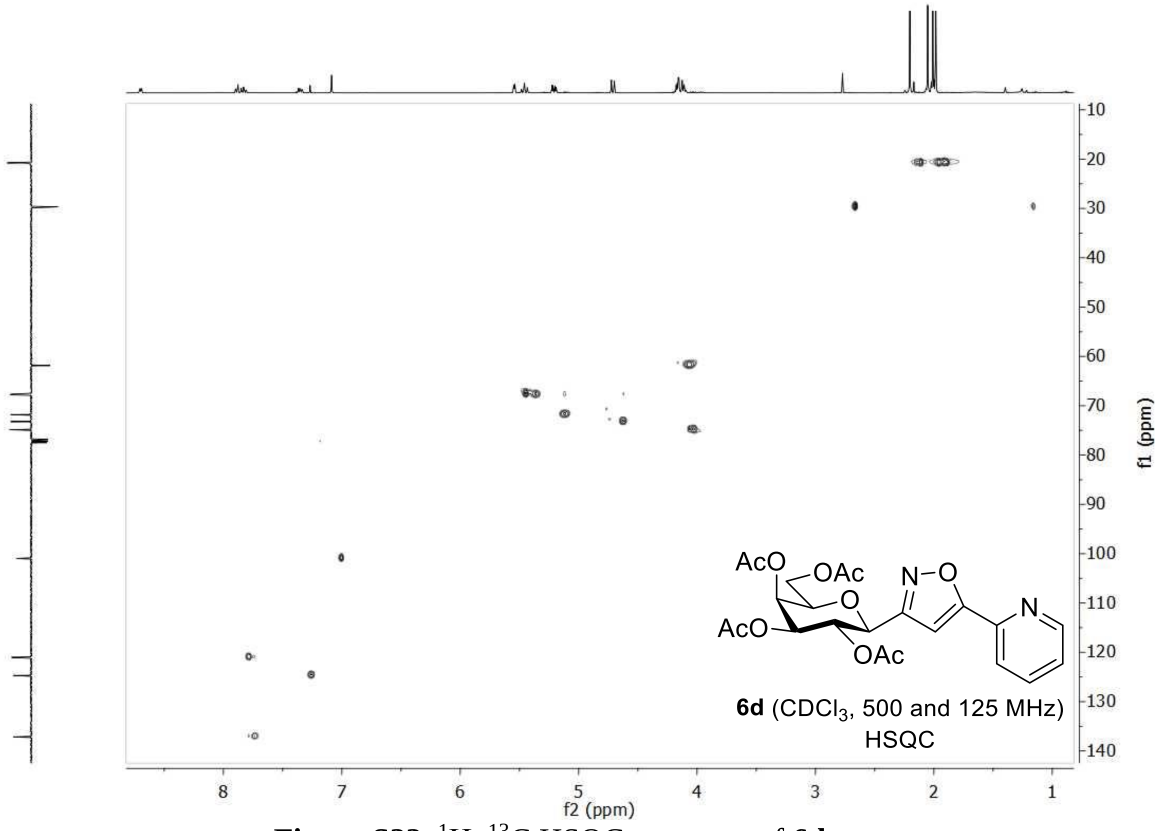

Figure S33. ^{1}H–^{13}C HSQC spectrum of **6d**

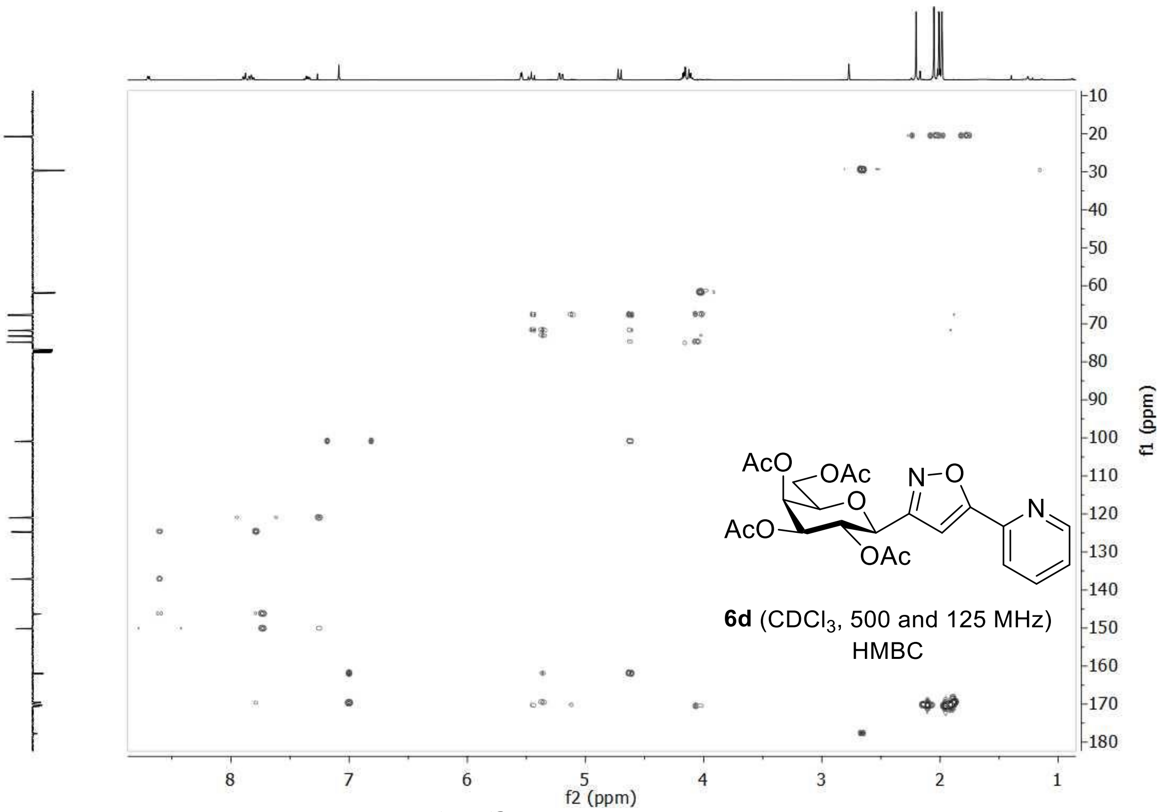

Figure S34. ^{1}H–^{13}C HMBC spectrum of **6d**

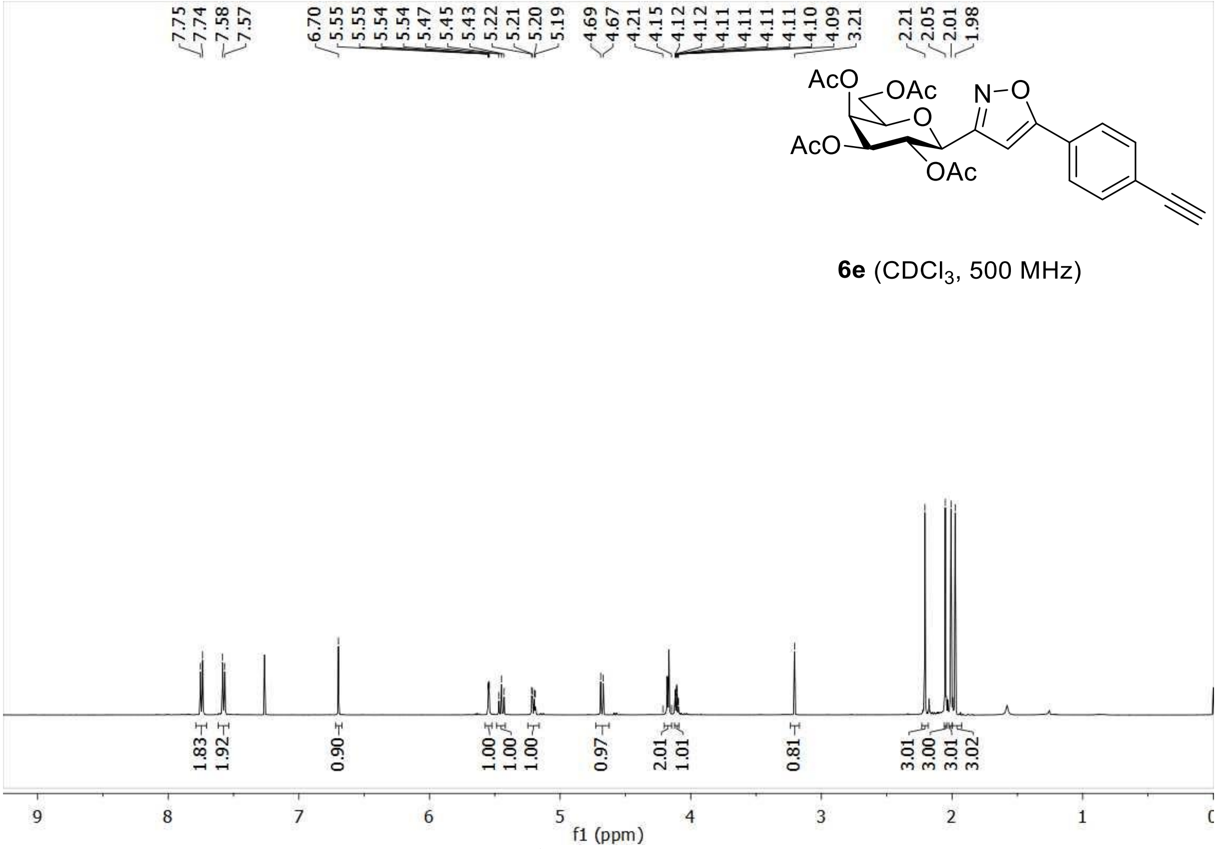

Figure S35. ^{1}H NMR spectrum of **6e**

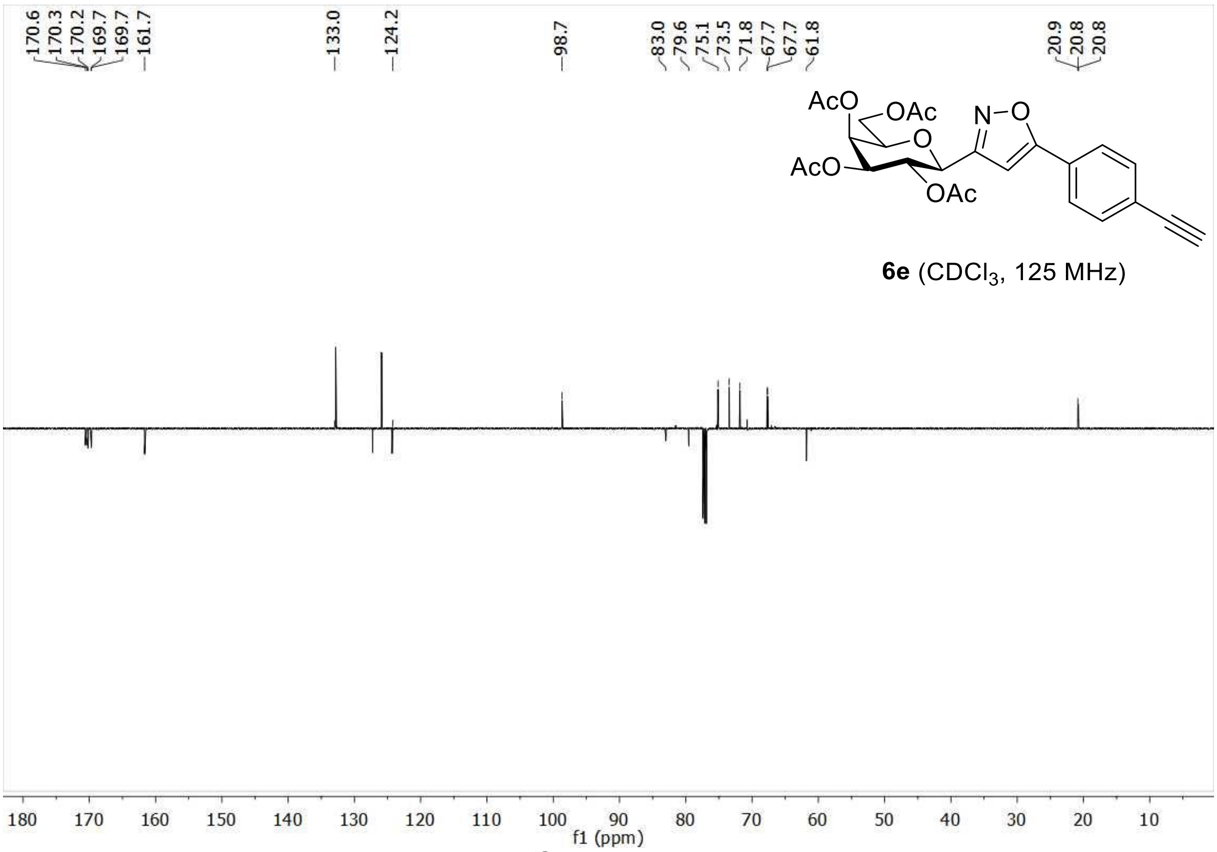

Figure S36. ^{13}C NMR spectrum of **6e**

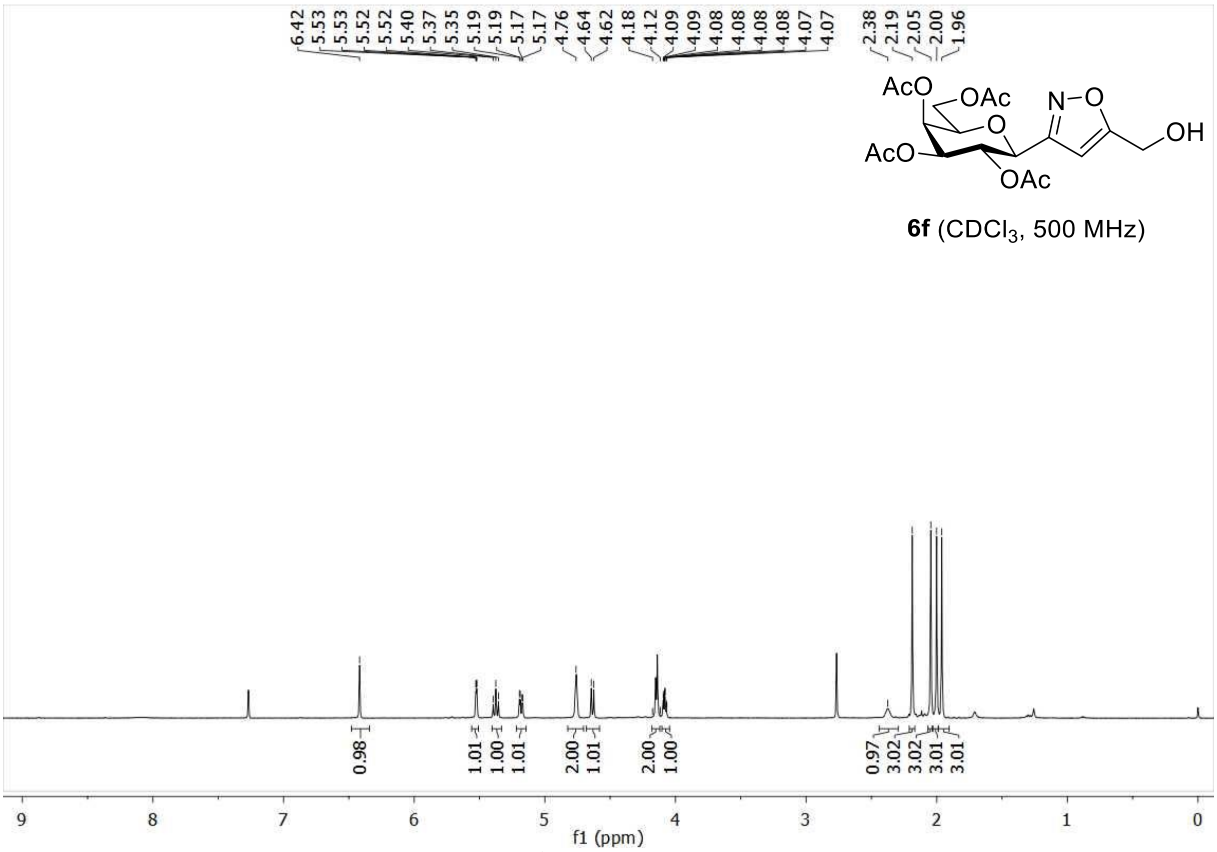

Figure S37. ^{1}H NMR spectrum of **6f**

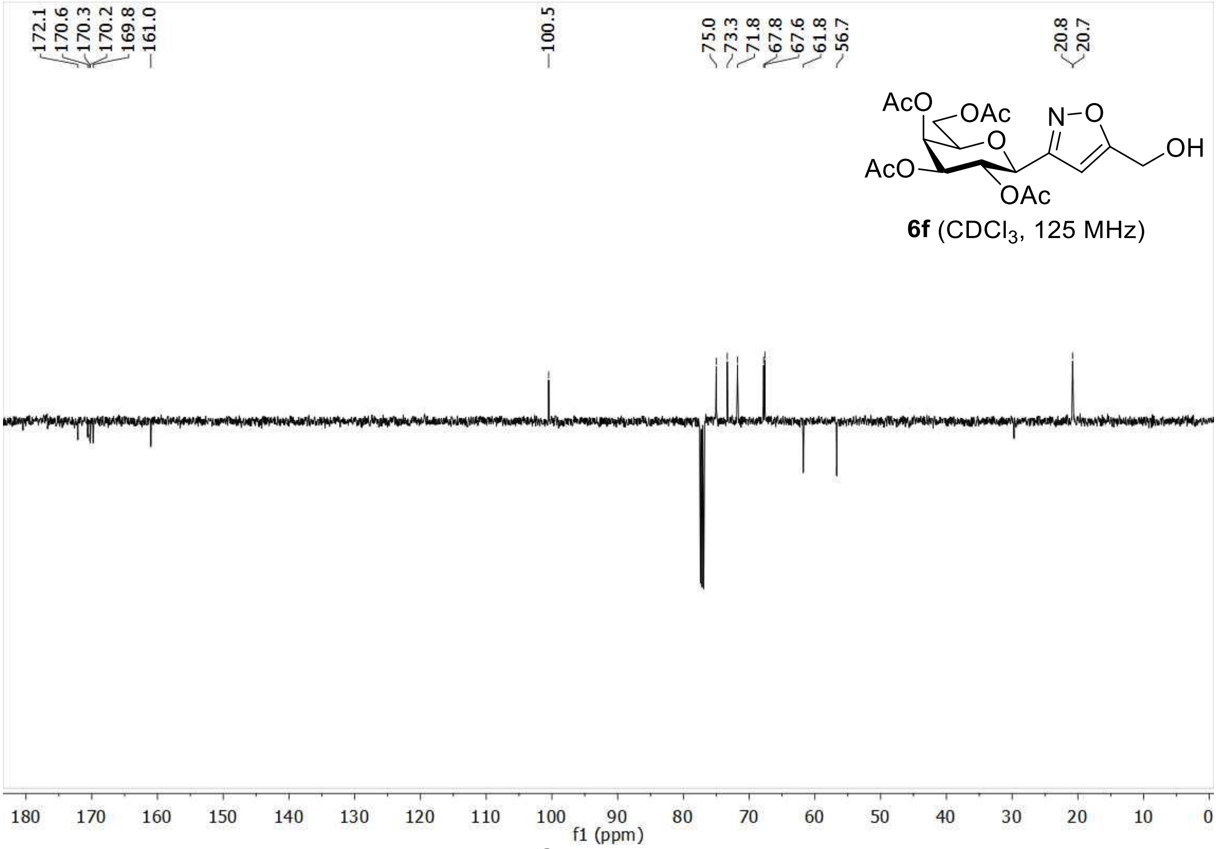

Figure S38. ^{13}C NMR spectrum of **6f**

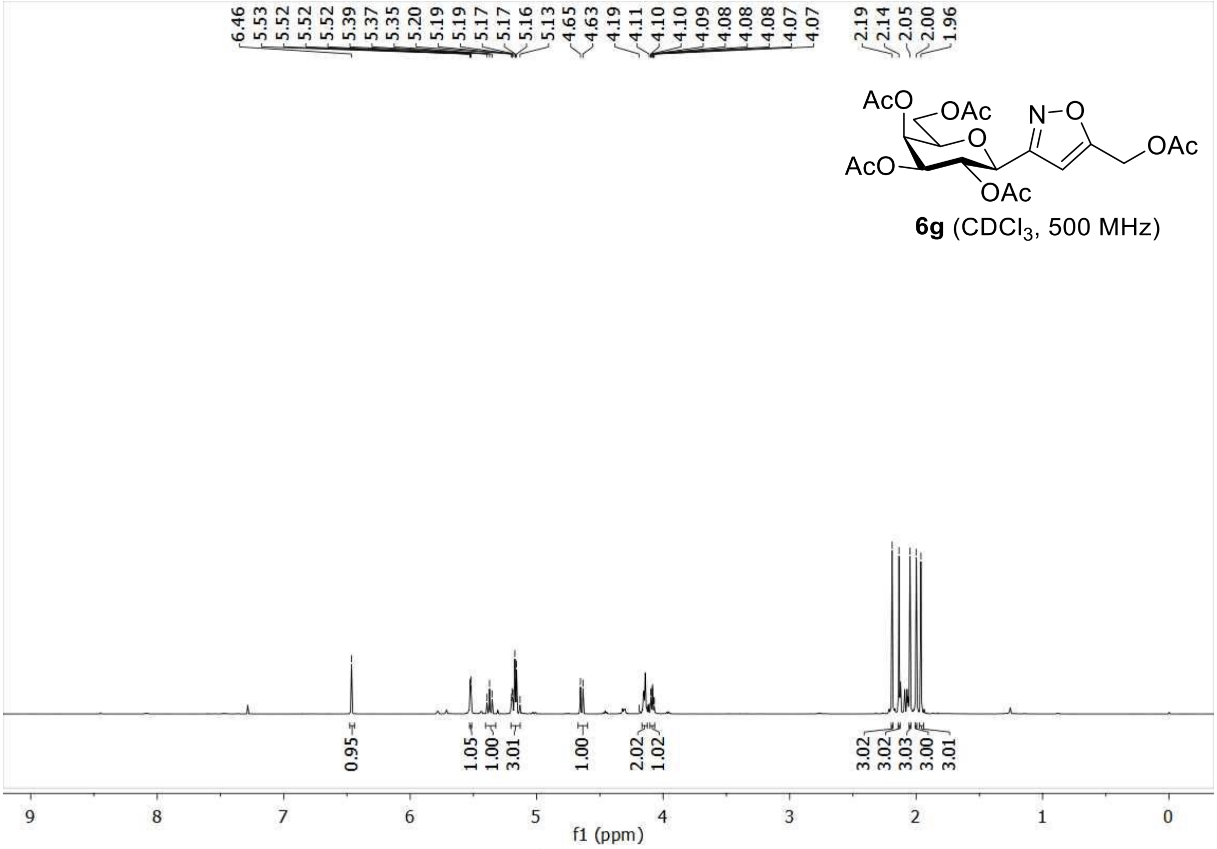

Figure S39. ^{1}H NMR spectrum of **6g**

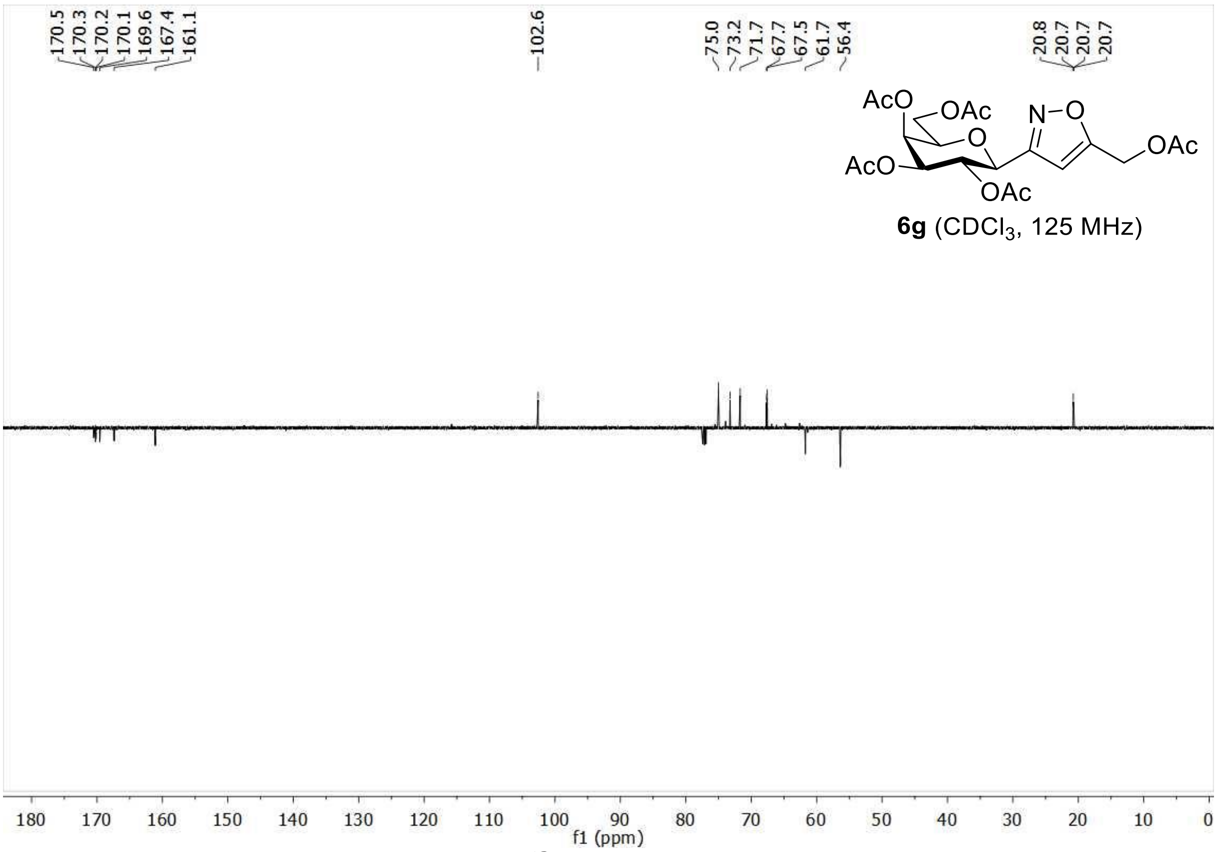

Figure S40. ^{13}C NMR spectrum of **6g**

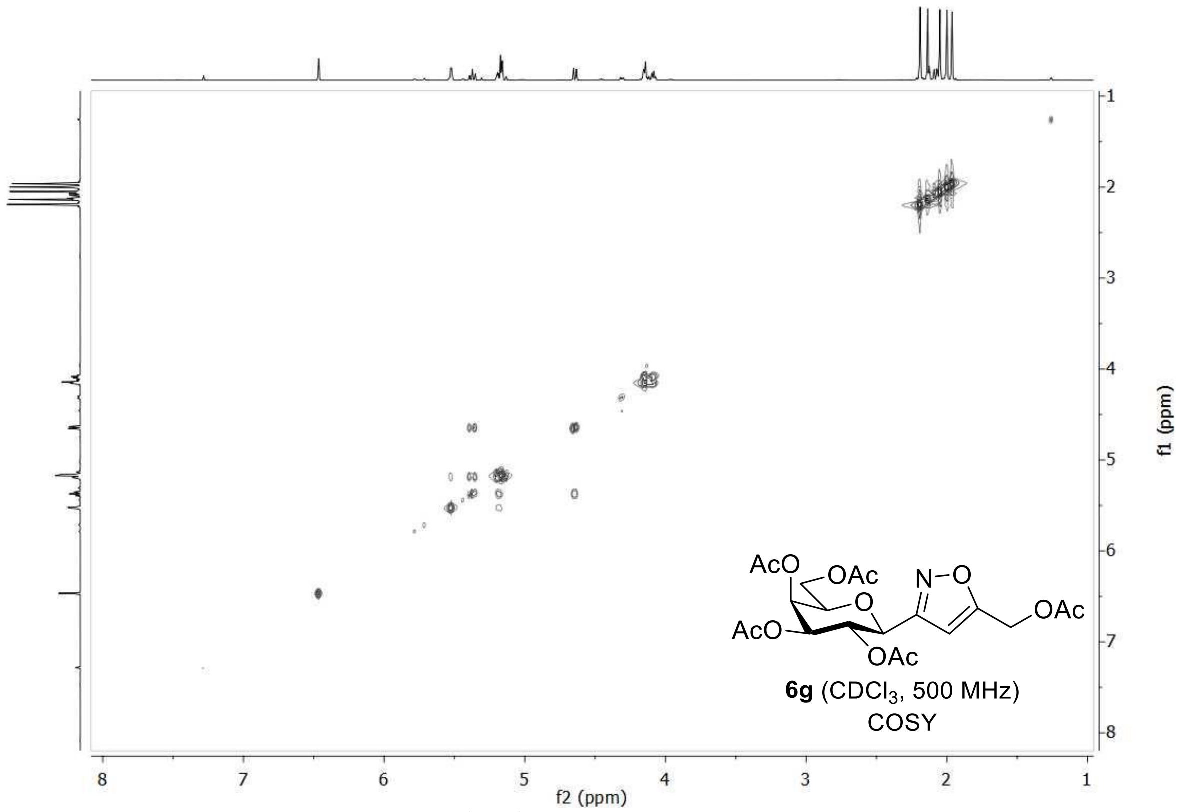

Figure S41. ^{1}H–^{1}H COSY spectrum of **6g**

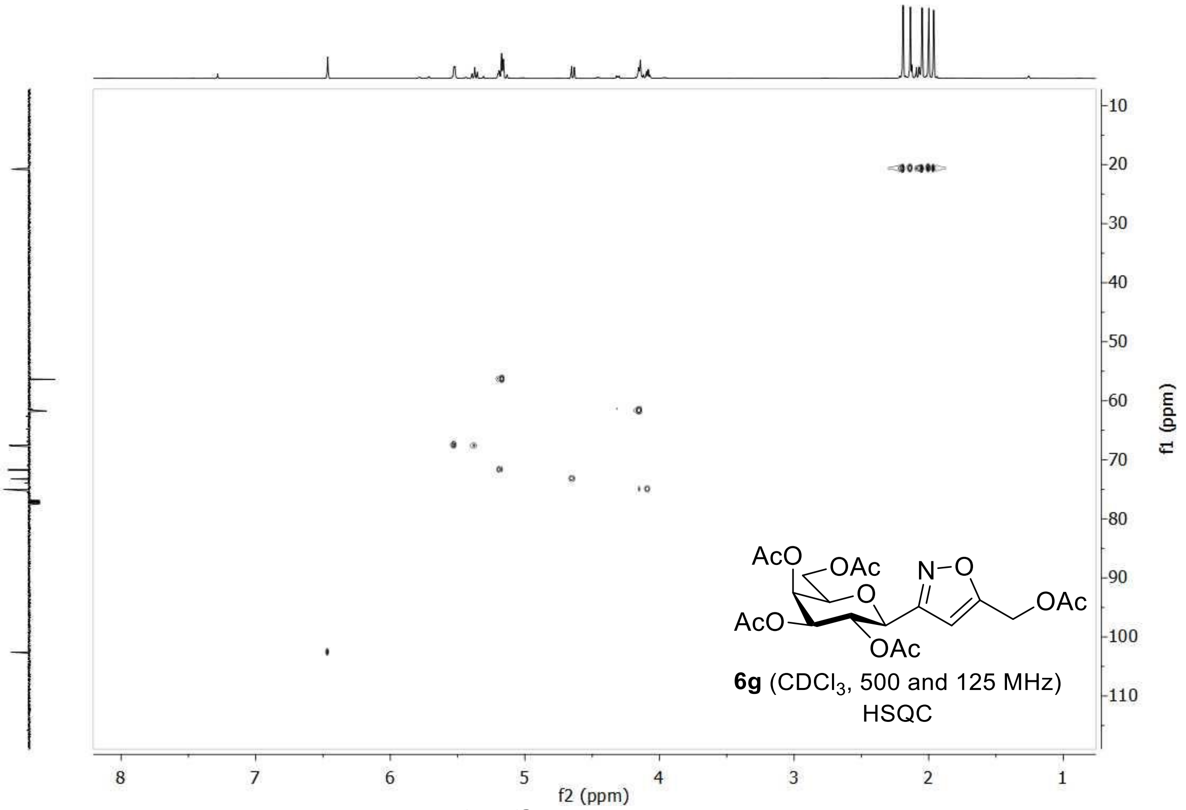

Figure S42. ^{1}H–^{13}C HSQC spectrum of **6g**

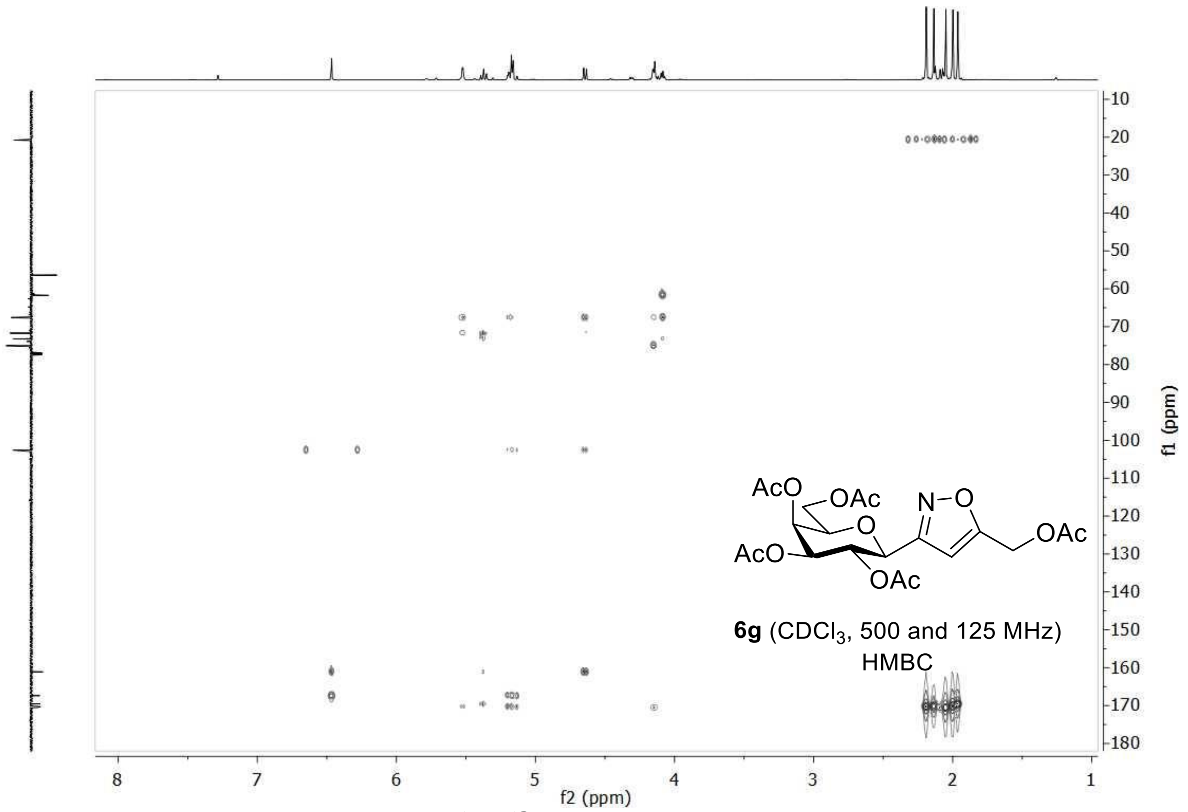

Figure S43. ^{1}H–^{13}C HMBC spectrum of **6g**

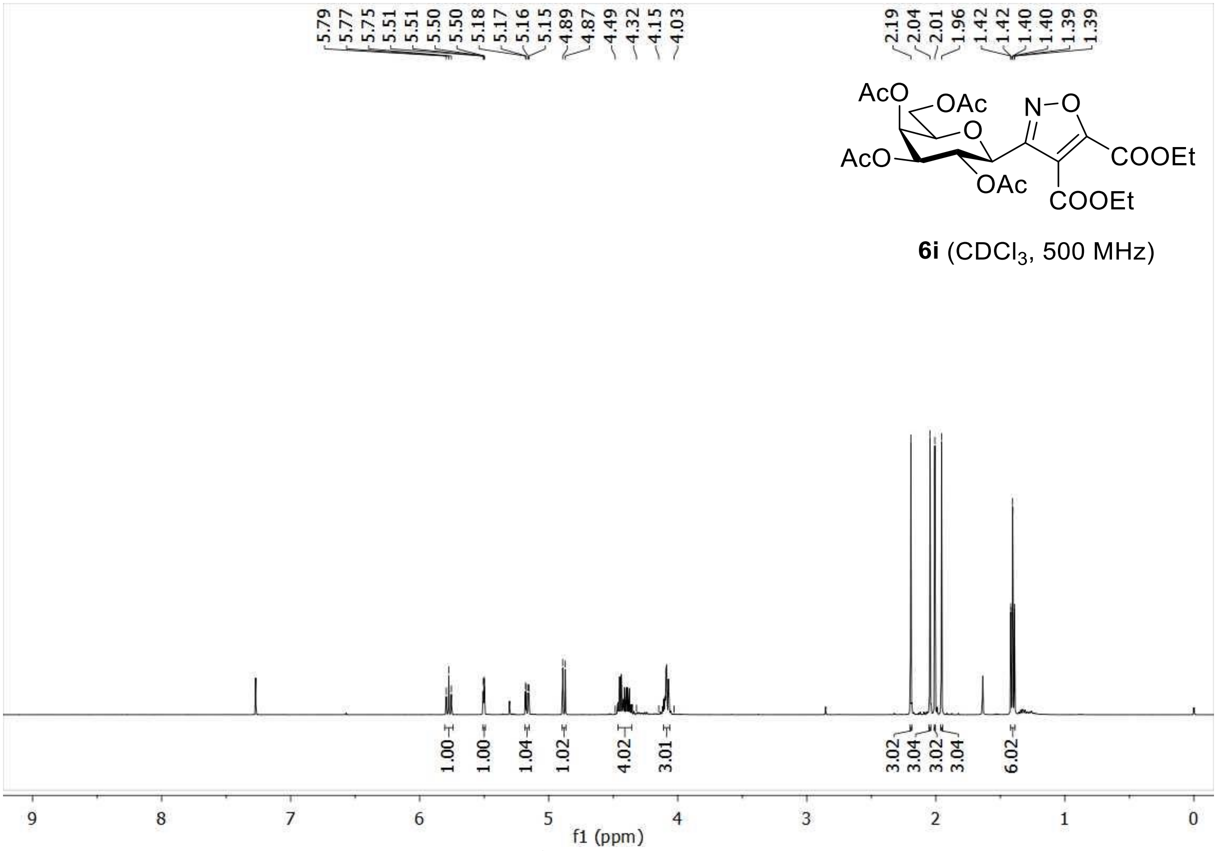

Figure S44. ^{1}H NMR spectrum of **6i**

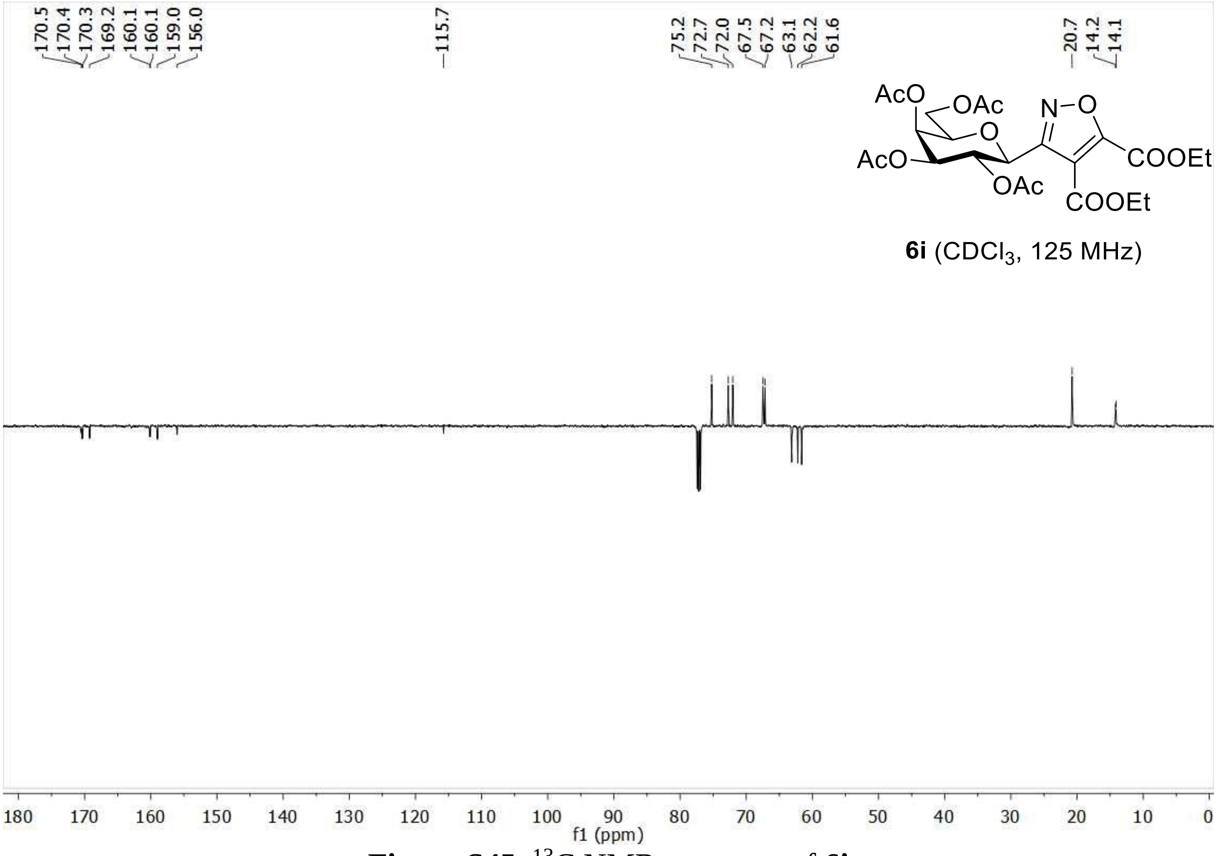

Figure S45. ^{13}C NMR spectrum of **6i**

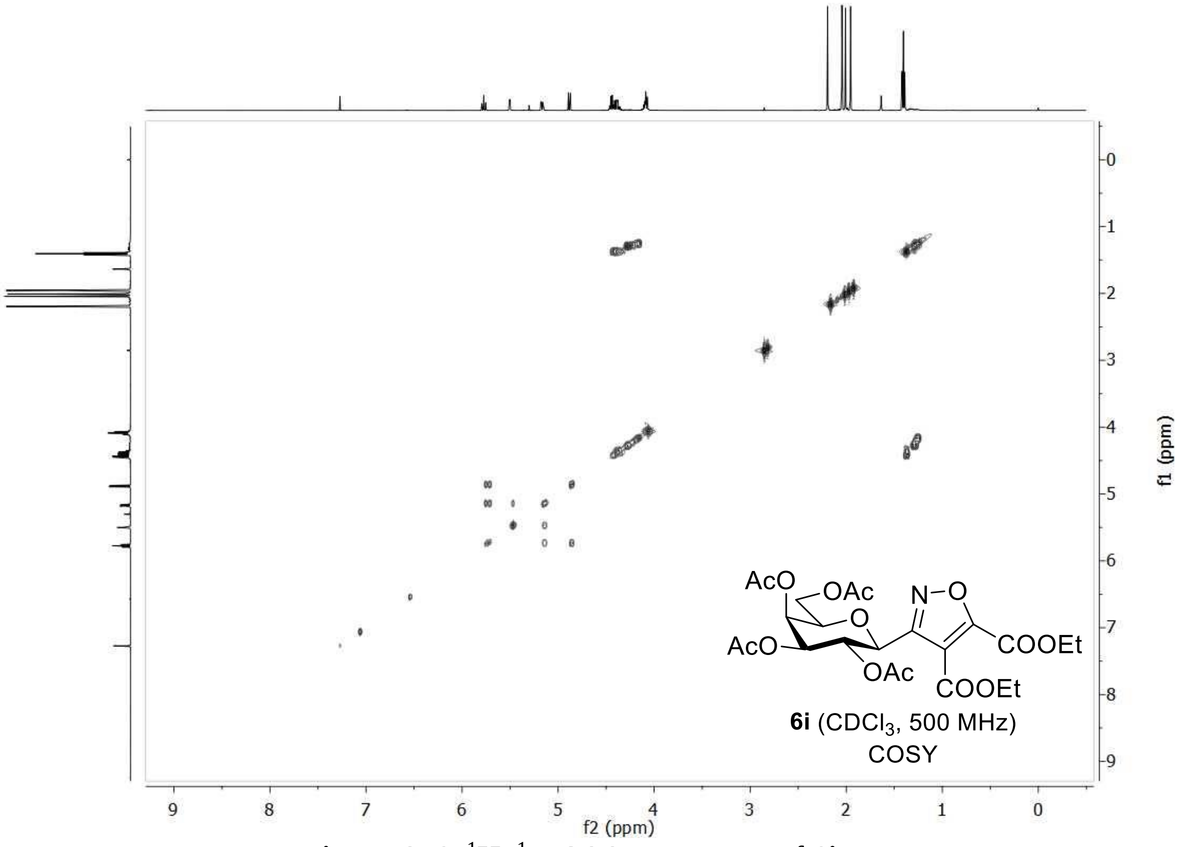

Figure S46. ^{1}H–^{1}H COSY spectrum of **6i**

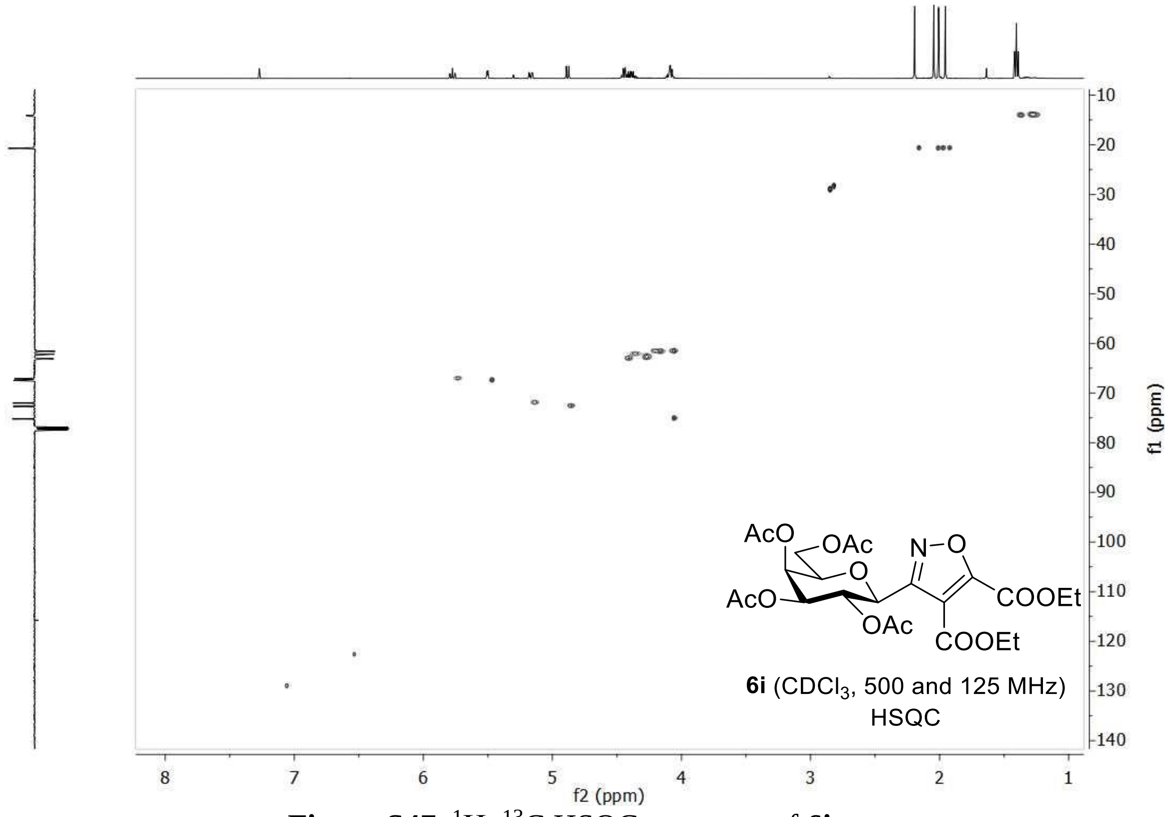

Figure S47. ^{1}H–^{13}C HSQC spectrum of **6i**

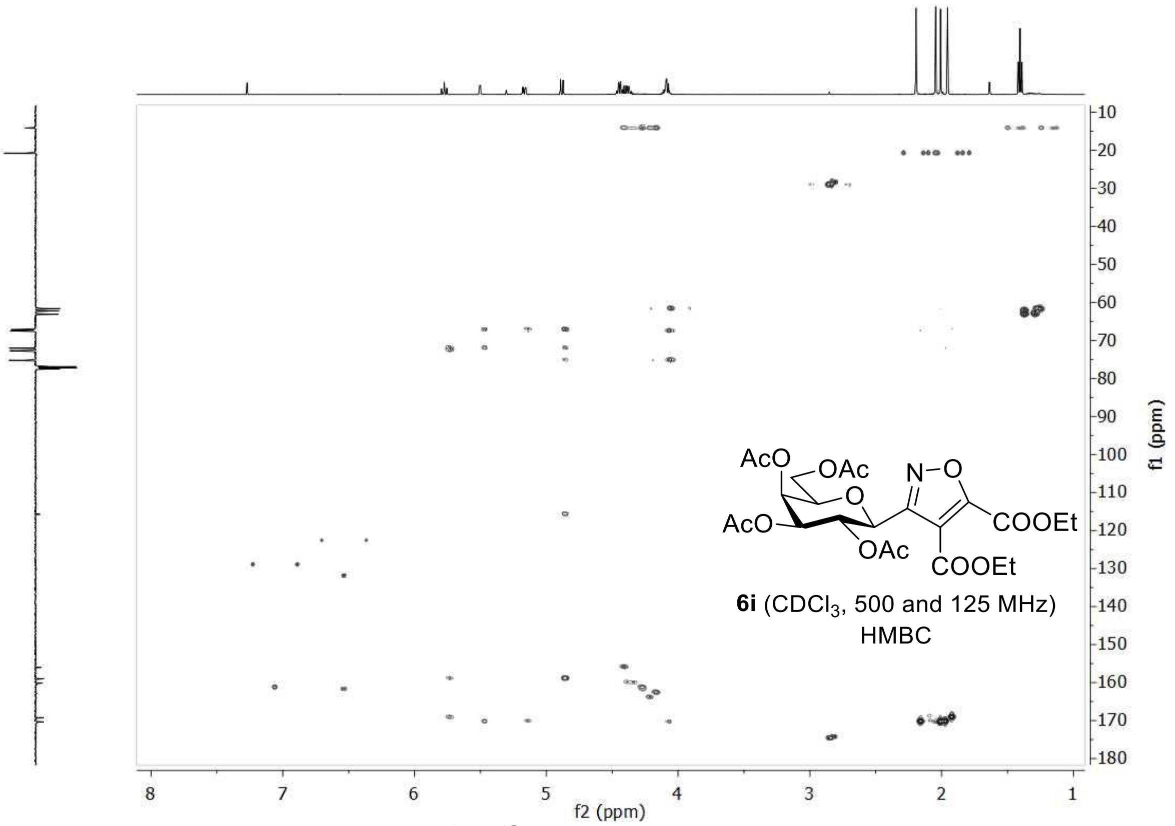

Figure S48. ^{1}H–^{13}C HMBC spectrum of **6i**

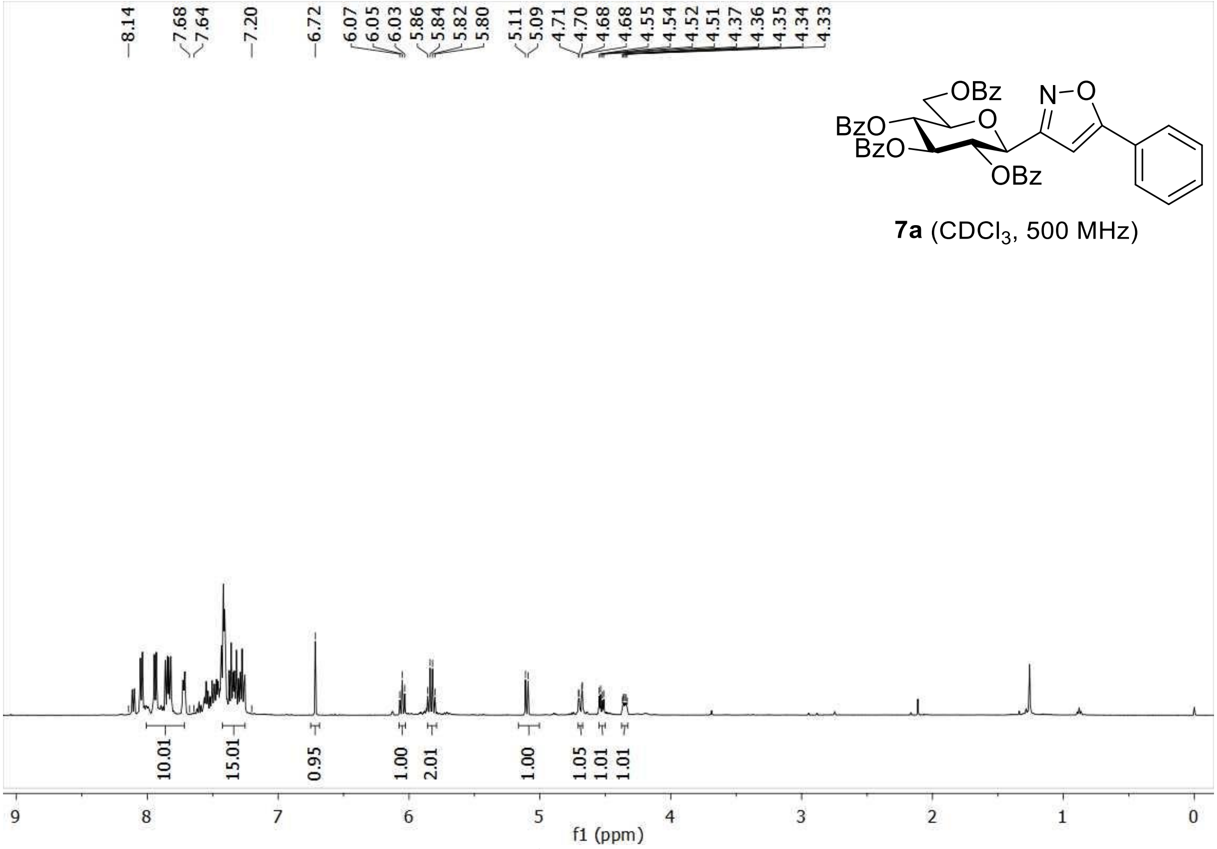

Figure S49. ^{1}H NMR spectrum of **7a**

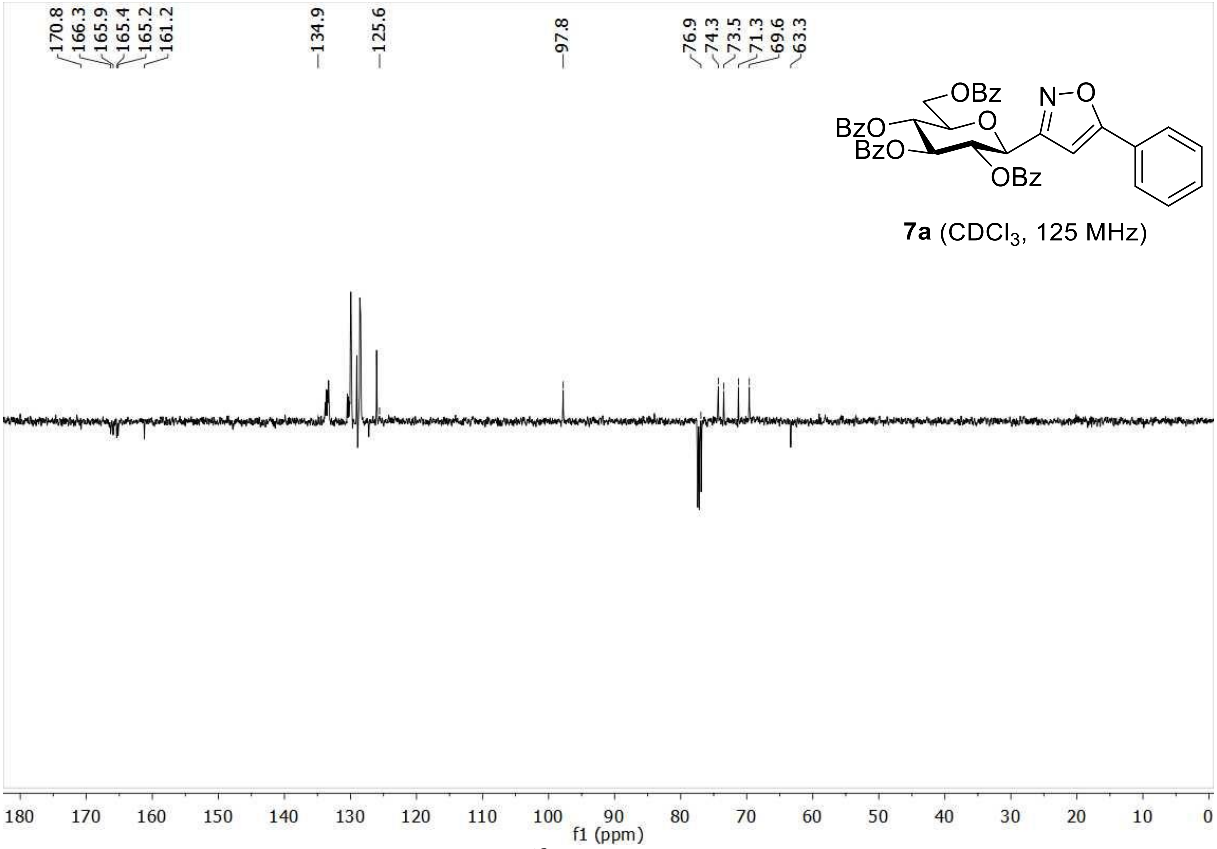

Figure S50. ^{13}C NMR spectrum of **7a**

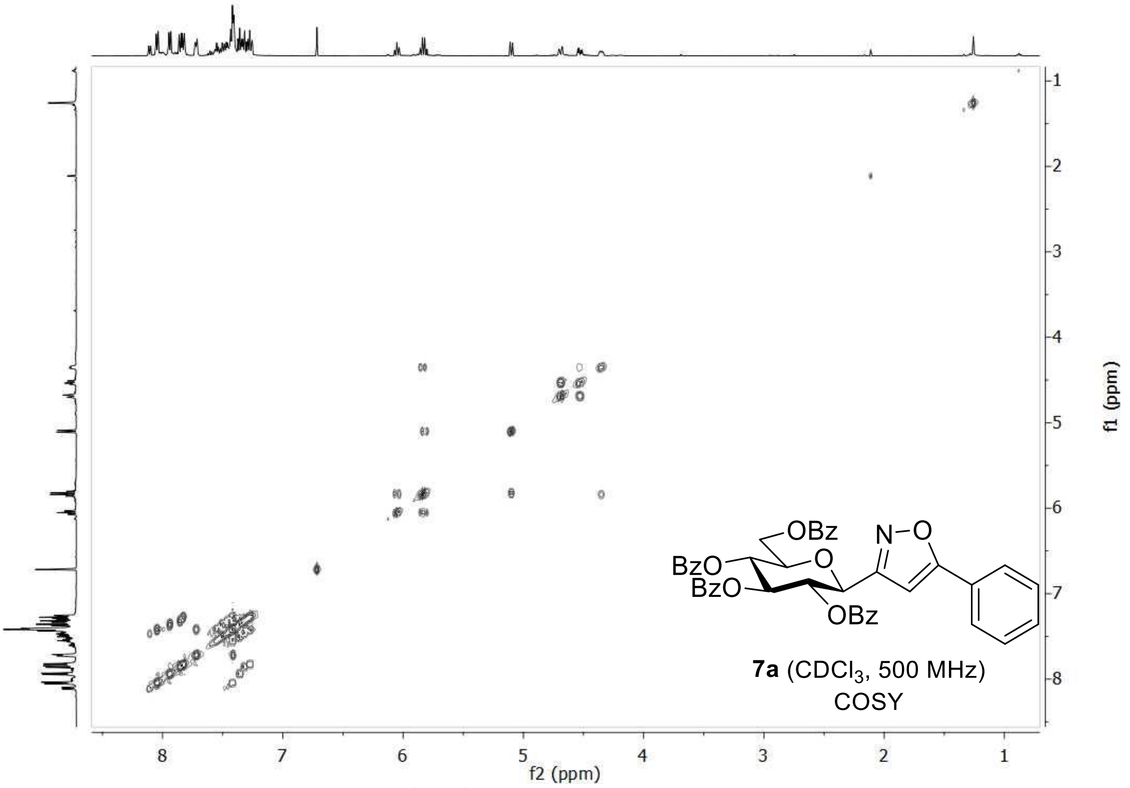

Figure S51. ^{1}H–^{1}H COSY spectrum of **7a**

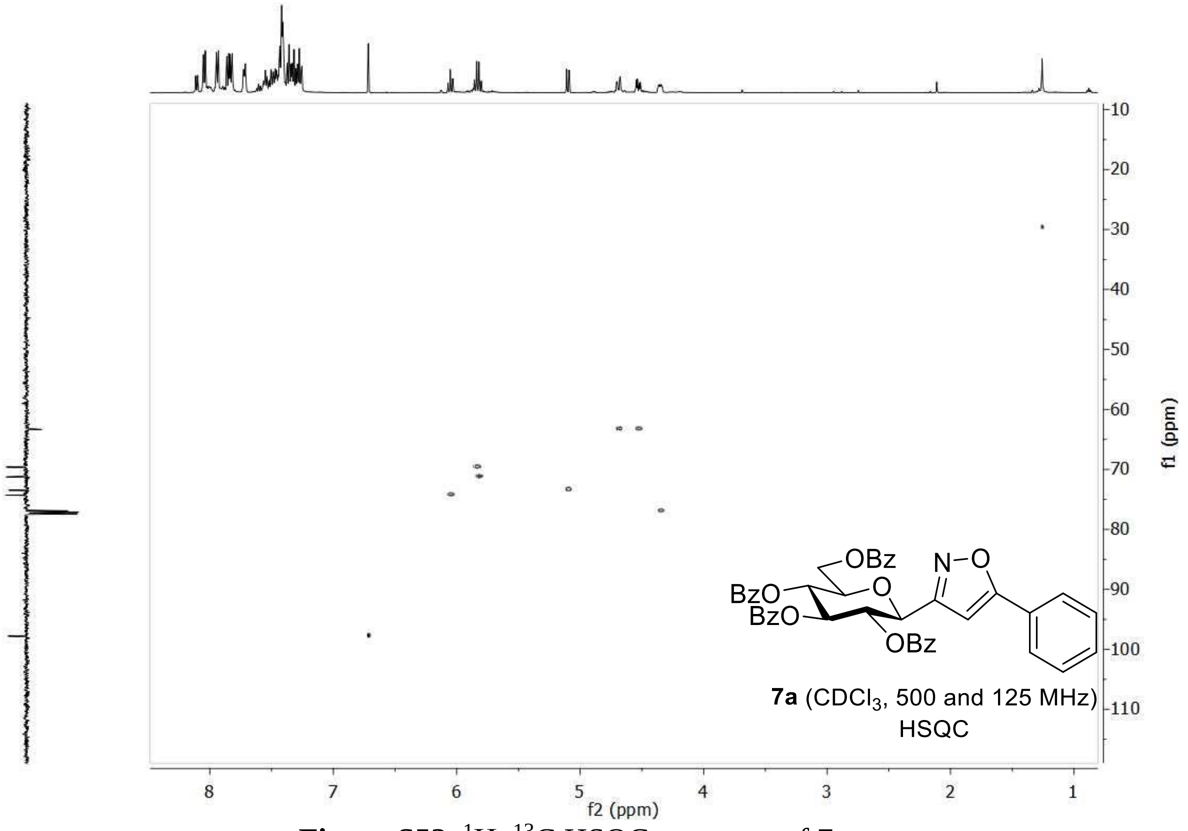

Figure S52. ^{1}H–^{13}C HSQC spectrum of **7a**

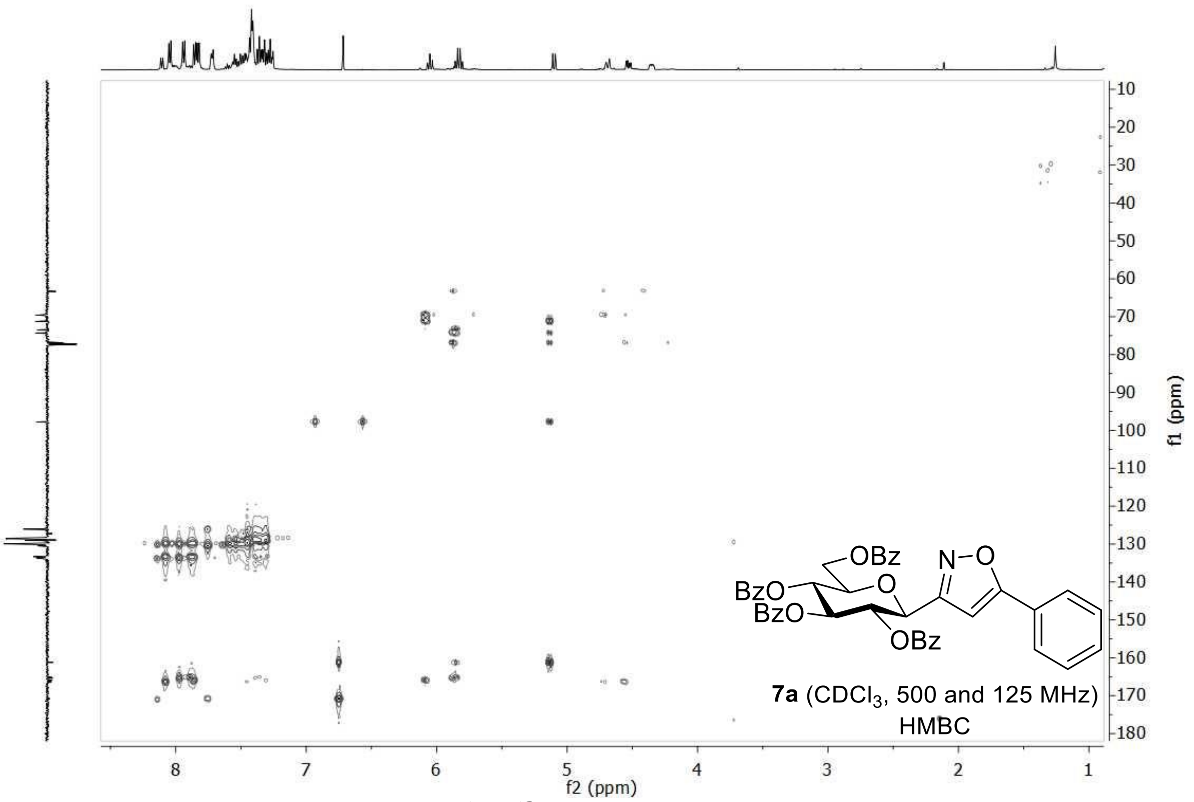

Figure S53. $^1H–^{13}C$ HMBC spectrum of **7a**

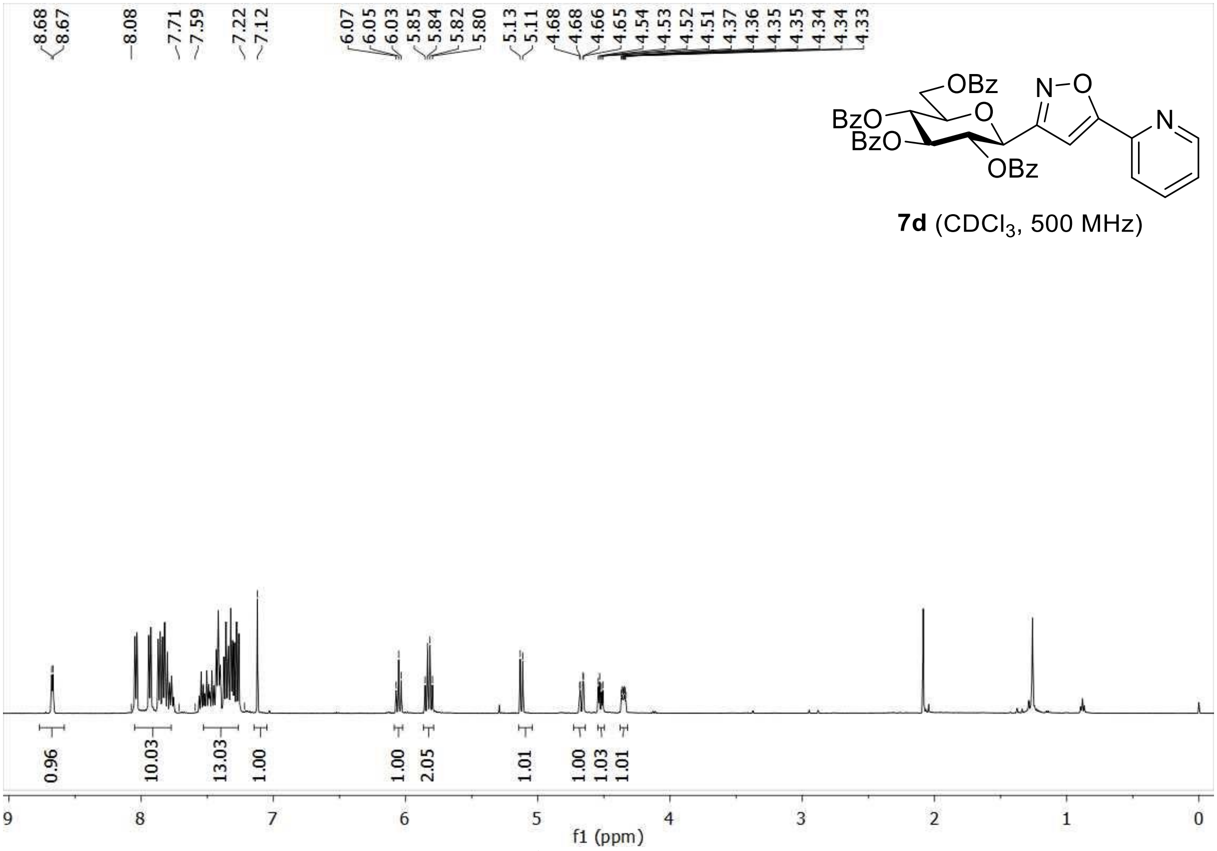

Figure S54. ^{1}H NMR spectrum of **7d**

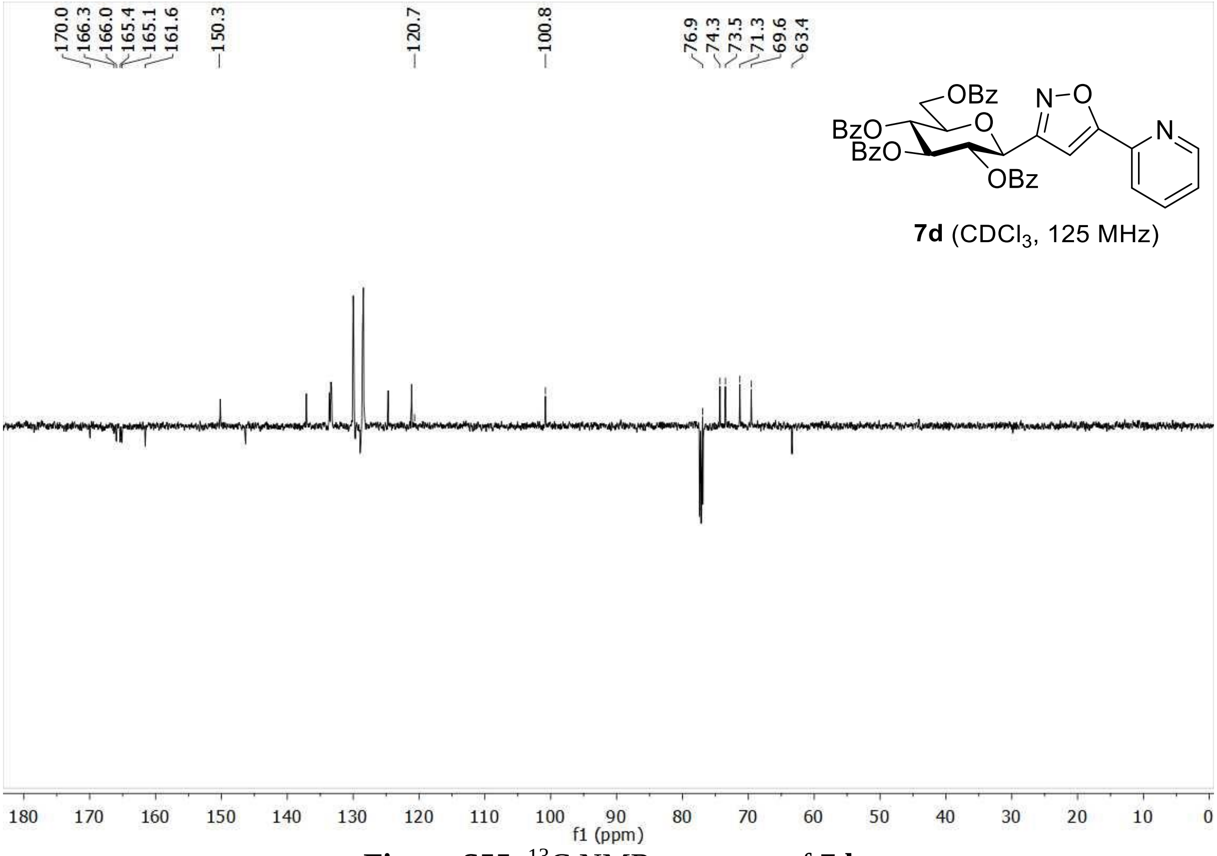

Figure S55. ^{13}C NMR spectrum of **7d**

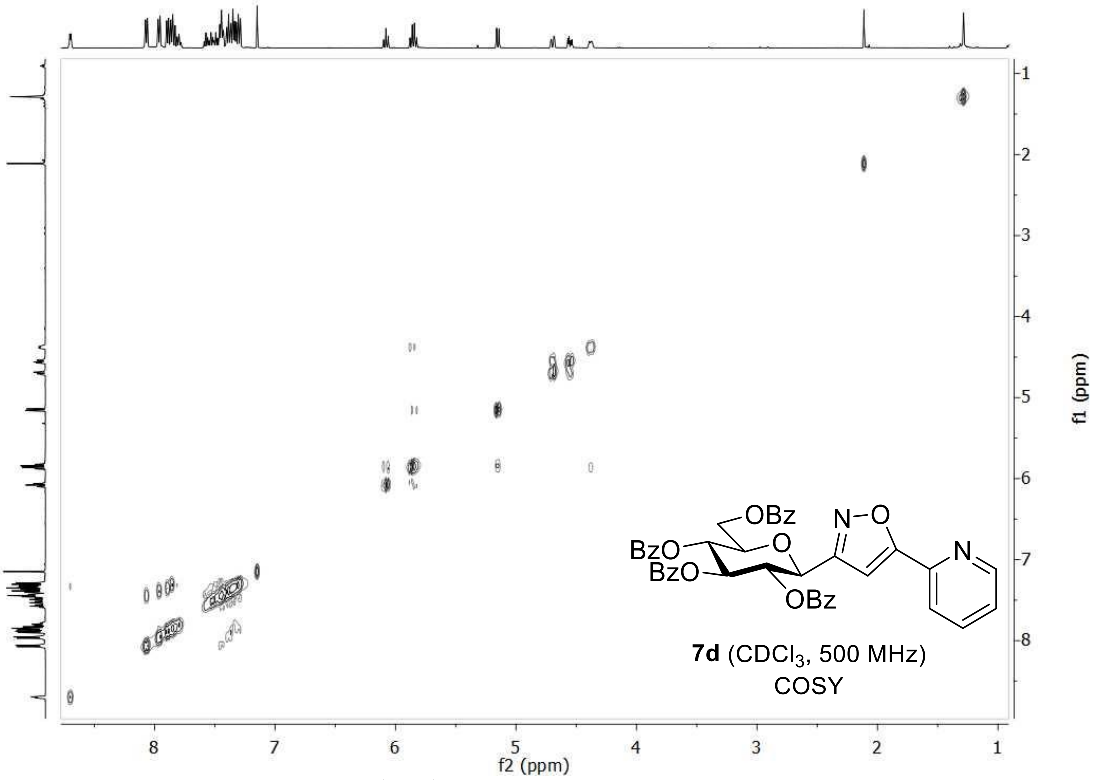

Figure S56. ^{1}H–^{1}H COSY spectrum of **7d**

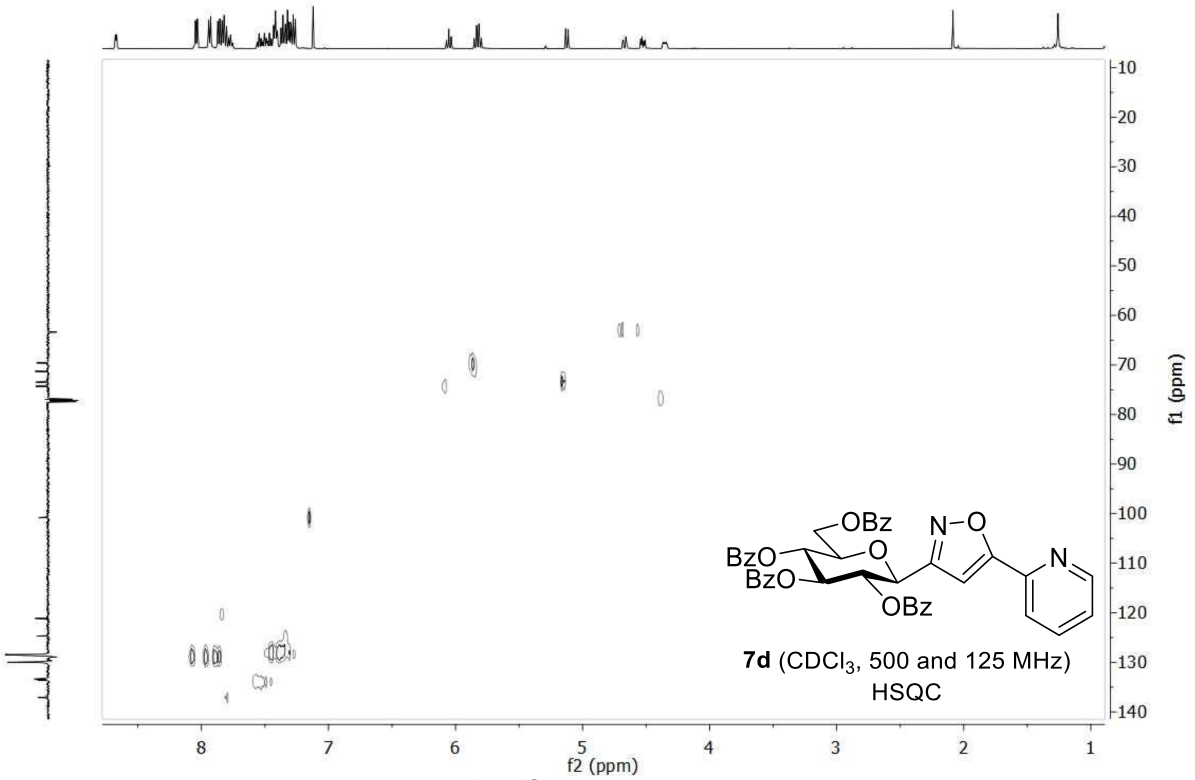

Figure S57. ^{1}H–^{13}C HSQC spectrum of **7d**

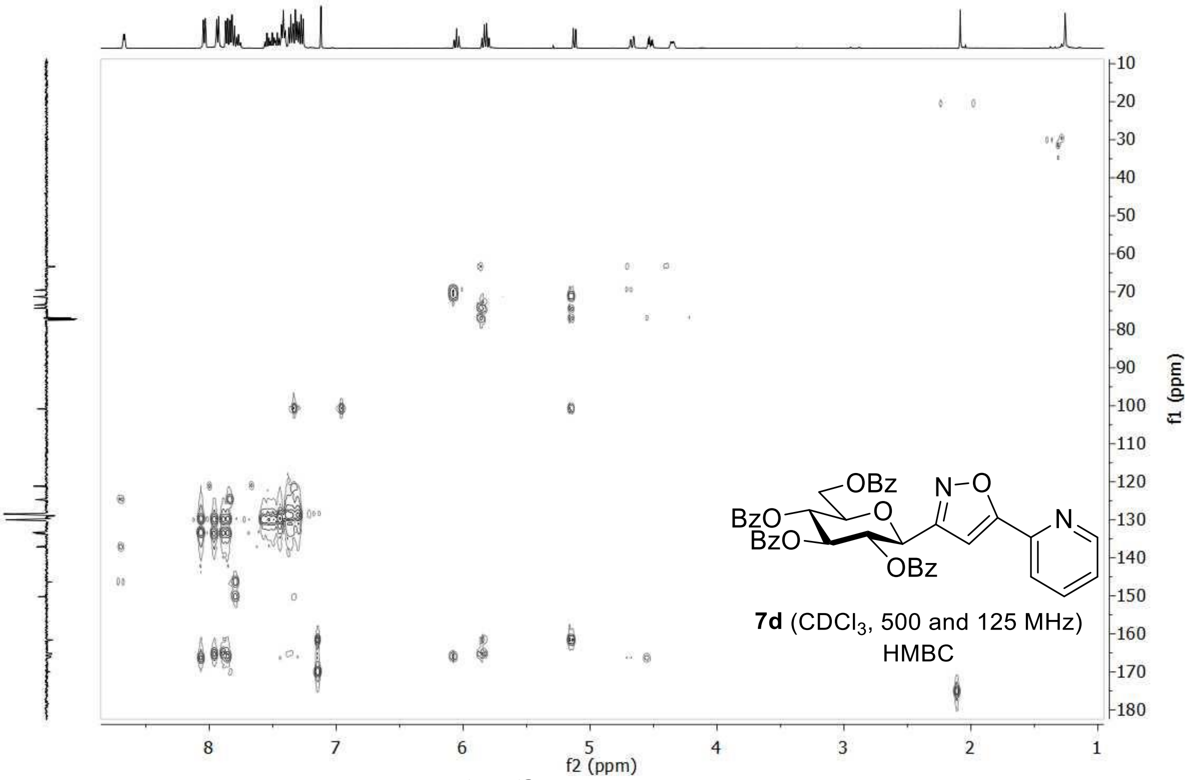

Figure S58. ^{1}H–^{13}C HMBC spectrum of **7d**

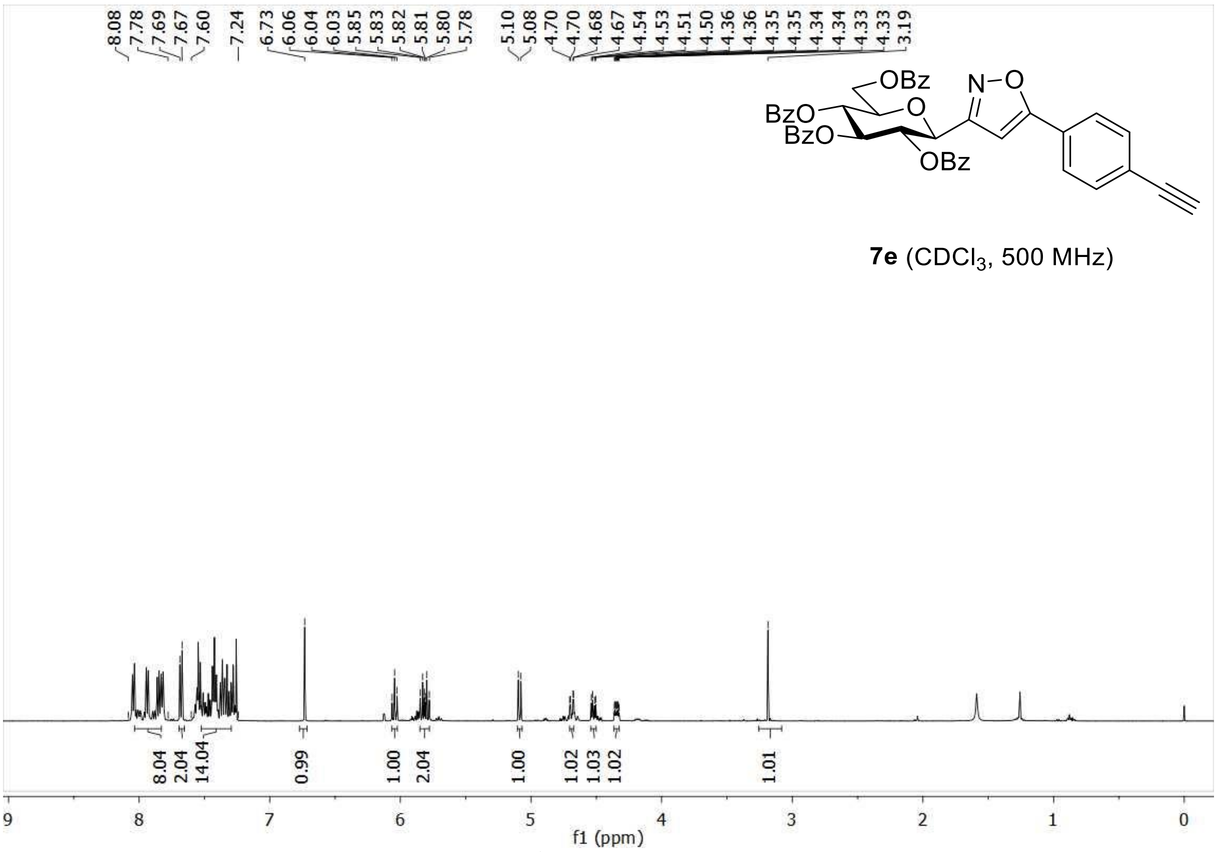

Figure S59. ^{1}H NMR spectrum of **7e**

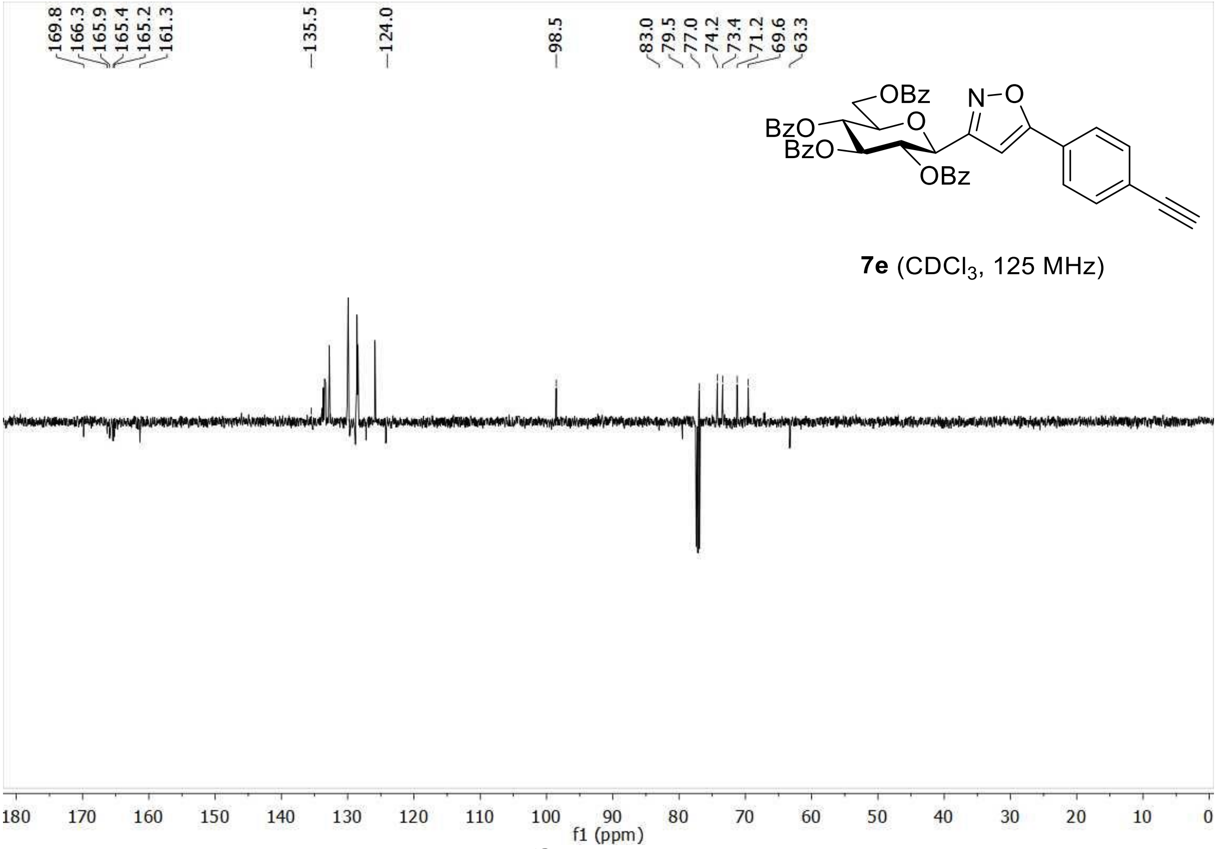

Figure S60. ^{13}C NMR spectrum of **7e**

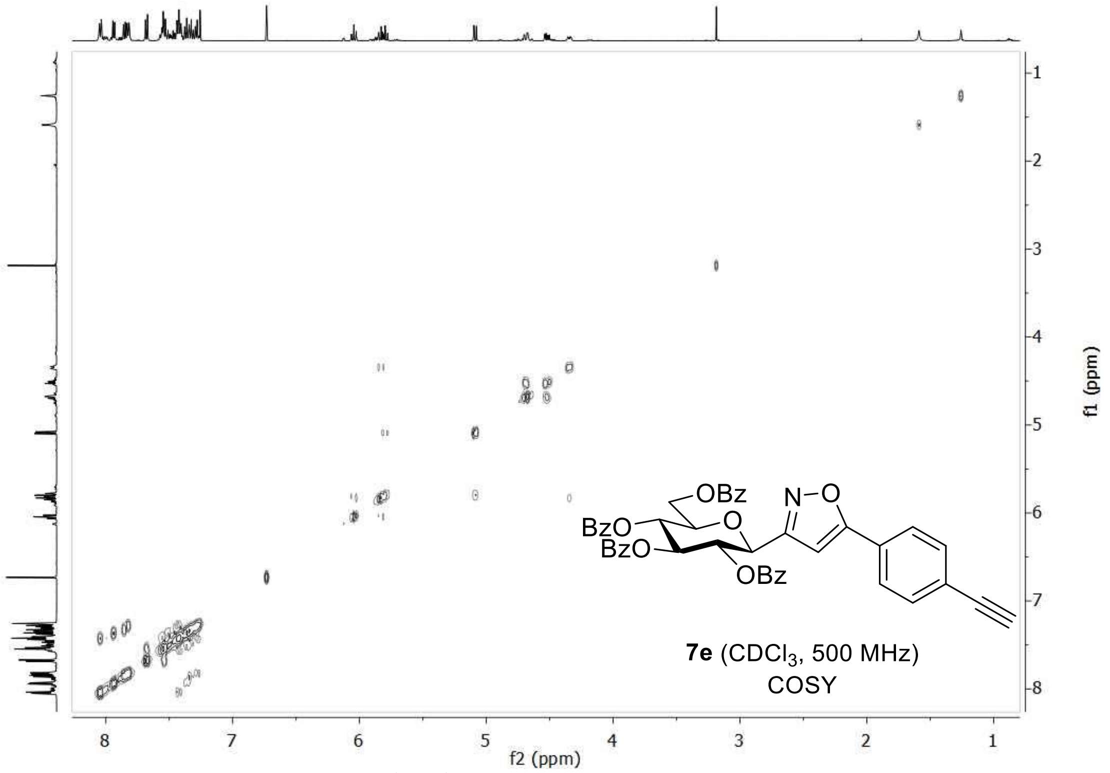

Figure S61. ^{1}H–^{1}H COSY spectrum of **7e**

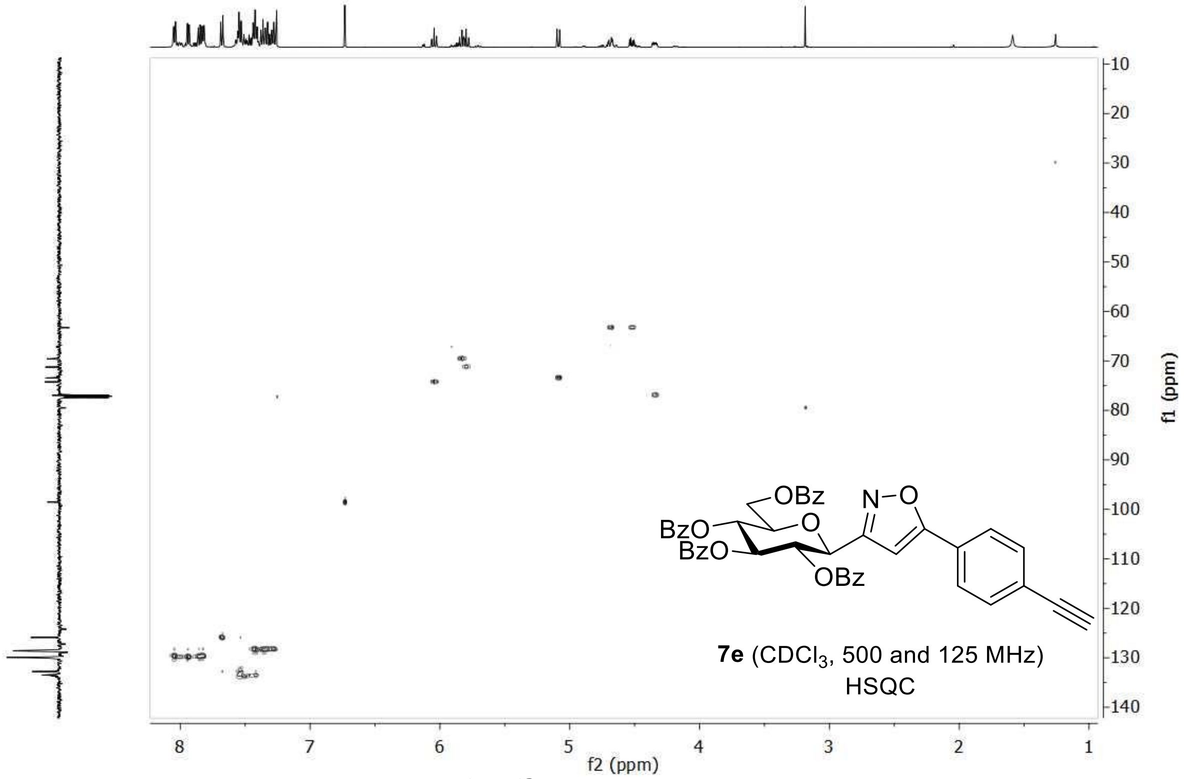

Figure S62. ^{1}H–^{13}C HSQC spectrum of **7e**

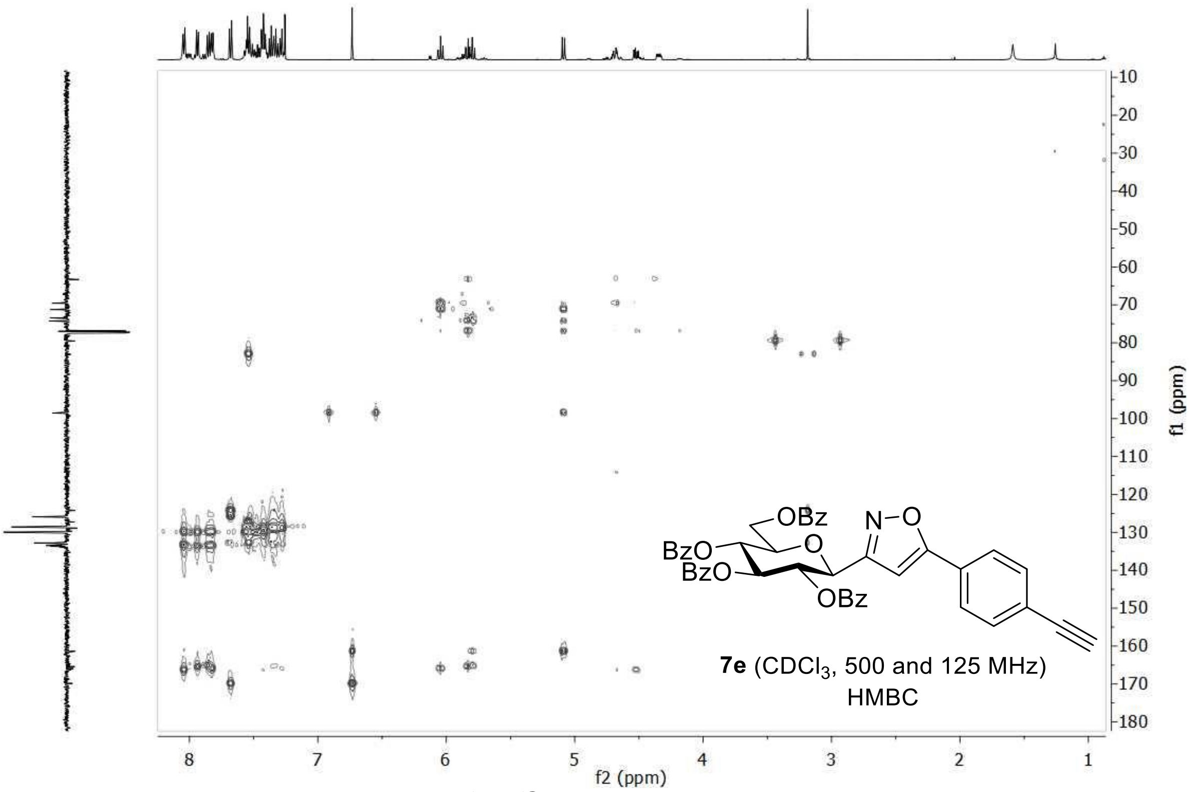

Figure S63. ^{1}H–^{13}C HMBC spectrum of **7e**

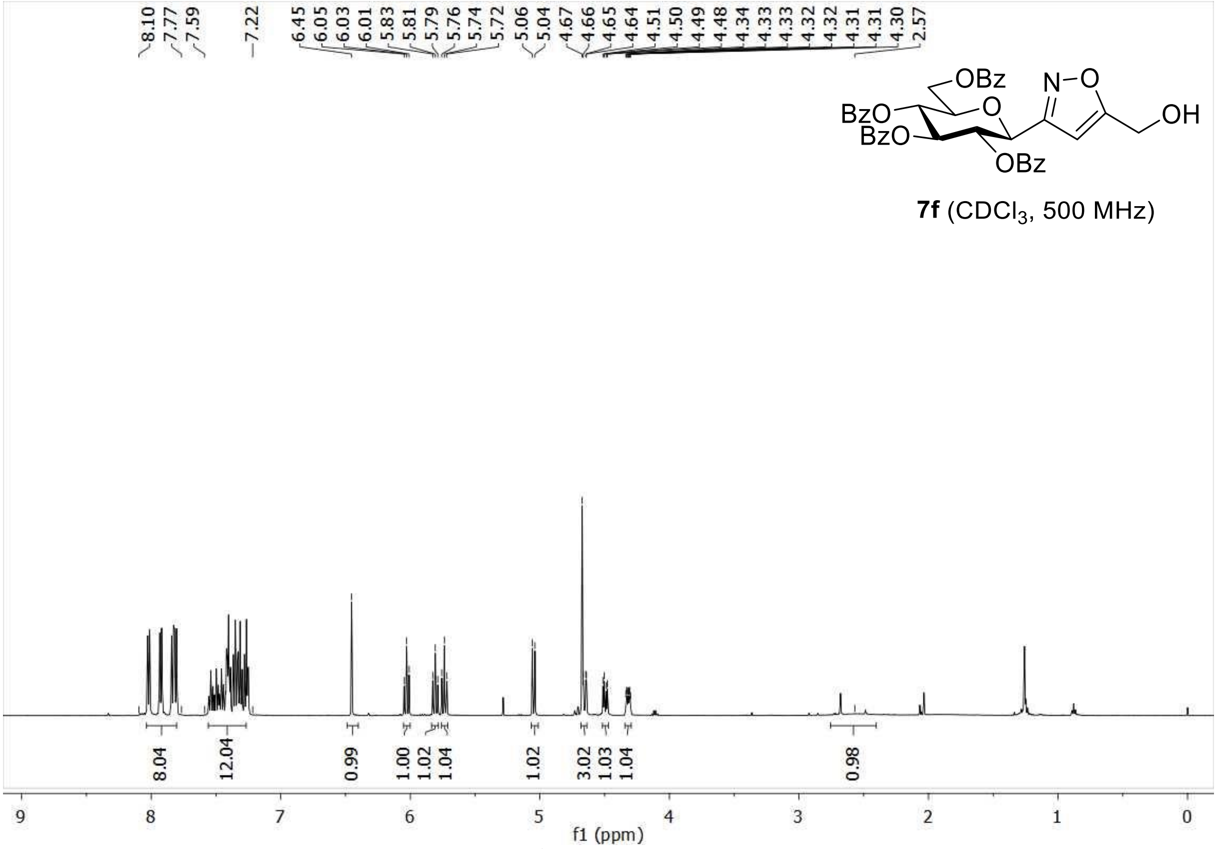

Figure S64. ^{1}H NMR spectrum of **7f**

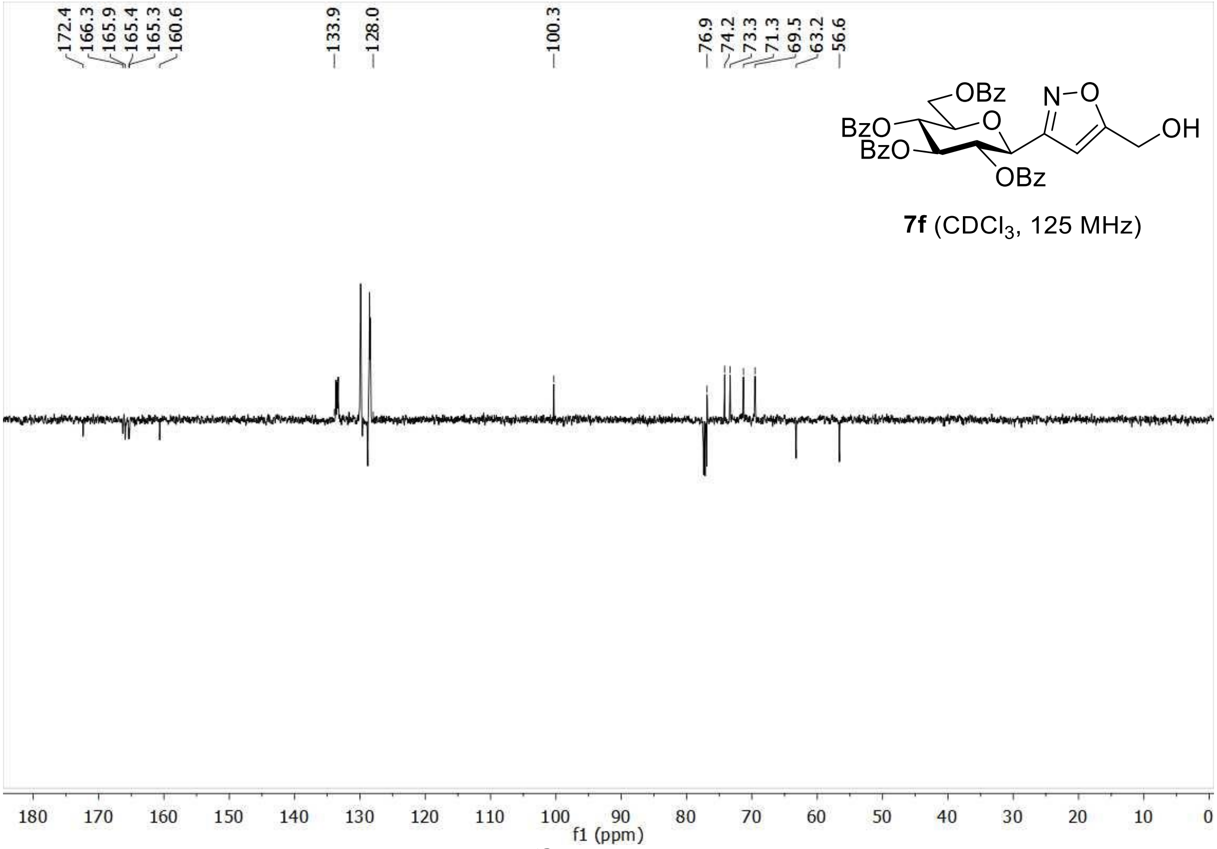

Figure S65. ^{13}C NMR spectrum of **7f**

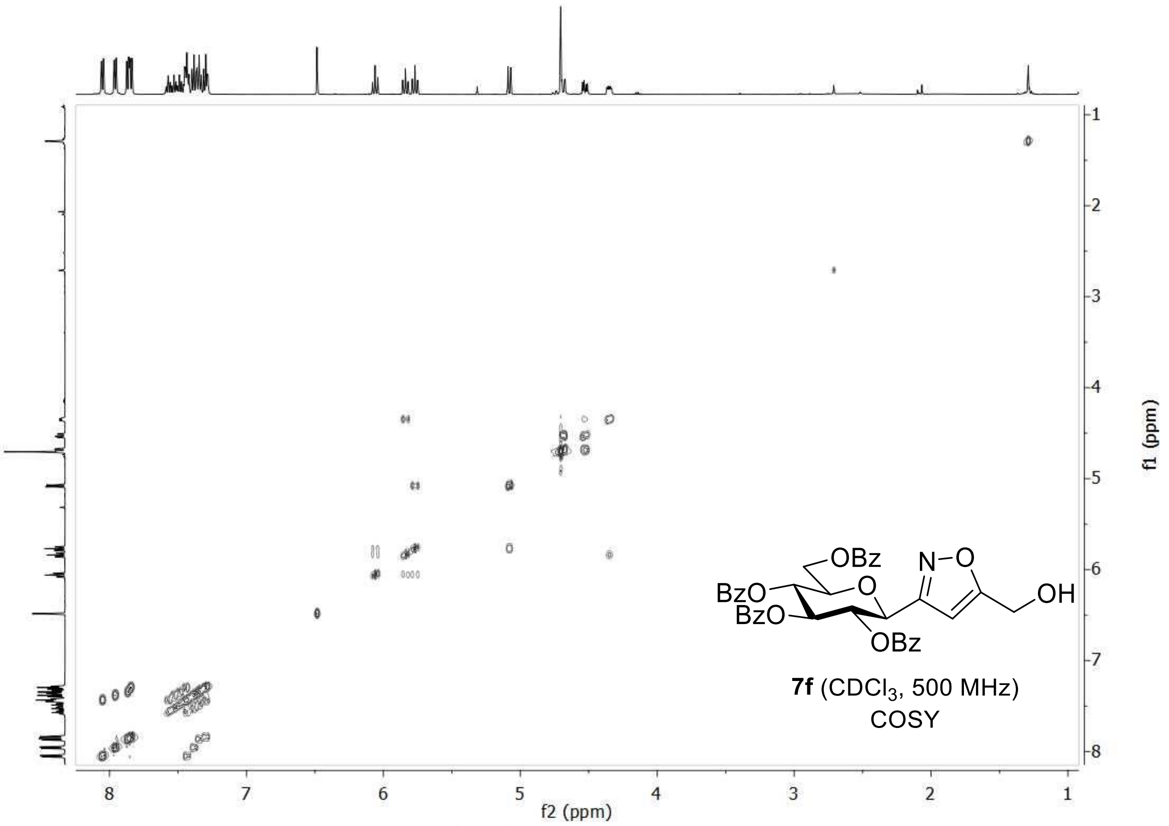

Figure S66. ^{1}H–^{1}H COSY spectrum of **7f**

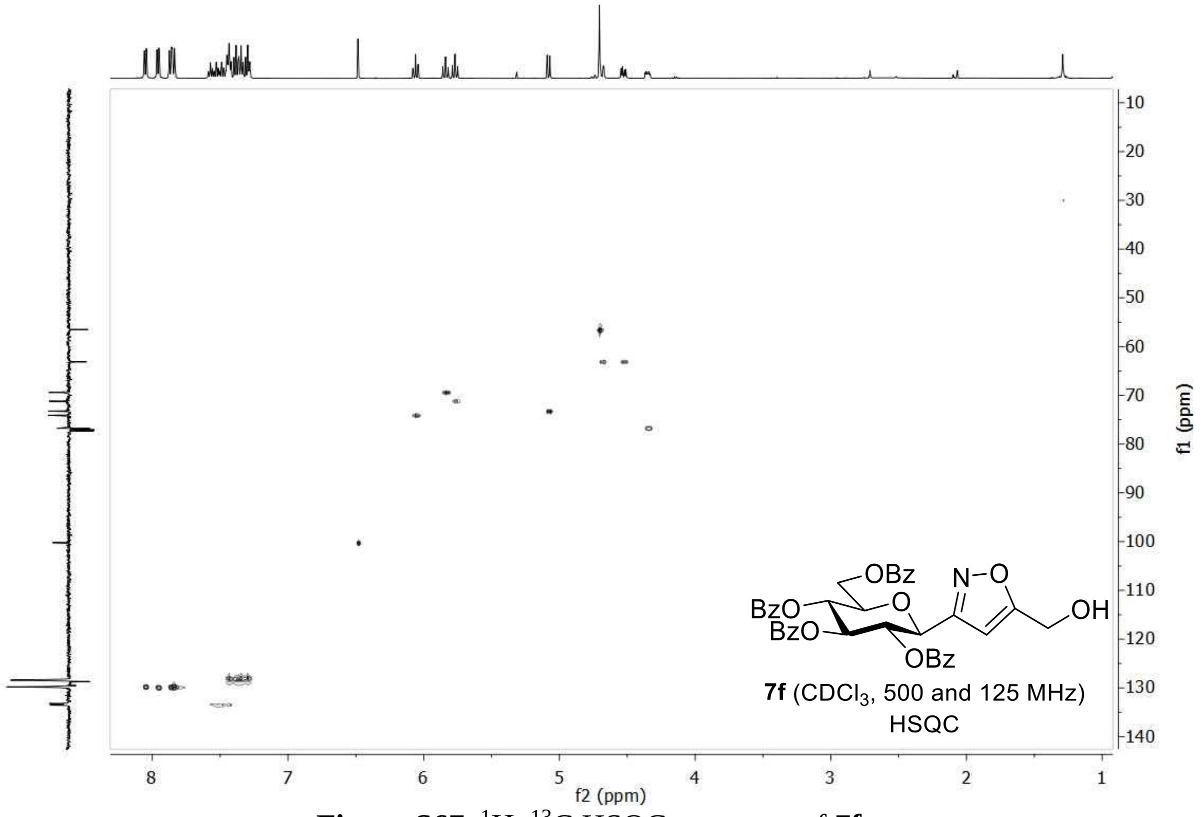

Figure S67. ^{1}H–^{13}C HSQC spectrum of **7f**

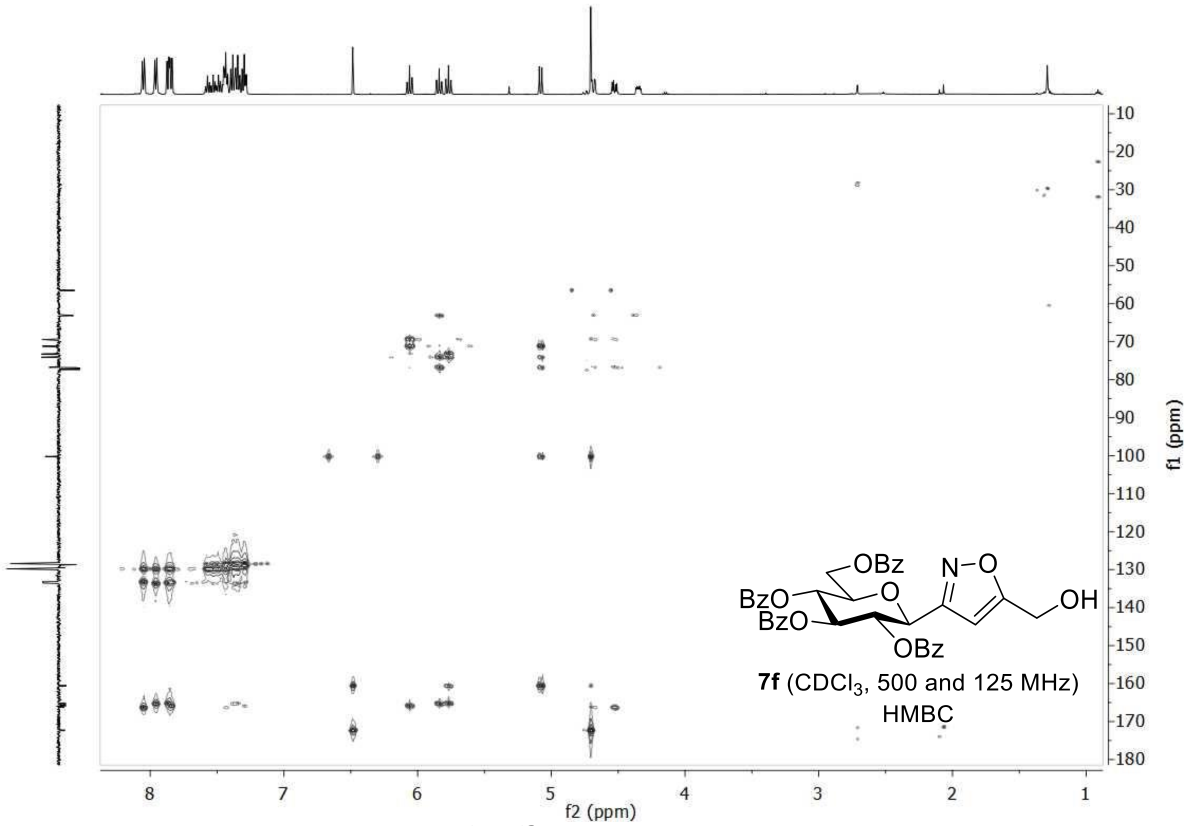

Figure S68. ^{1}H–^{13}C HMBC spectrum of **7f**

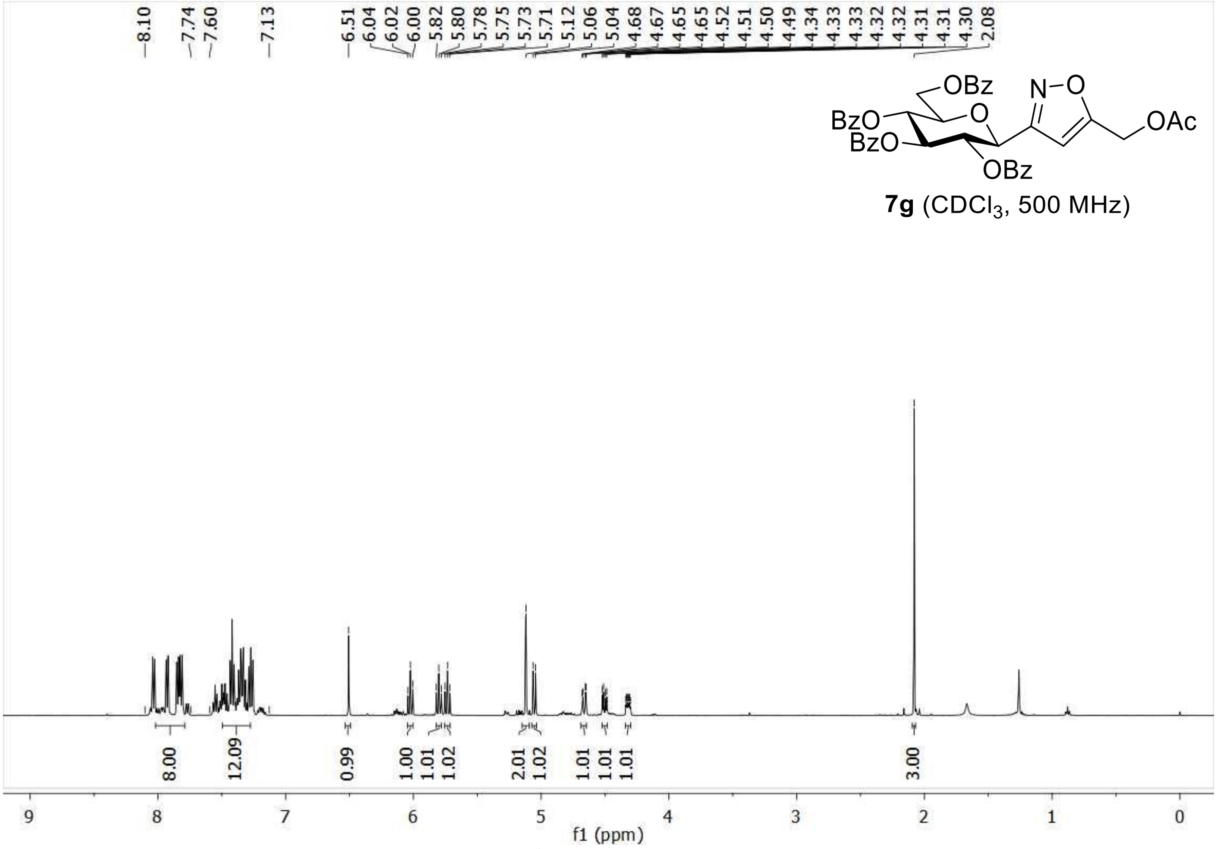

Figure S69. ^{1}H NMR spectrum of **7g**

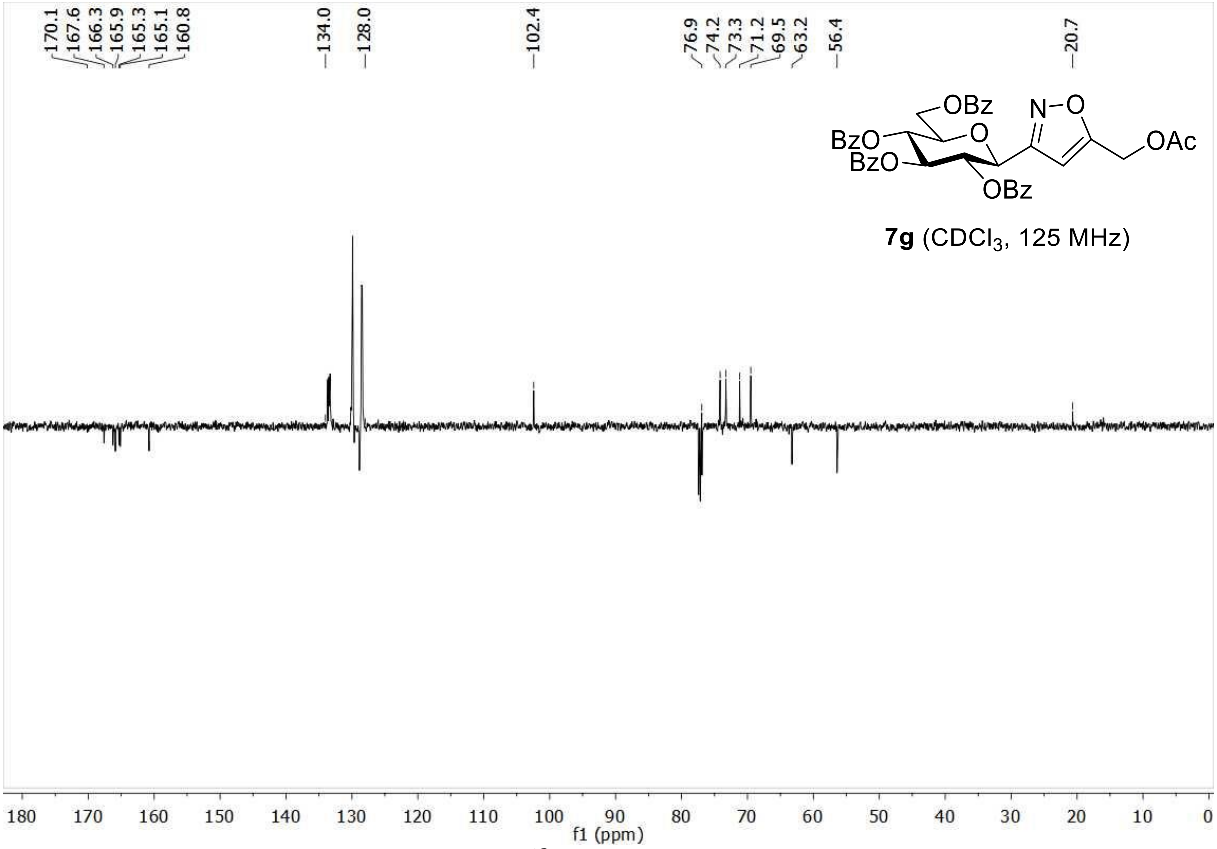

Figure S70. ^{13}C NMR spectrum of **7g**

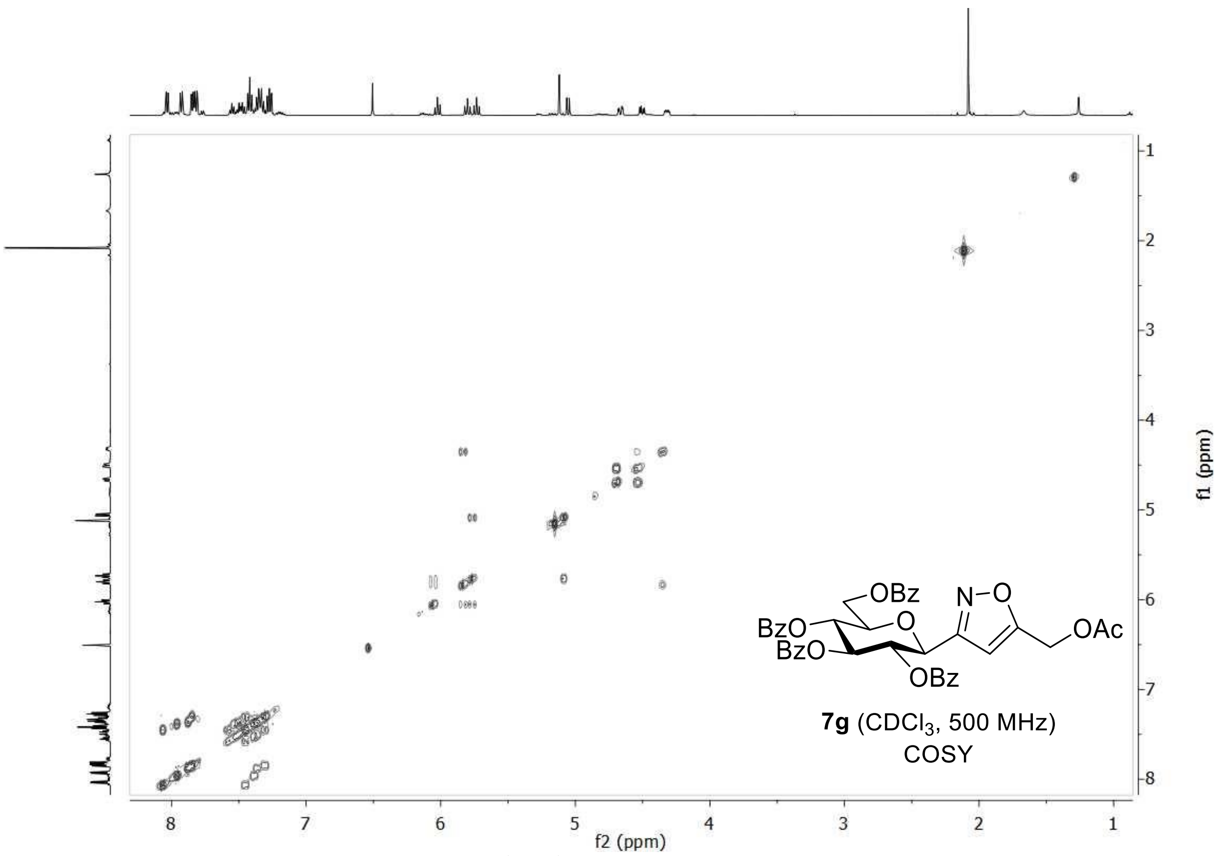

Figure S71. ¹H–¹H COSY spectrum of **7g**

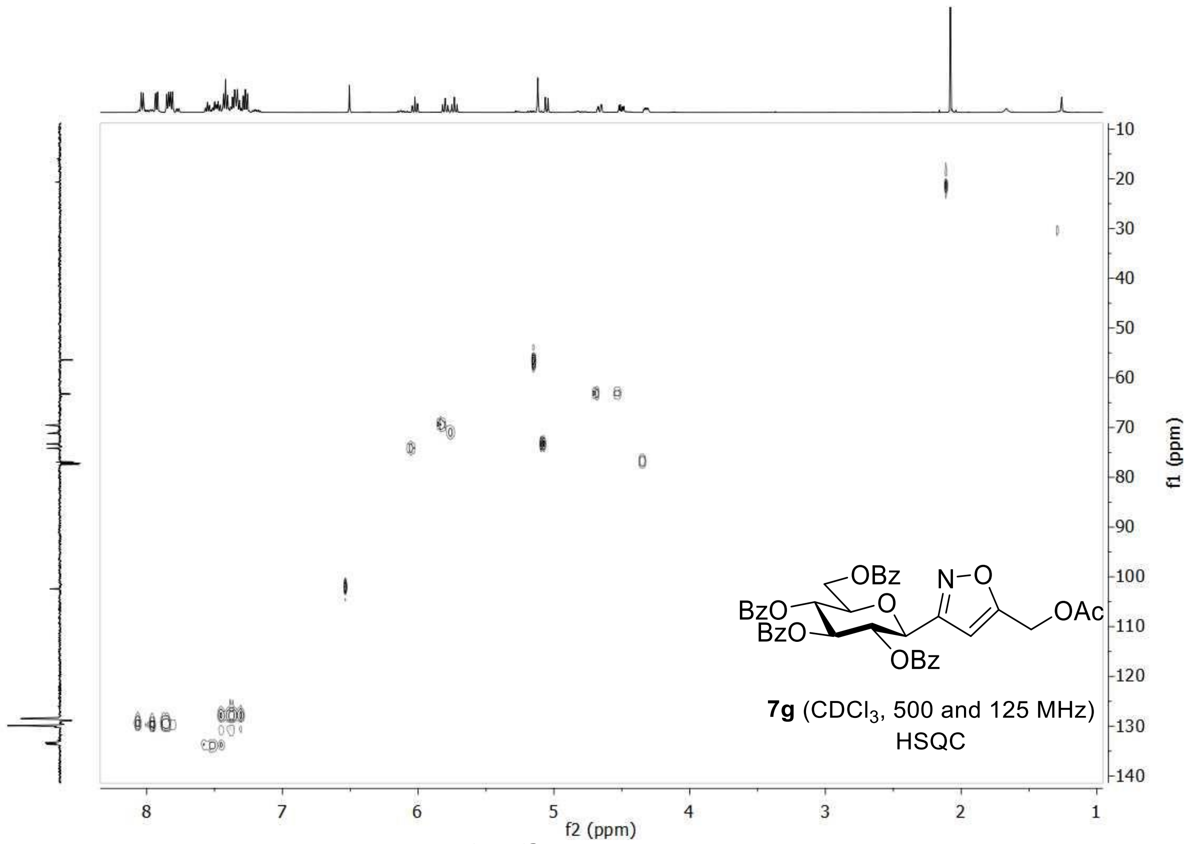

Figure S72. ¹H–¹³C HSQC spectrum of **7g**

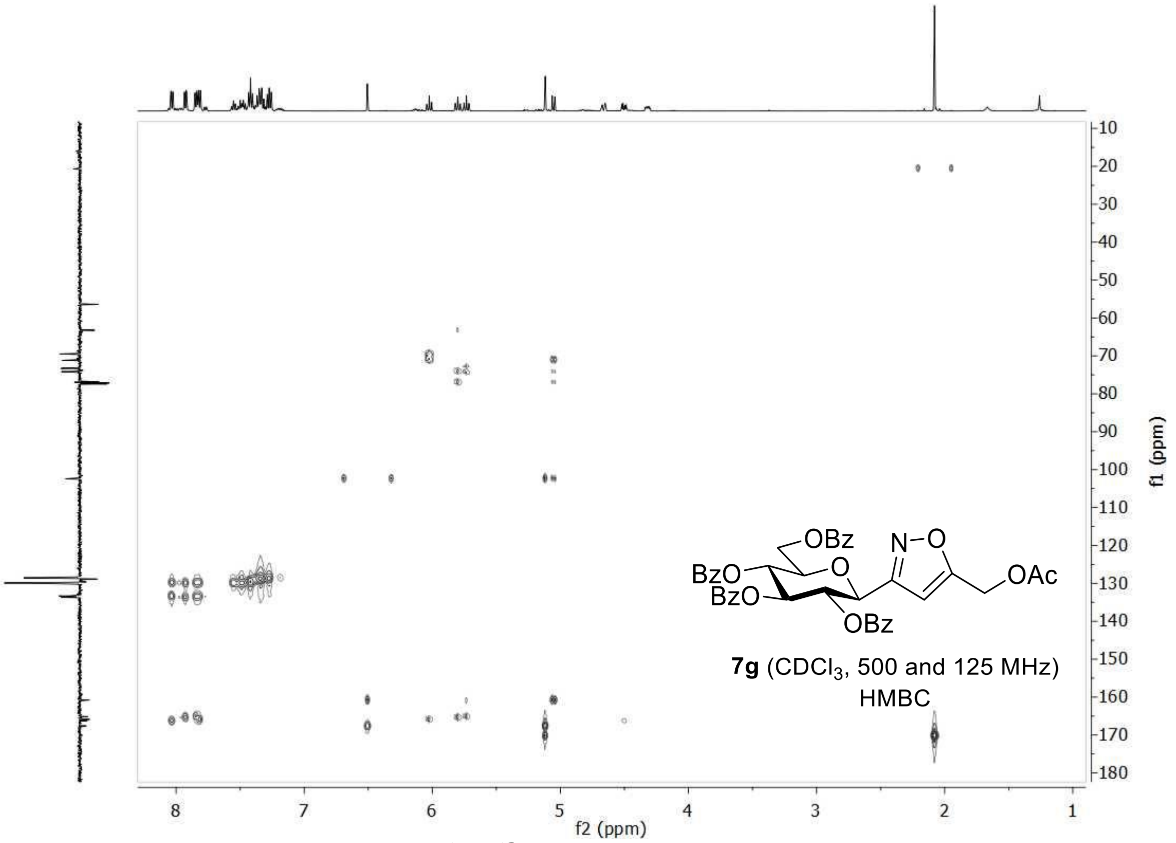

Figure S73. ^{1}H–^{13}C HMBC spectrum of **7g**

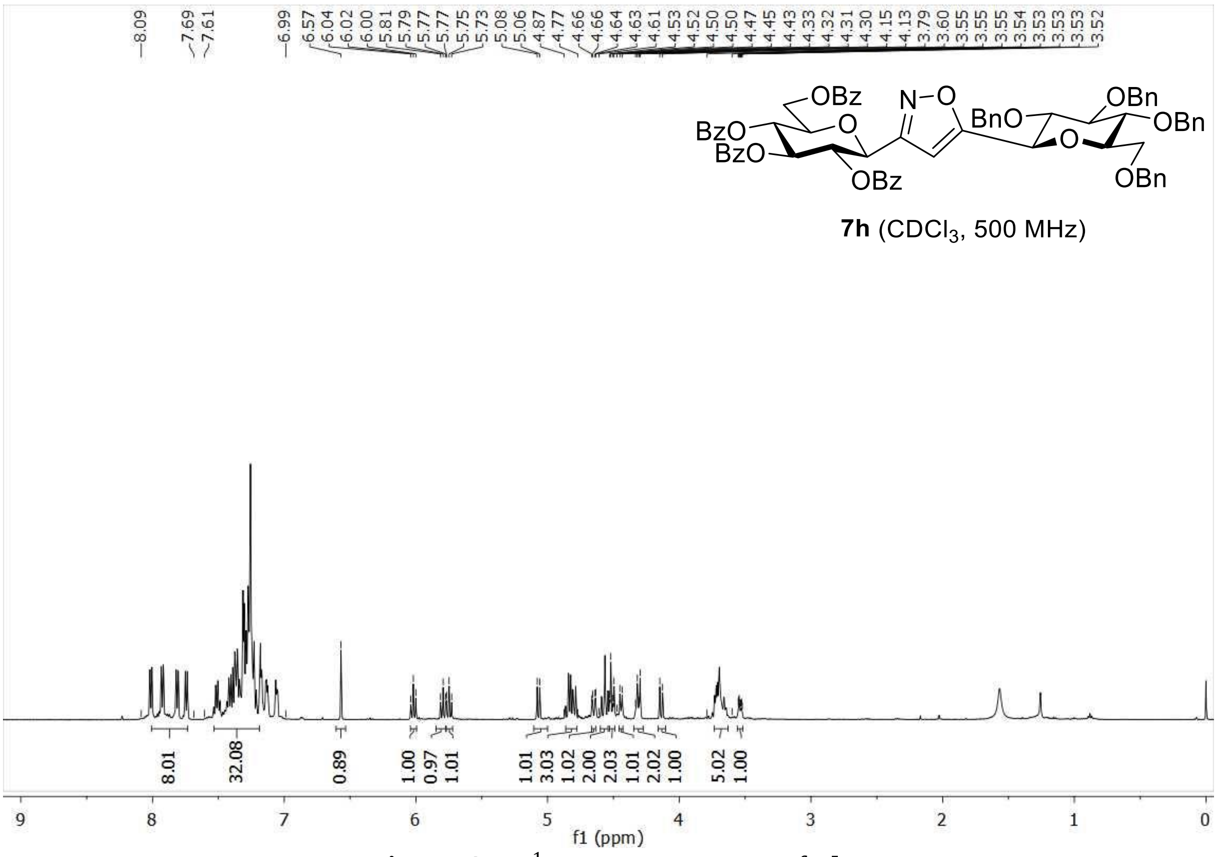

Figure S74. ^{1}H NMR spectrum of **7h**

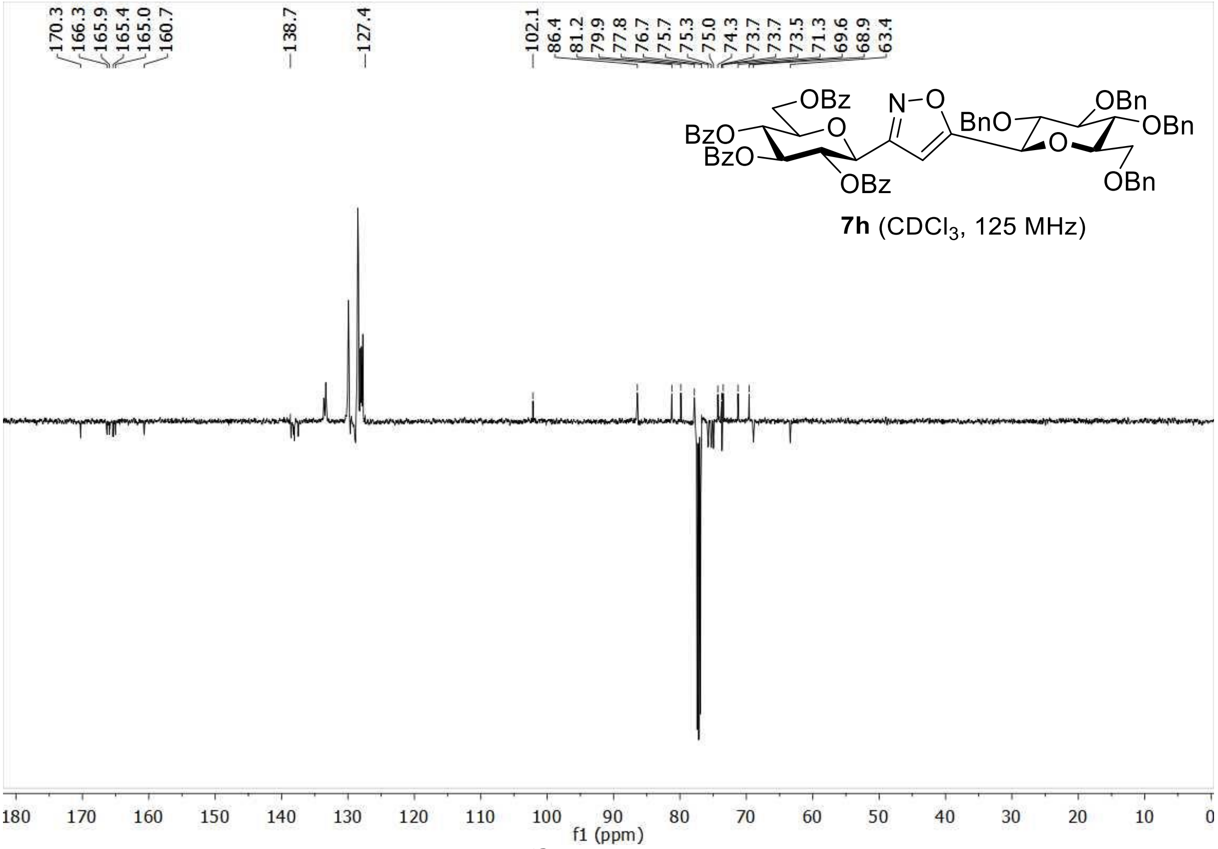

Figure S75. ^{13}C NMR spectrum of **7h**

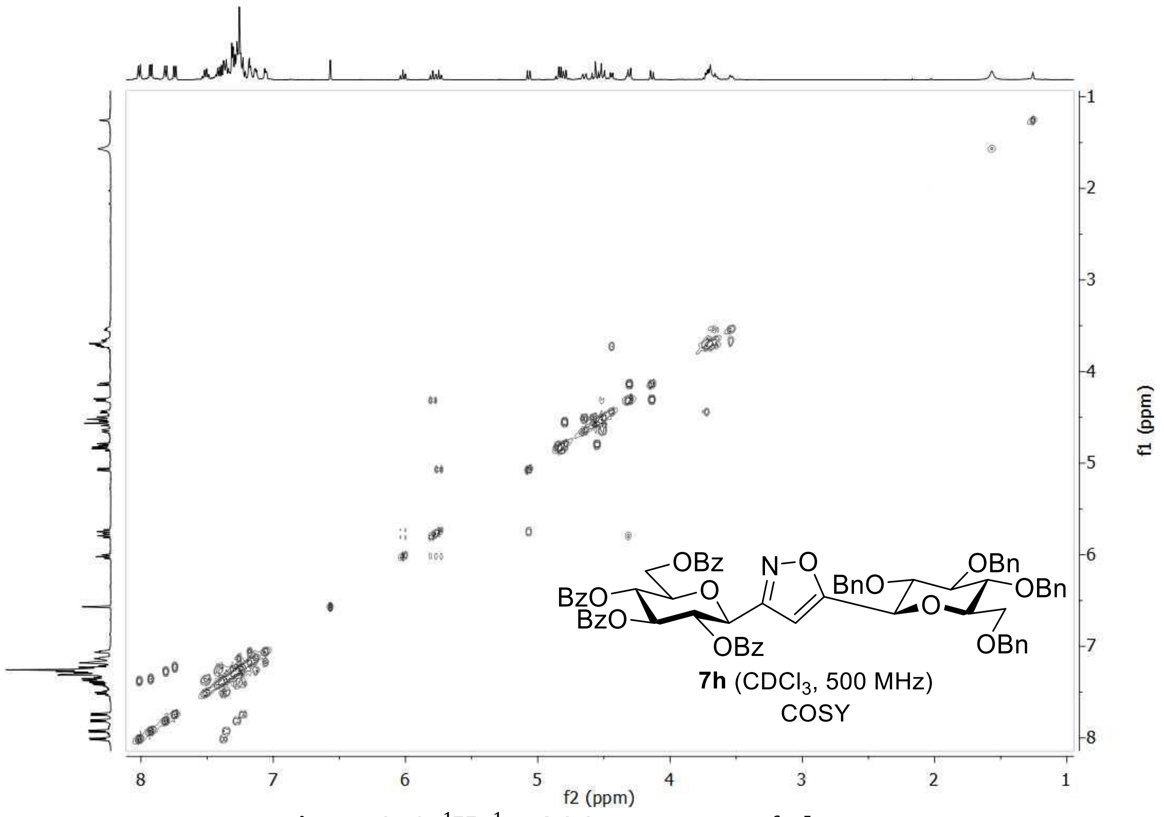

Figure S76. $^{1}H–^{1}H$ COSY spectrum of **7h**

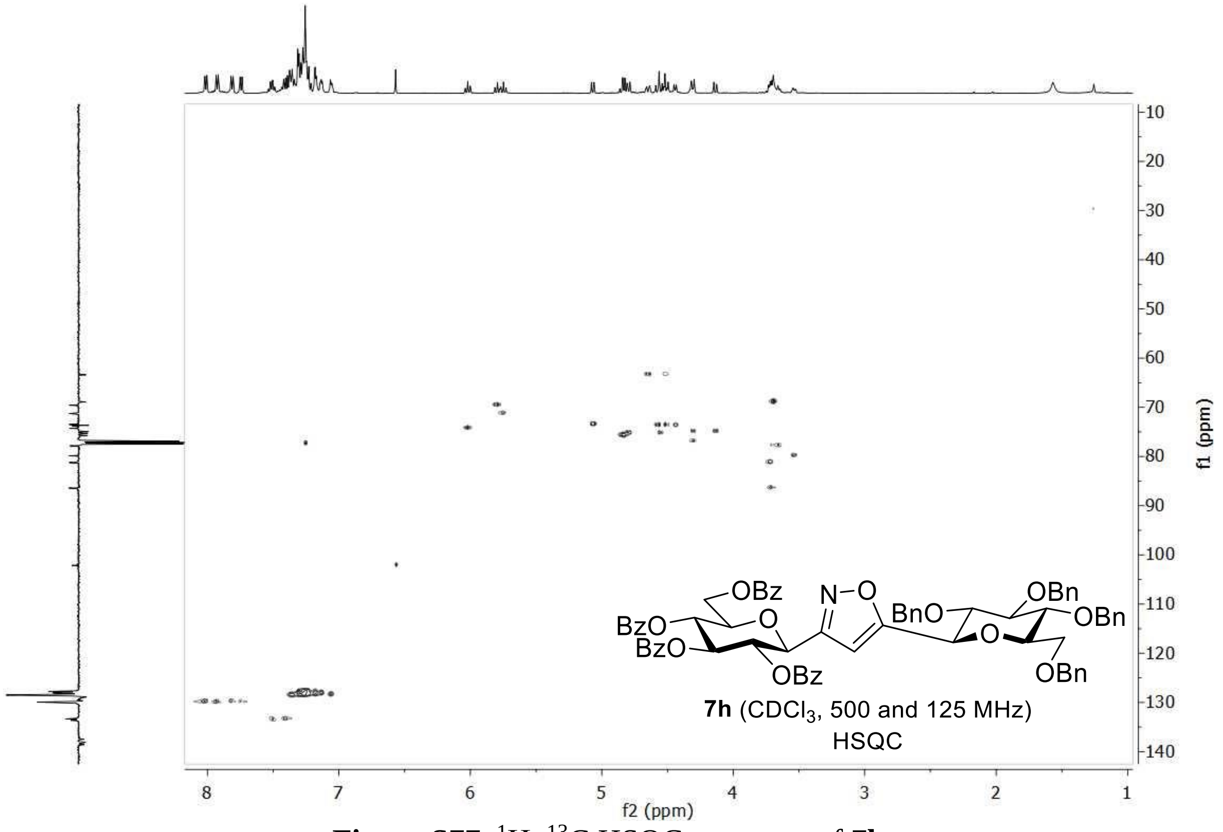

Figure S77. $^{1}H–^{13}C$ HSQC spectrum of **7h**

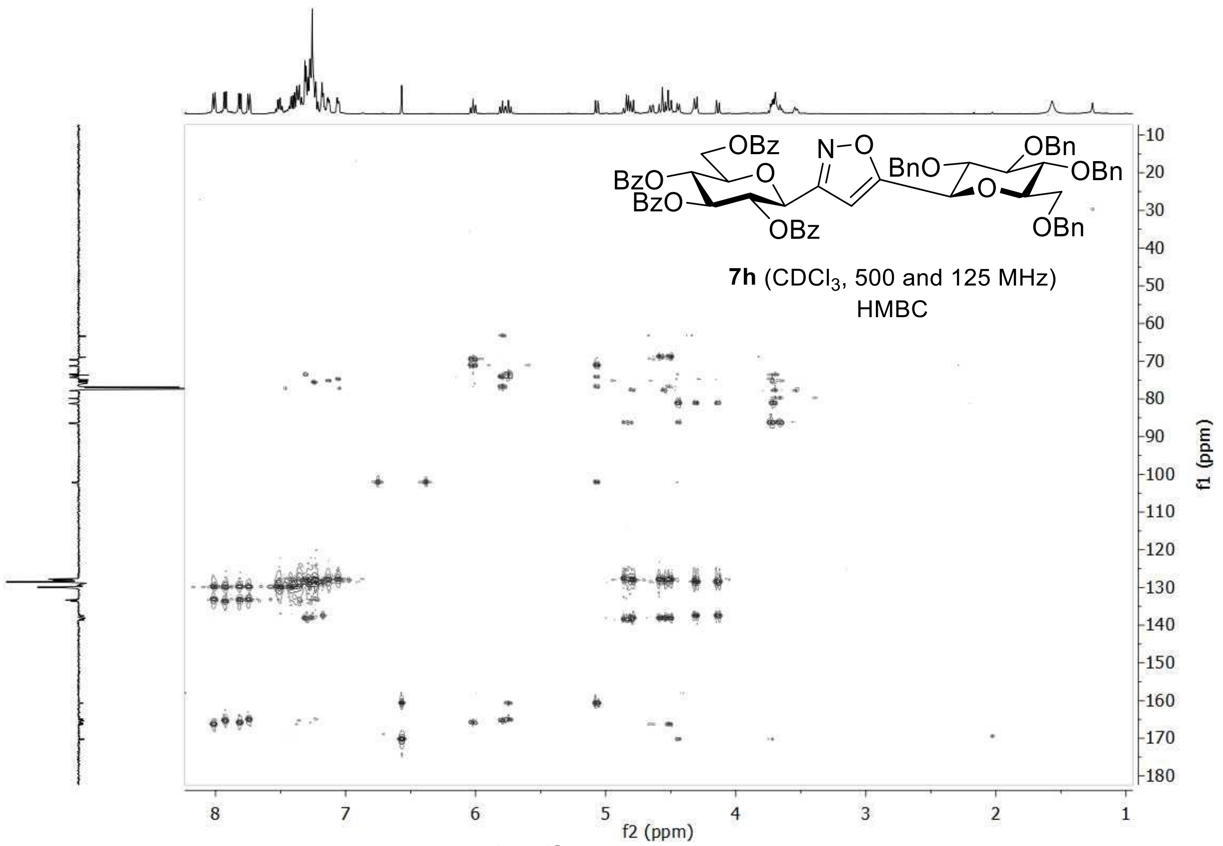

Figure S78. ^{1}H–^{13}C HMBC spectrum of **7h**

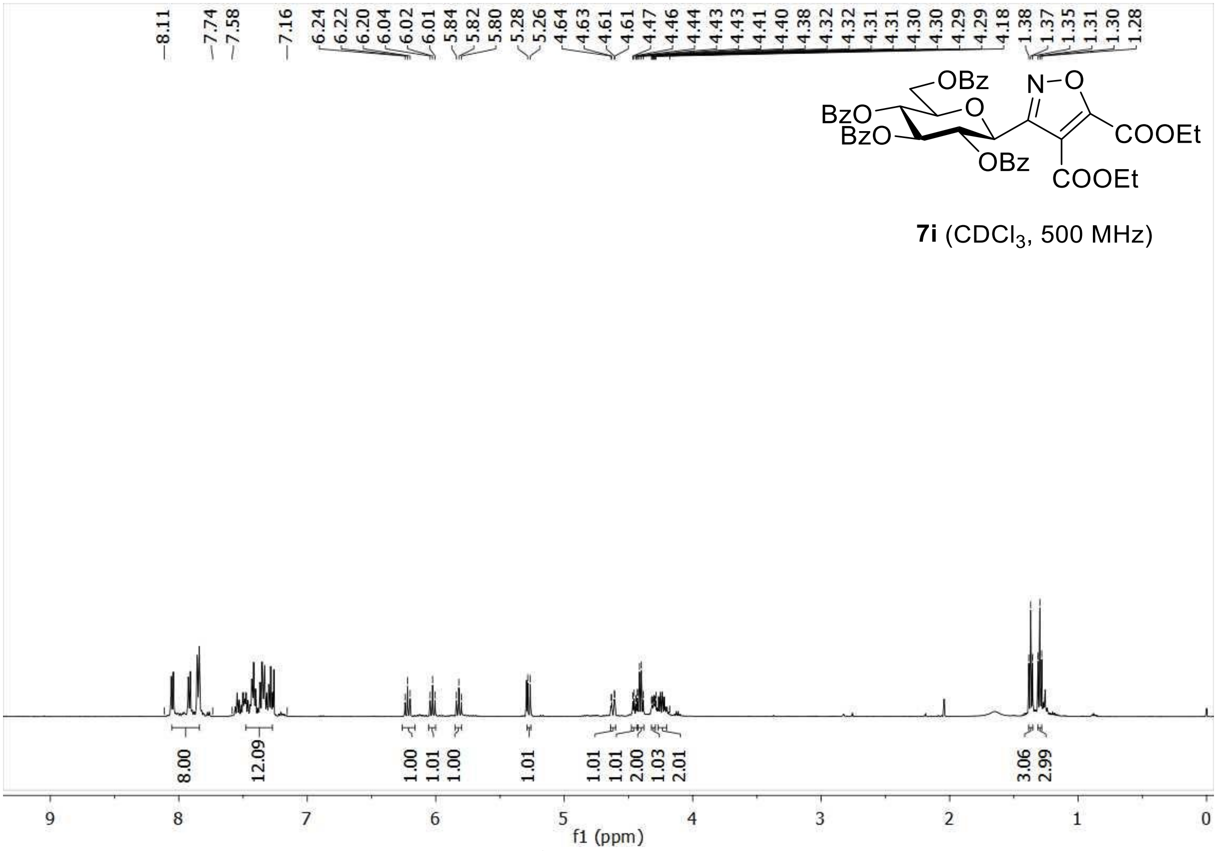

Figure S79. ^{1}H NMR spectrum of **7i**

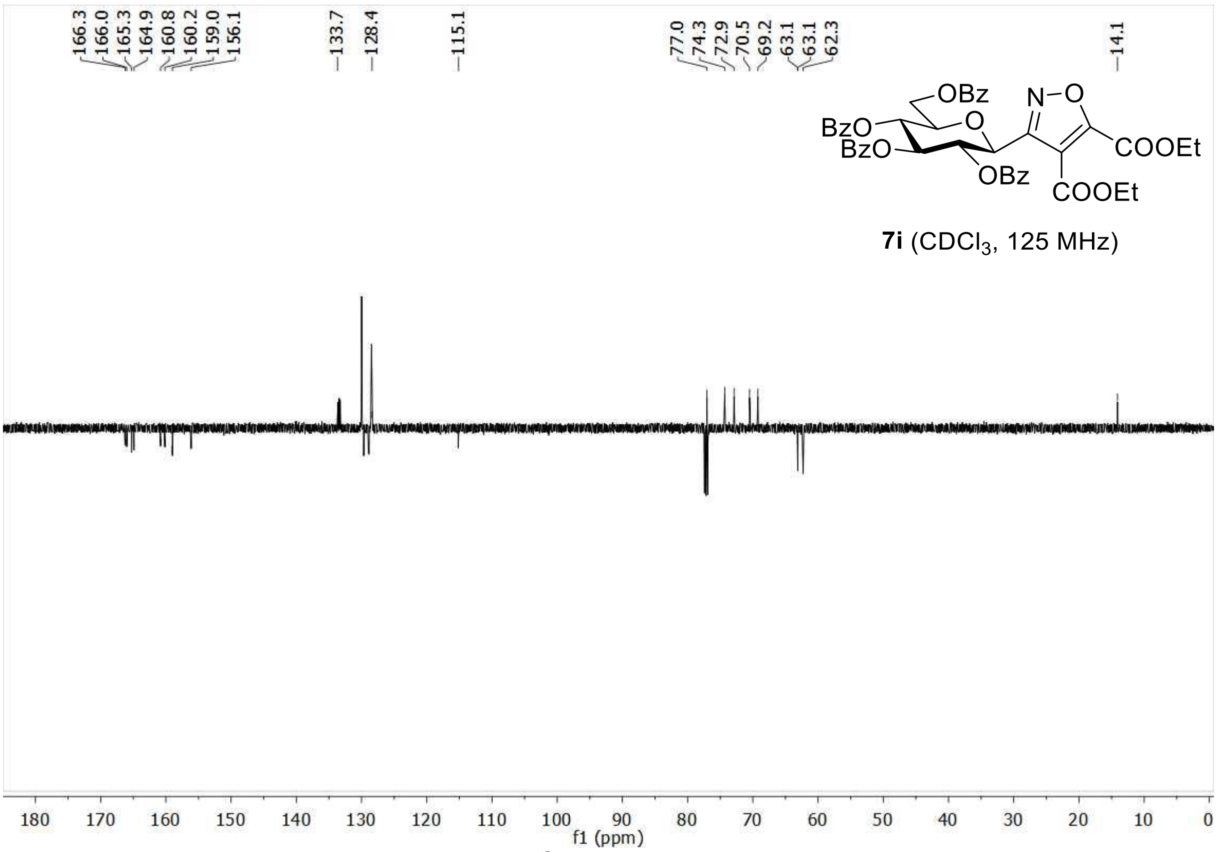

Figure S80. ^{13}C NMR spectrum of **7i**

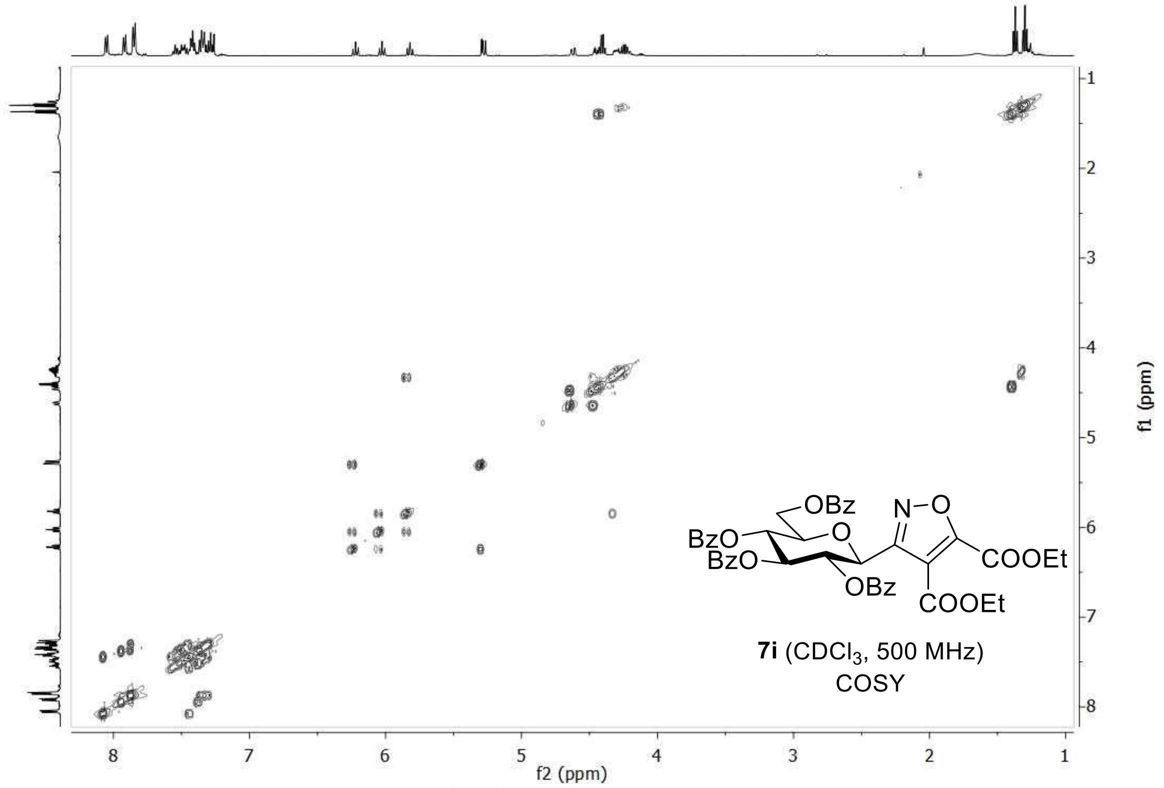

Figure S81. ^{1}H–^{1}H COSY spectrum of **7i**

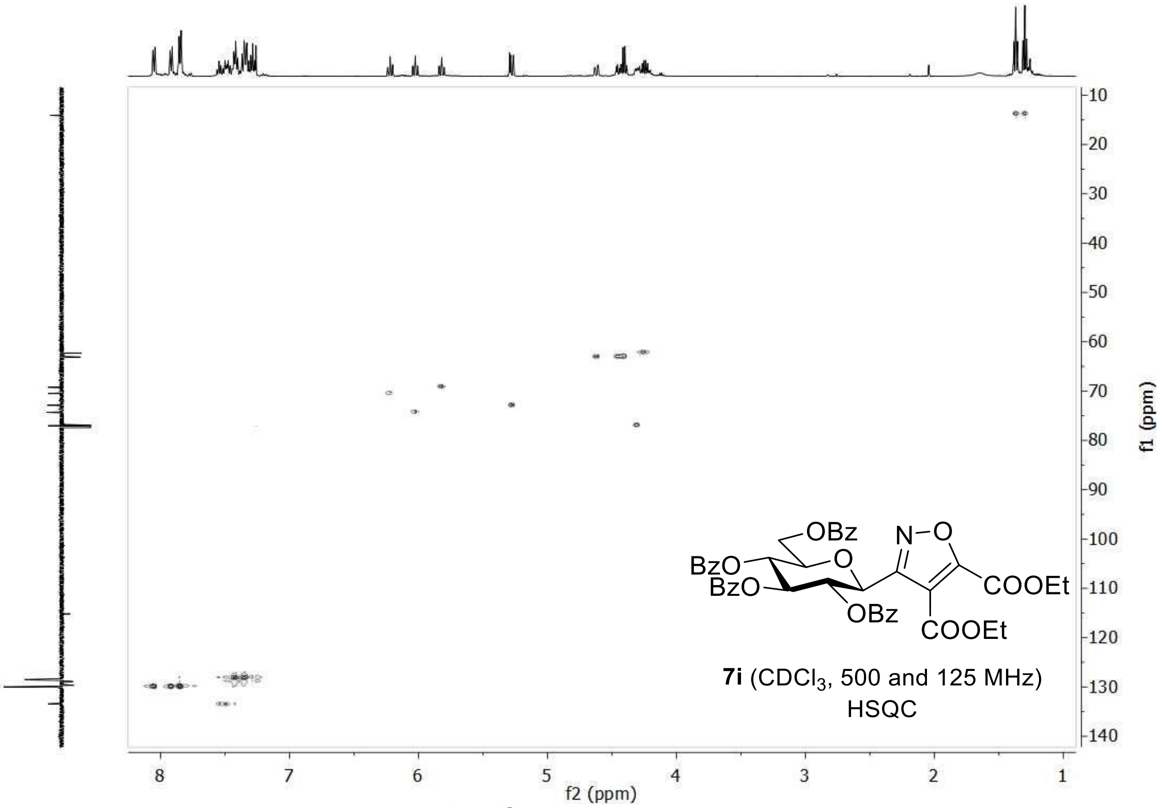

Figure S82. ^{1}H–^{13}C HSQC spectrum of **7i**

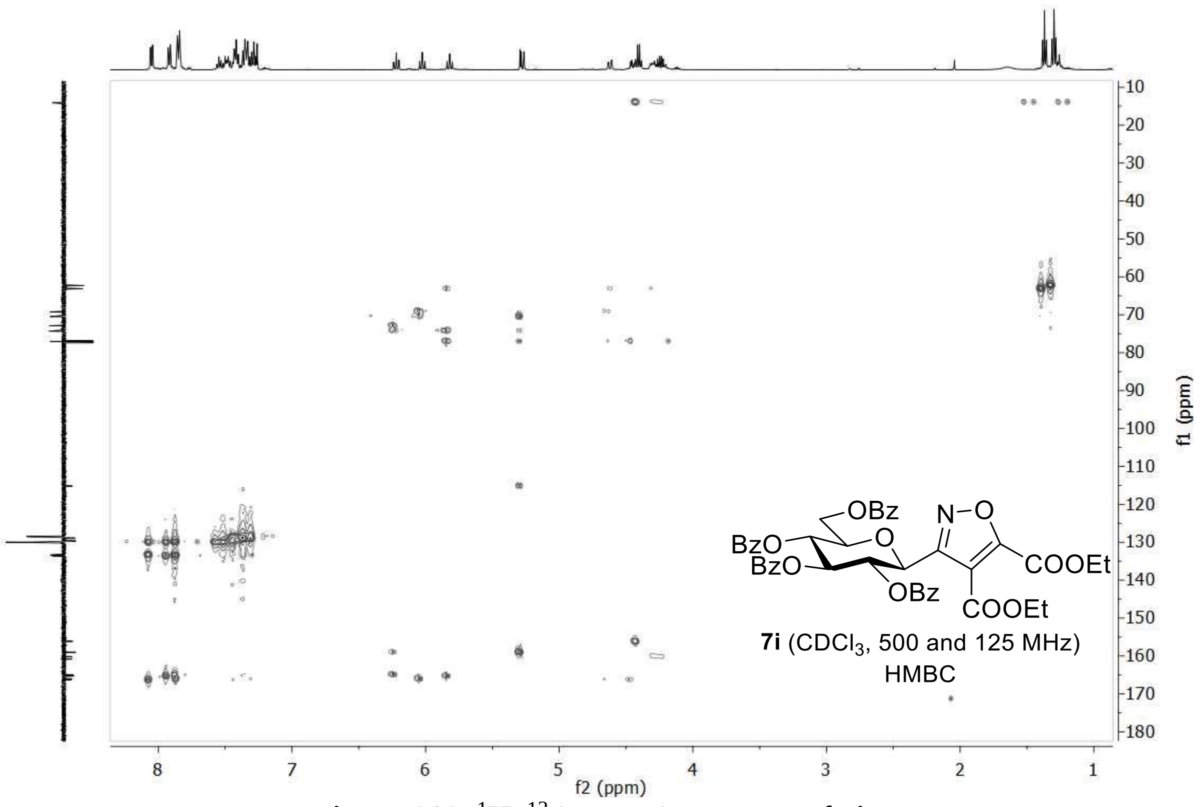

Figure S83. ^{1}H–^{13}C HMBC spectrum of **7i**

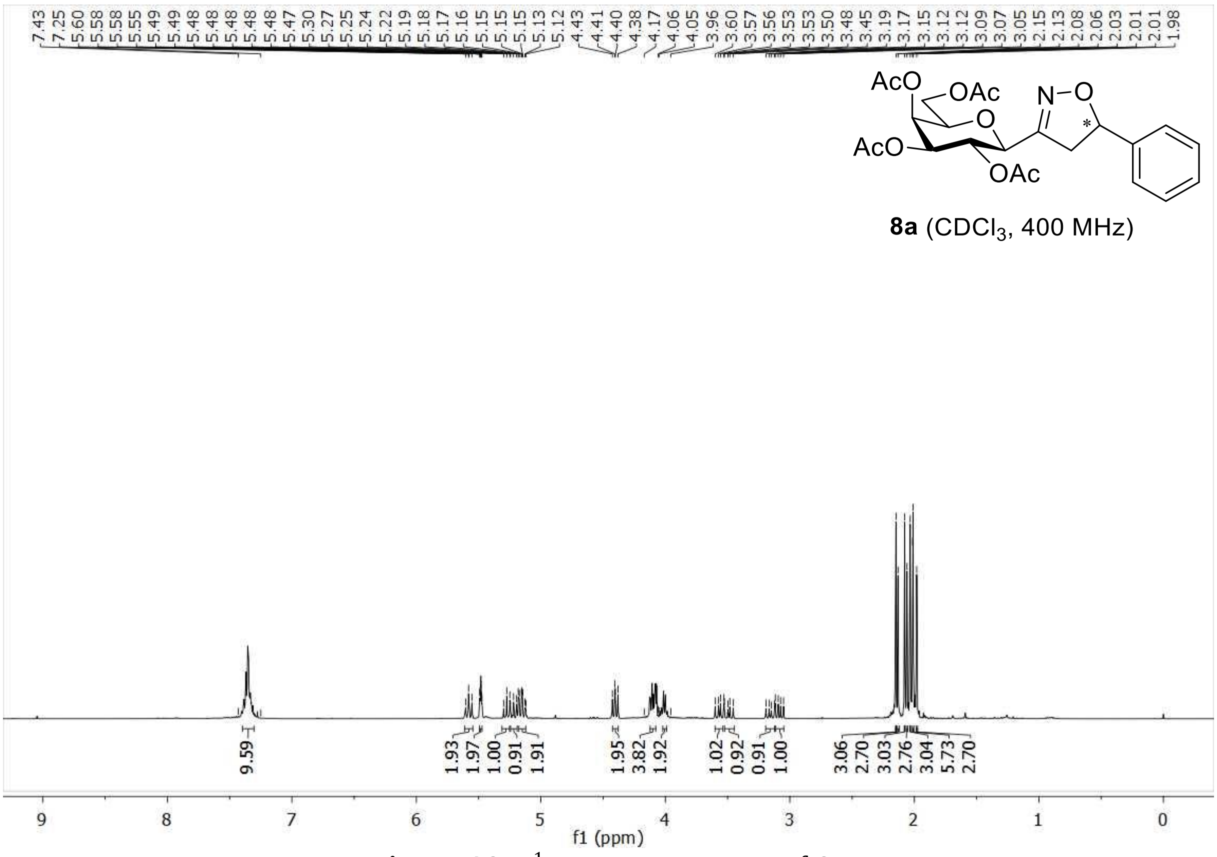

Figure S84. ^{1}H NMR spectrum of **8a**

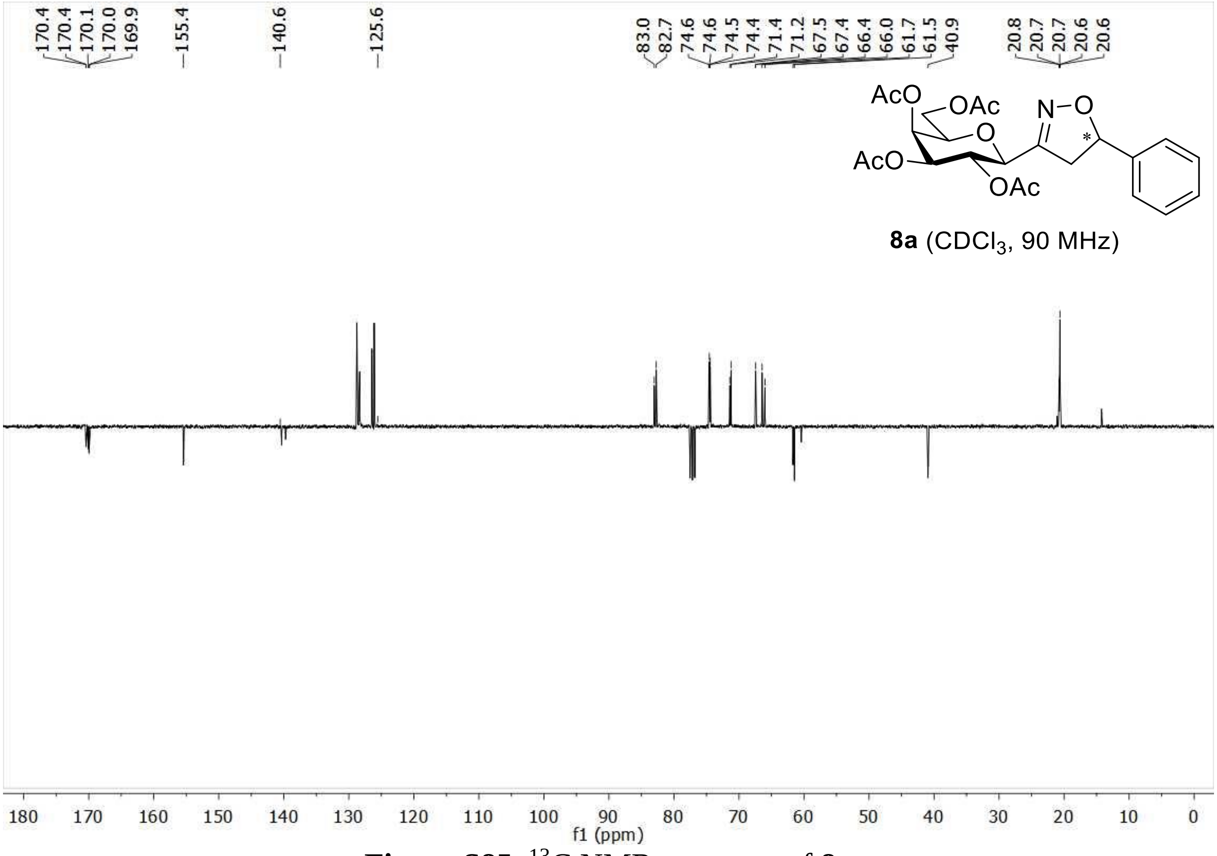

Figure S85. ^{13}C NMR spectrum of **8a**

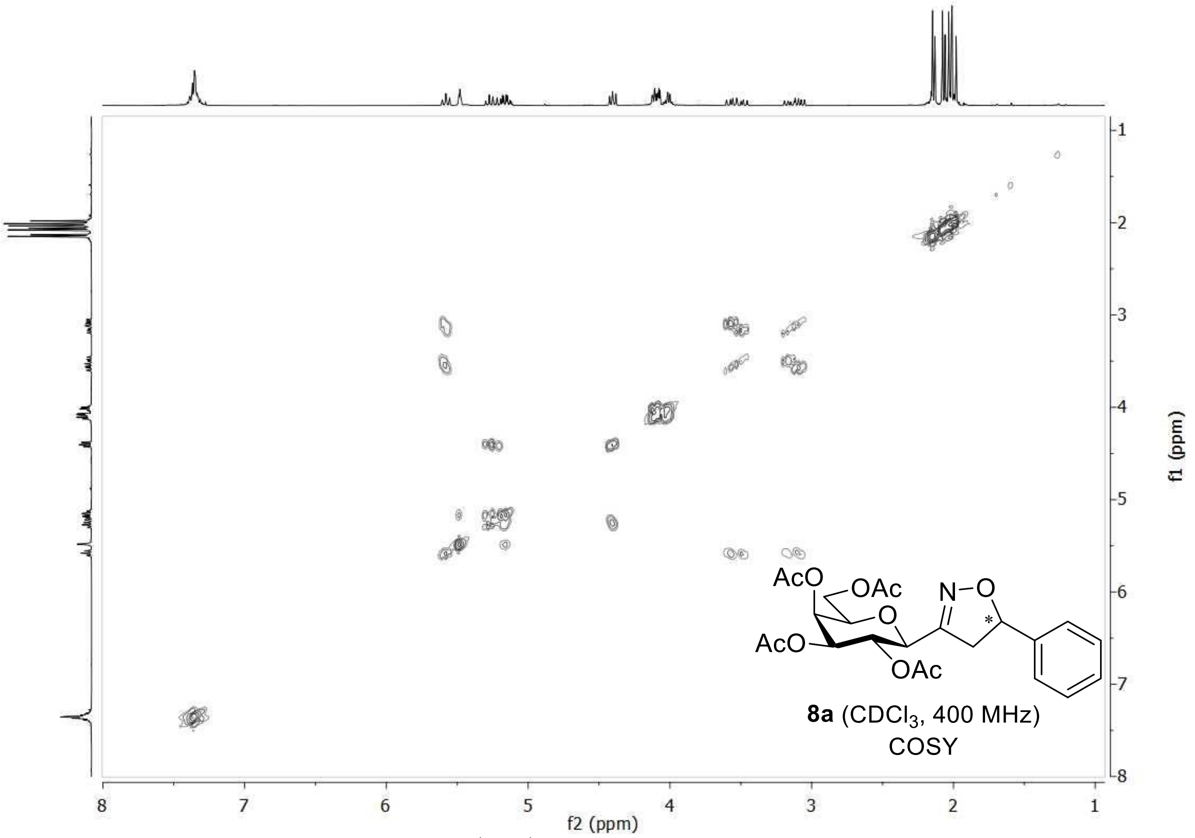

Figure S86. ^{1}H–^{1}H COSY spectrum of **8a**

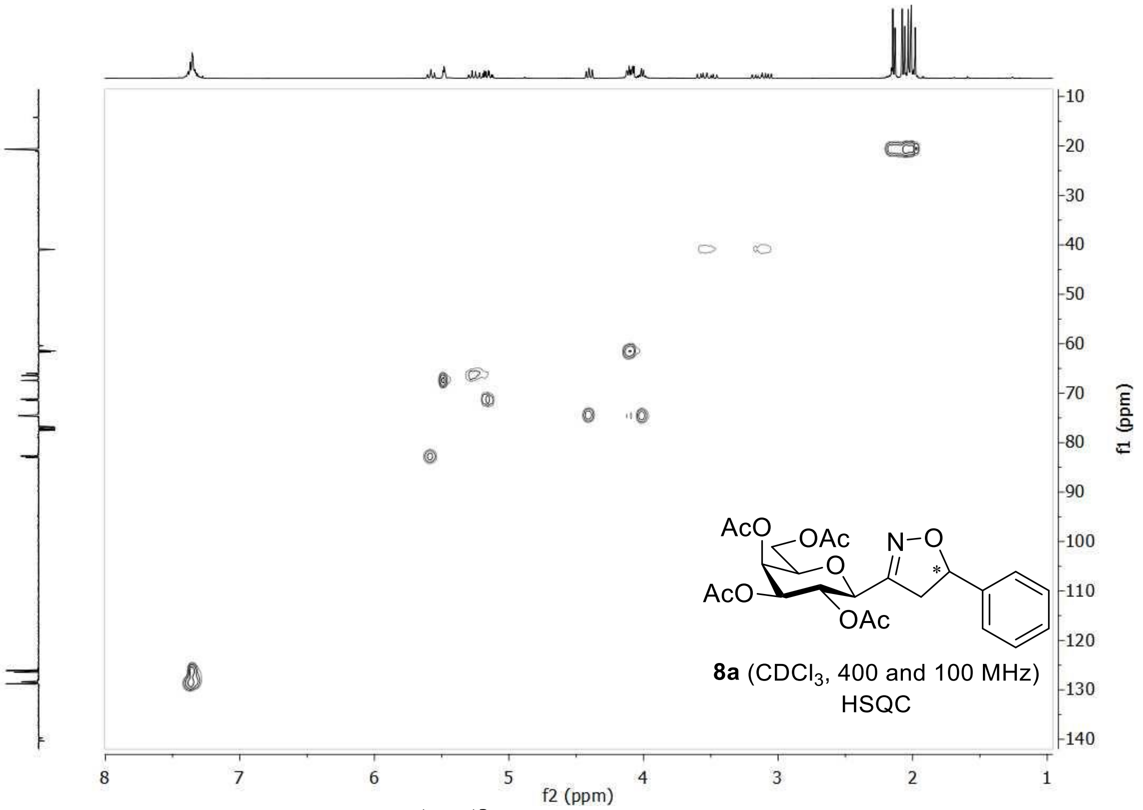

Figure S87. ^{1}H–^{13}C HSQC spectrum of **8a**

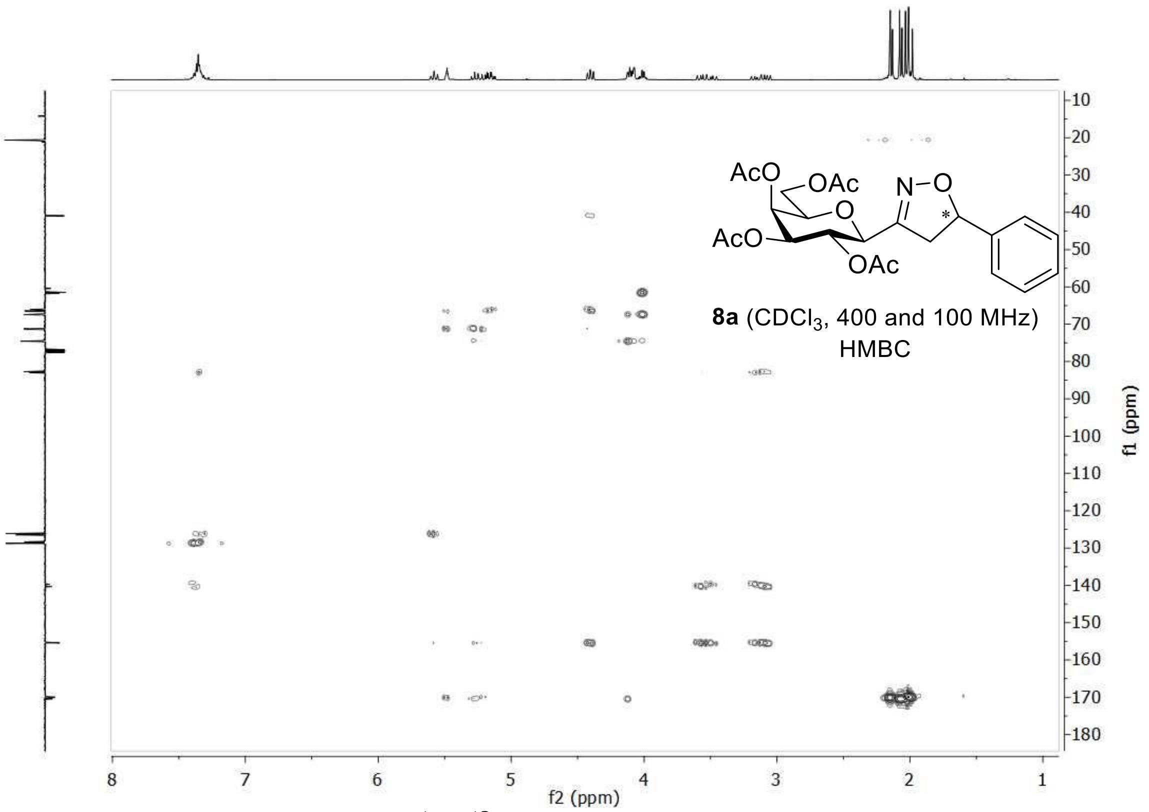

Figure S88. ^{1}H–^{13}C HMBC spectrum of **8a**

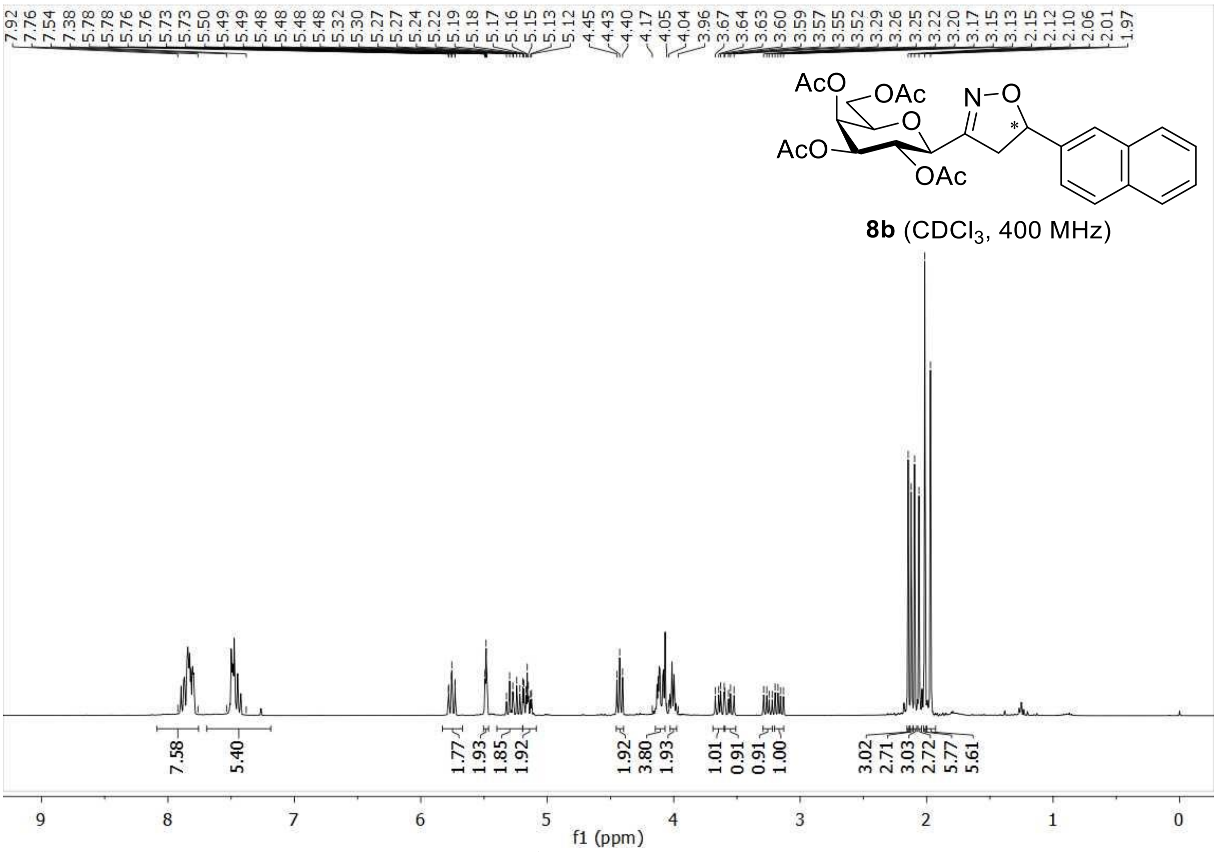

Figure S89. ^{1}H NMR spectrum of **8b**

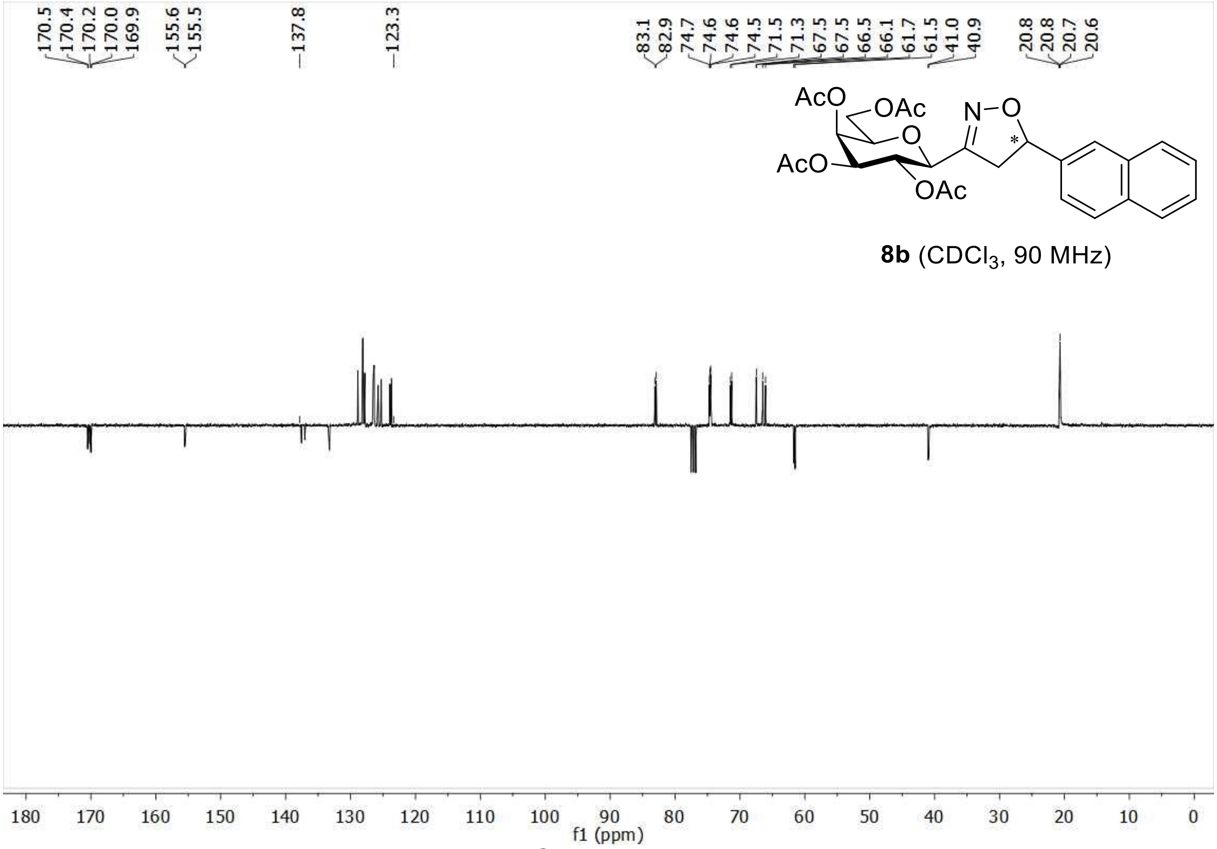

Figure S90. ^{13}C NMR spectrum of **8b**

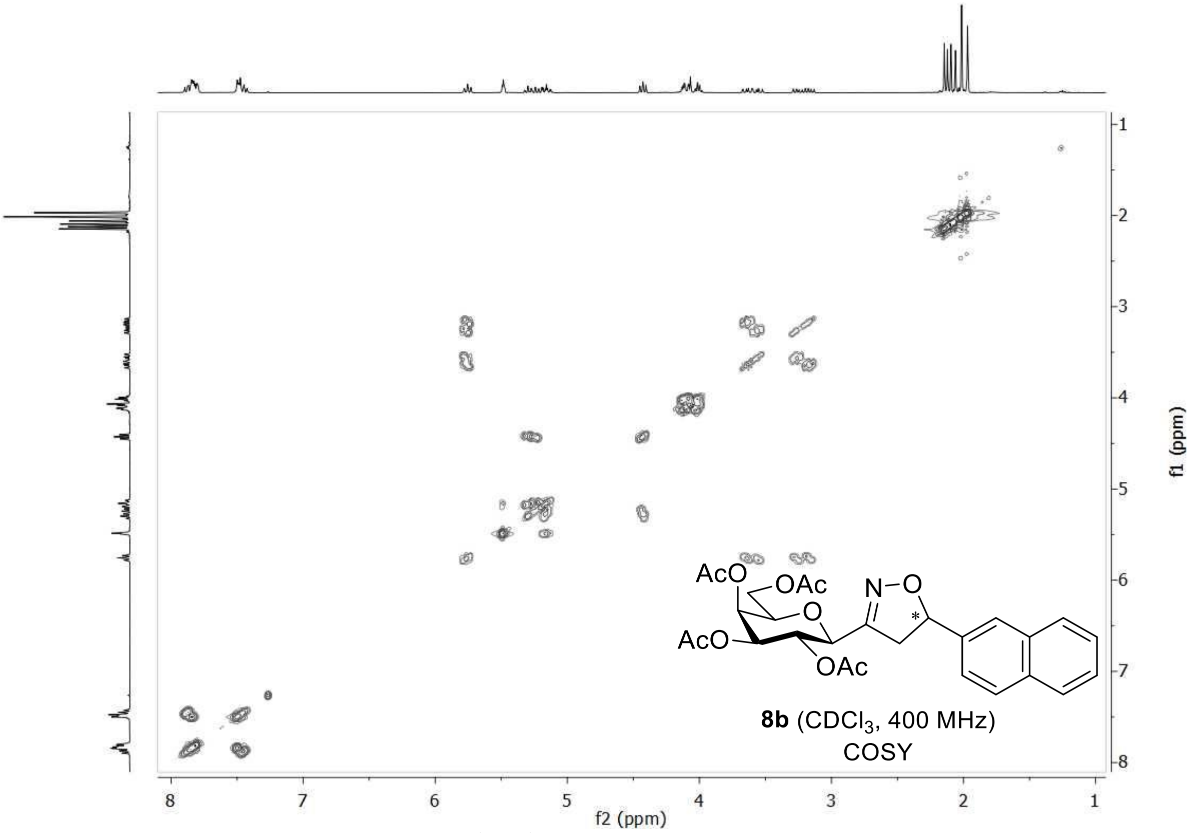

Figure S91. ^{1}H–^{1}H COSY spectrum of **8b**

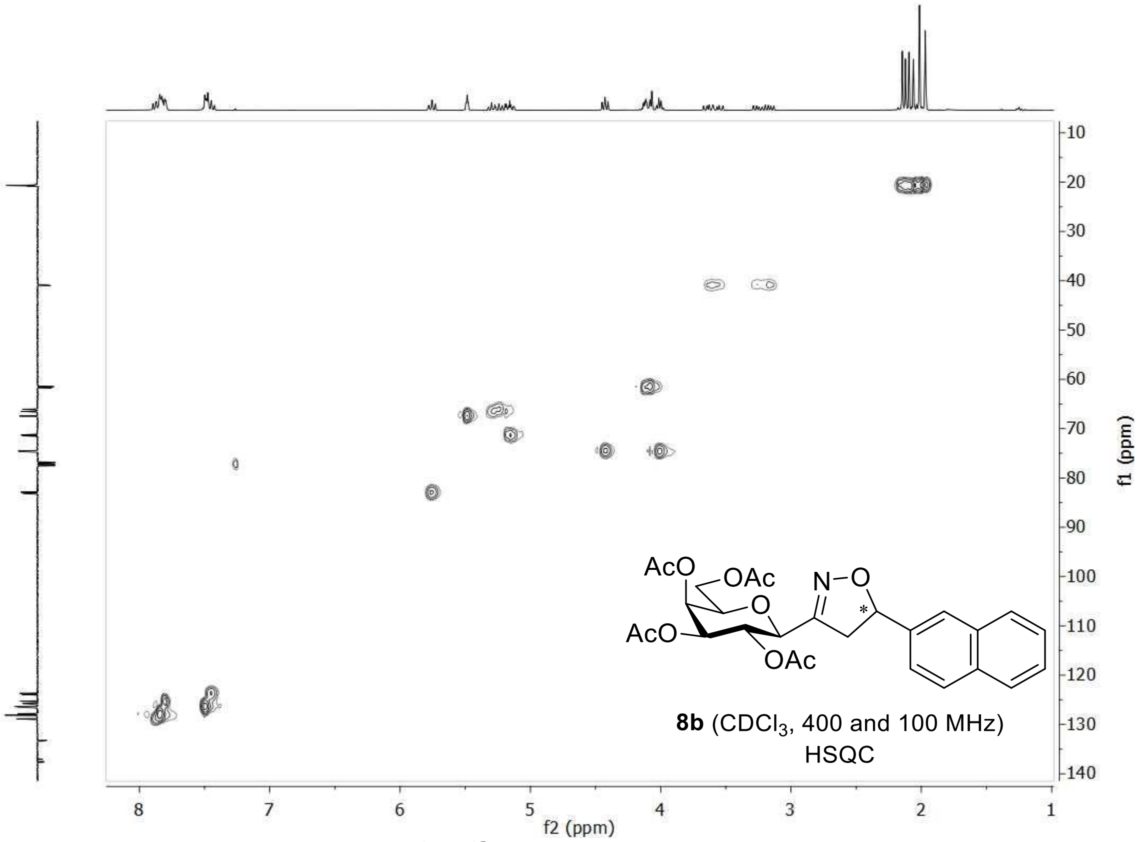

Figure S92. ^{1}H–^{13}C HSQC spectrum of **8b**

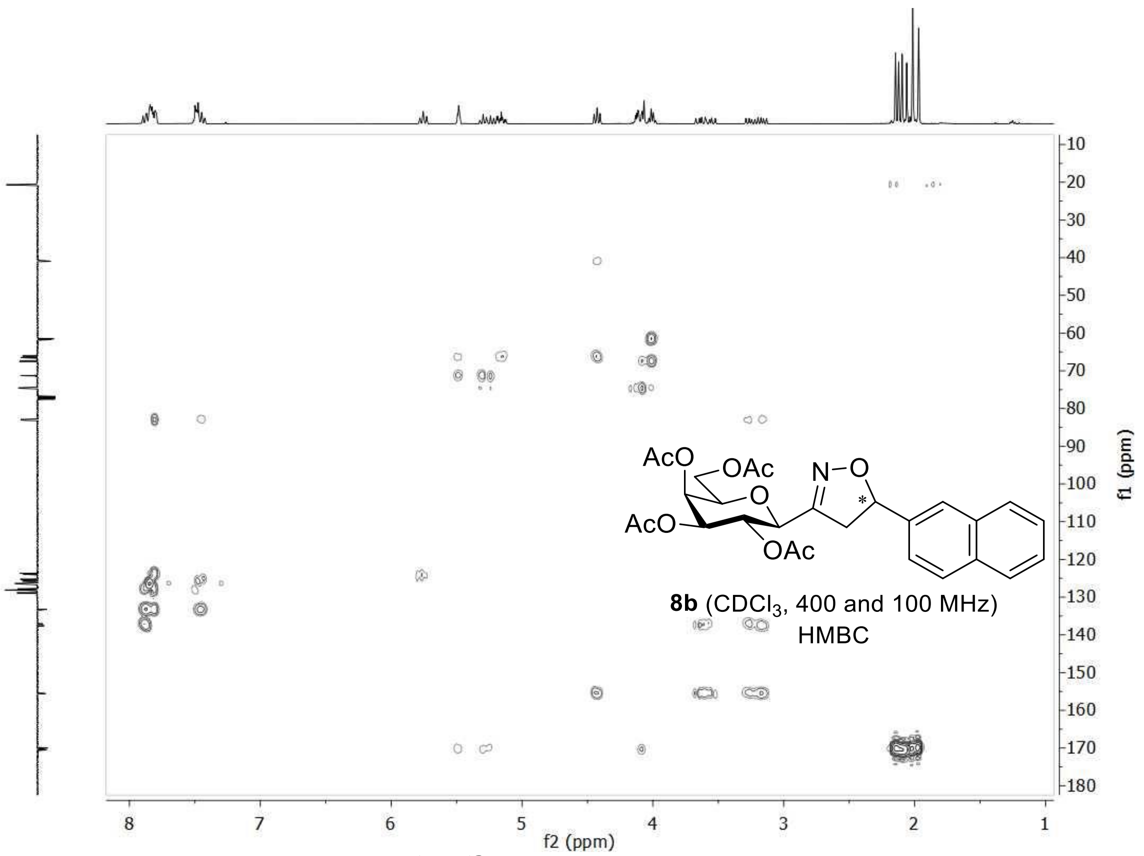

Figure S93. $^{1}H–^{13}C$ HMBC spectrum of **8b**

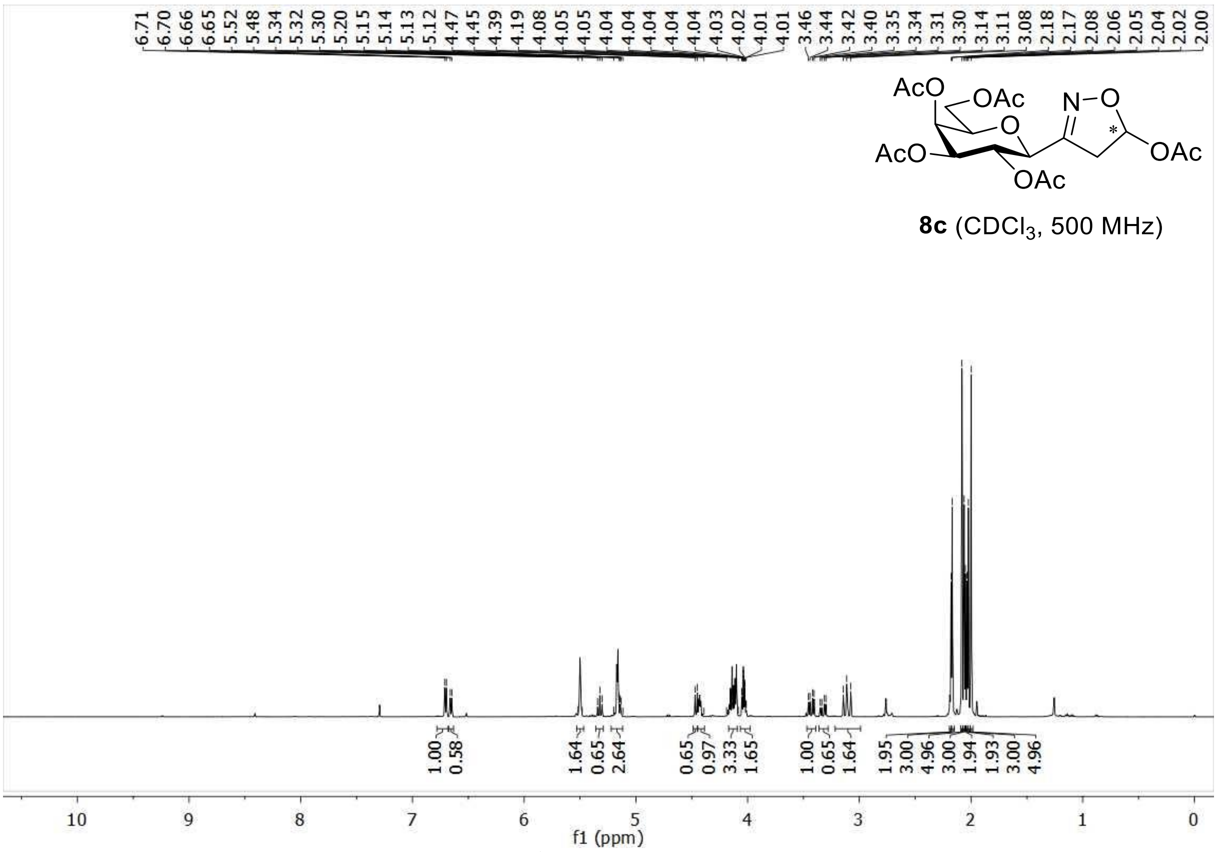

Figure S94. ^{1}H NMR spectrum of **8c**

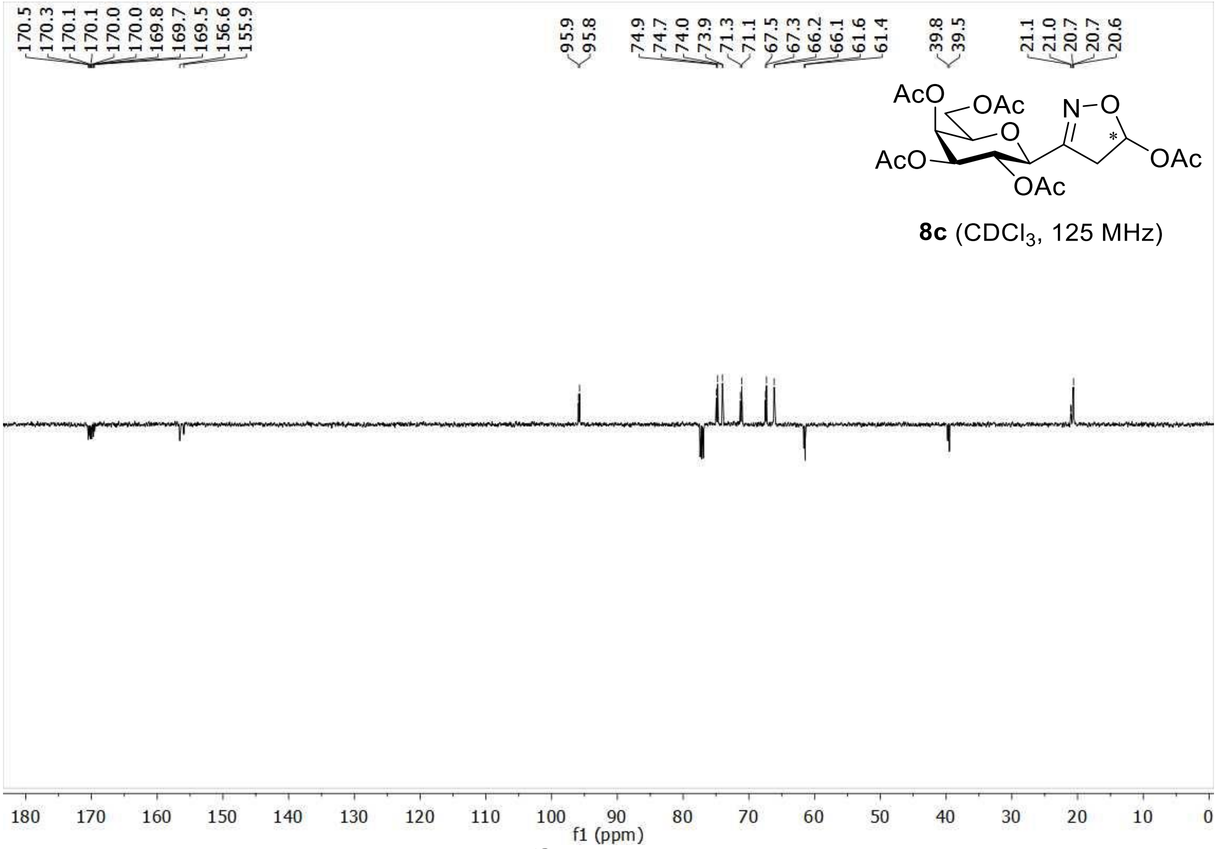

Figure S95. ^{13}C NMR spectrum of **8c**

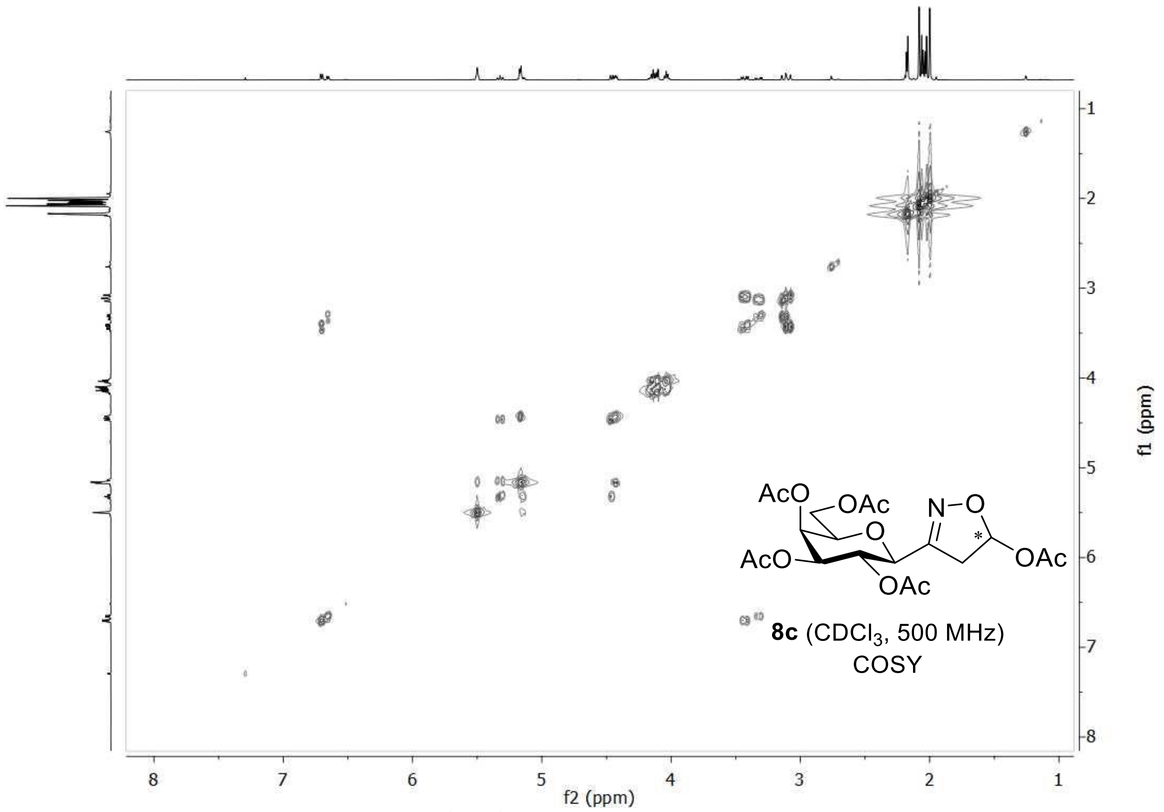

Figure S96. ^{1}H–^{1}H COSY spectrum of **8c**

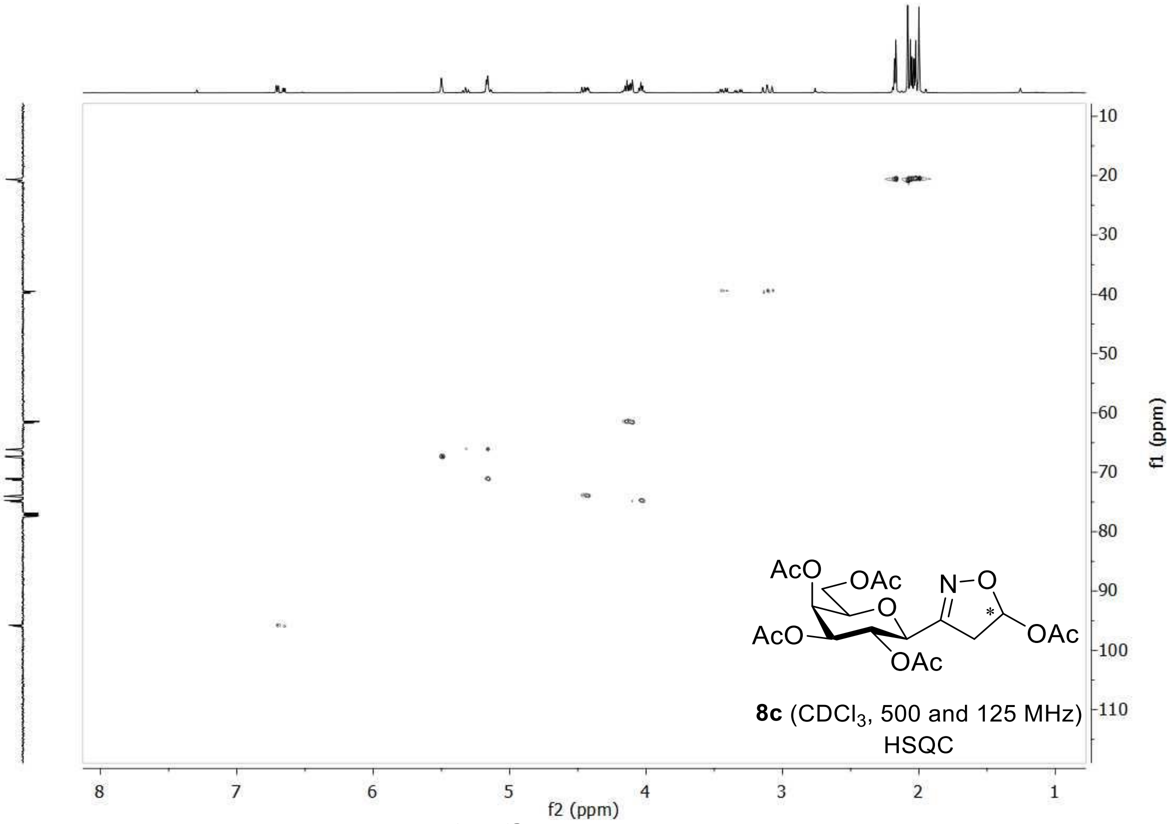

Figure S97. ^{1}H–^{13}C HSQC spectrum of **8c**

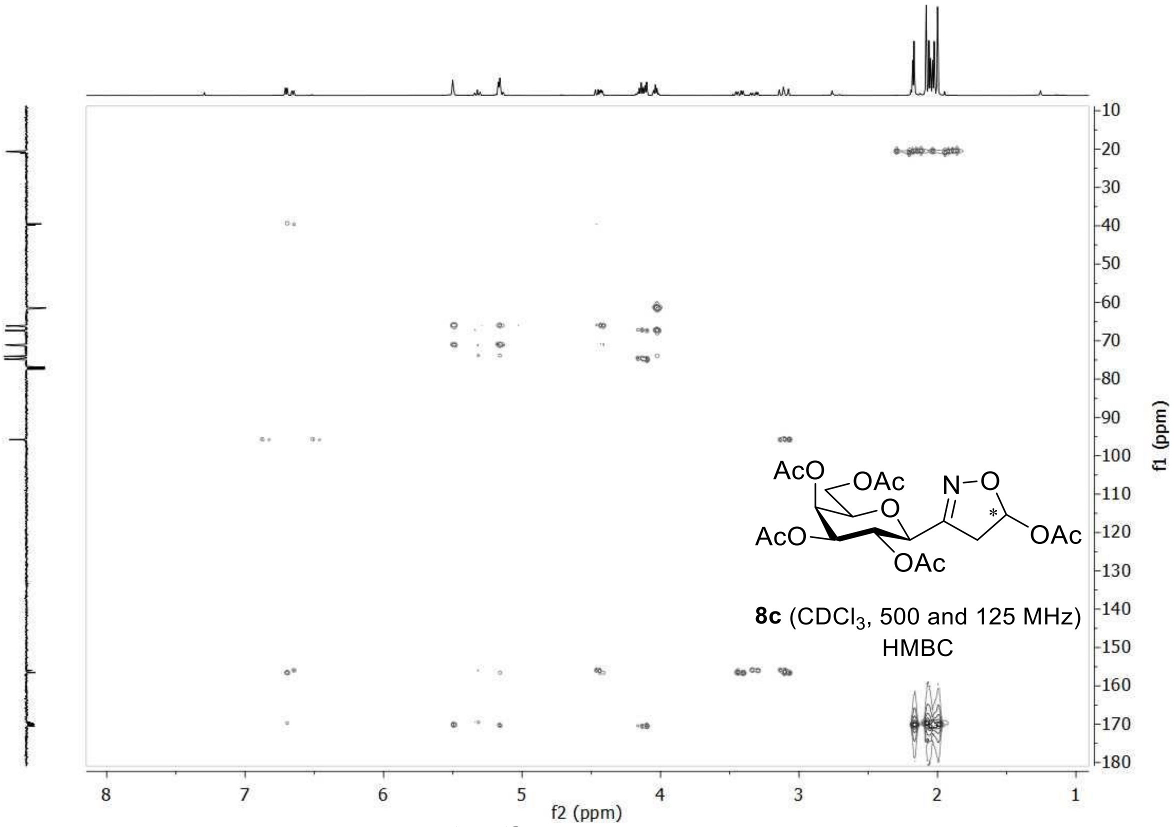

Figure S98. ^{1}H–^{13}C HMBC spectrum of **8c**

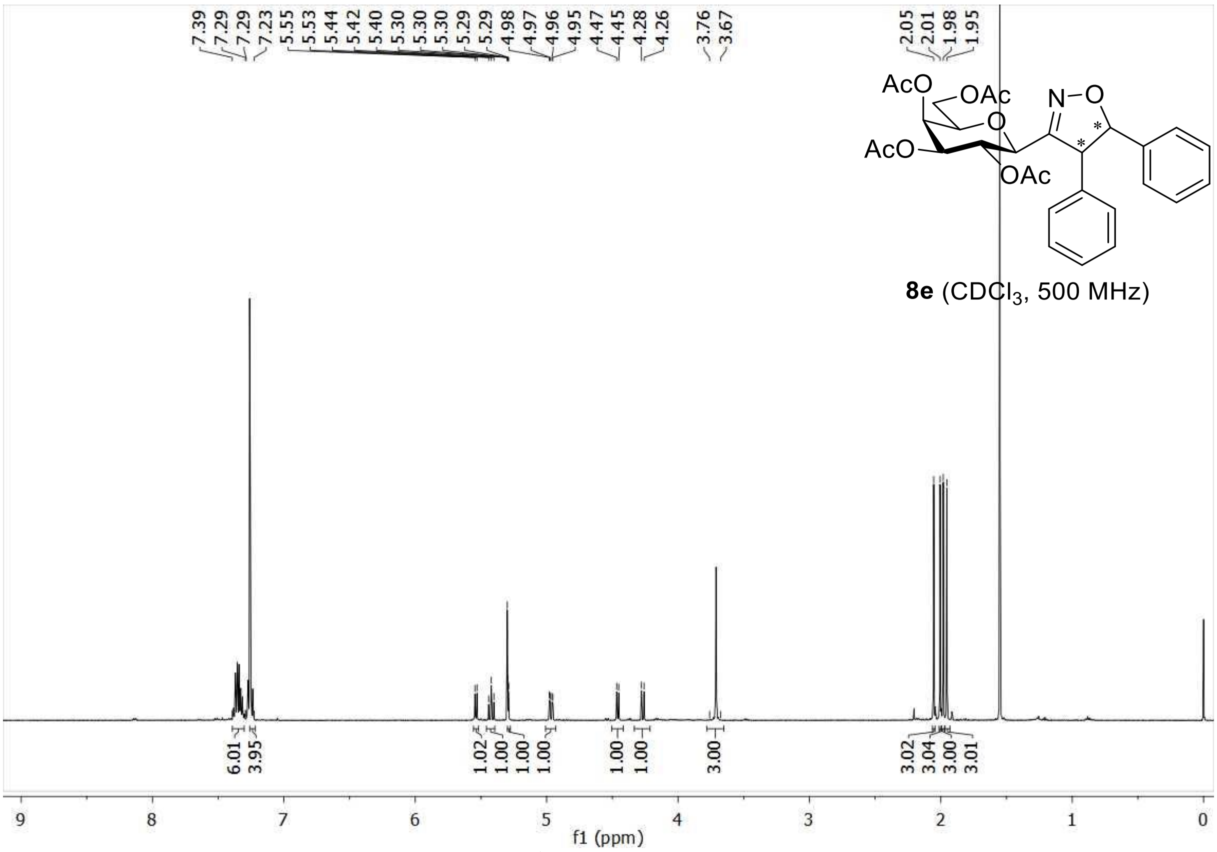

Figure S99. ^{1}H NMR spectrum of **8e**

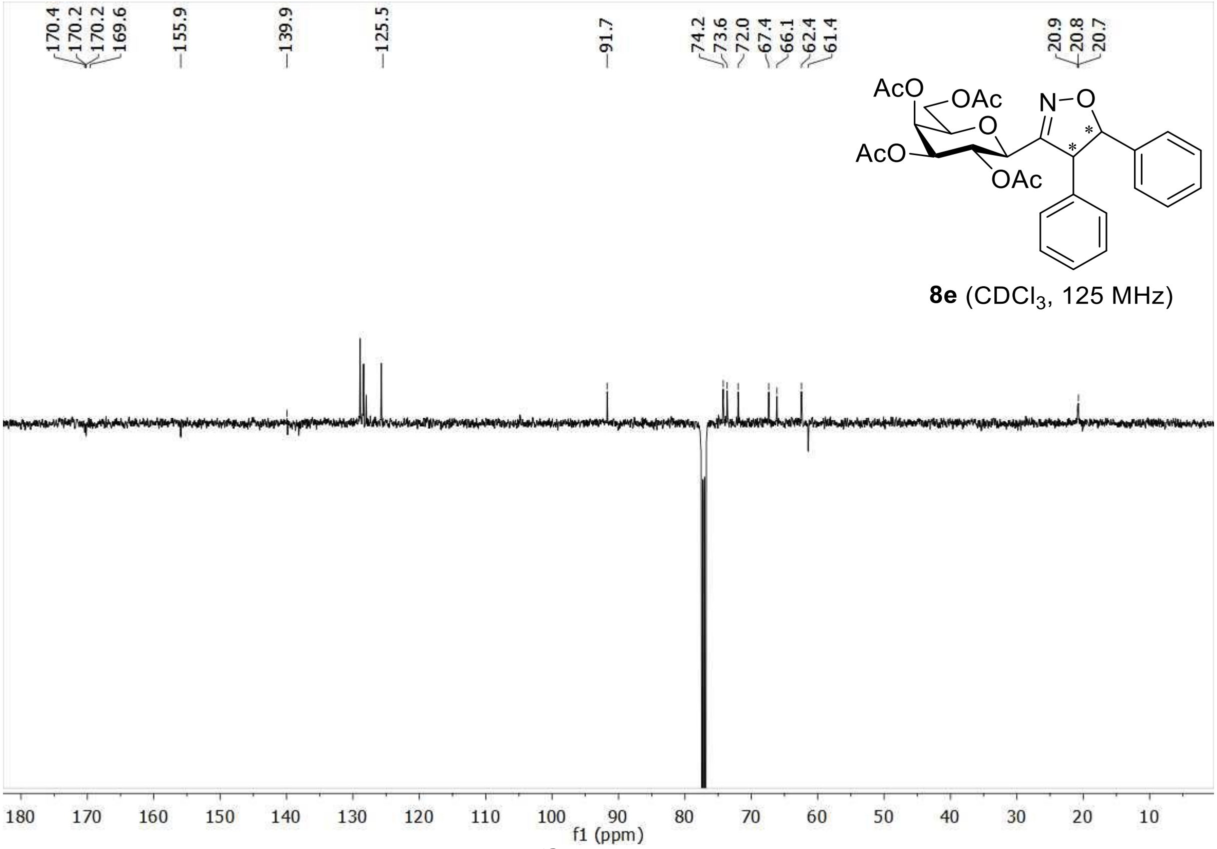

Figure S100. ^{13}C NMR spectrum of **8e**

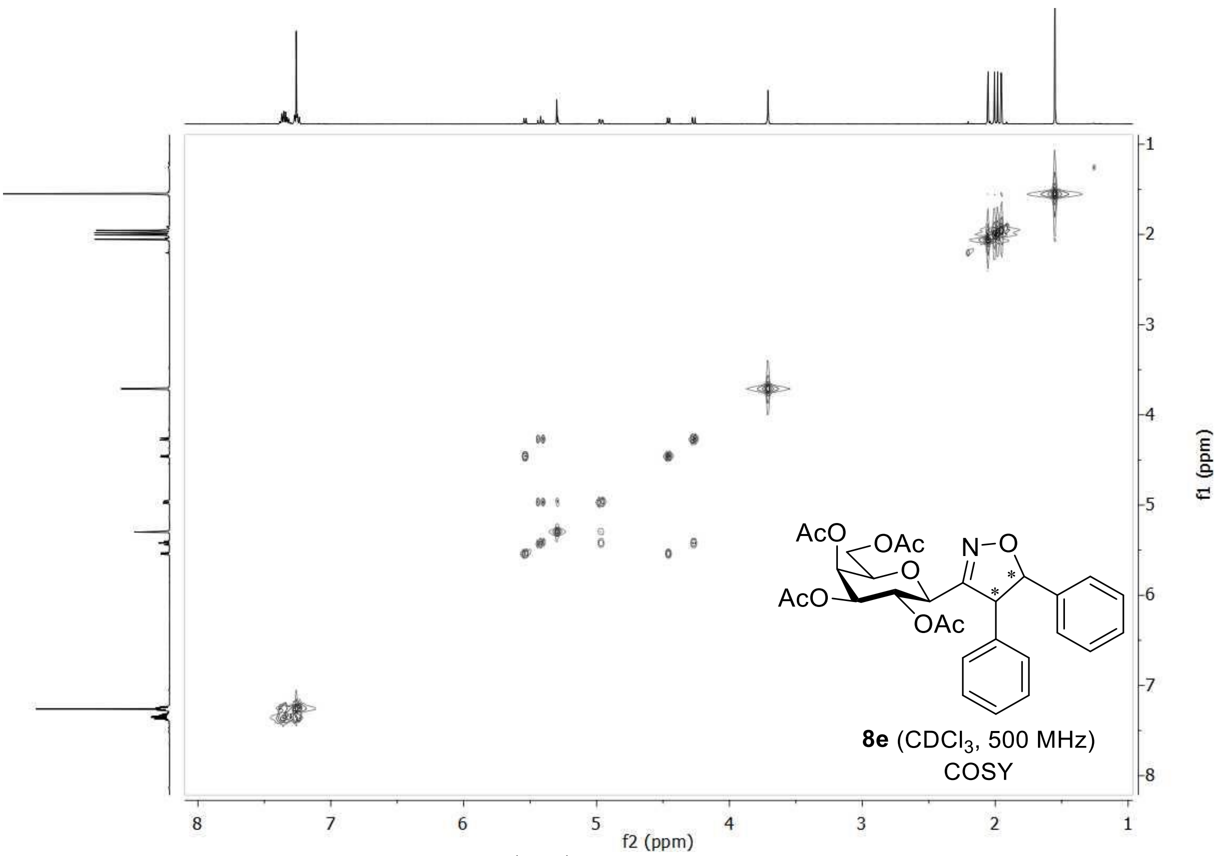

Figure S101. ^{1}H–^{1}H COSY spectrum of **8e**

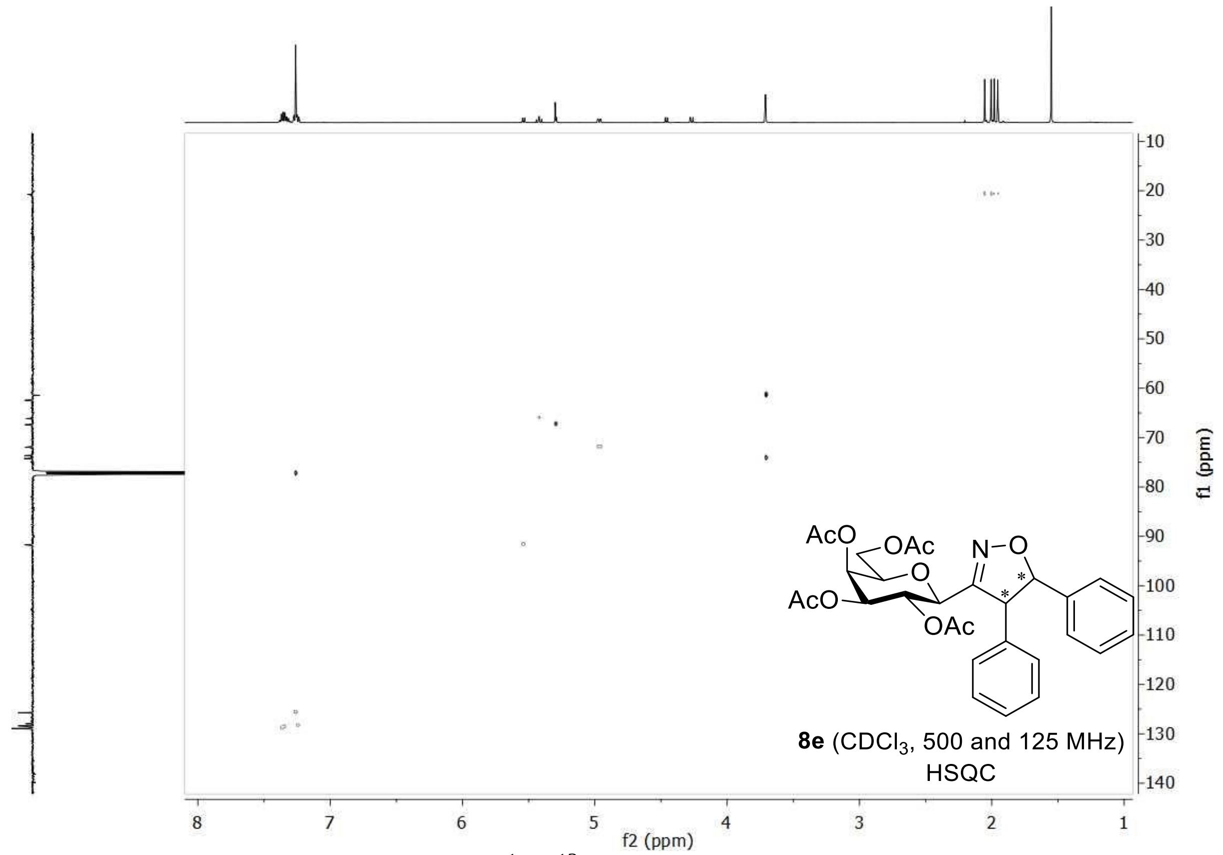

Figure S102. ^{1}H–^{13}C HSQC spectrum of **8e**

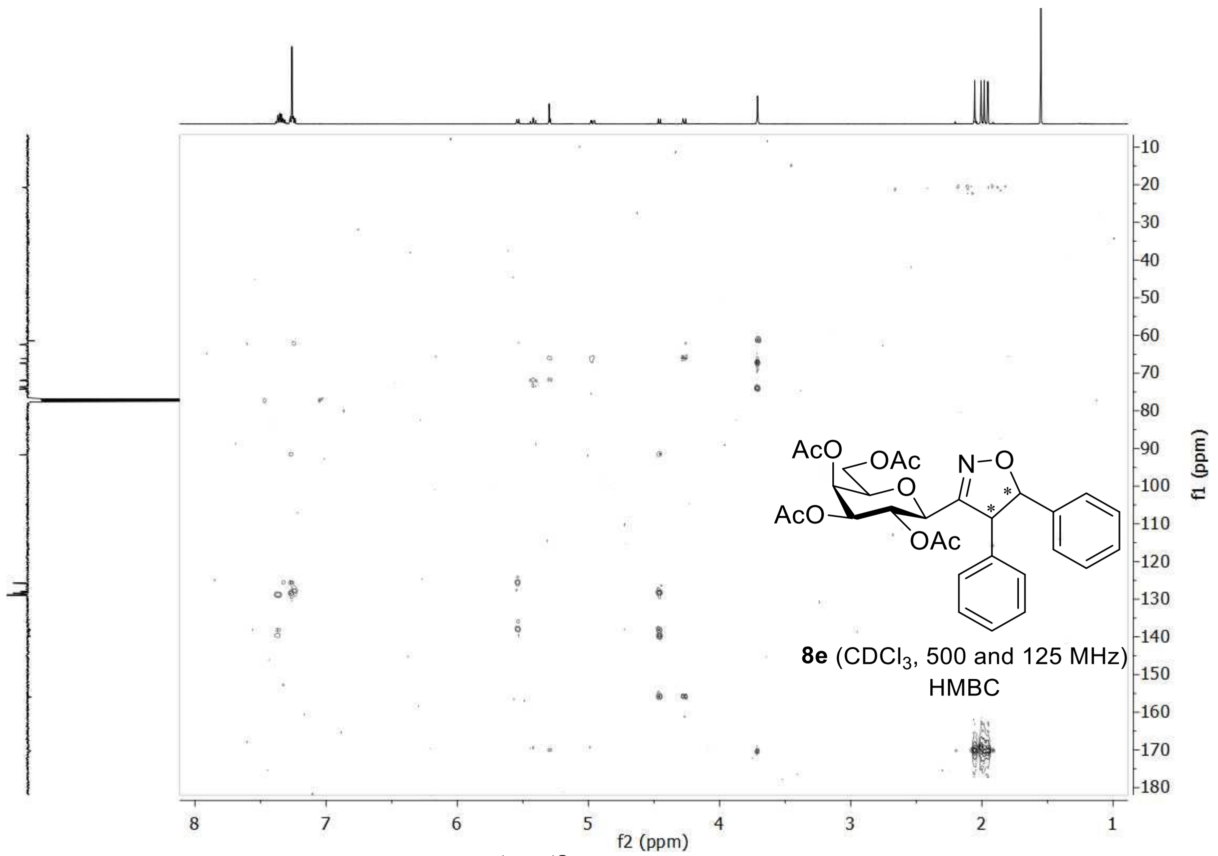

Figure S103. ^{1}H–^{13}C HMBC spectrum of **8e**

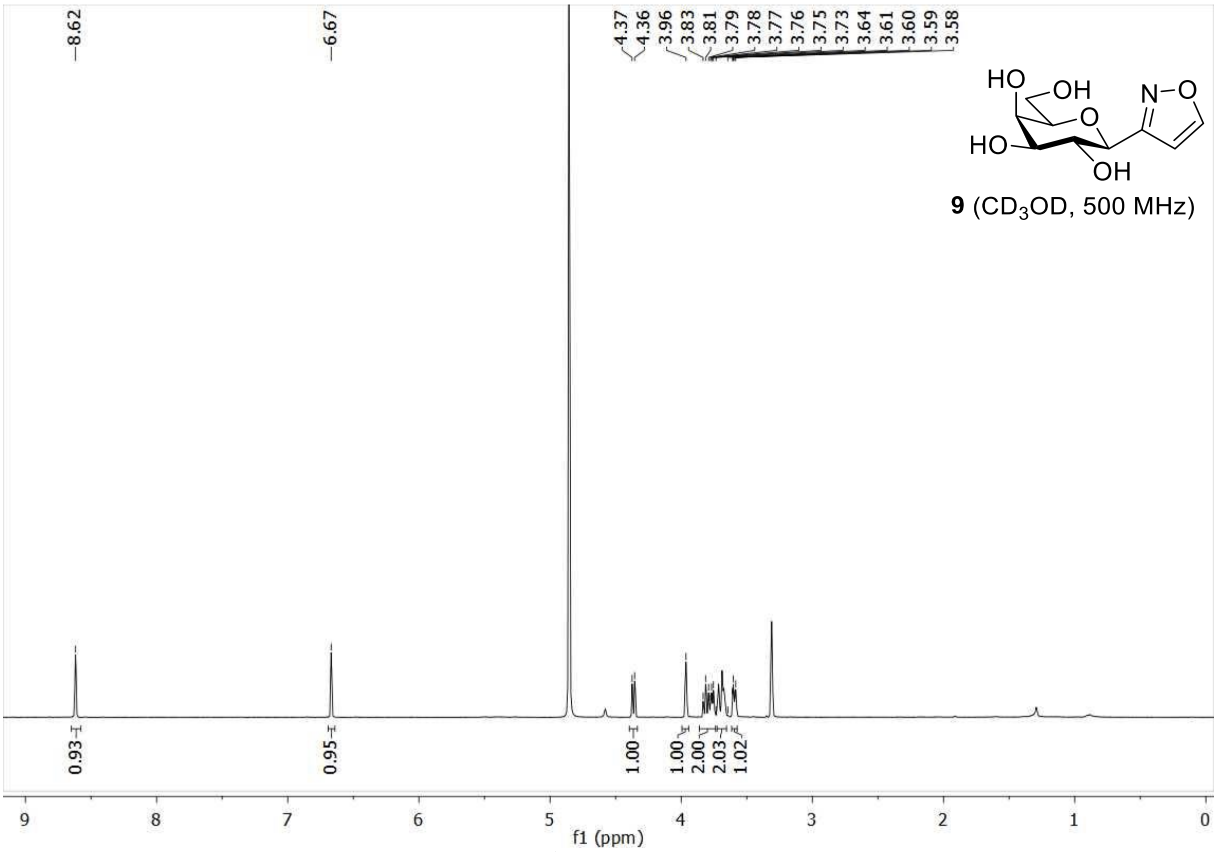

Figure S104. ^{1}H NMR spectrum of **9**

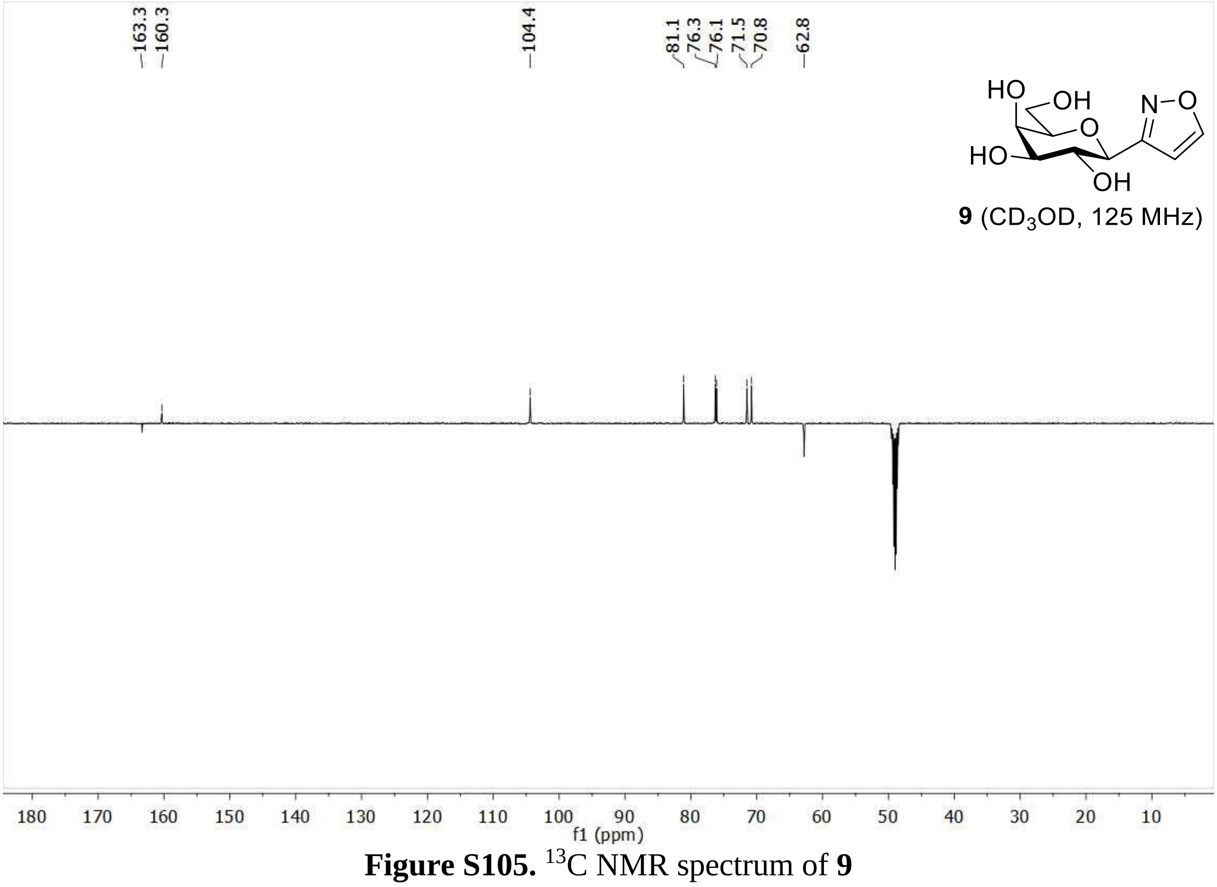

Figure S105. ^{13}C NMR spectrum of **9**

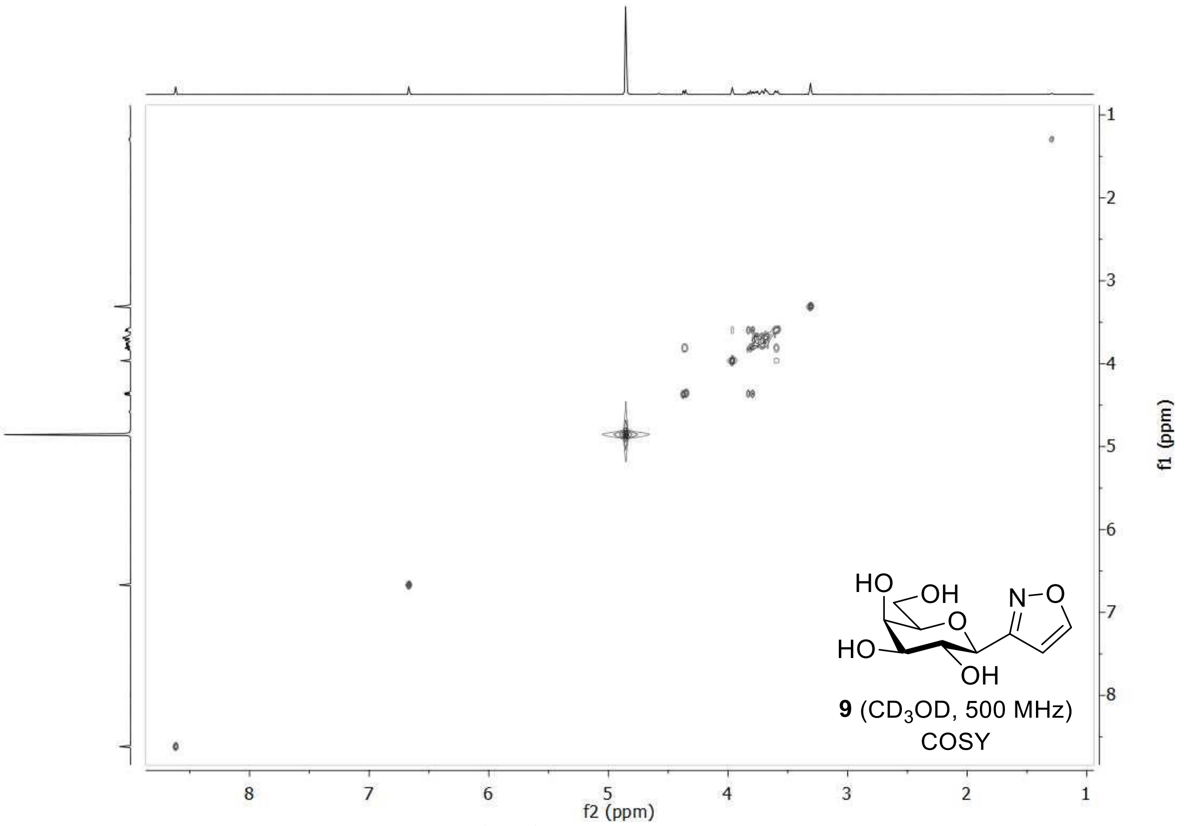

Figure S106. ^{1}H–^{1}H COSY spectrum of **9**

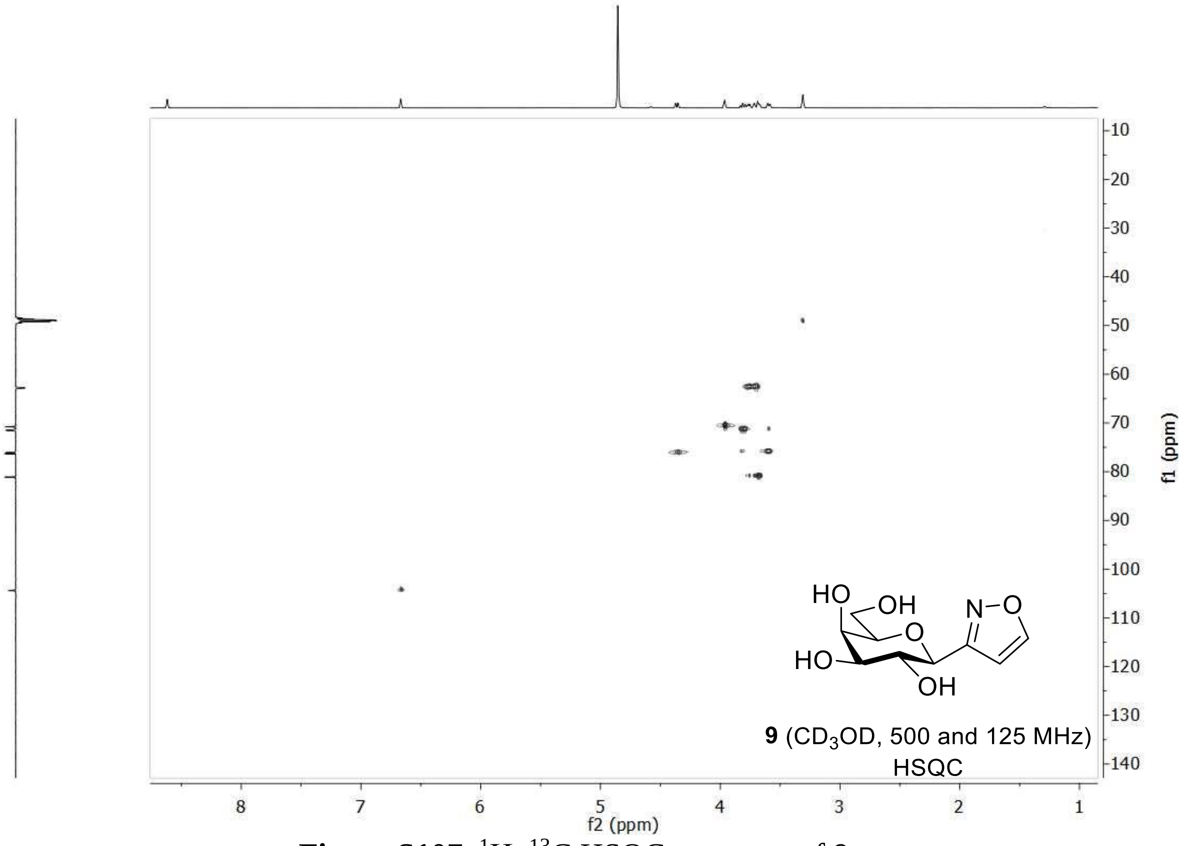

Figure S107. ^{1}H–^{13}C HSQC spectrum of **9**

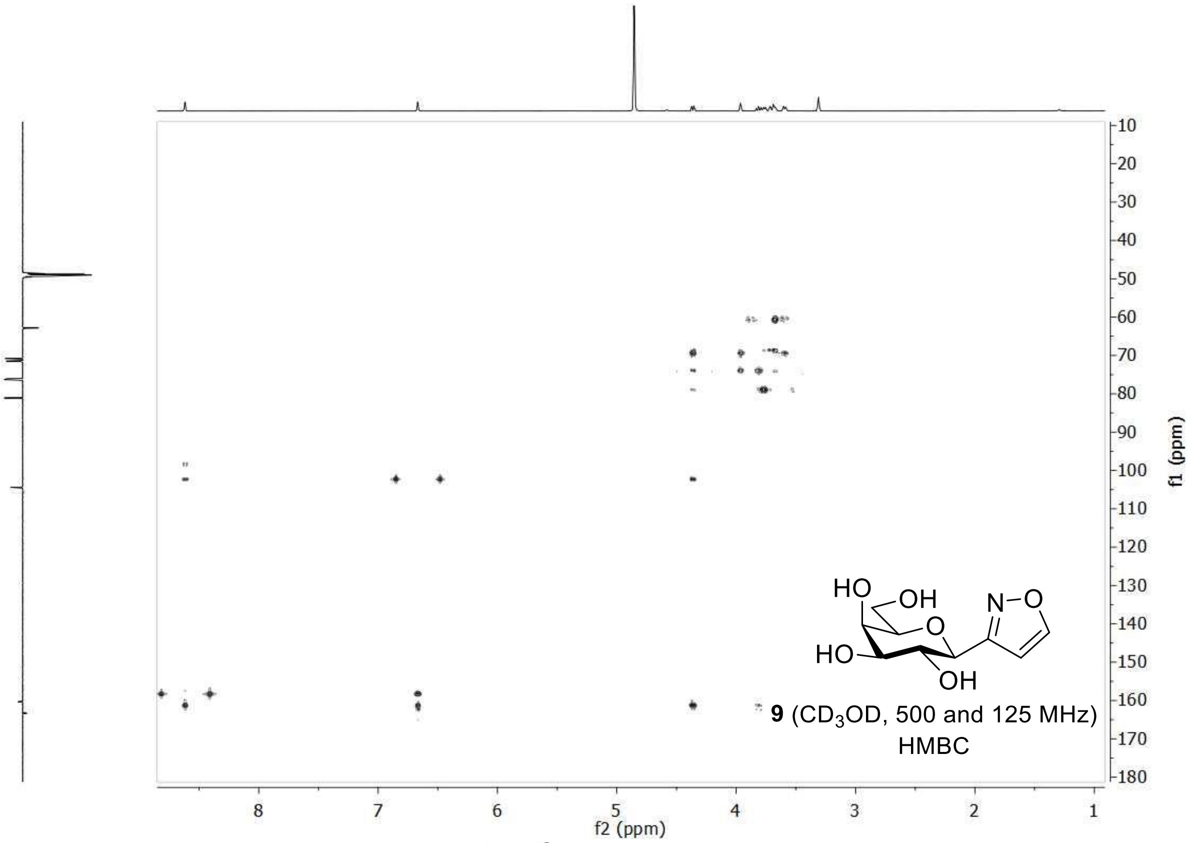

Figure S108. ^{1}H–^{13}C HMBC spectrum of **9**

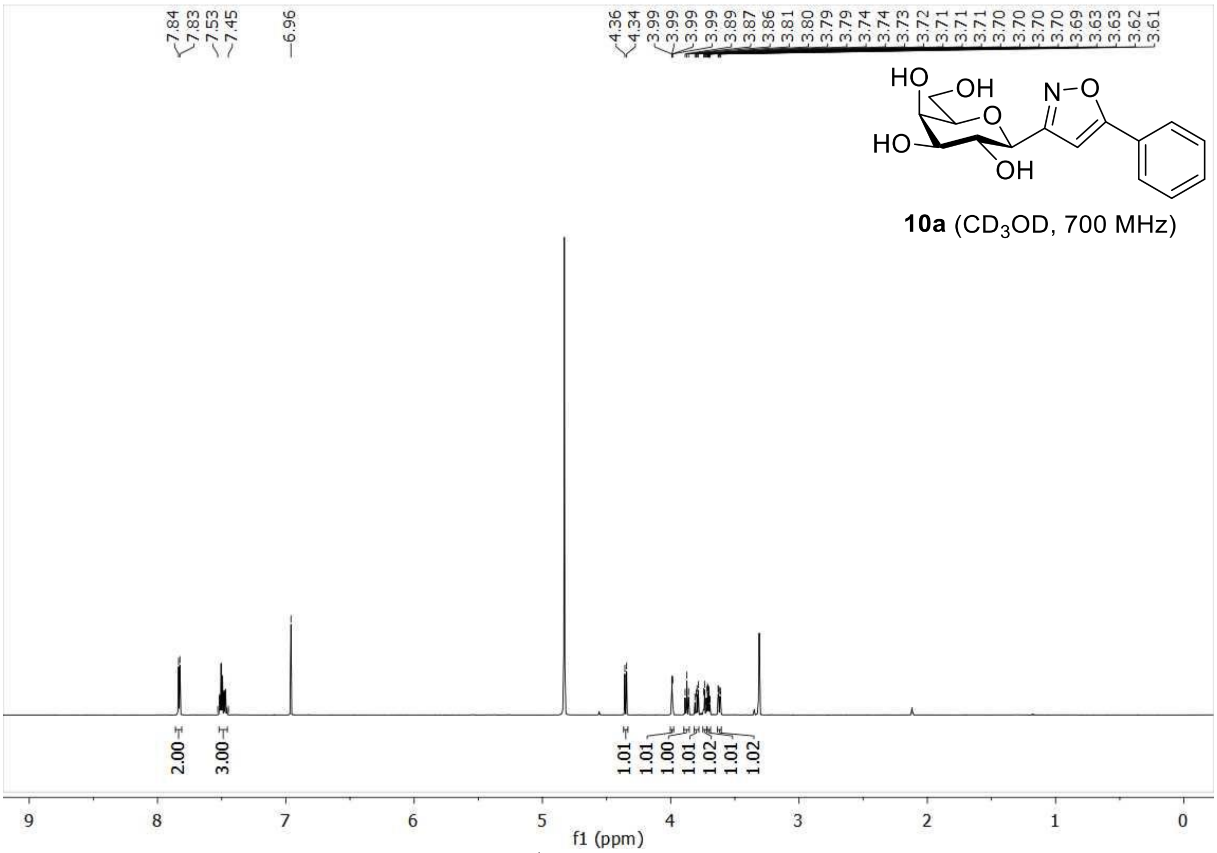

Figure S109. ^{1}H NMR spectrum of **10a**

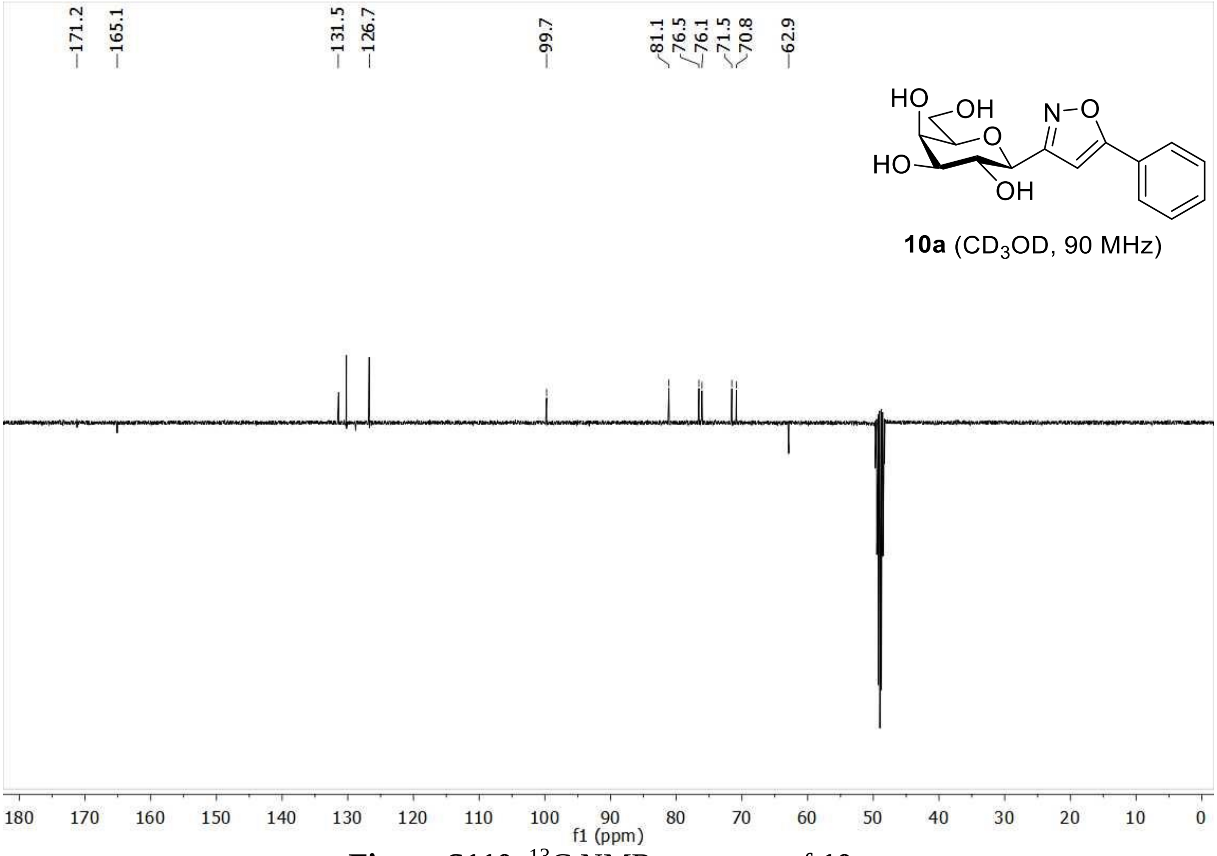

Figure S110. ^{13}C NMR spectrum of **10a**

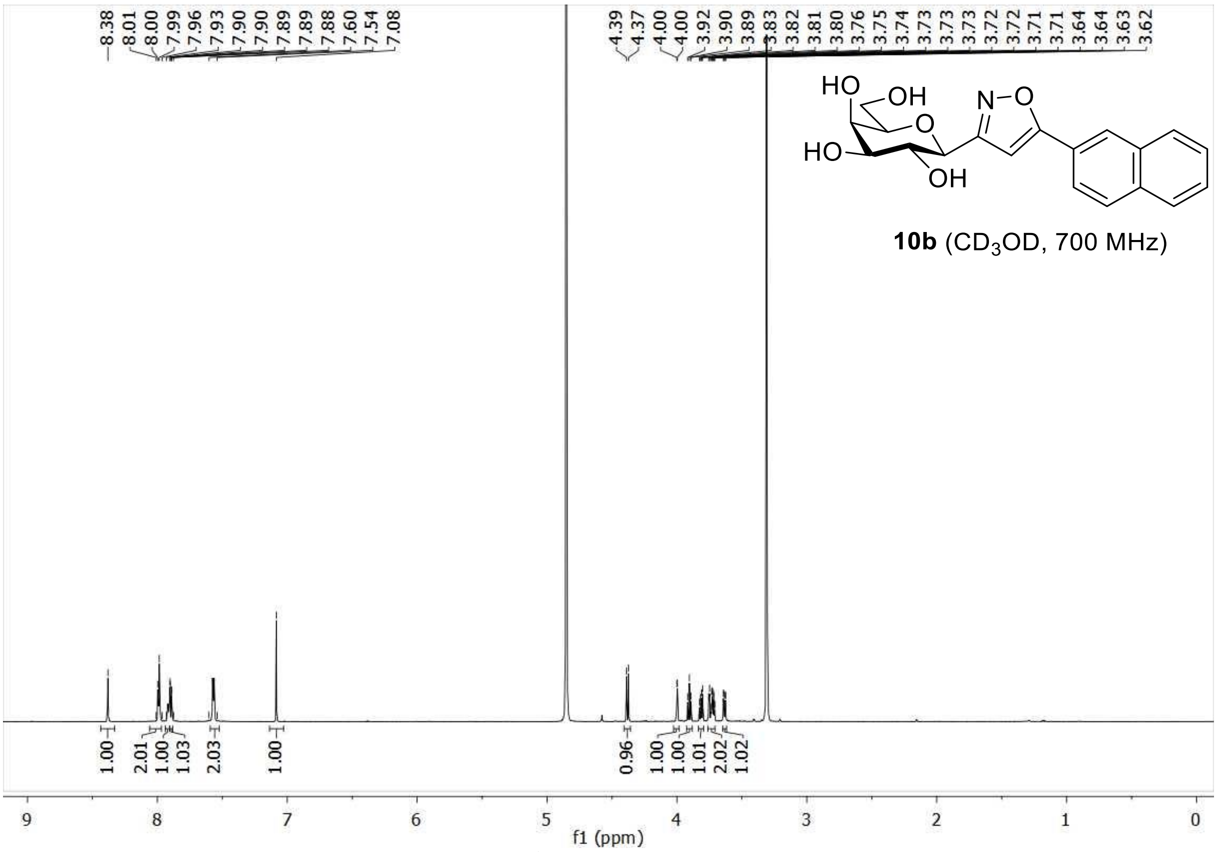

Figure S111. ^{1}H NMR spectrum of **10b**

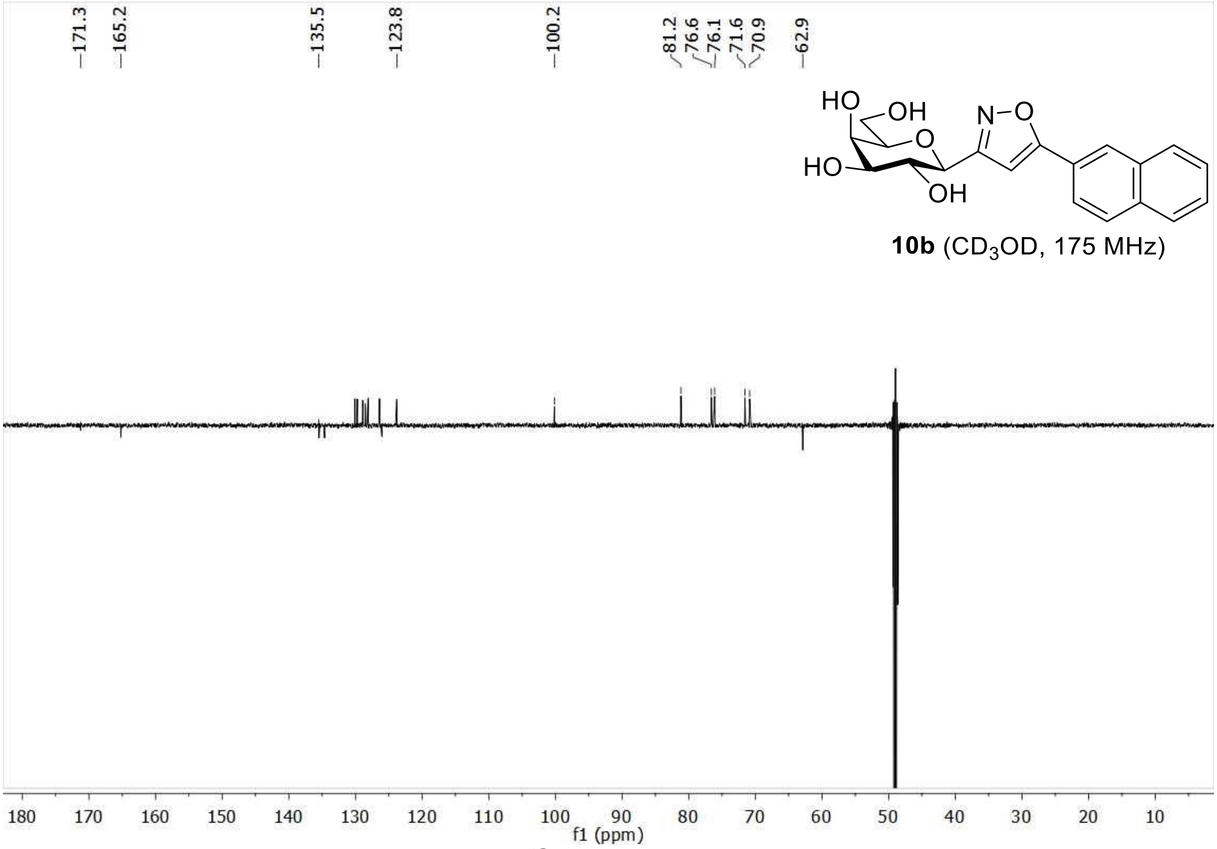

Figure S112. ^{13}C NMR spectrum of **10b**

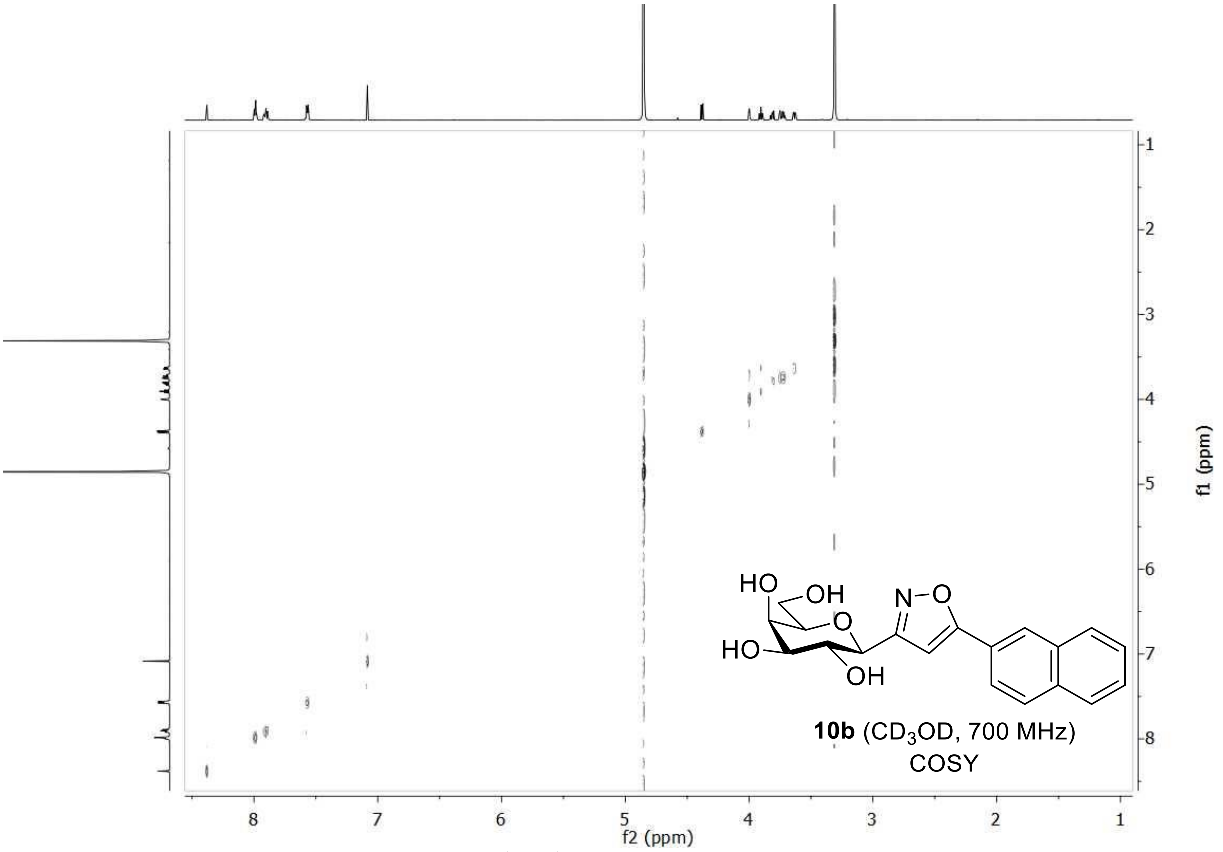

Figure S113. ^{1}H–^{1}H COSY spectrum of **10b**

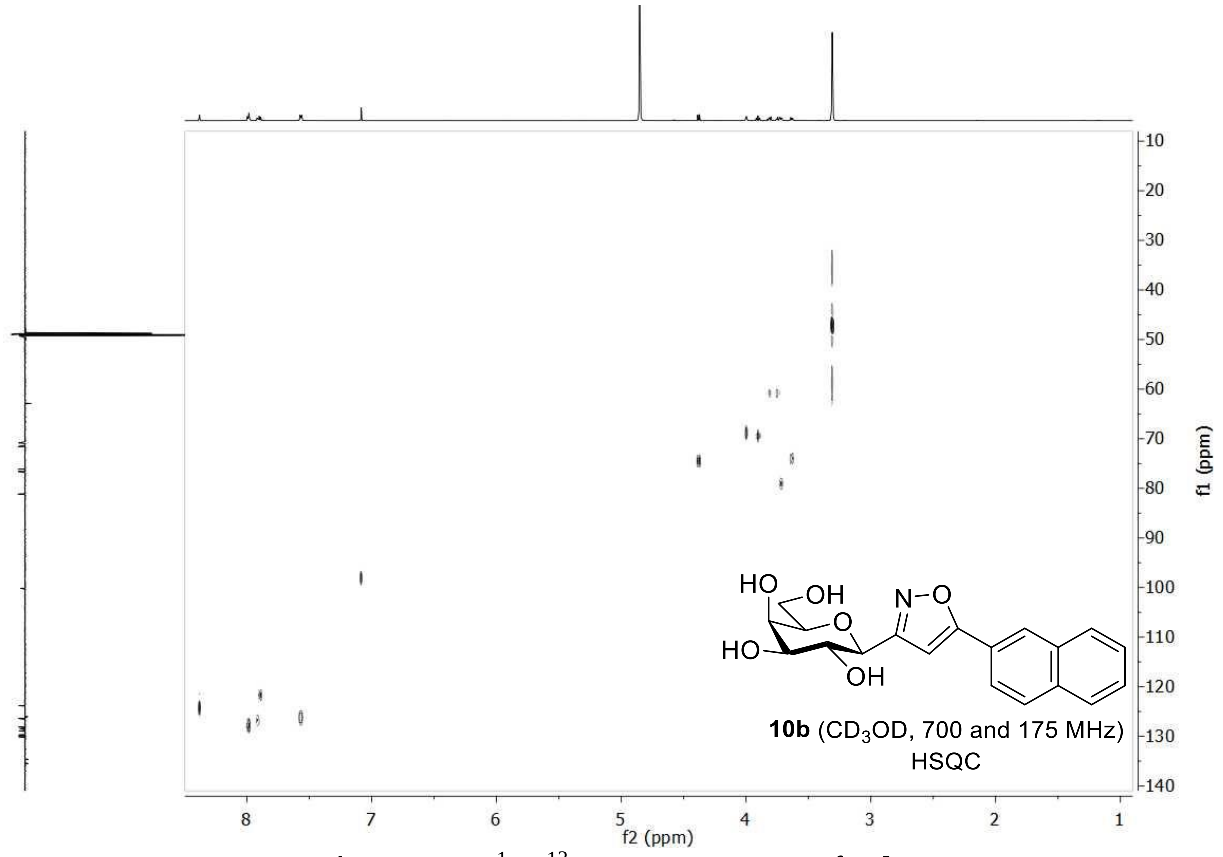

Figure S114. ^{1}H–^{13}C HSQC spectrum of **10b**

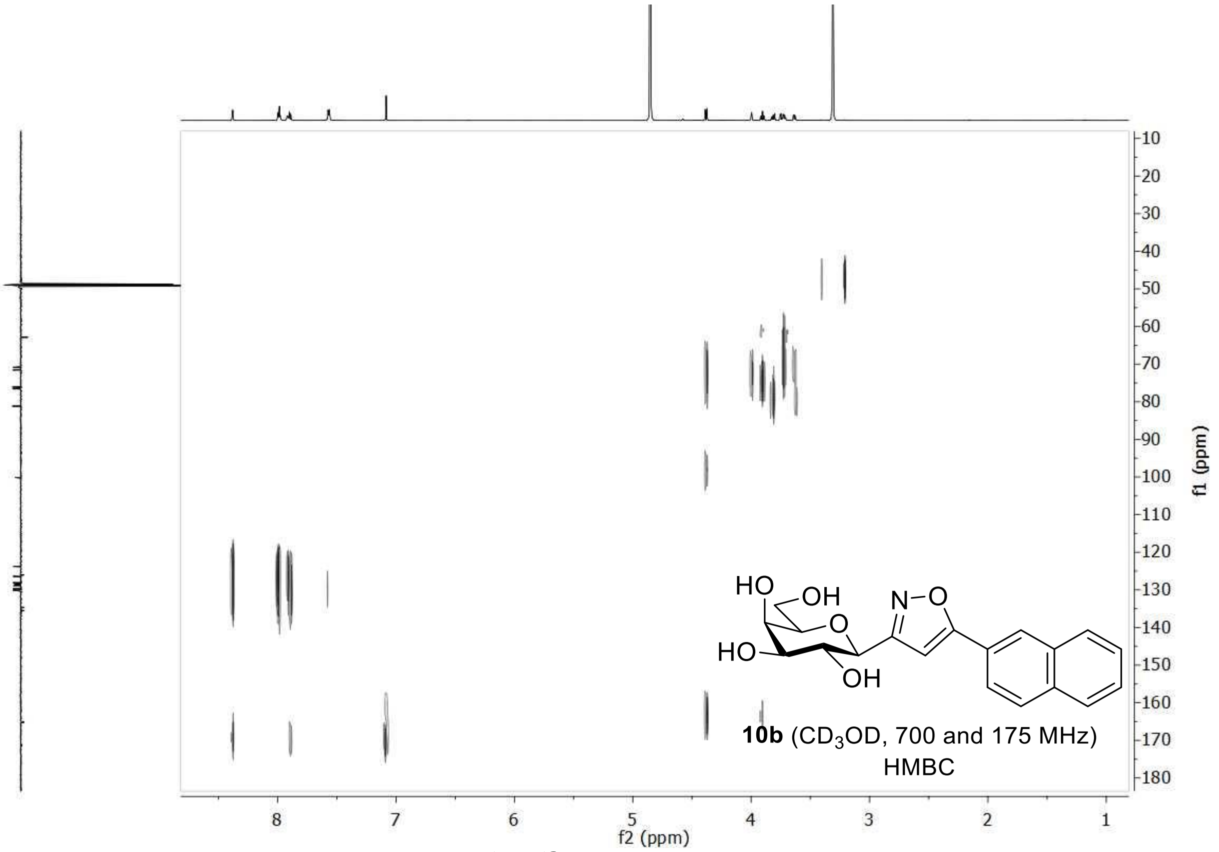

Figure S115. ^{1}H–^{13}C HMBC spectrum of **10b**

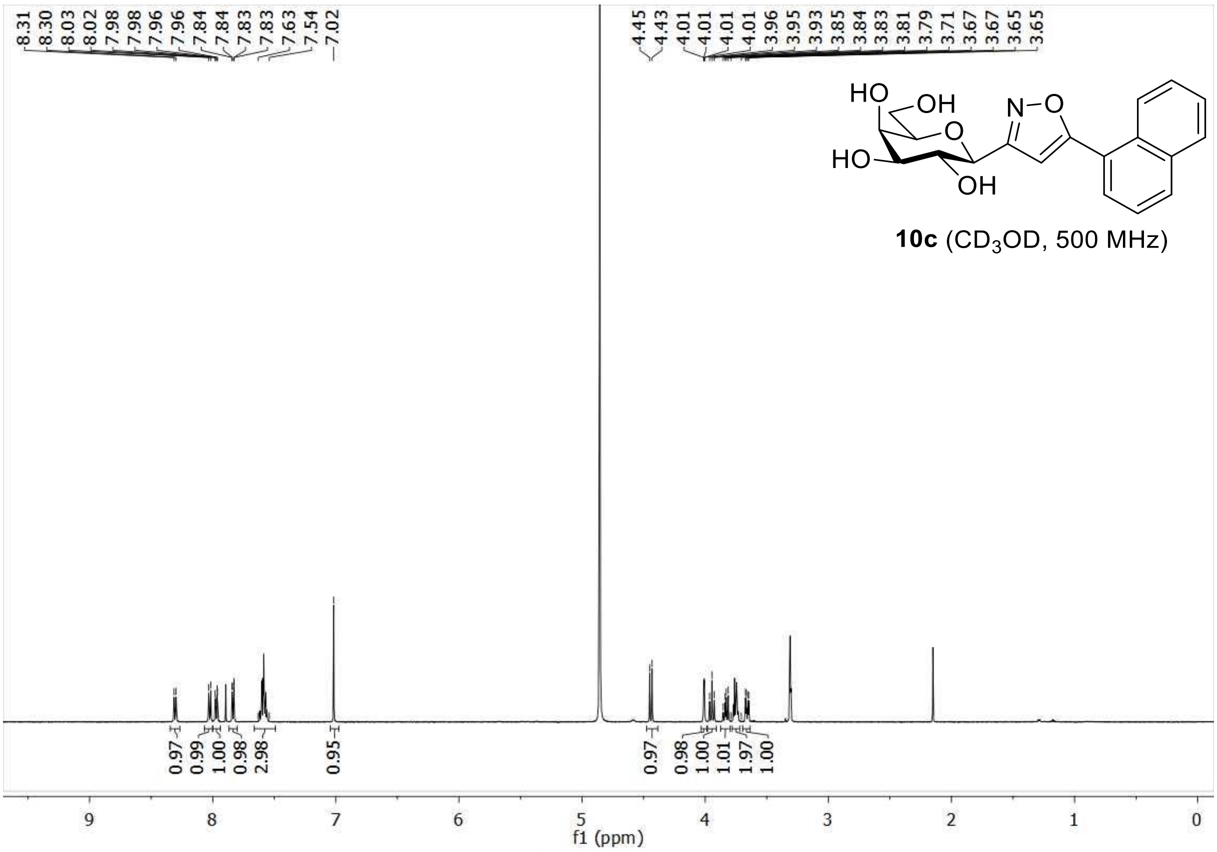

Figure S116. ^{1}H NMR spectrum of **10c**

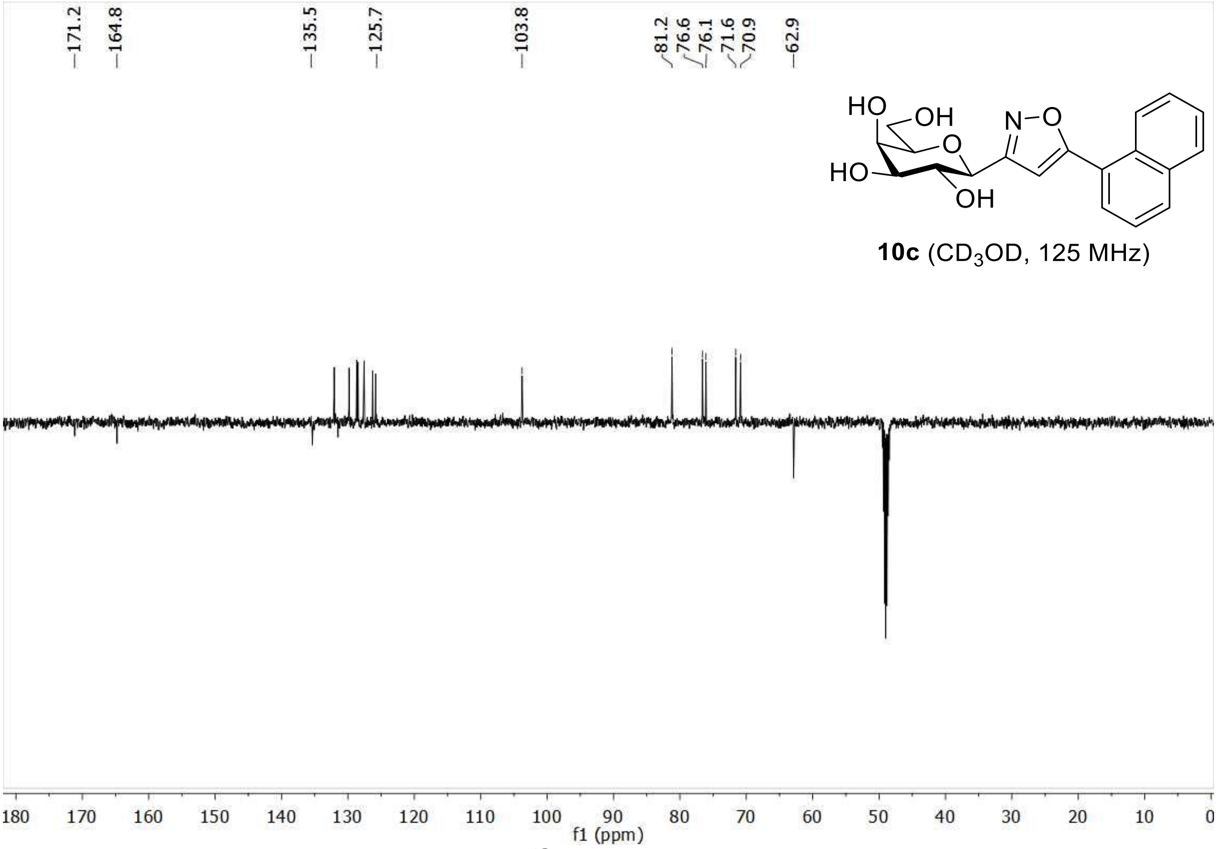

Figure S117. ^{13}C NMR spectrum of **10c**

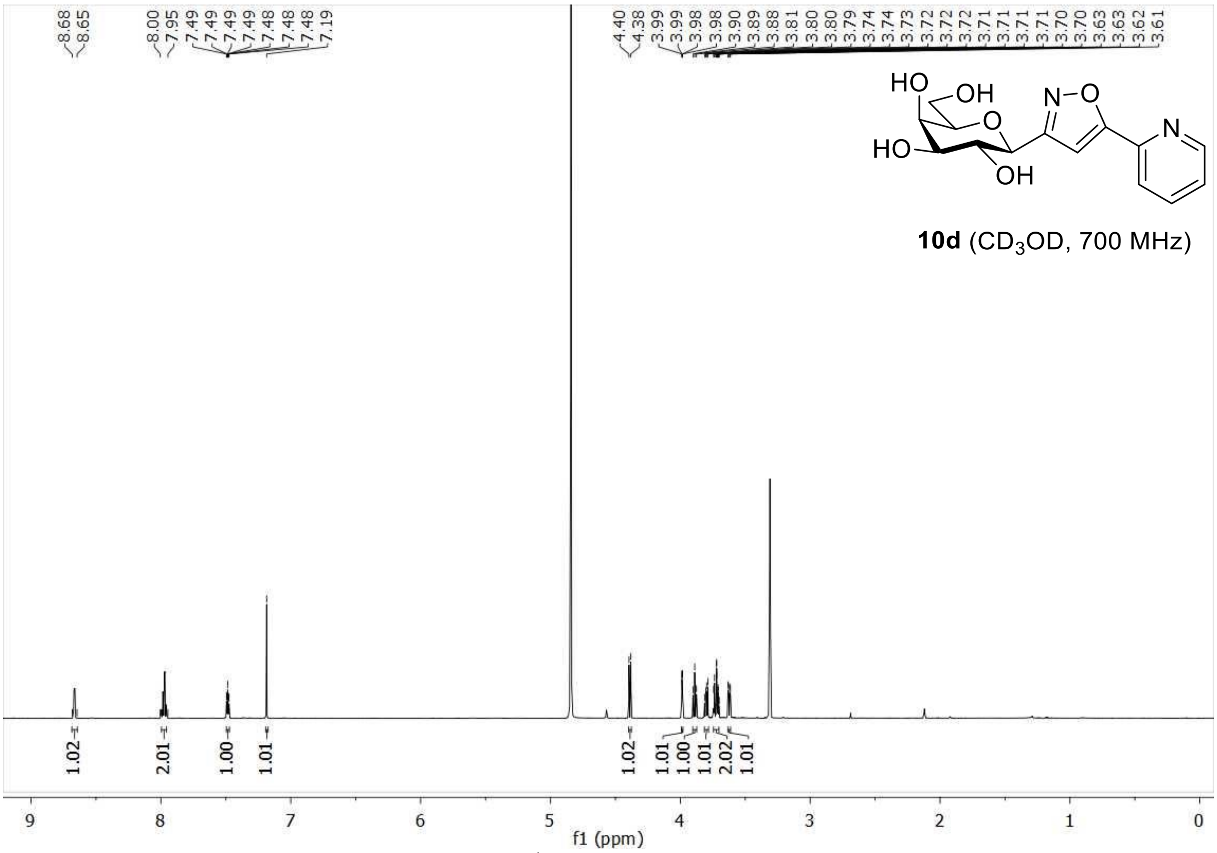

Figure S118. ^{1}H NMR spectrum of **10d**

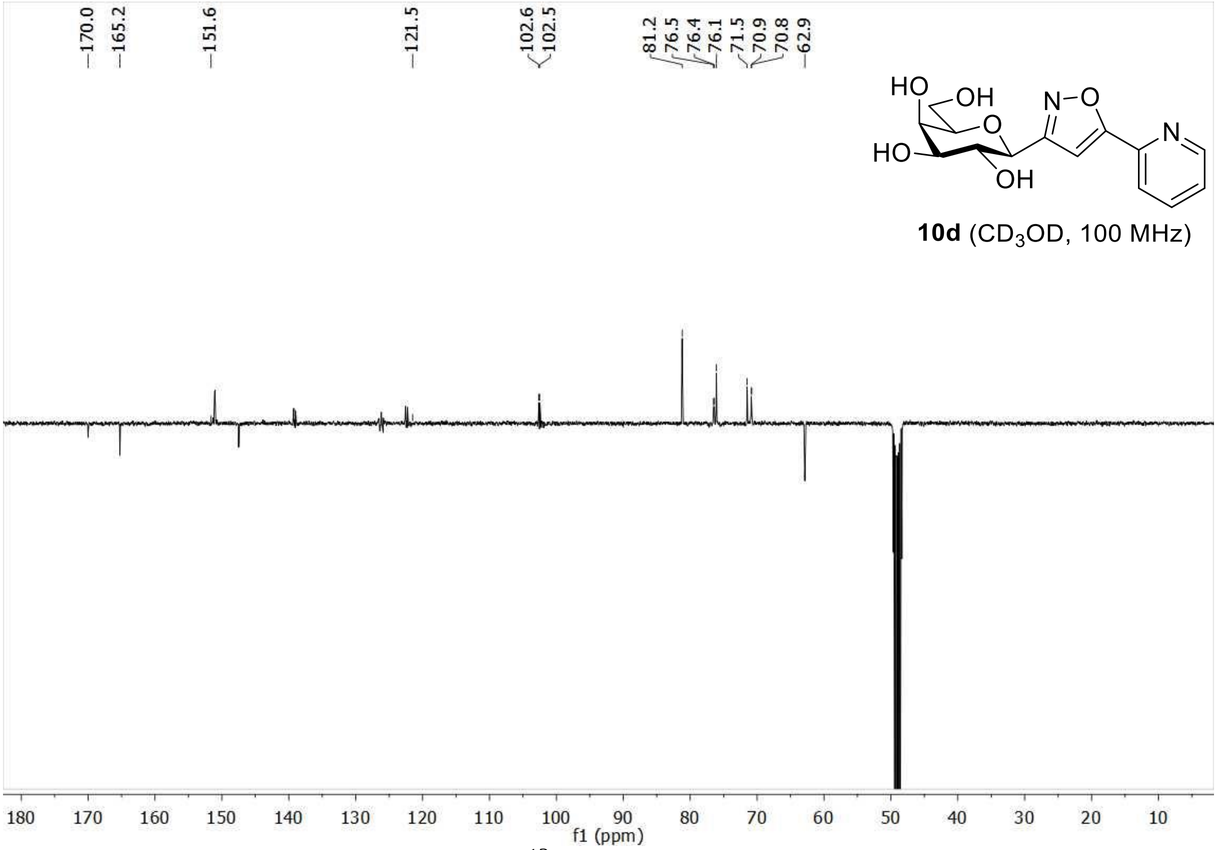

Figure S119. ^{13}C NMR spectrum of **10d**

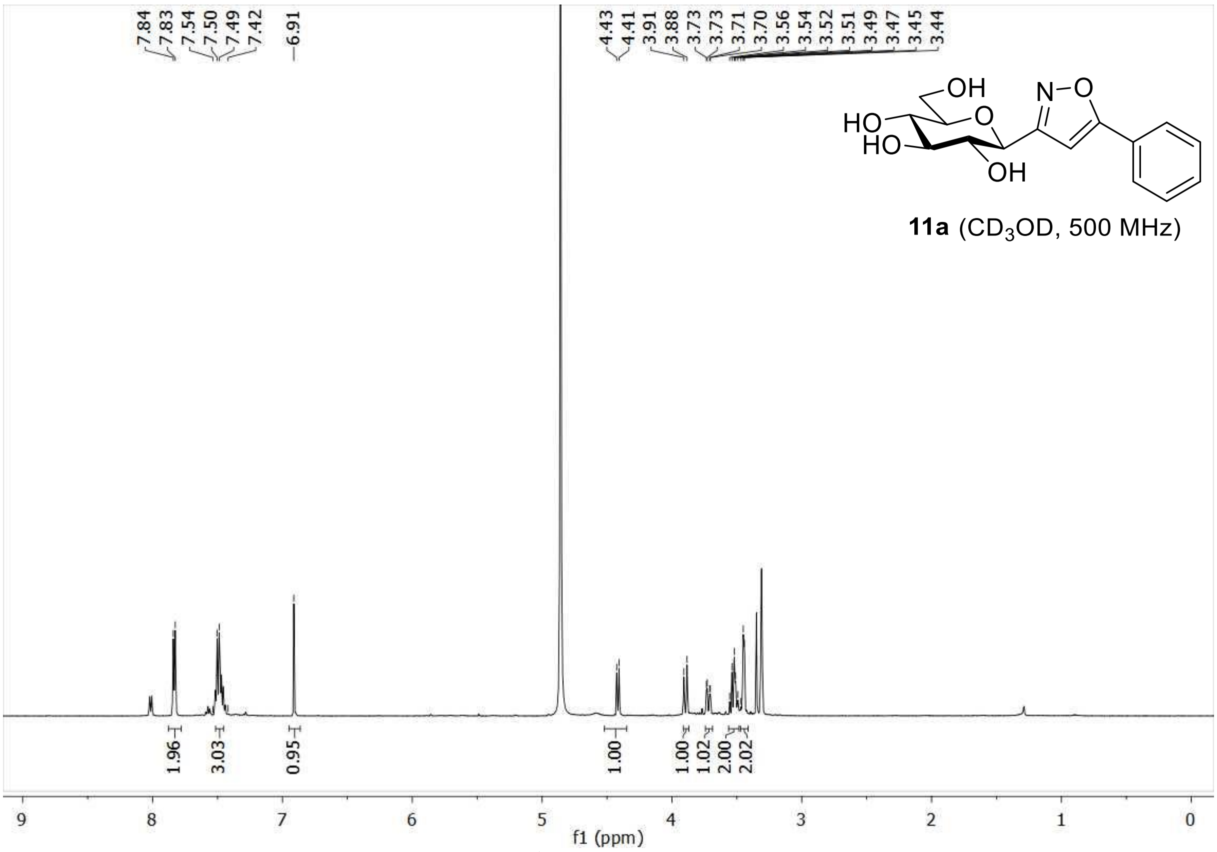

Figure S120. ^{1}H NMR spectrum of **11a**

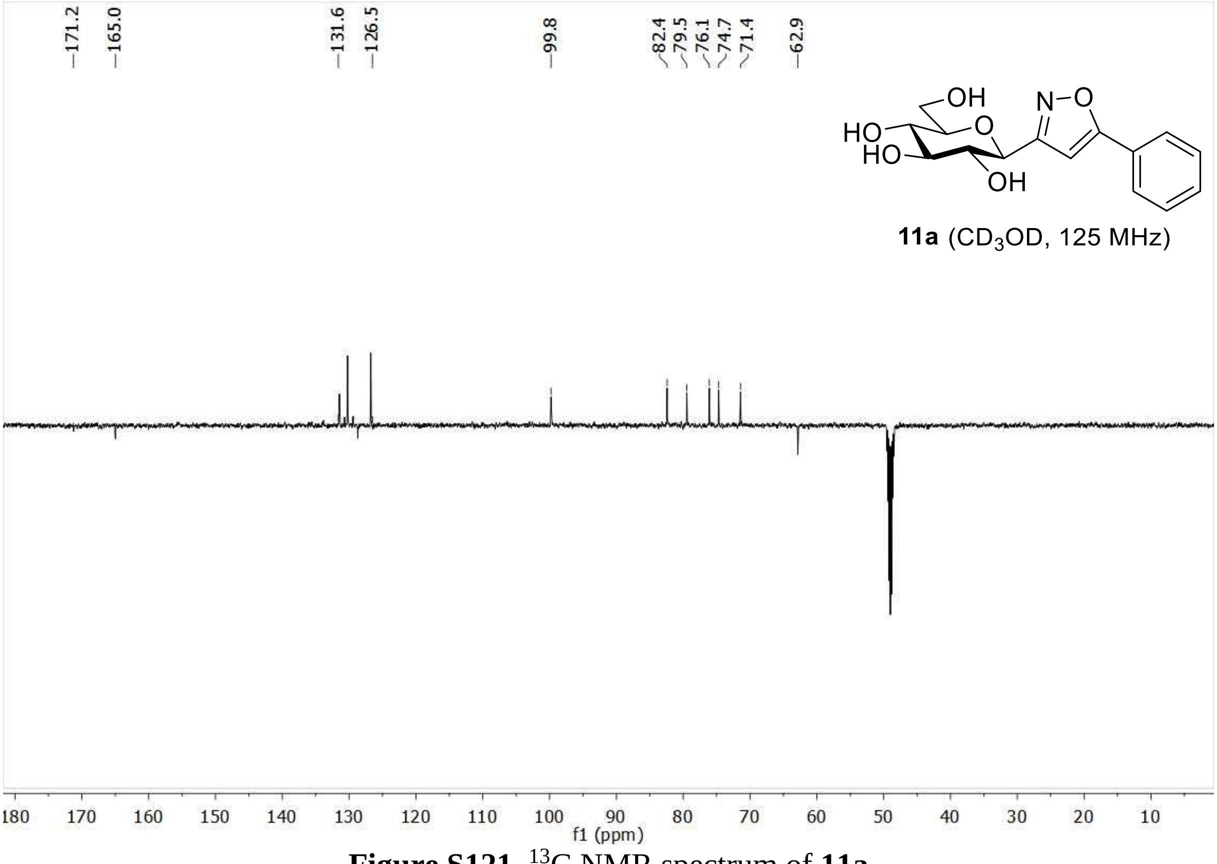

Figure S121. ^{13}C NMR spectrum of **11a**

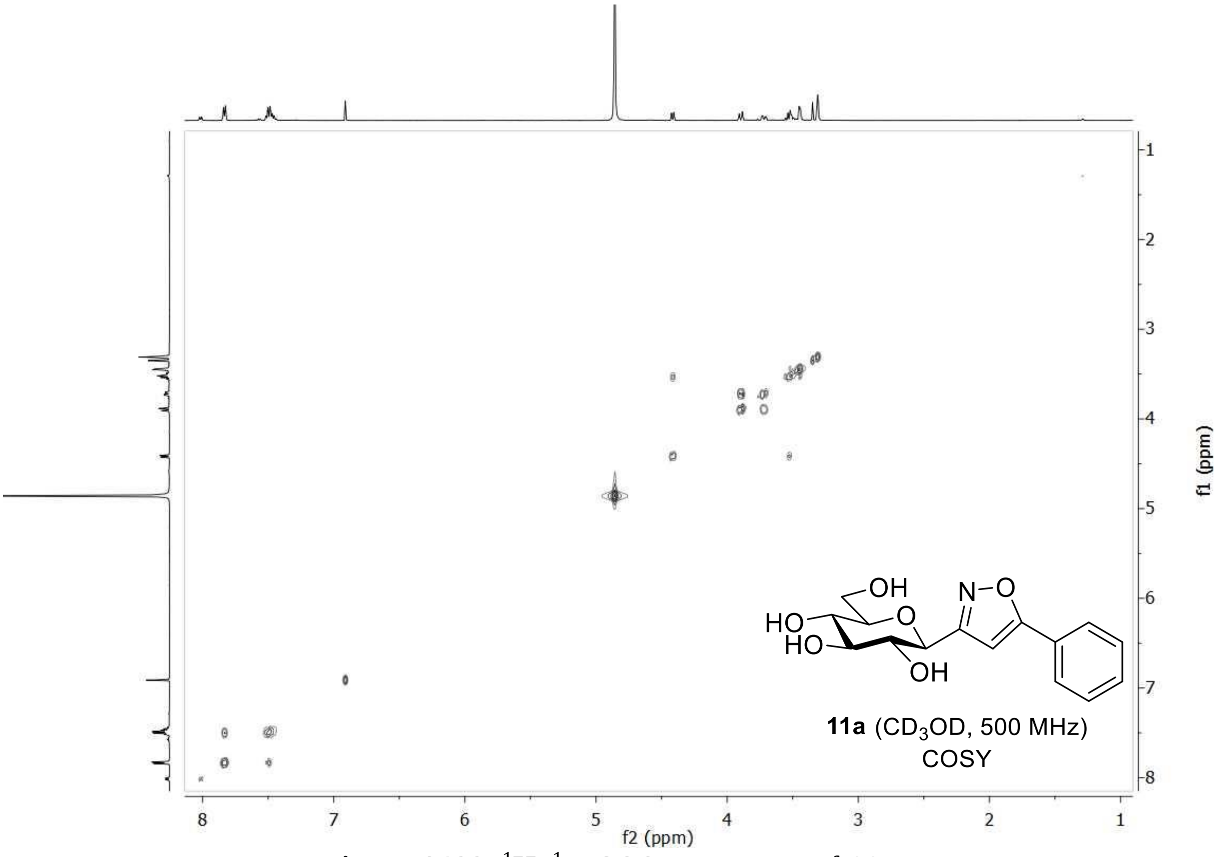

Figure S122. ^{1}H–^{1}H COSY spectrum of **11a**

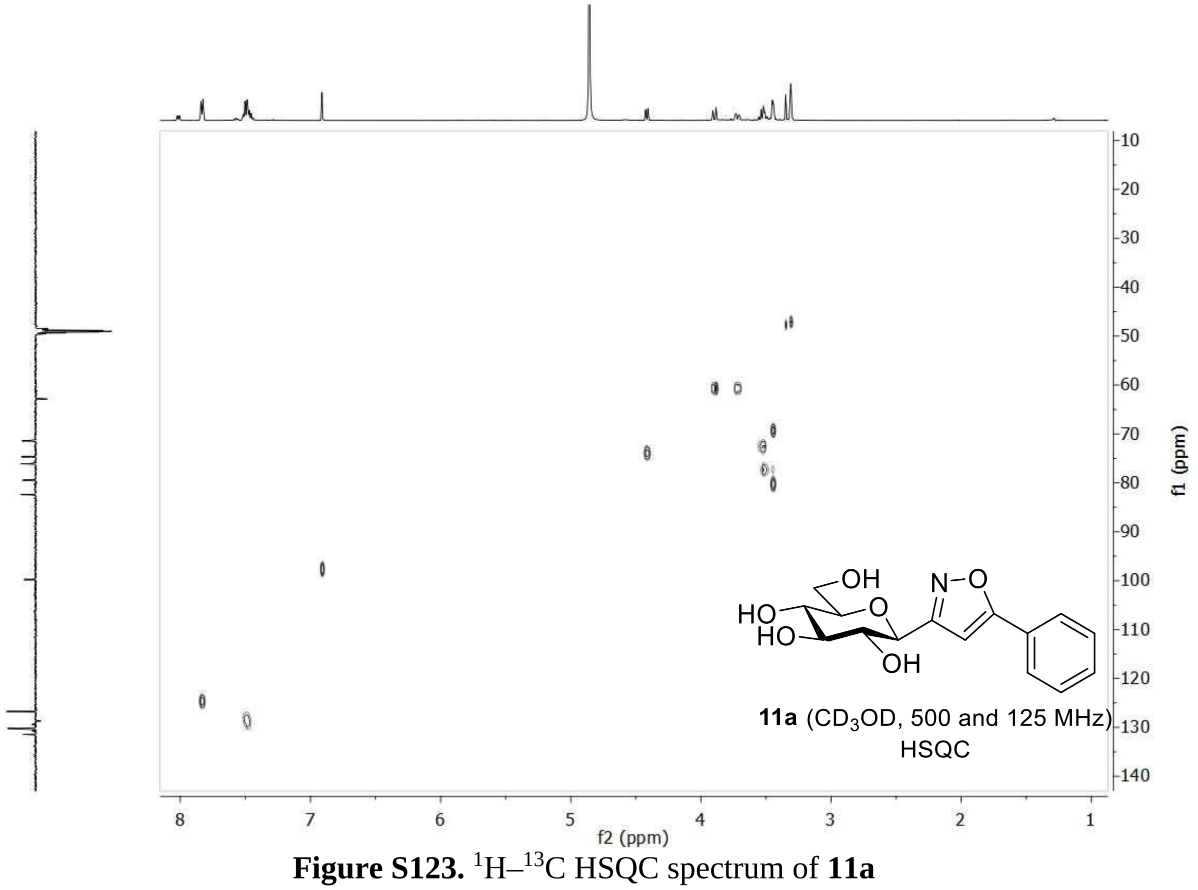

Figure S123. ^{1}H–^{13}C HSQC spectrum of **11a**

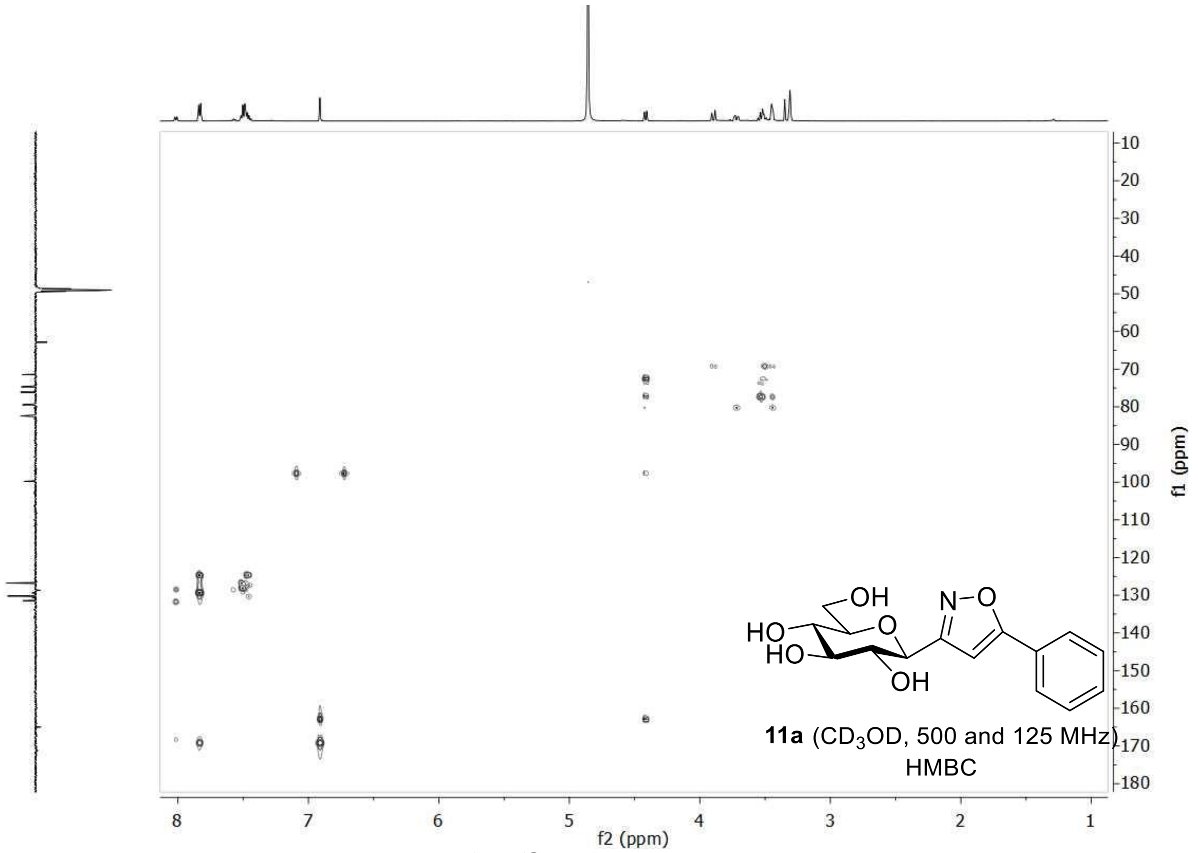

Figure S124. ^{1}H–^{13}C HMBC spectrum of **11a**

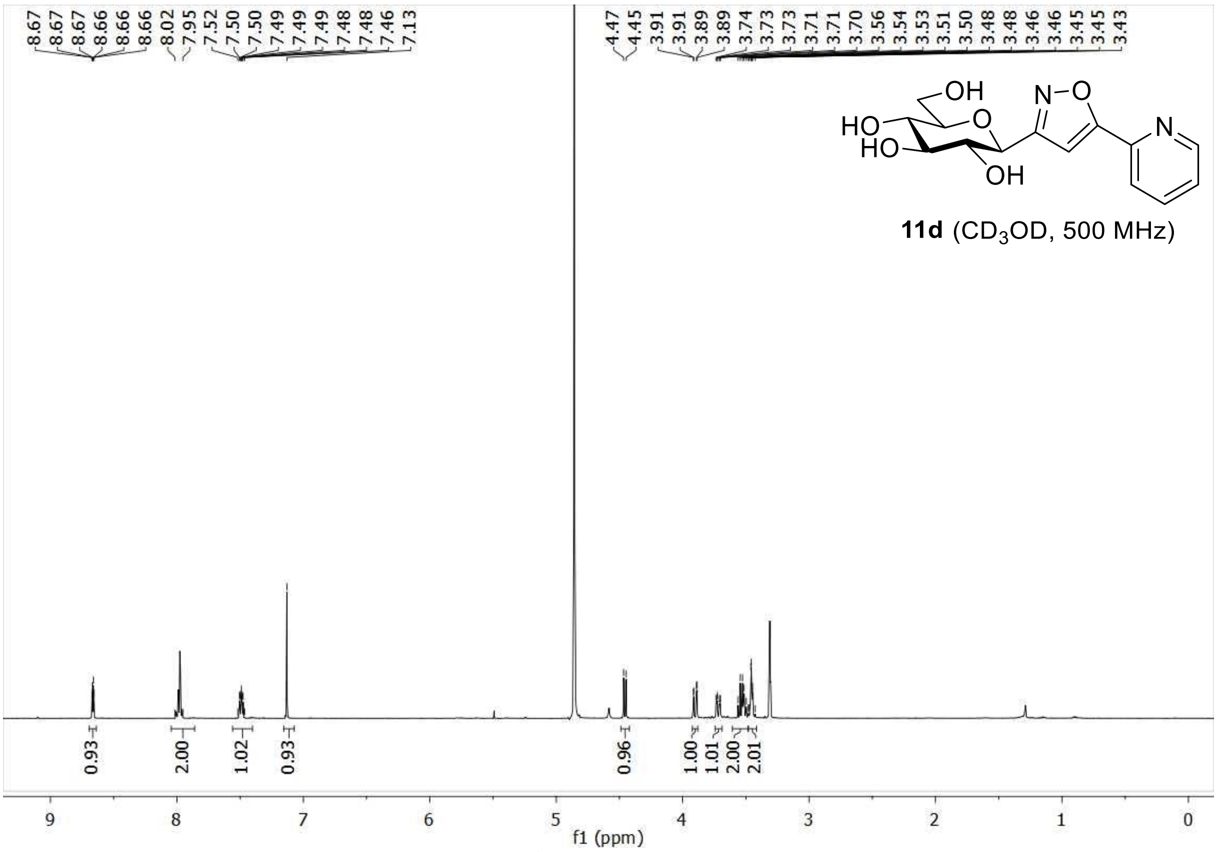

Figure S125. ^{1}H NMR spectrum of **11d**

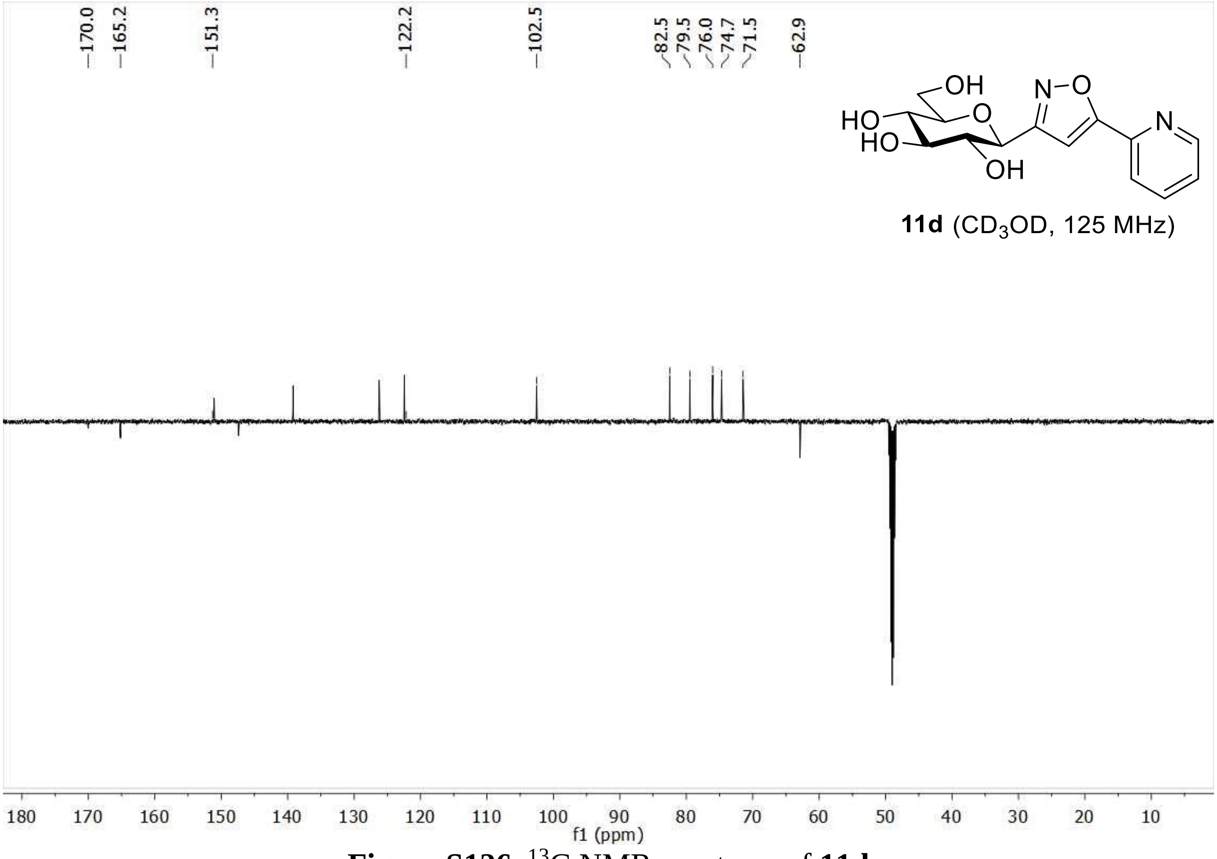

Figure S126. ^{13}C NMR spectrum of **11d**

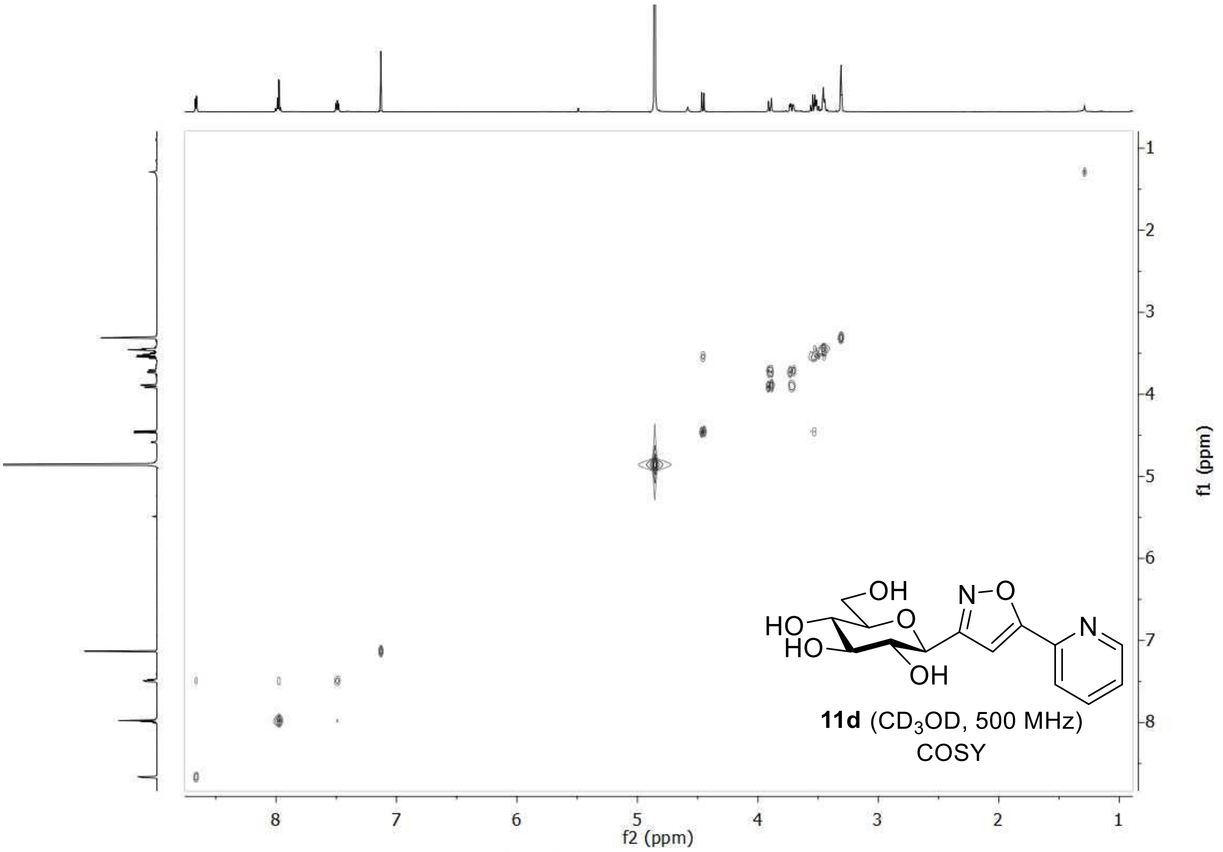

Figure S127. ^{1}H–^{1}H COSY spectrum of **11d**

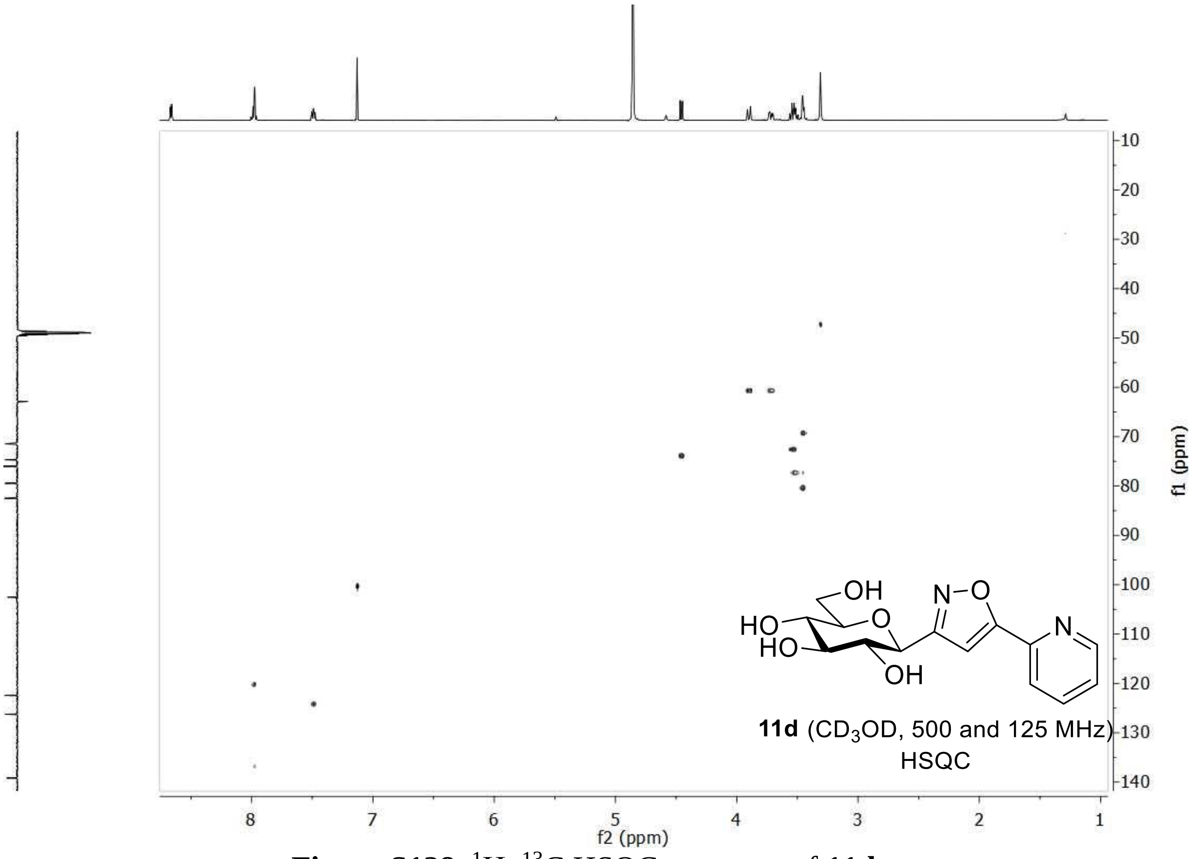

Figure S128. ^{1}H–^{13}C HSQC spectrum of **11d**

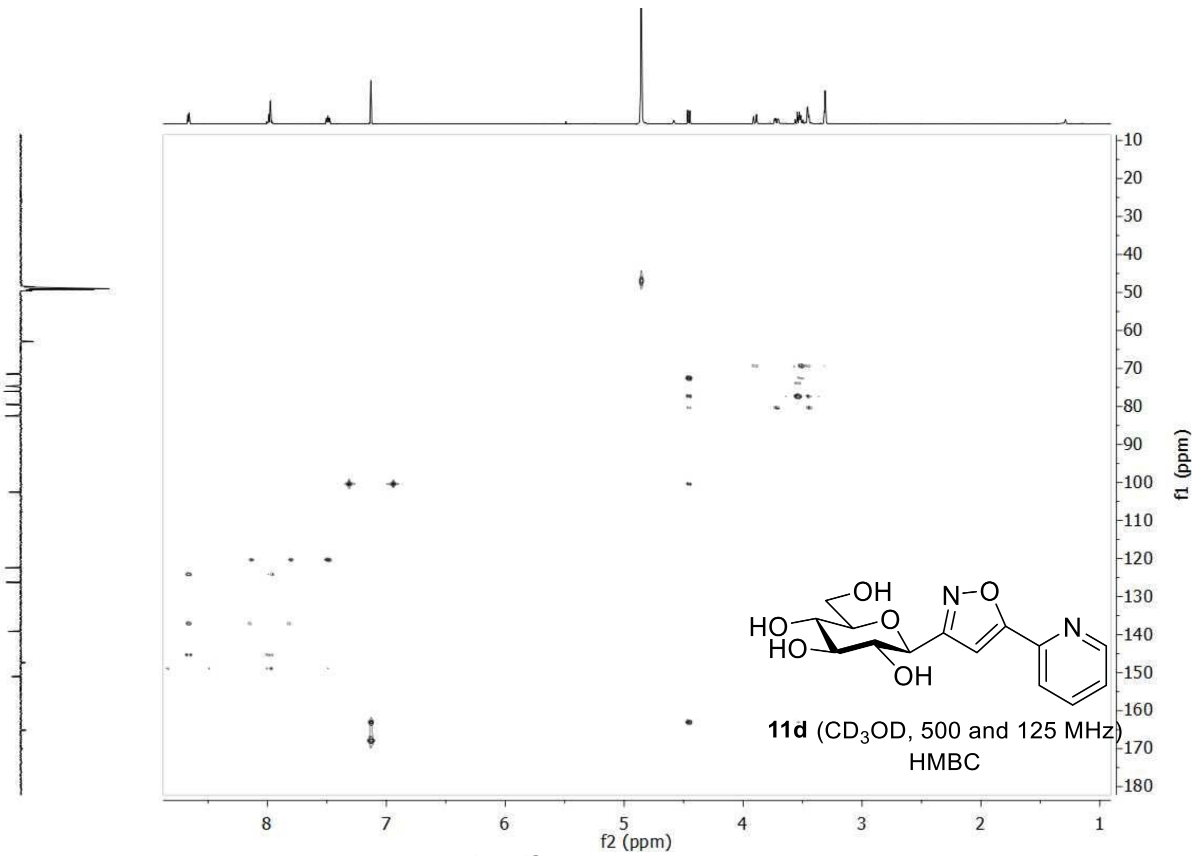

Figure S129. ^{1}H–^{13}C HMBC spectrum of **11d**

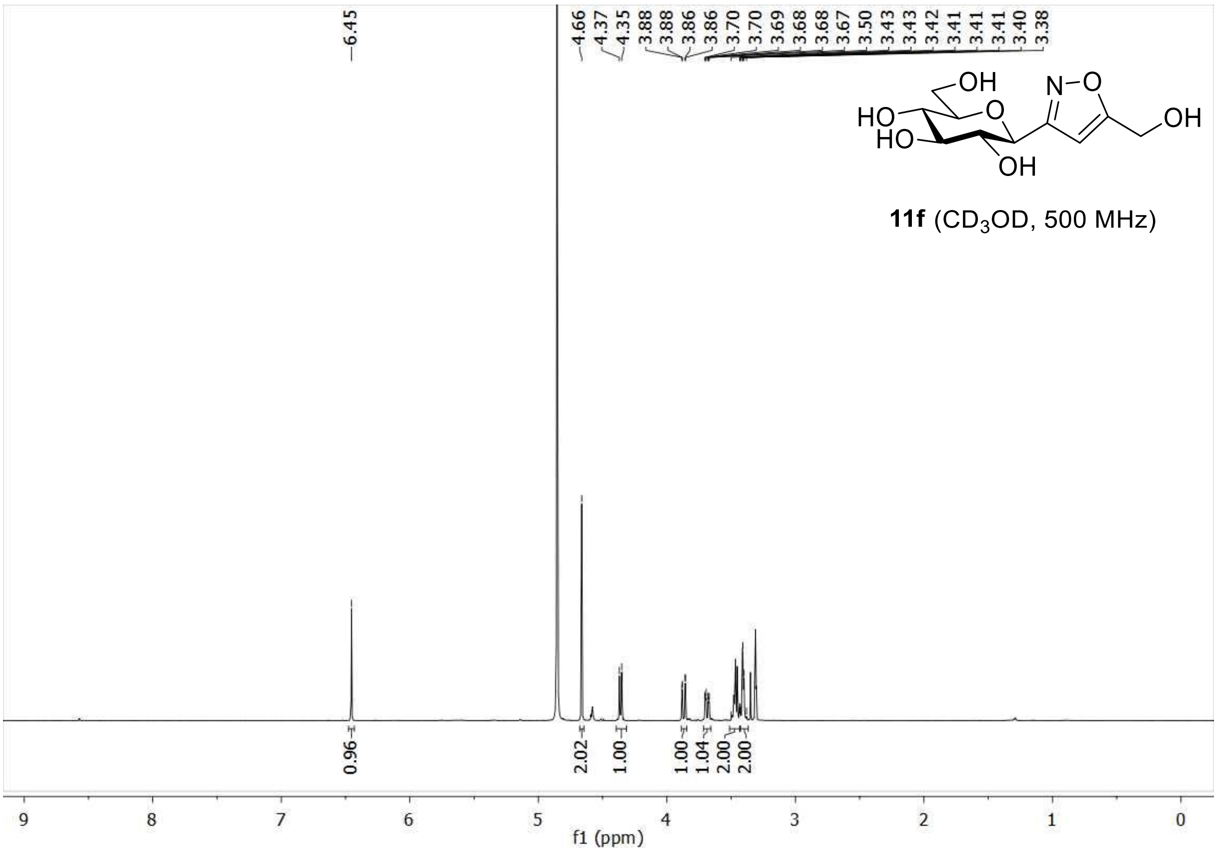

Figure S130. ^{1}H NMR spectrum of **11f**

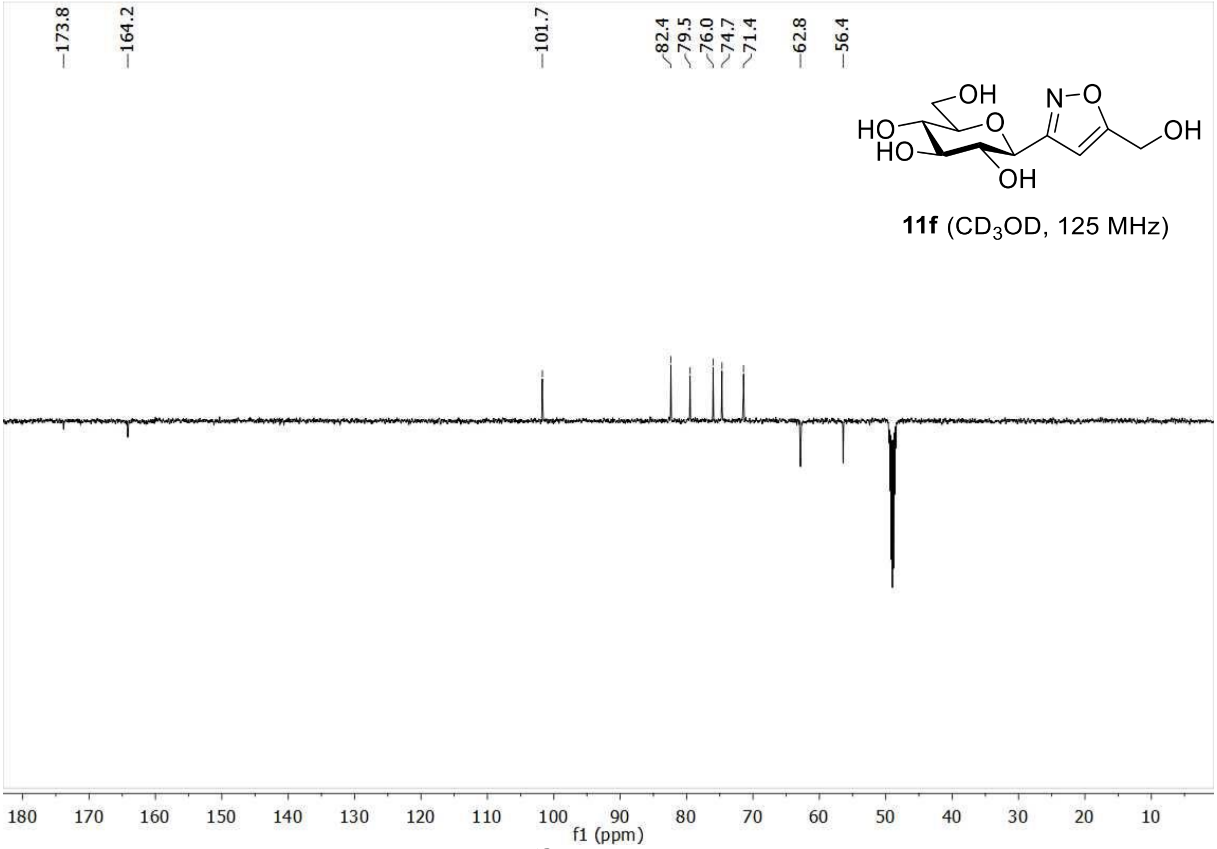

Figure S131. ^{13}C NMR spectrum of **11f**

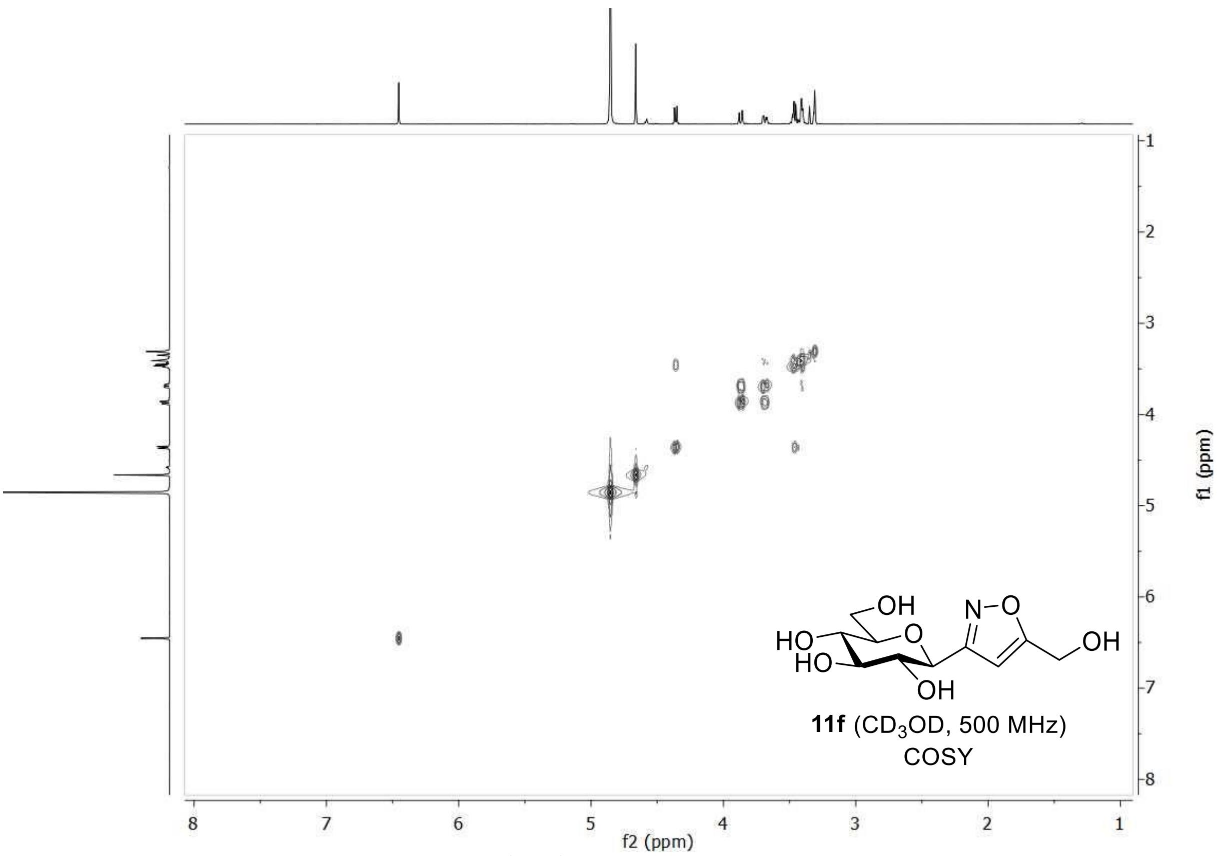

Figure S132. ^{1}H–^{1}H COSY spectrum of **11f**

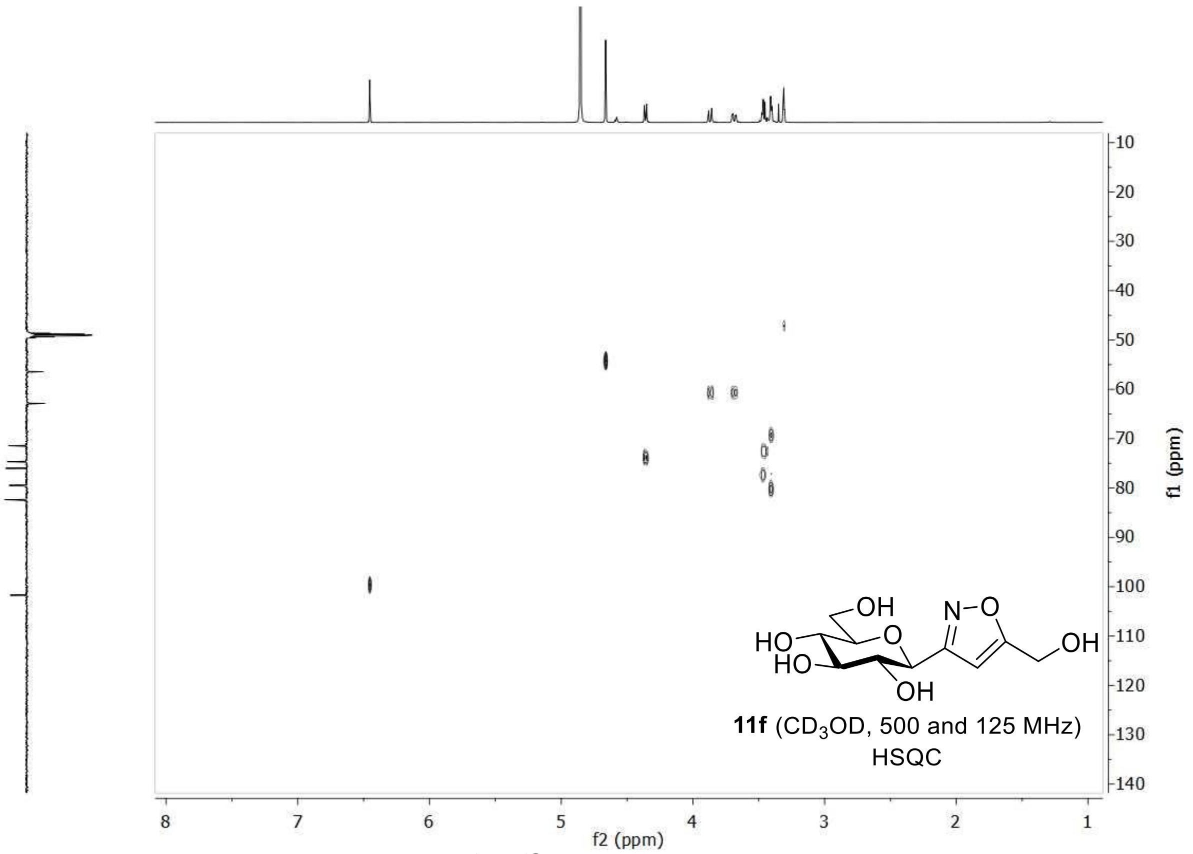

Figure S133. ^{1}H–^{13}C HSQC spectrum of **11f**

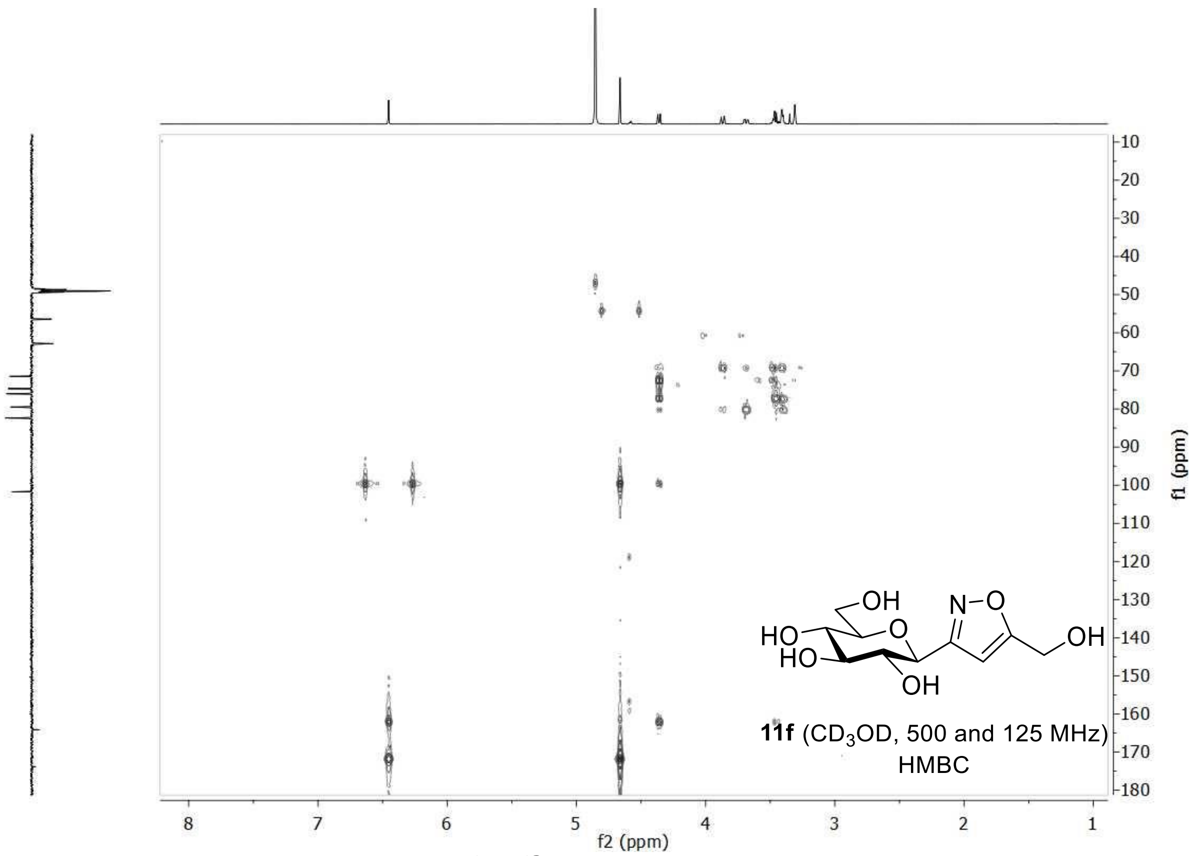

Figure S134. ^{1}H–^{13}C HMBC spectrum of **11f**

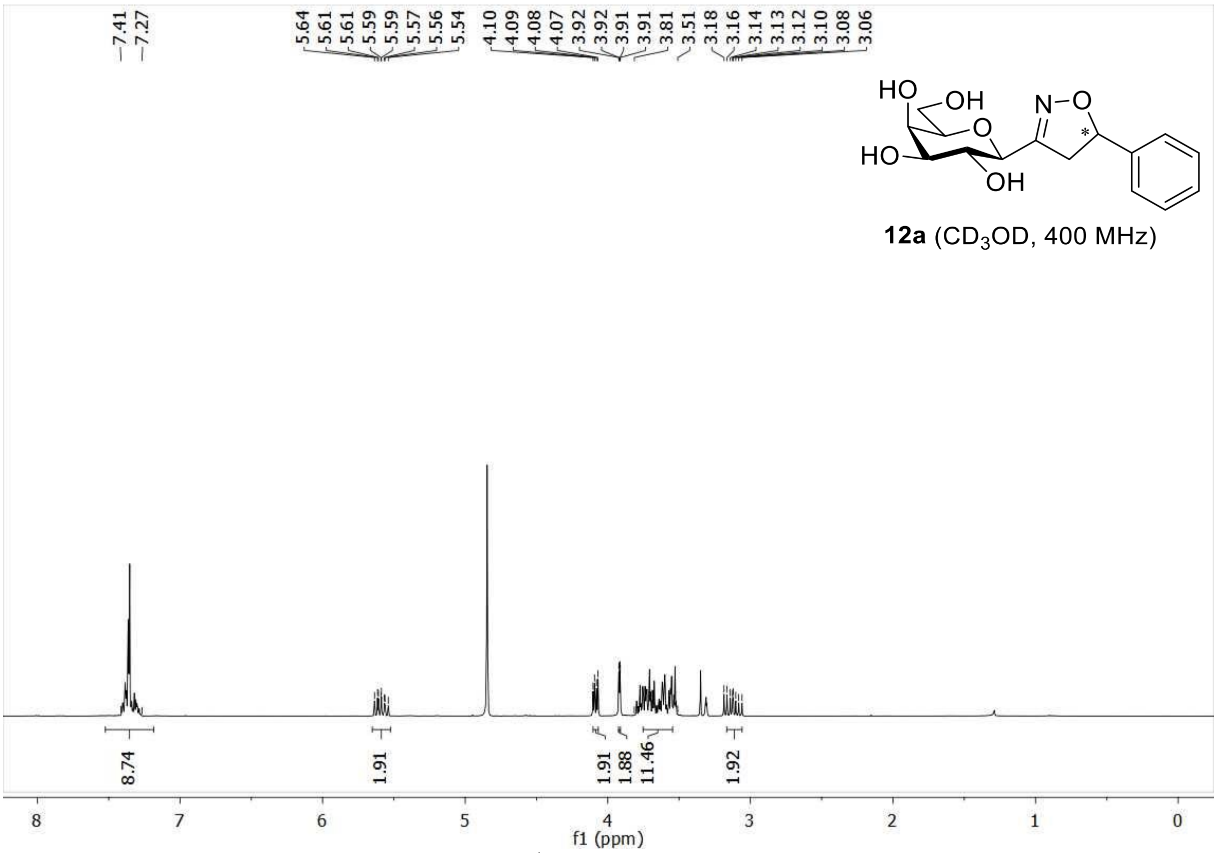

Figure S135. ^{1}H NMR spectrum of **12a**

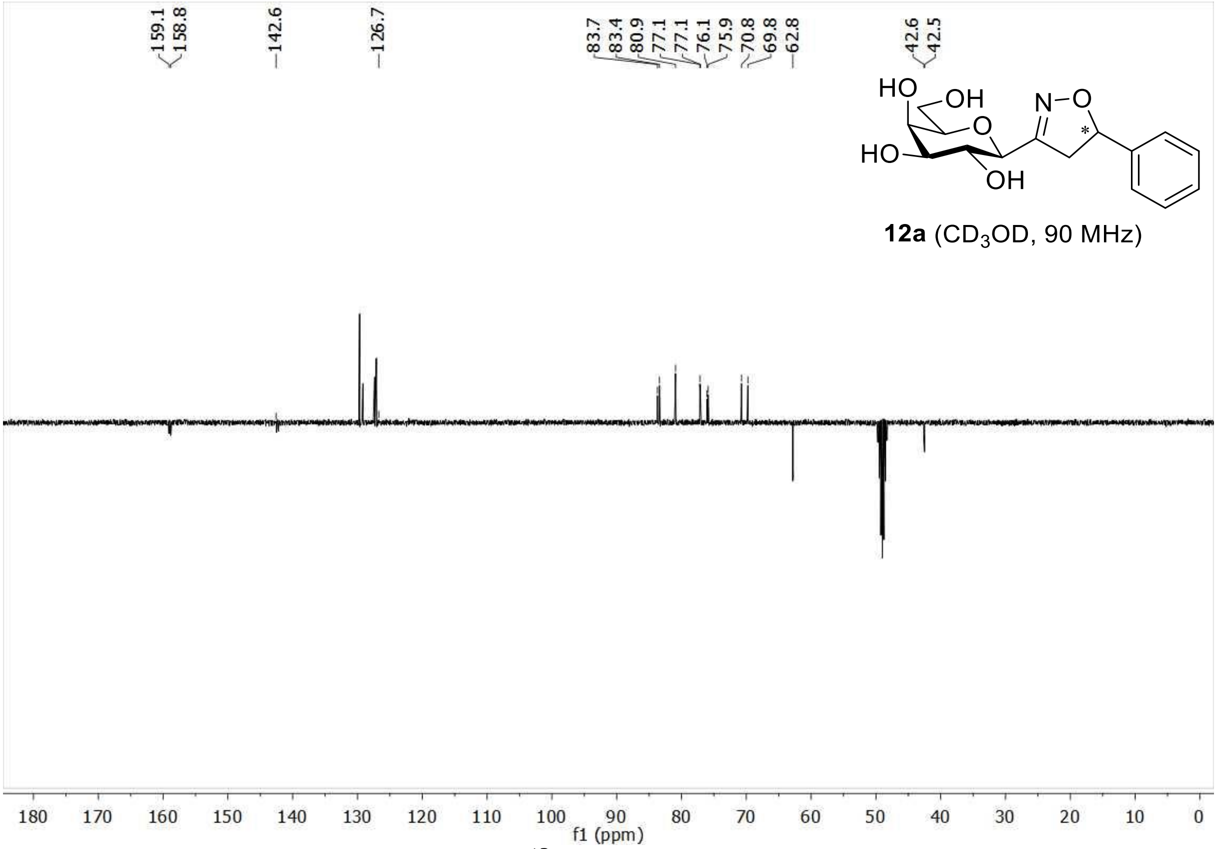

Figure S136. ^{13}C NMR spectrum of **12a**

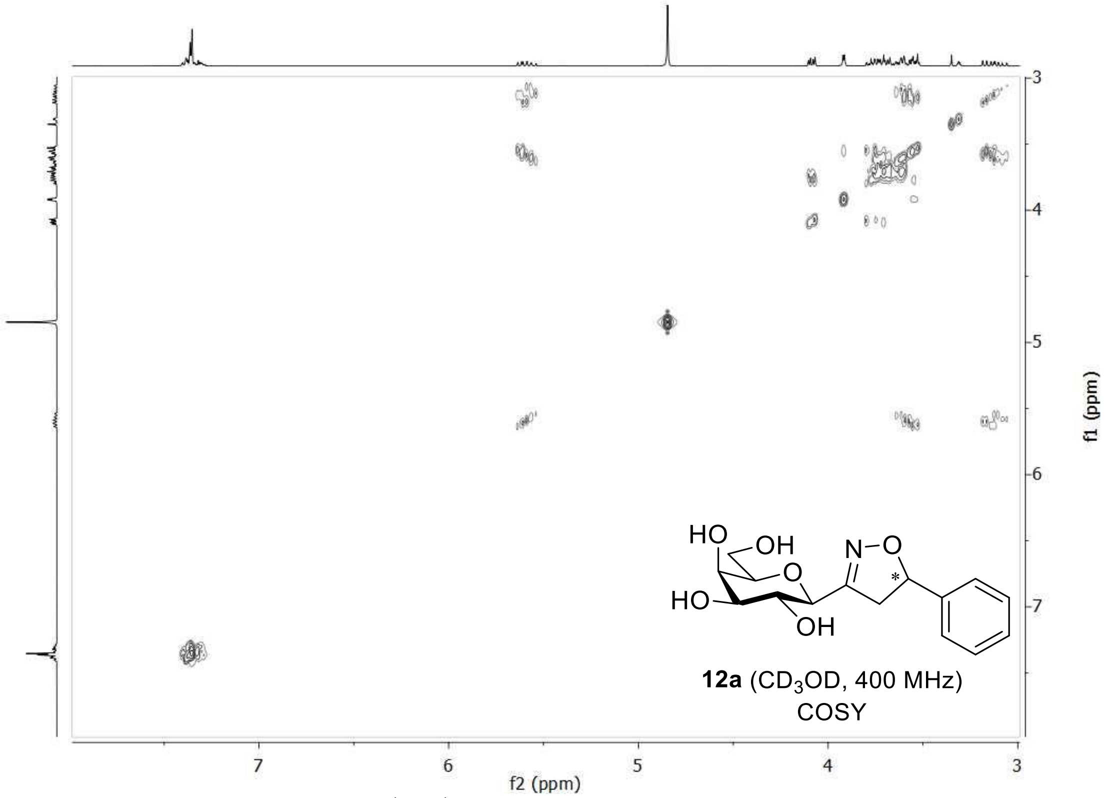

Figure S137. ^{1}H–^{1}H COSY spectrum of **12a**

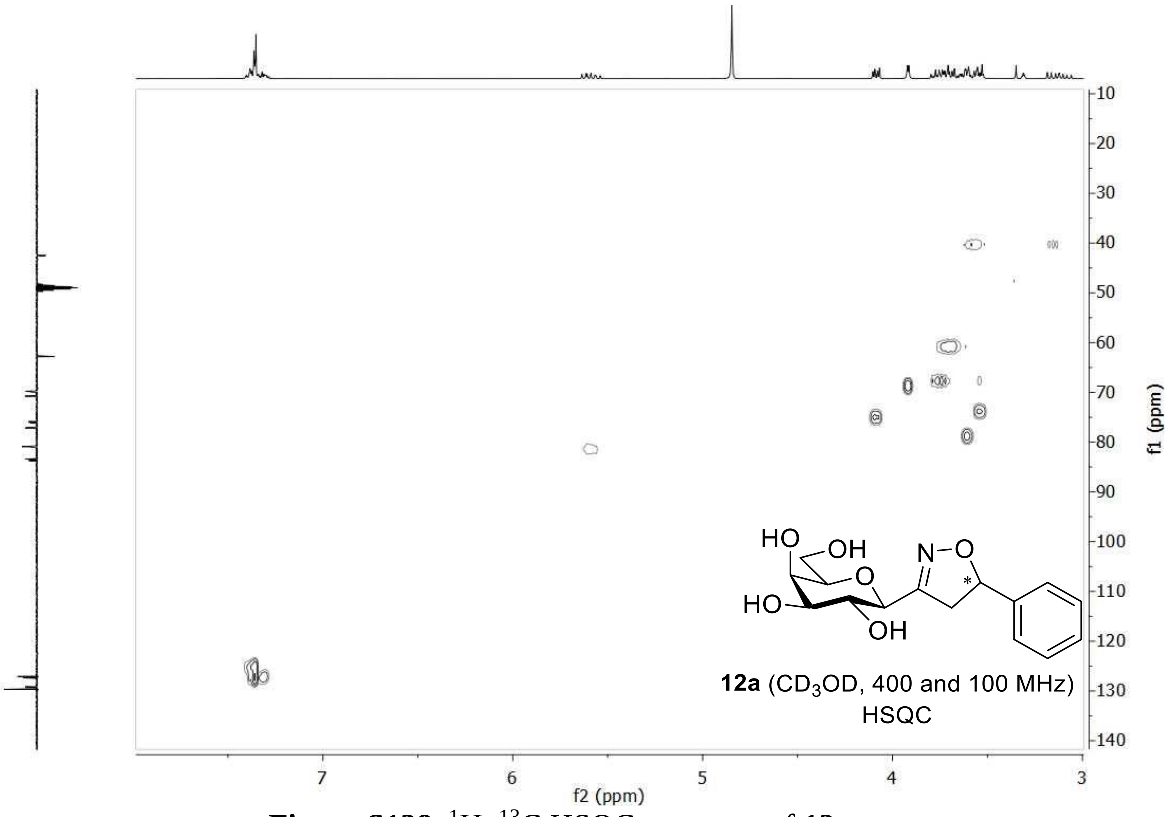

Figure S138. ^{1}H–^{13}C HSQC spectrum of **12a**

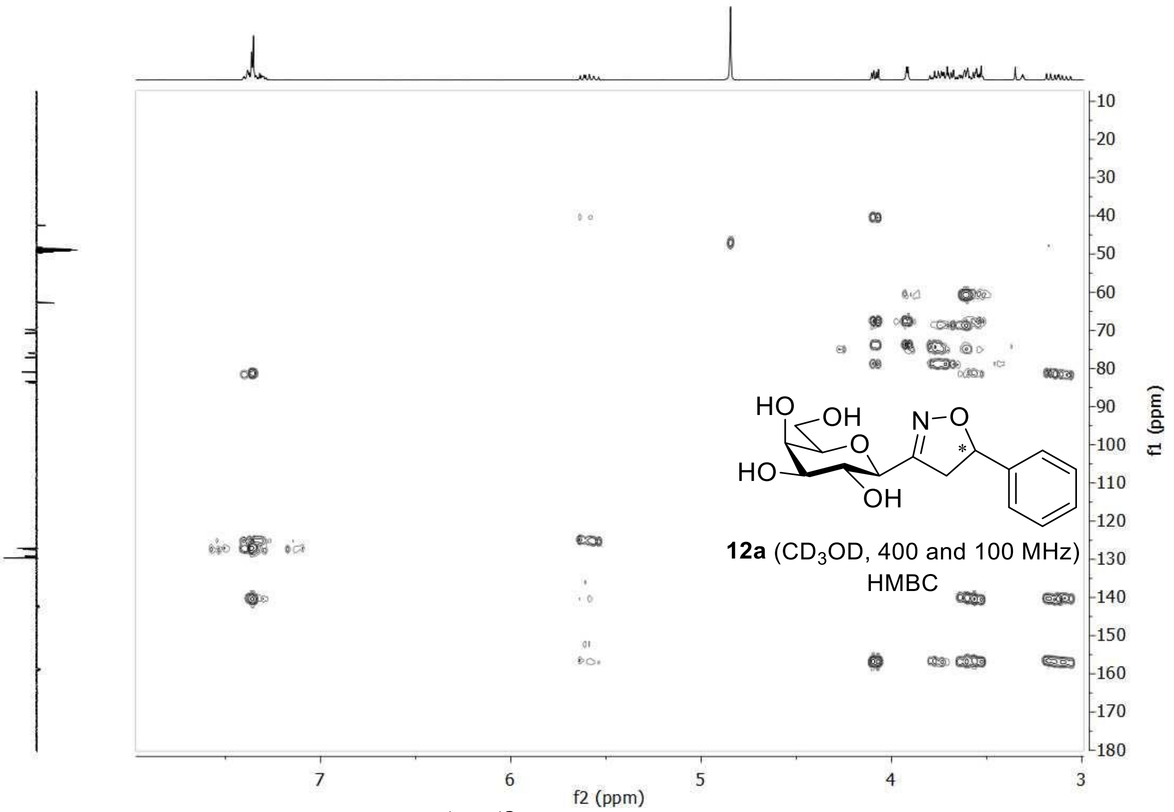

Figure S139. $^{1}H–^{13}C$ HMBC spectrum of **12a**

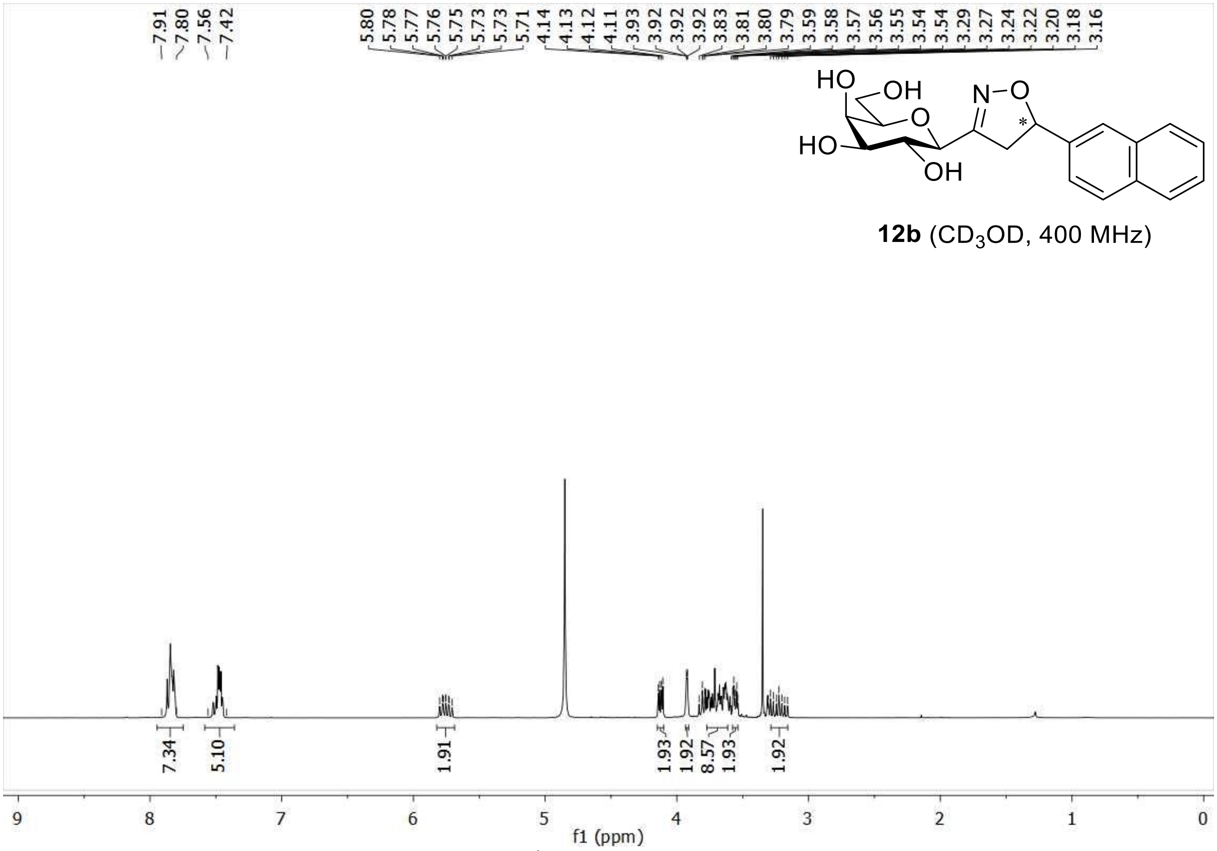

Figure S140. ^{1}H NMR spectrum of **12b**

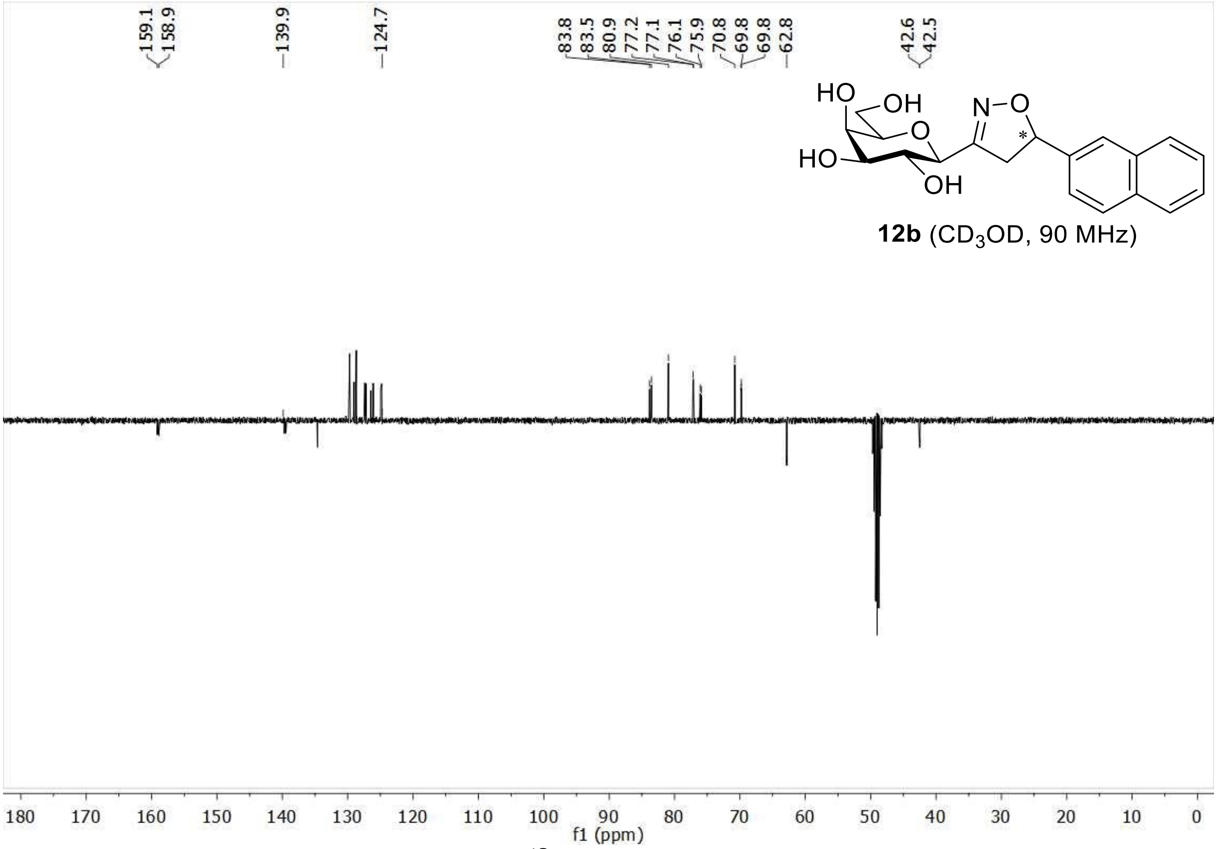

Figure S141. ^{13}C NMR spectrum of **12b**

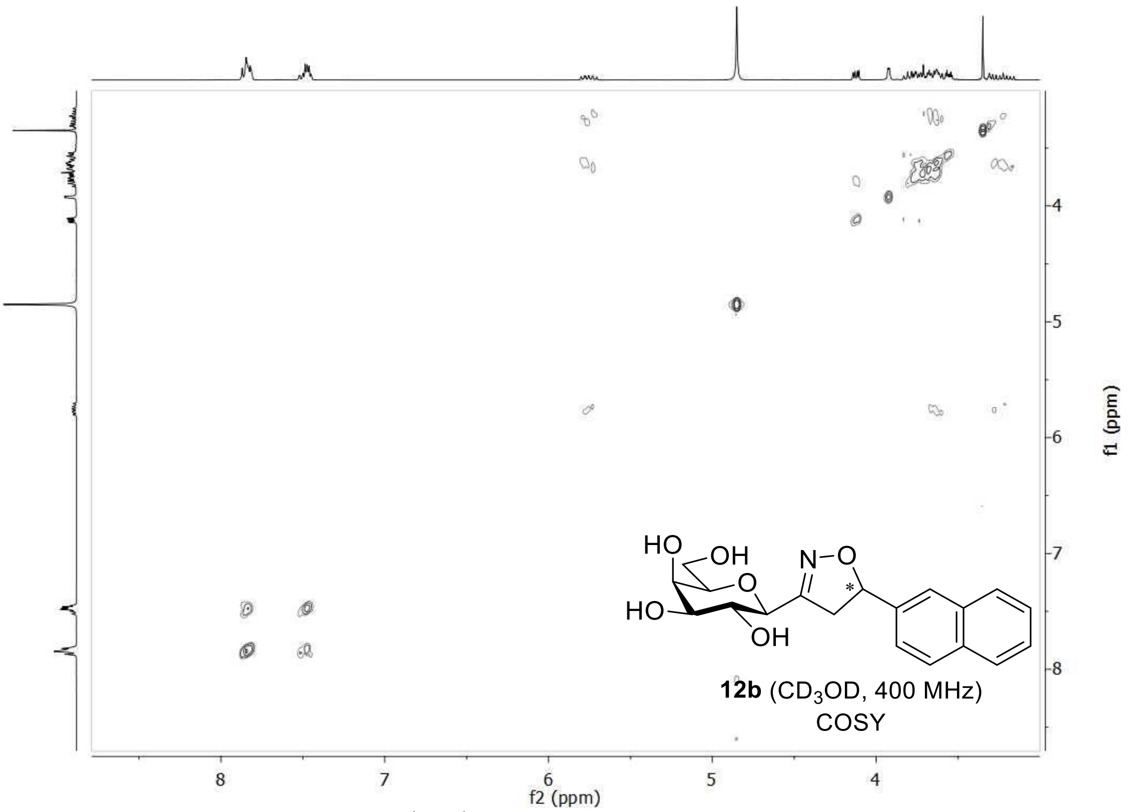

Figure S142. ^{1}H–^{1}H COSY spectrum of **12b**

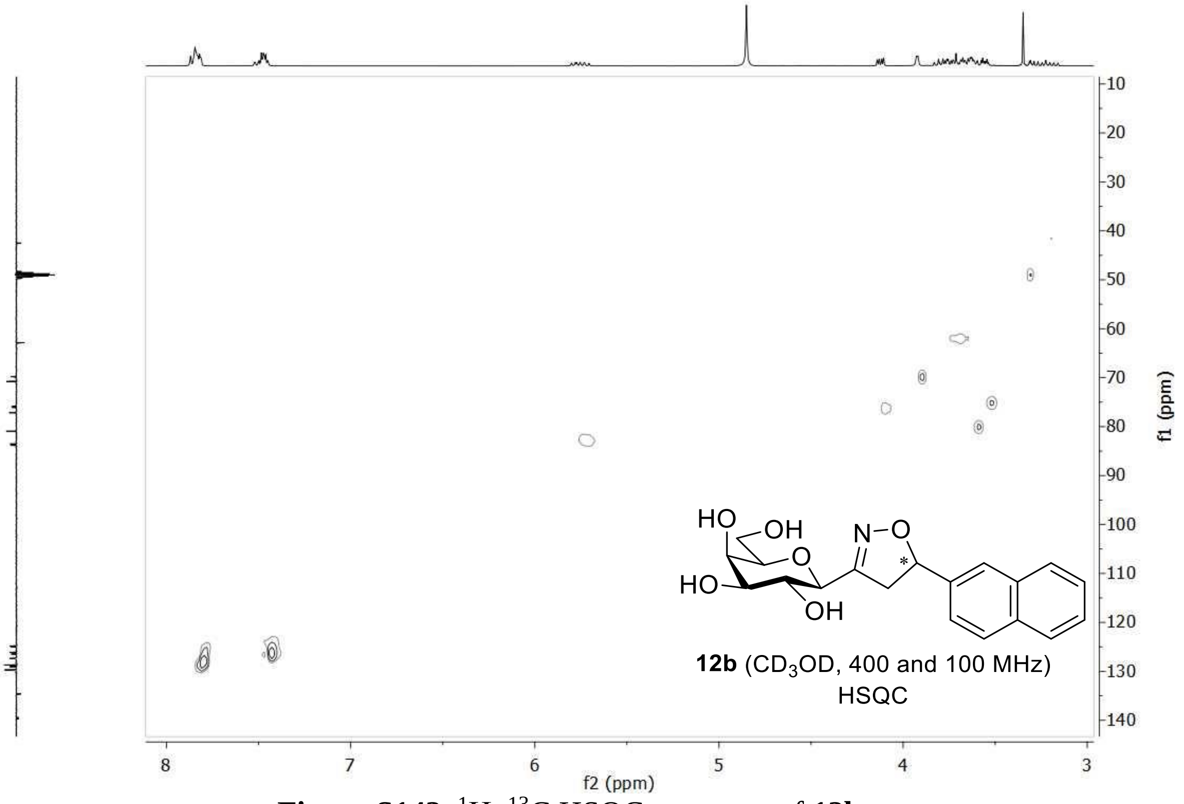

Figure S143. ^{1}H–^{13}C HSQC spectrum of **12b**

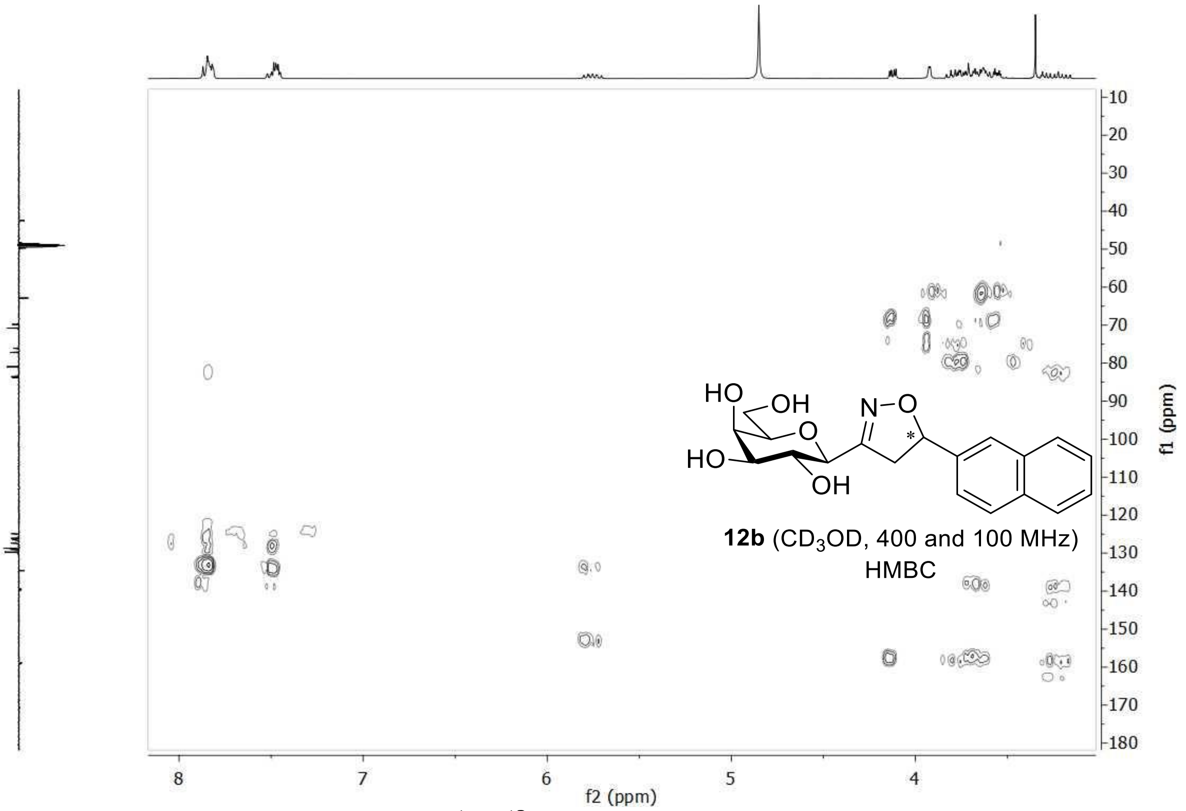

Figure S144. ^{1}H–^{13}C HMBC spectrum of **12b**

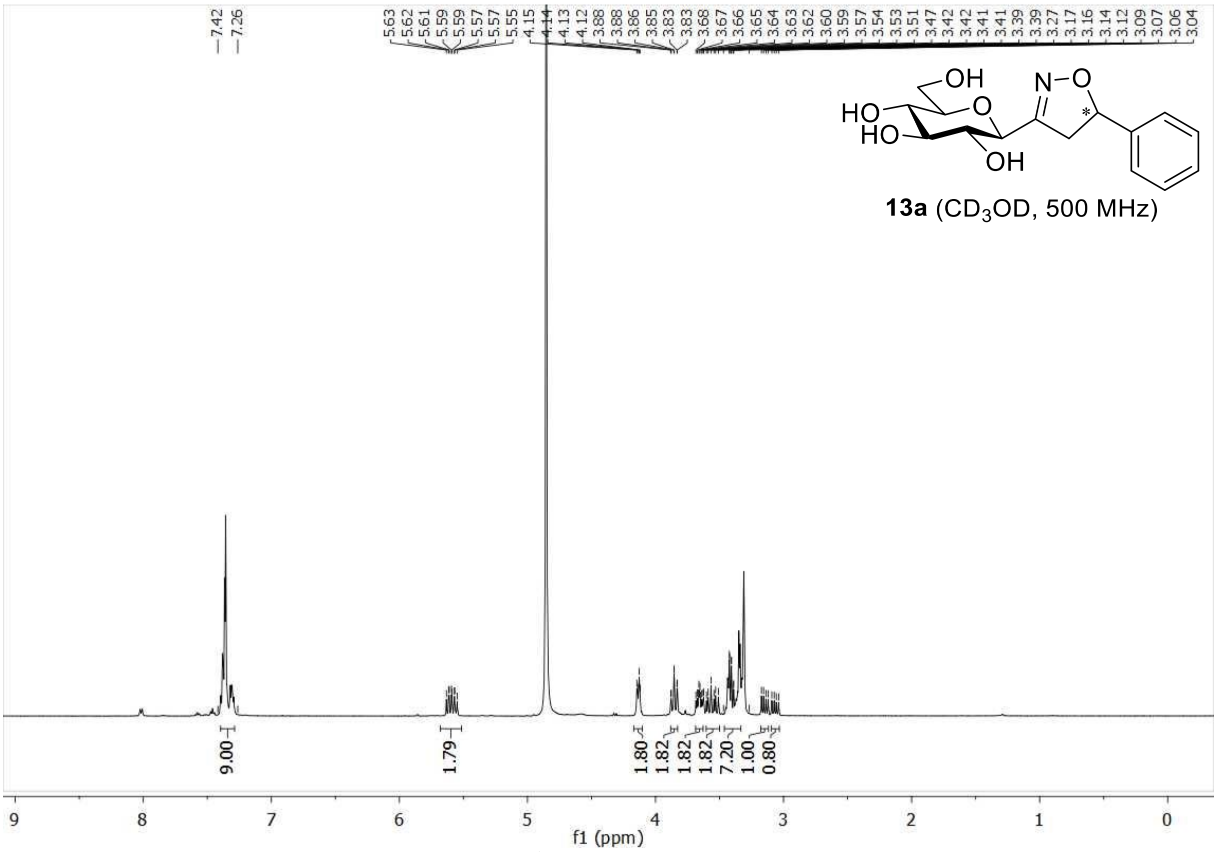

Figure S145. ^{1}H NMR spectrum of **13a**

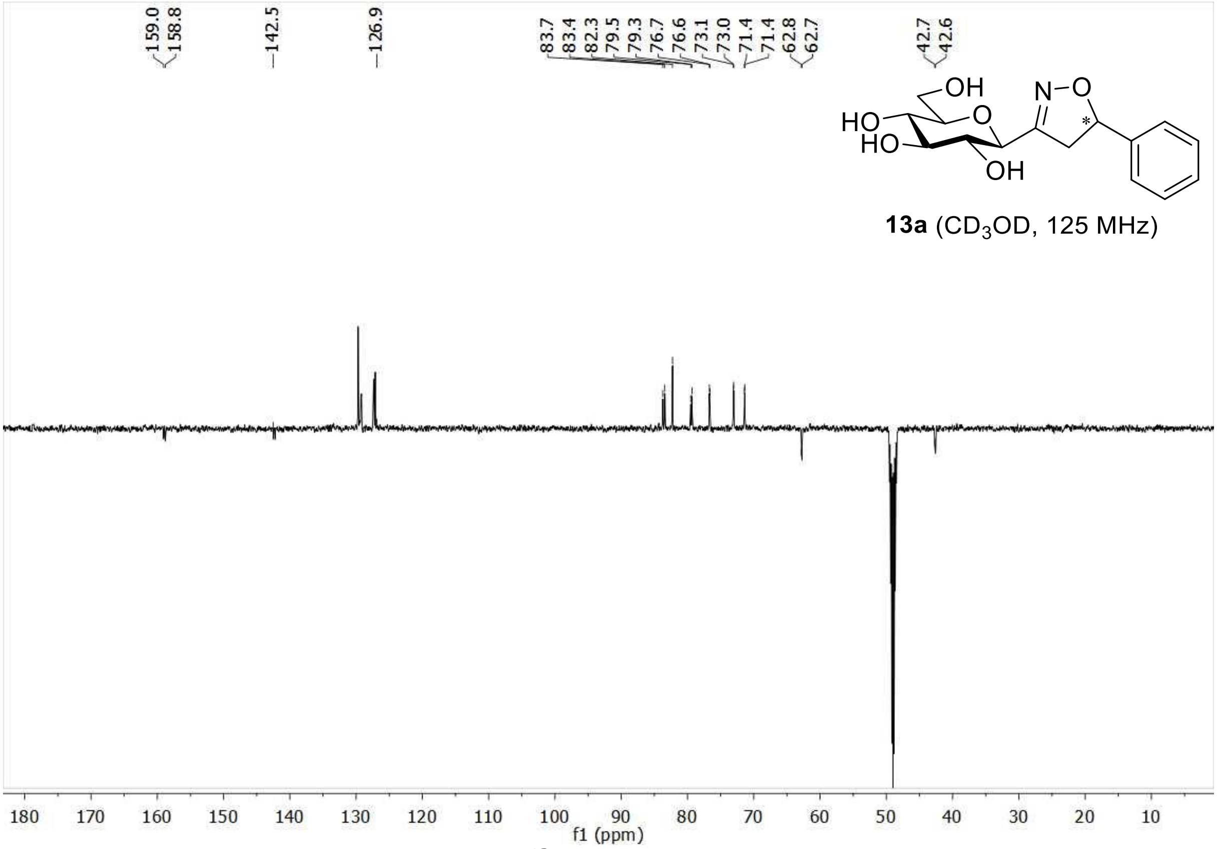

Figure S146. ^{13}C NMR spectrum of **13a**

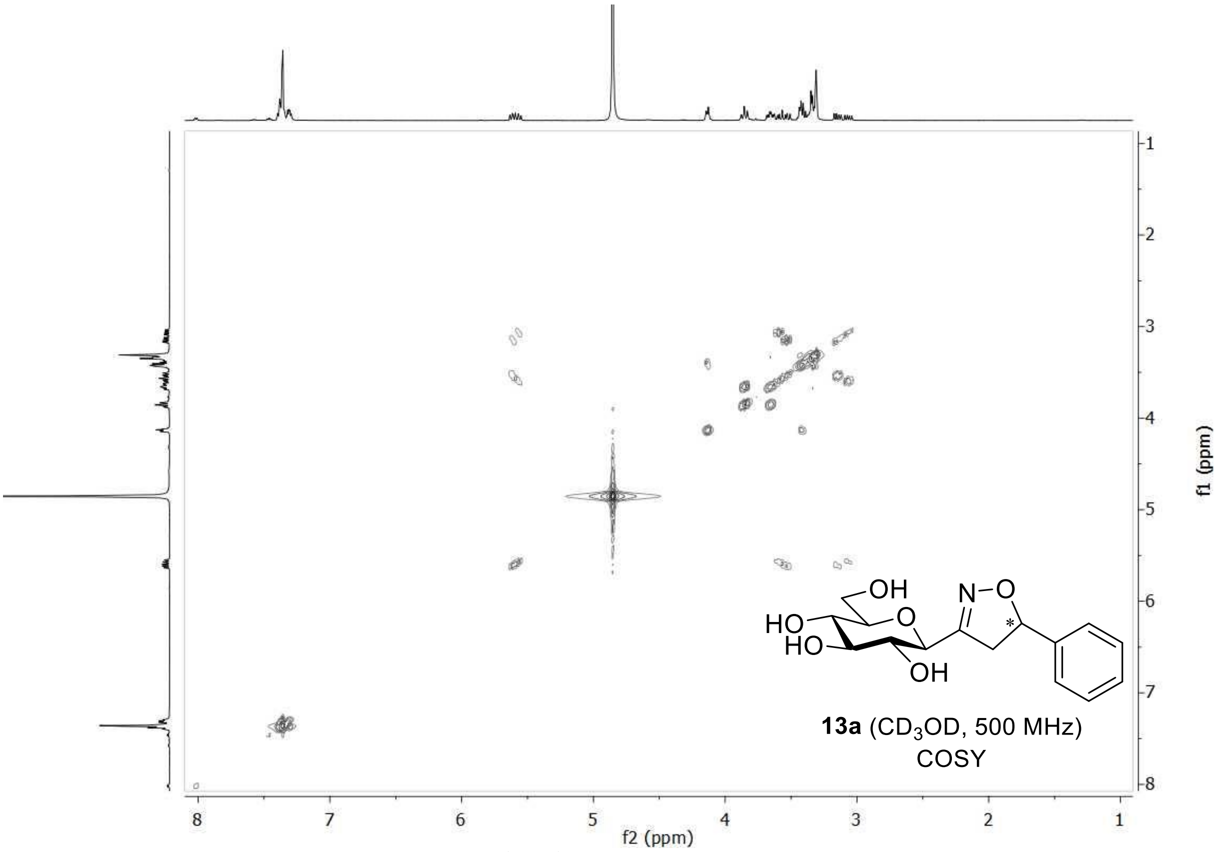

Figure S147. ^{1}H–^{1}H COSY spectrum of **13a**

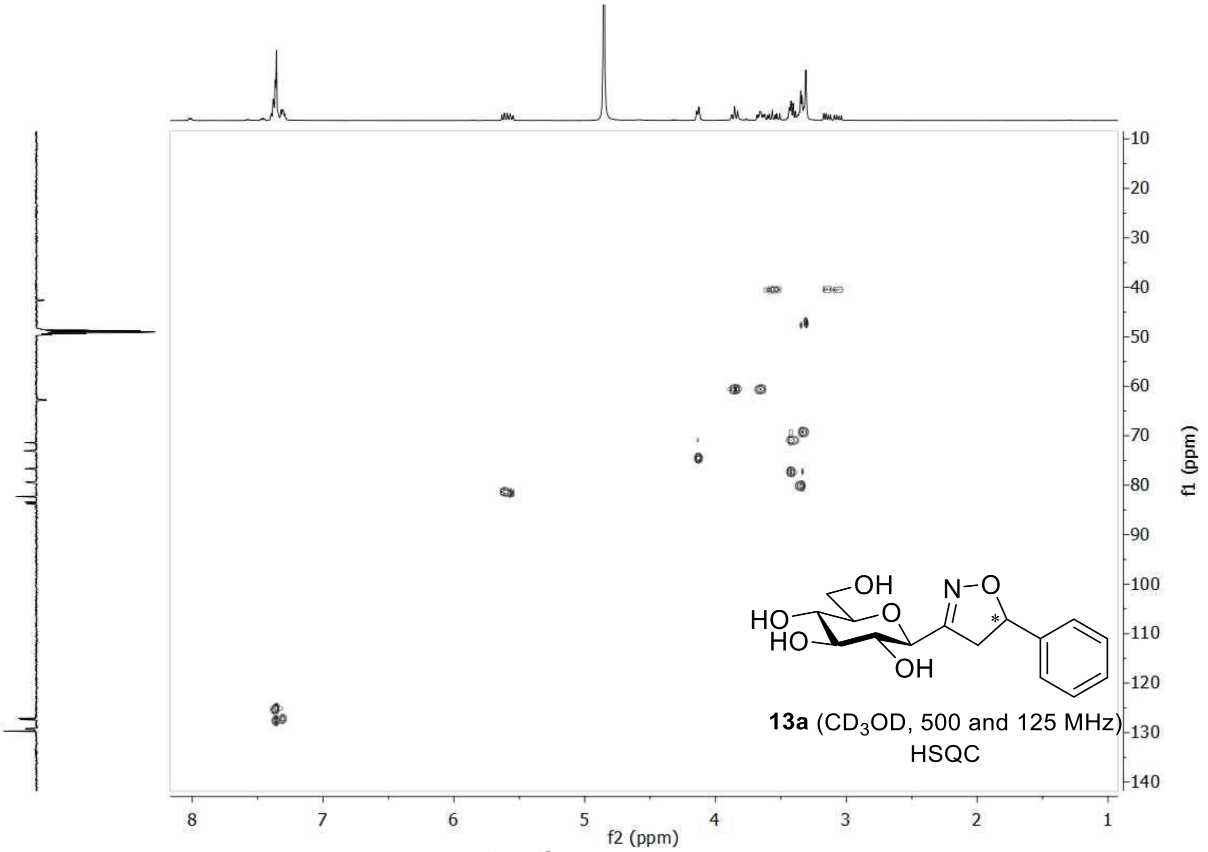

Figure S148. ^{1}H–^{13}C HSQC spectrum of **13a**

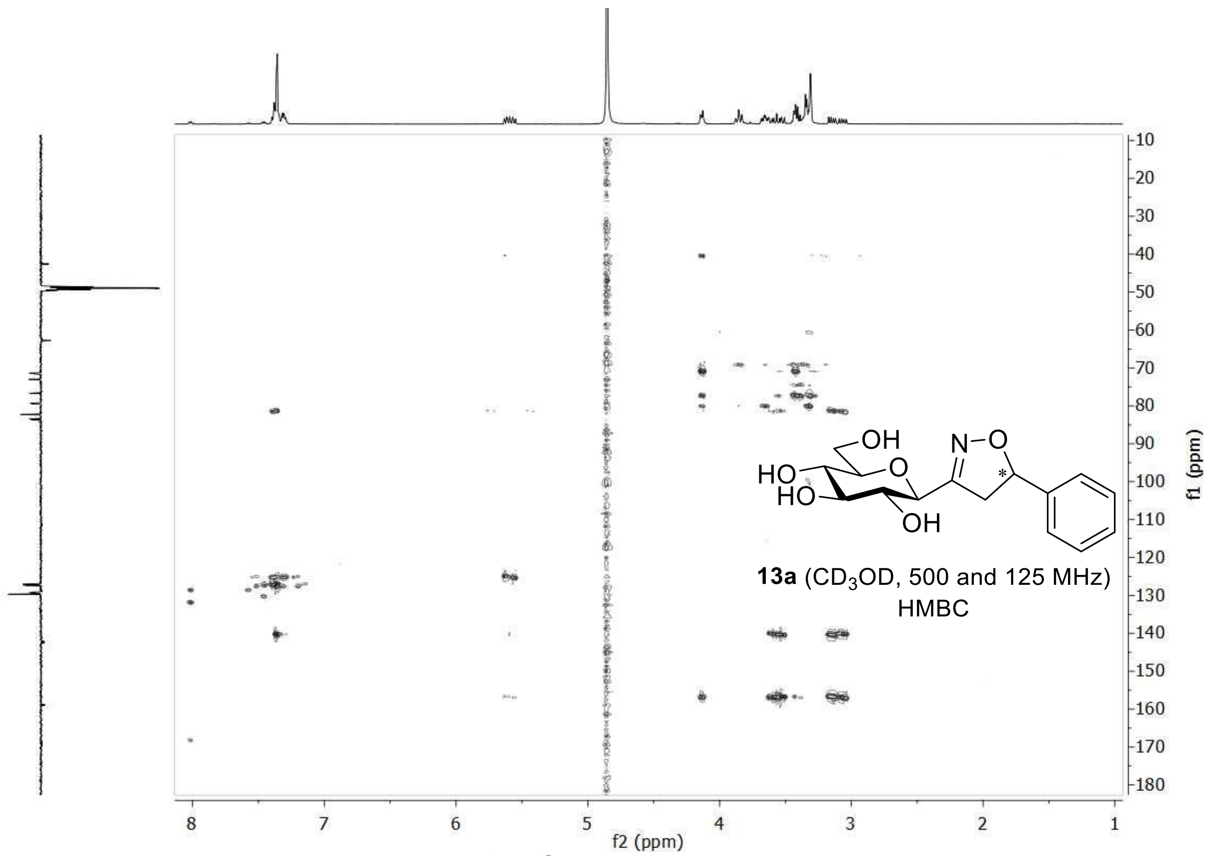

Figure S149. ^{1}H–^{13}C HMBC spectrum of **13a**

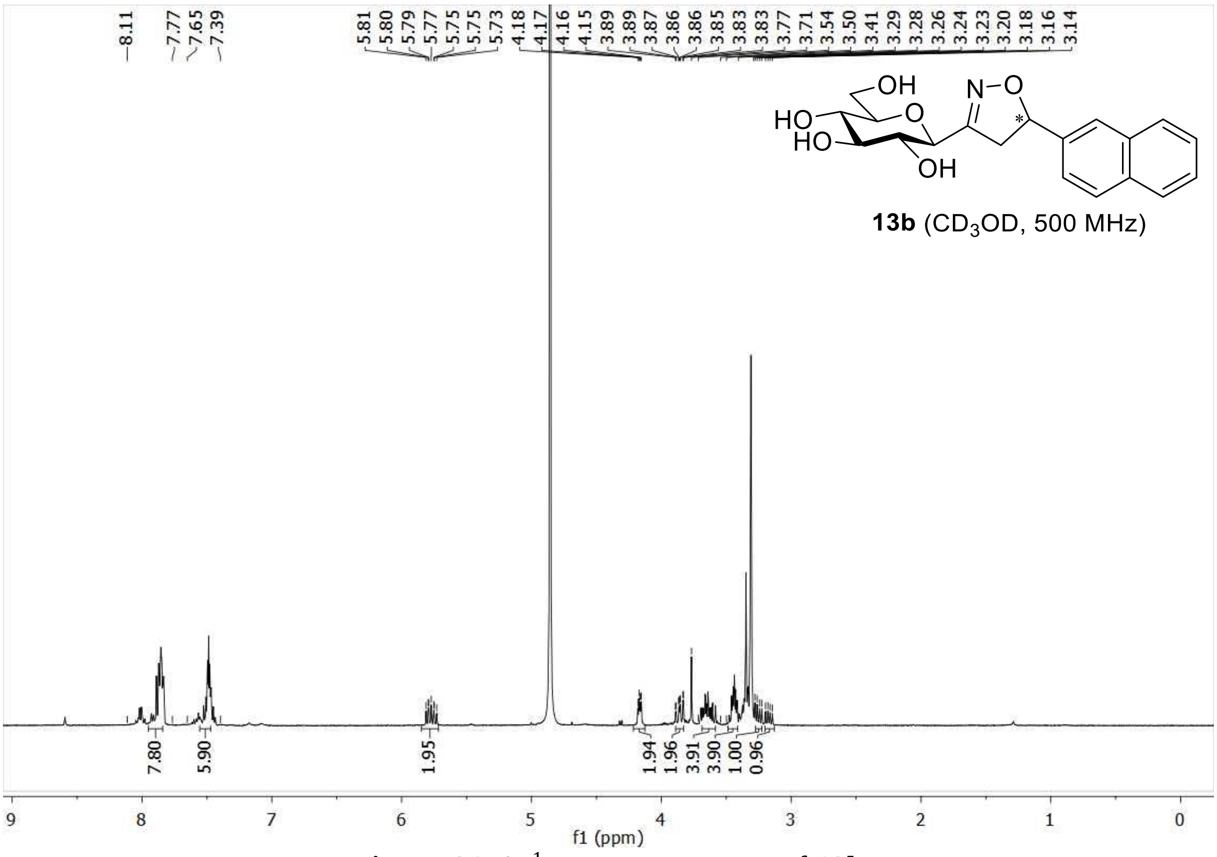

Figure S150. ^{1}H NMR spectrum of **13b**

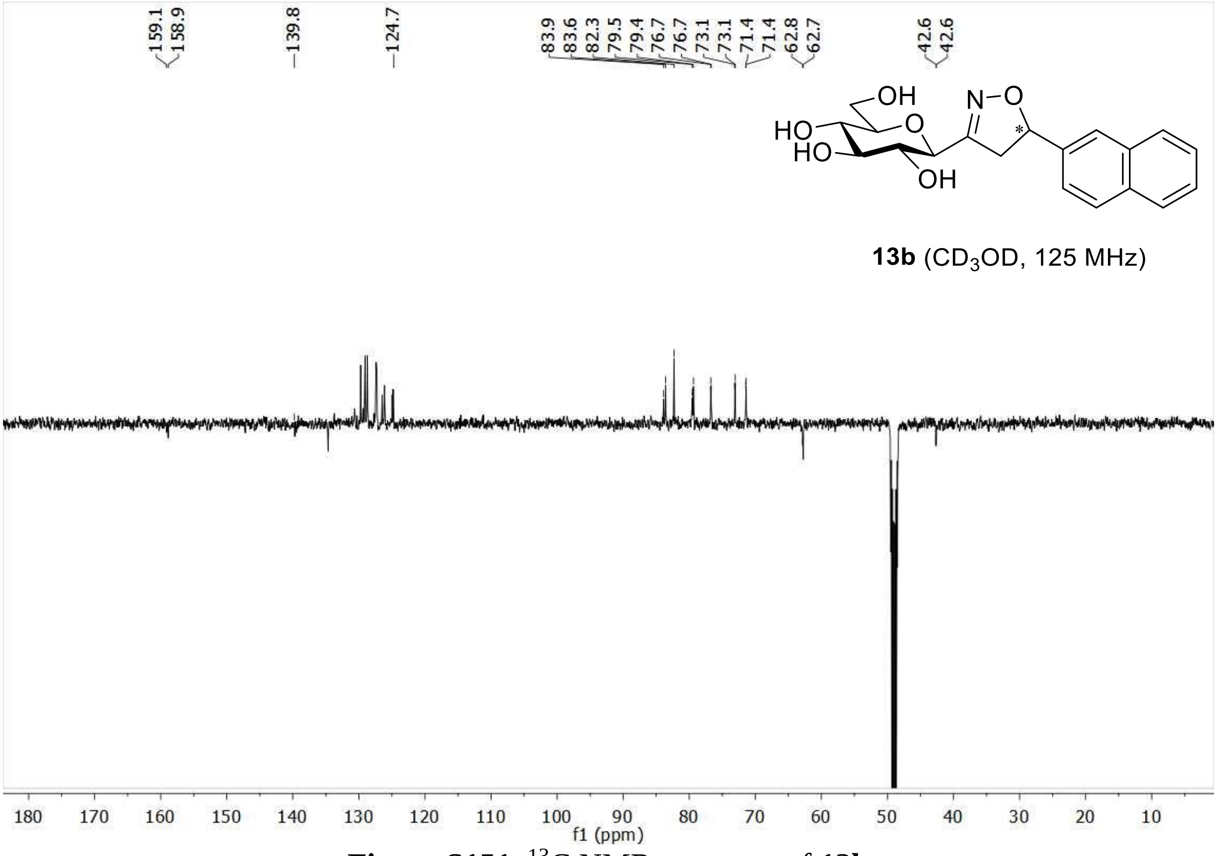

Figure S151. ^{13}C NMR spectrum of **13b**

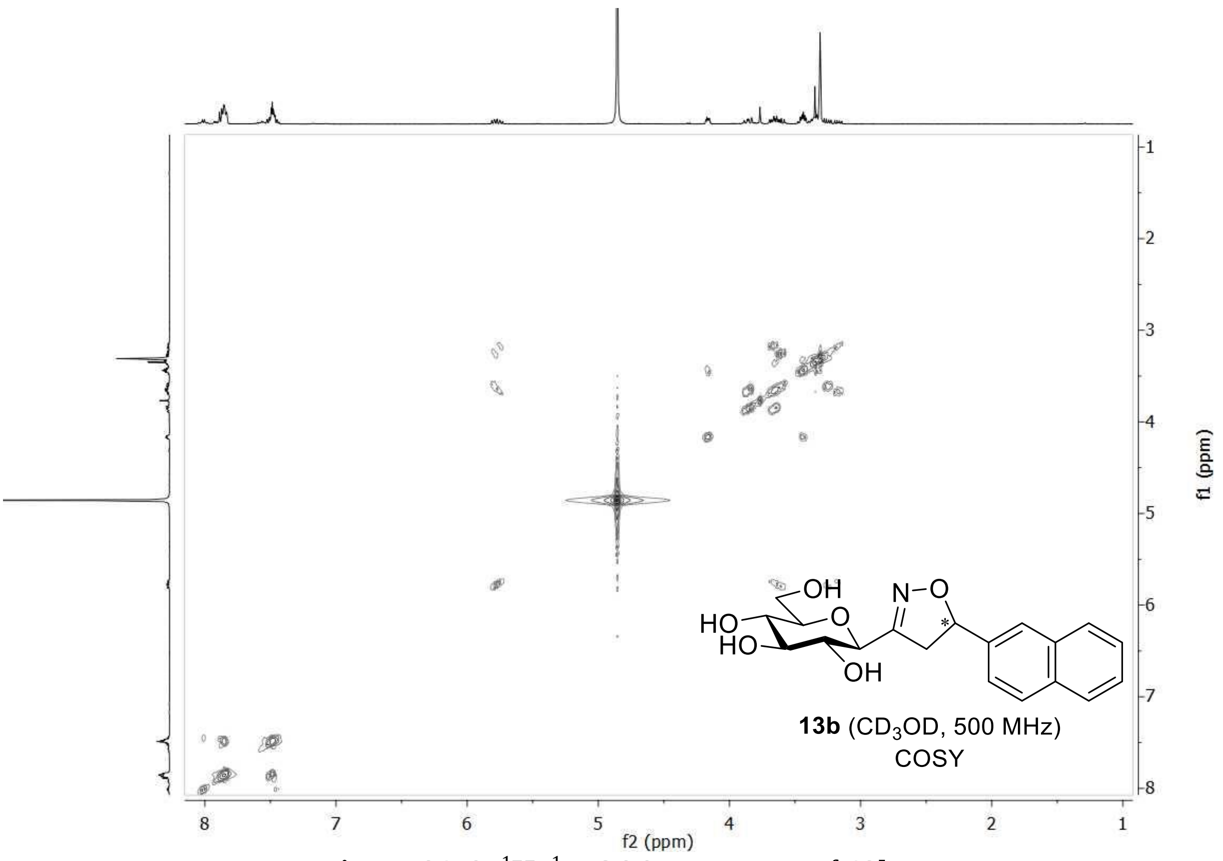

Figure S152. $^{1}H–^{1}H$ COSY spectrum of **13b**

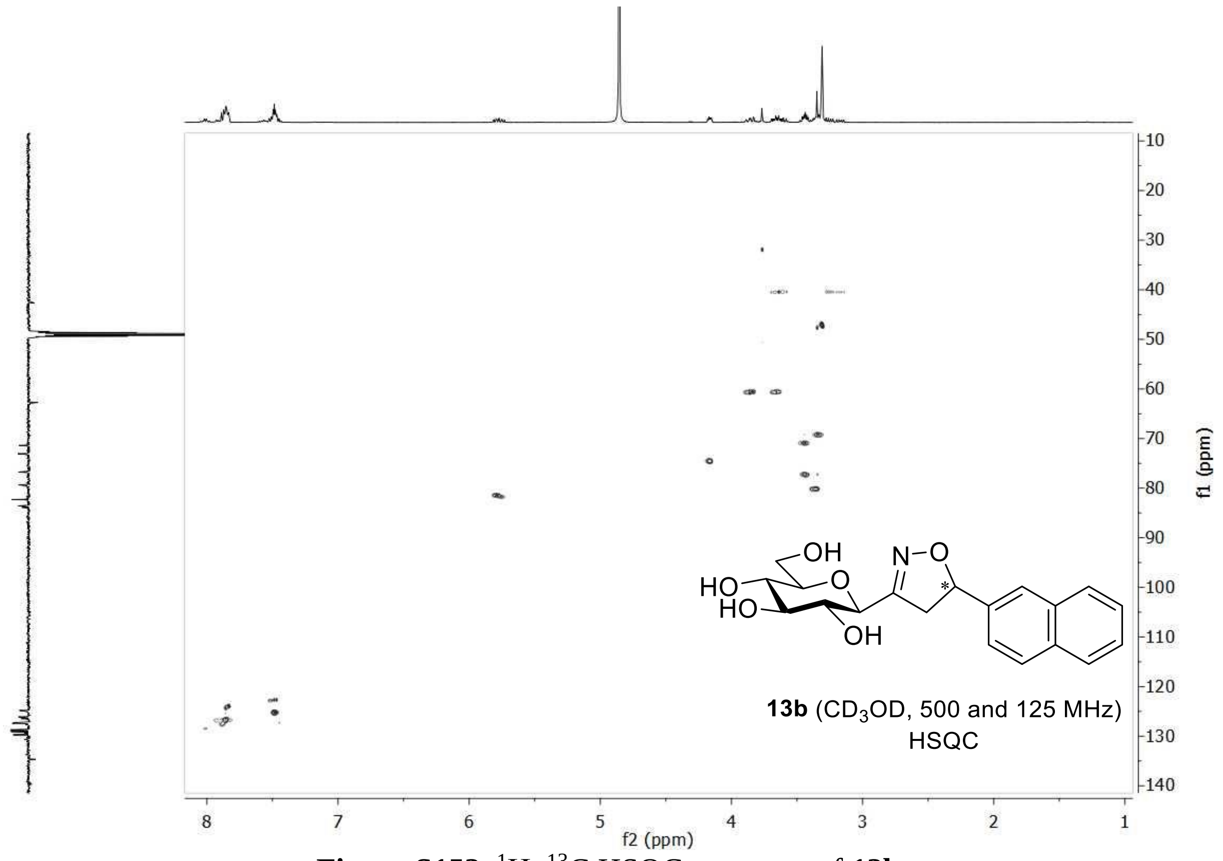

Figure S153. $^{1}H–^{13}C$ HSQC spectrum of **13b**

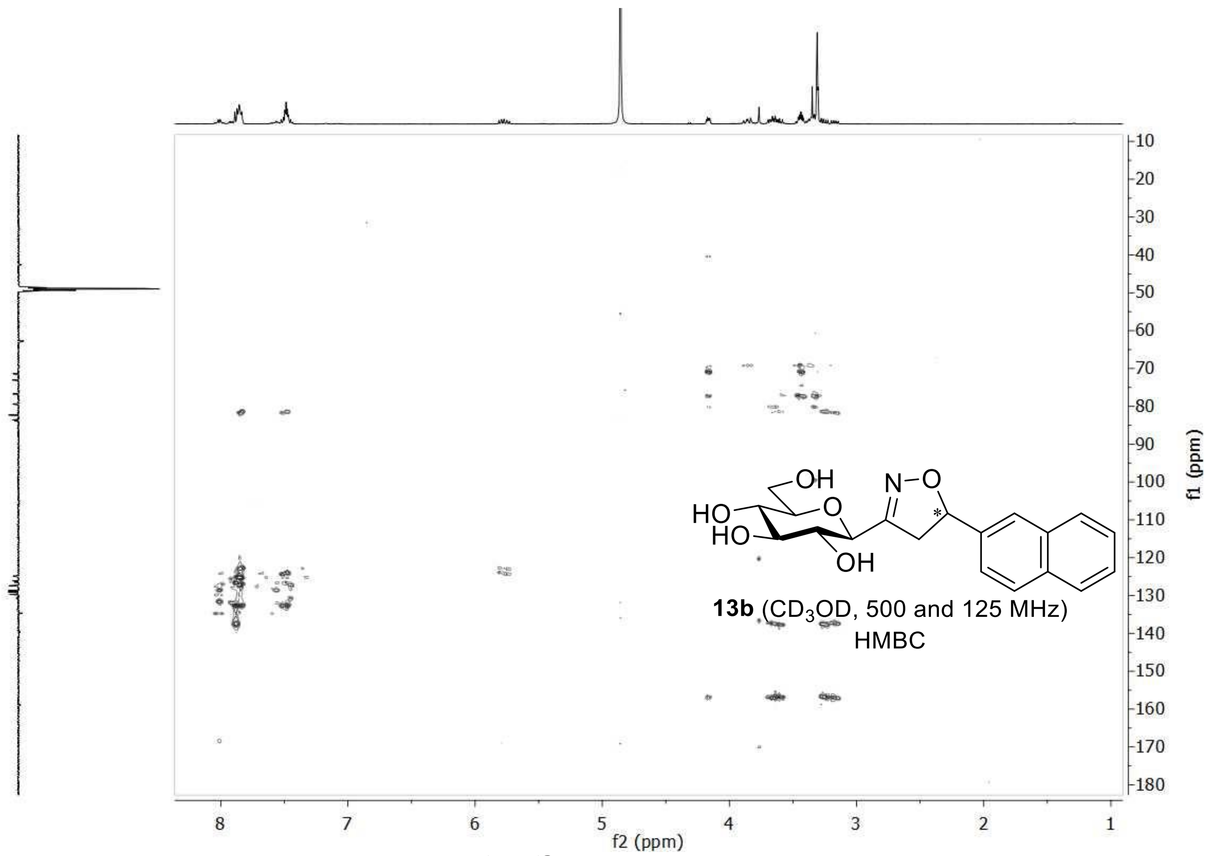

Figure S154. ^{1}H–^{13}C HMBC spectrum of **13b**

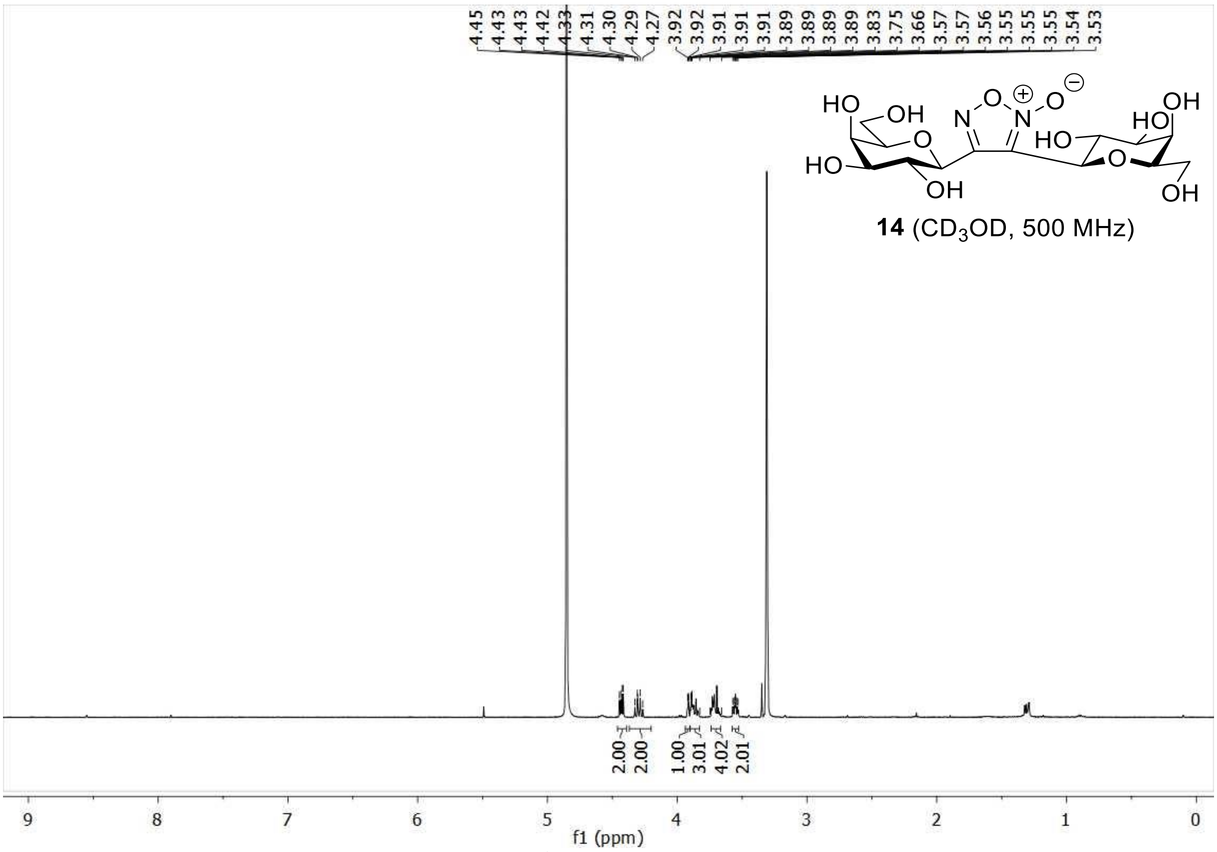

Figure S155. ^{1}H NMR spectrum of **14**

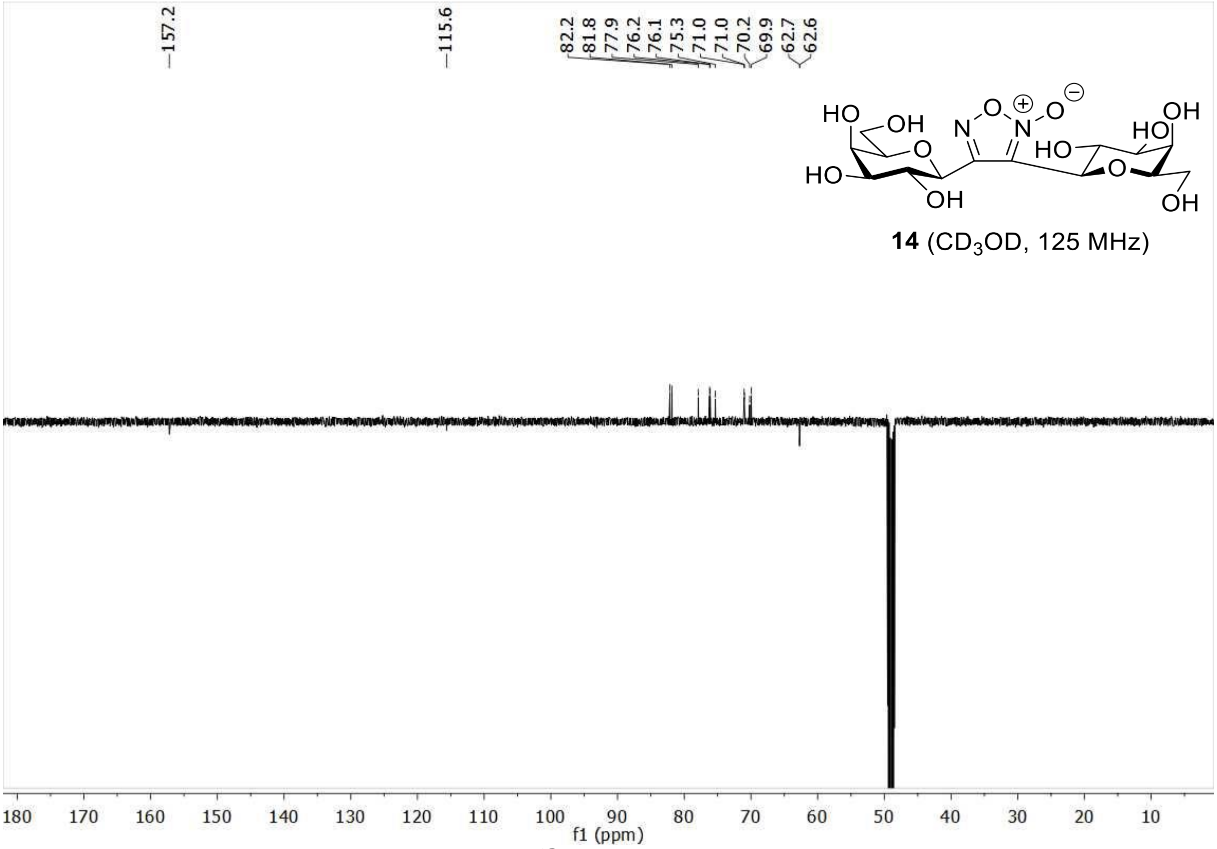

Figure S156. ^{13}C NMR spectrum of **14**

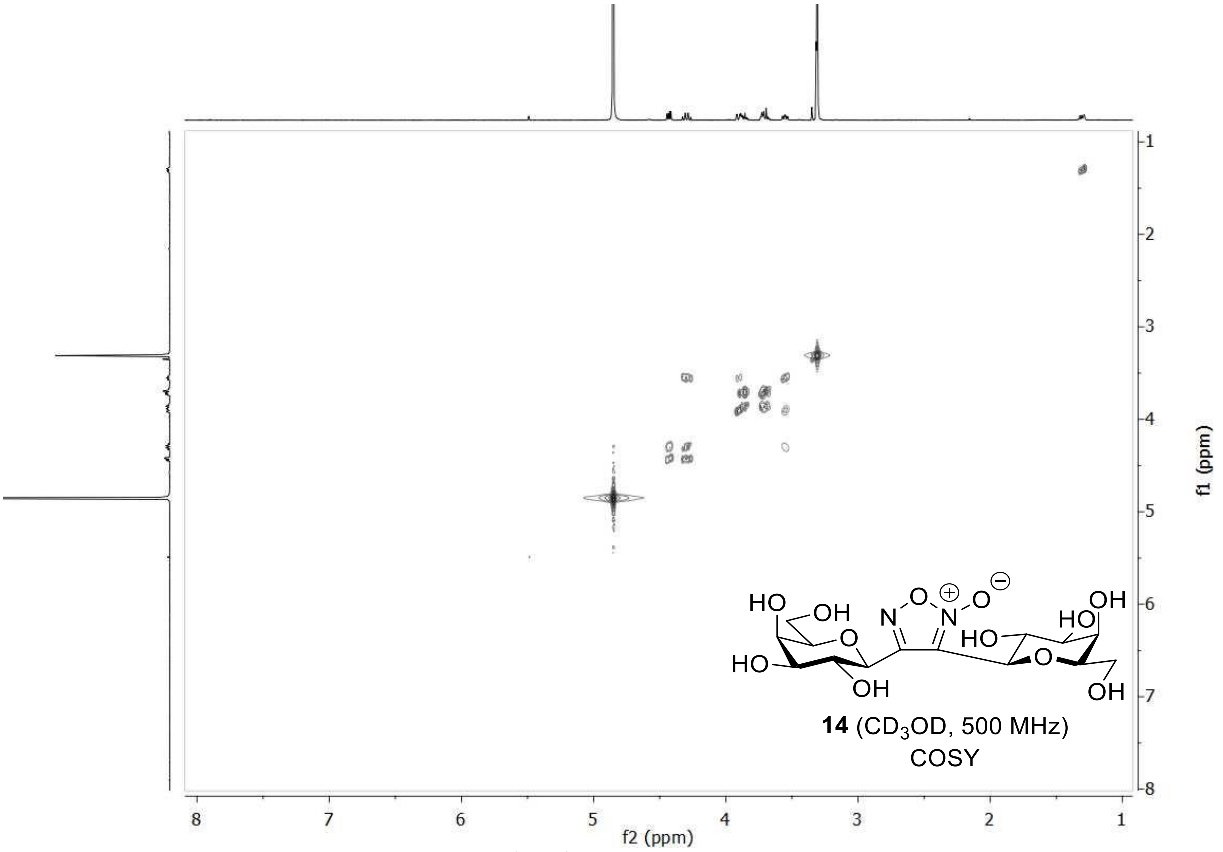

Figure S157. ^{1}H–^{1}H COSY spectrum of **14**

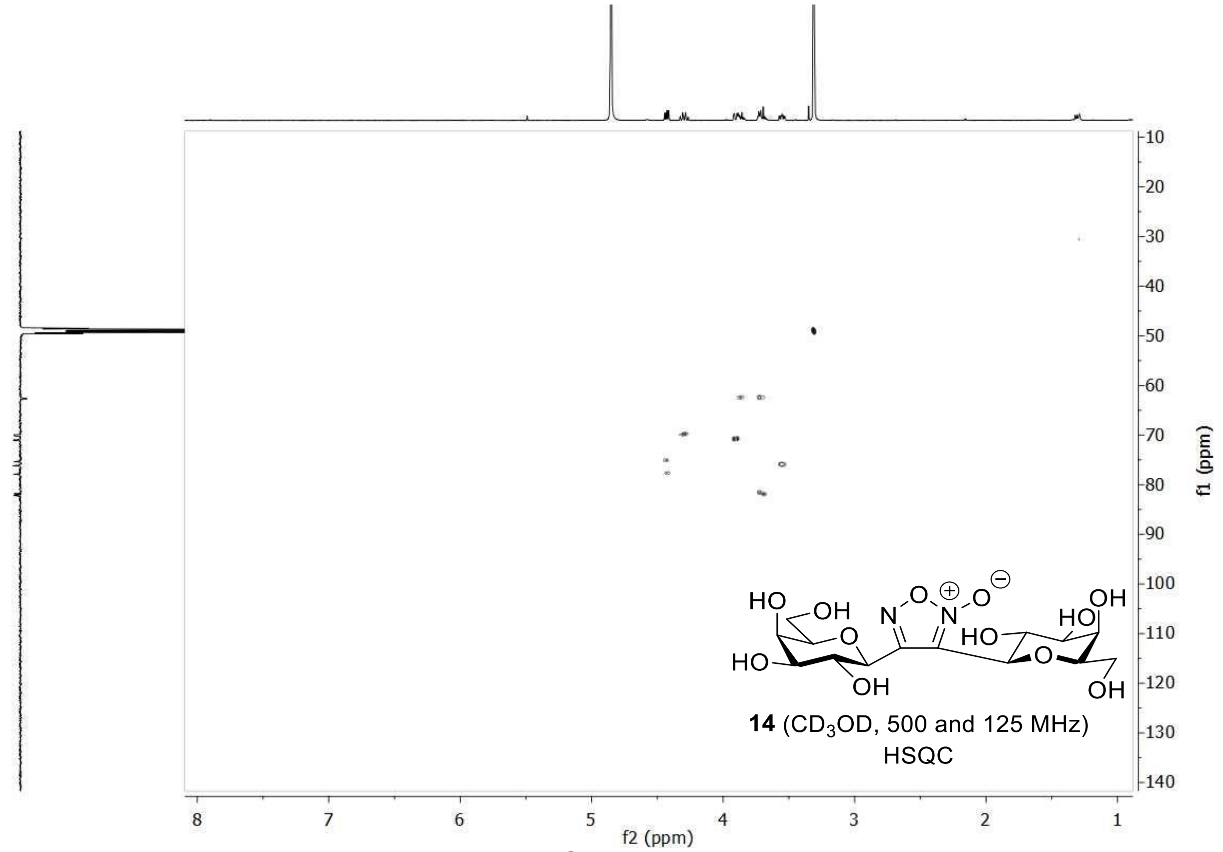

Figure S158. ^{1}H–^{13}C HSQC spectrum of **14**

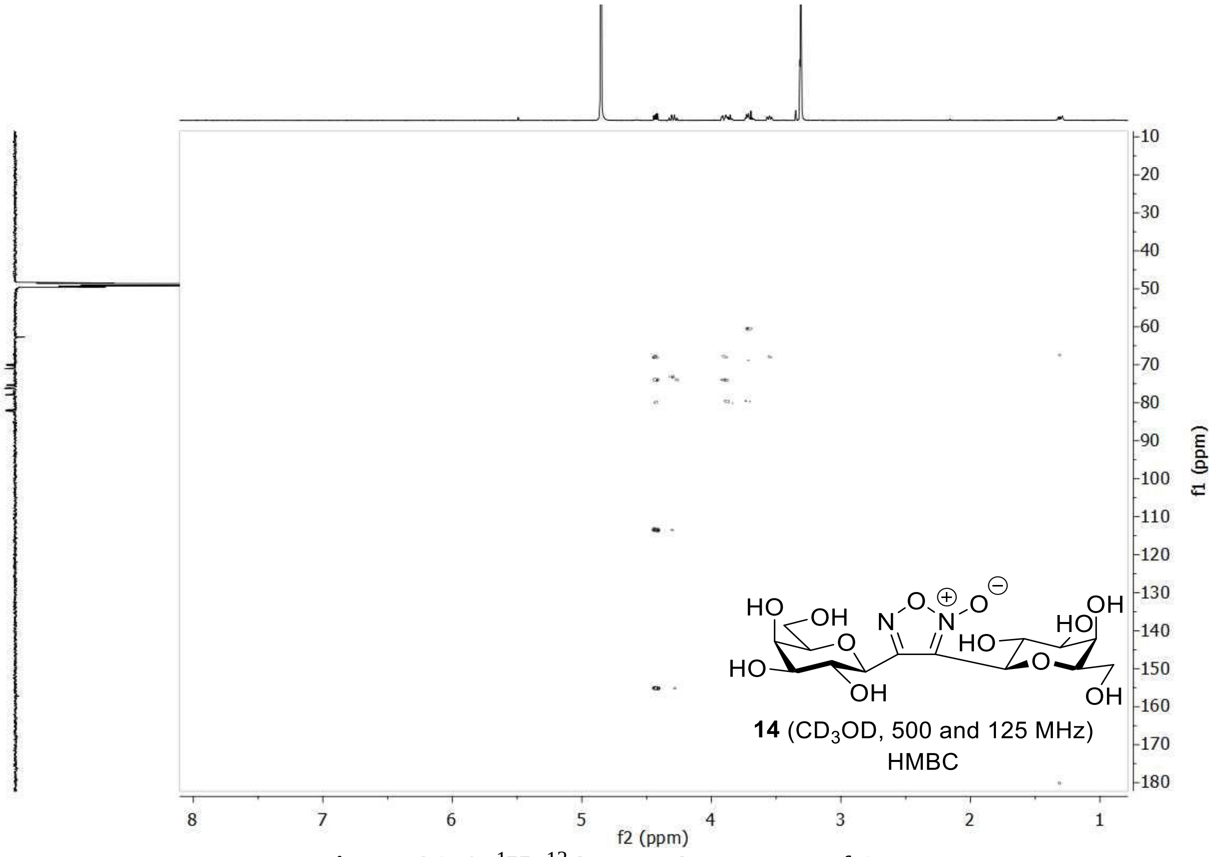

Figure S159. ^{1}H–^{13}C HMBC spectrum of **14**

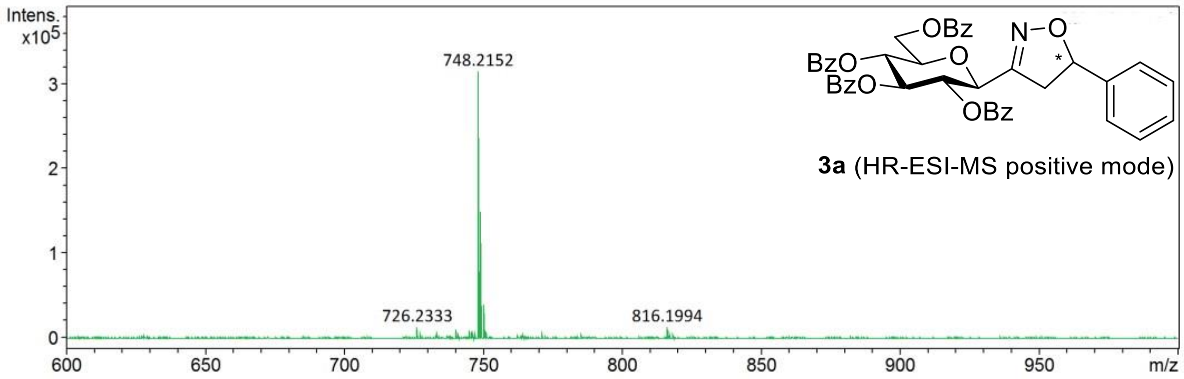

Figure S160. HR-ESI-MS spectrum of **3a**

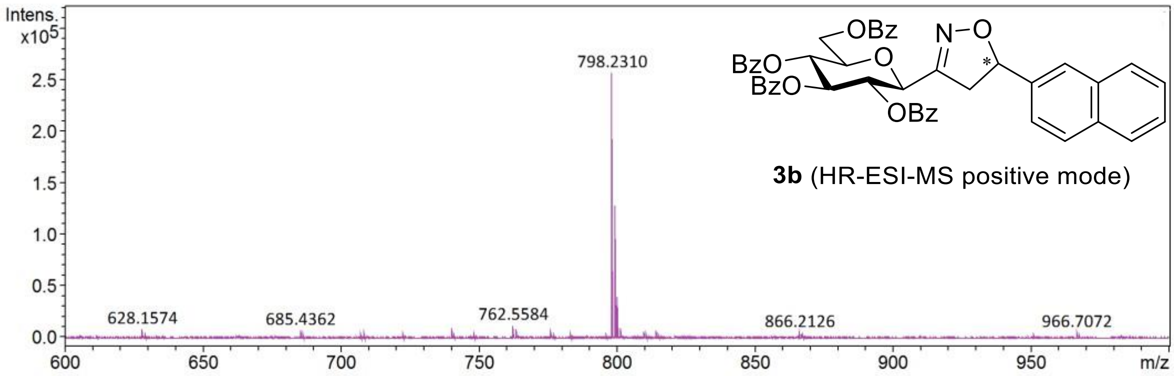

Figure S161. HR-ESI-MS spectrum of **3b**

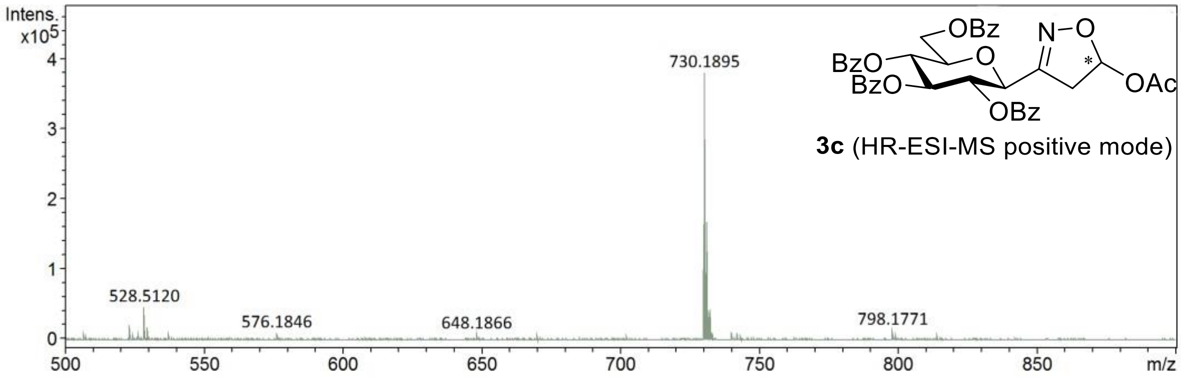

Figure S162. HR-ESI-MS spectrum of **3c**

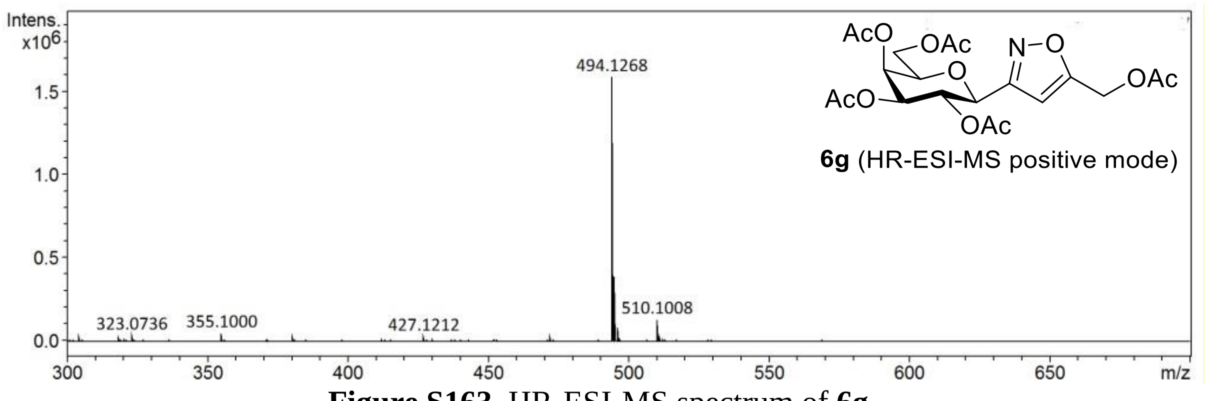

Figure S163. HR-ESI-MS spectrum of **6g**

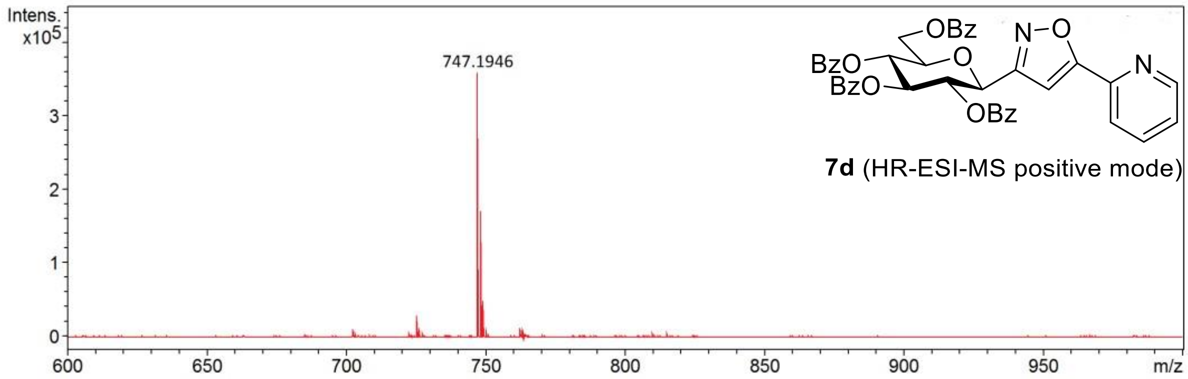

Figure S164. HR-ESI-MS spectrum of **7d**

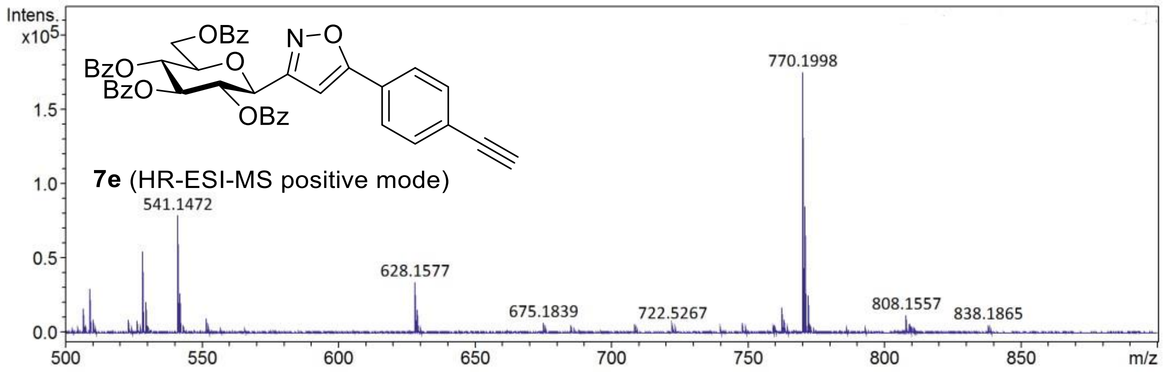

Figure S165. HR-ESI-MS spectrum of **7e**

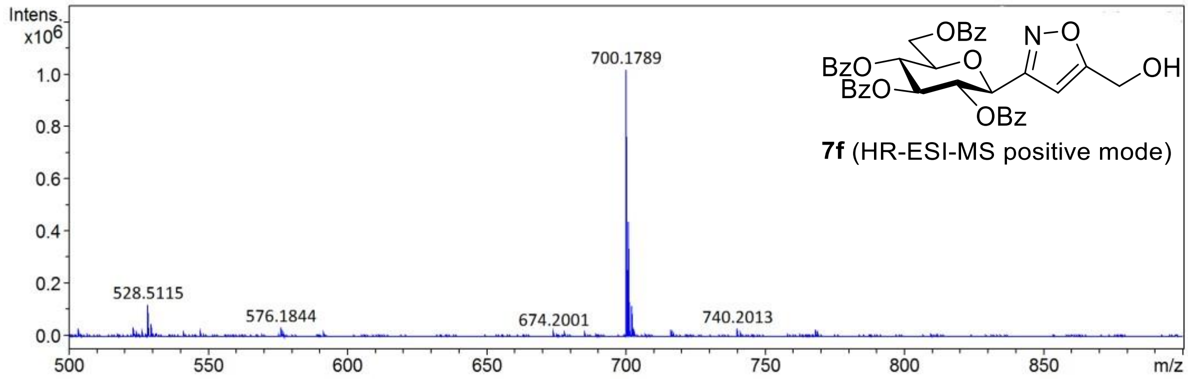

Figure S166. HR-ESI-MS spectrum of **7f**

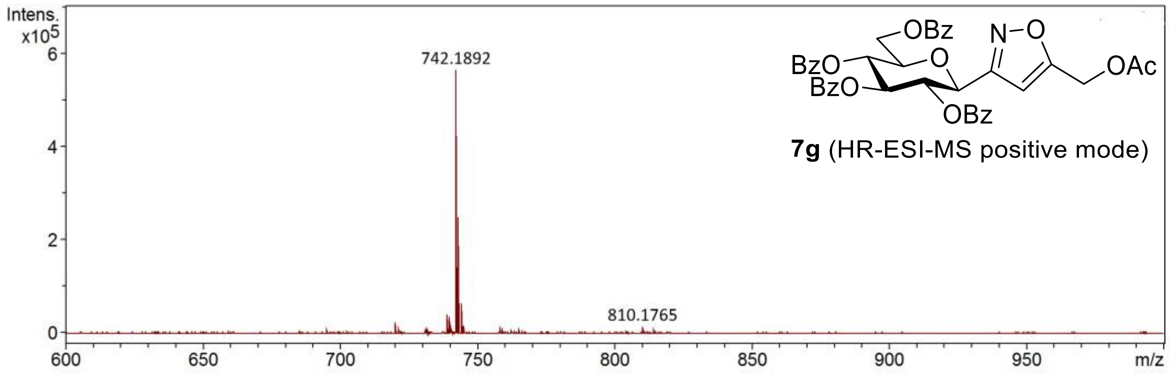

Figure S167. HR-ESI-MS spectrum of **7g**

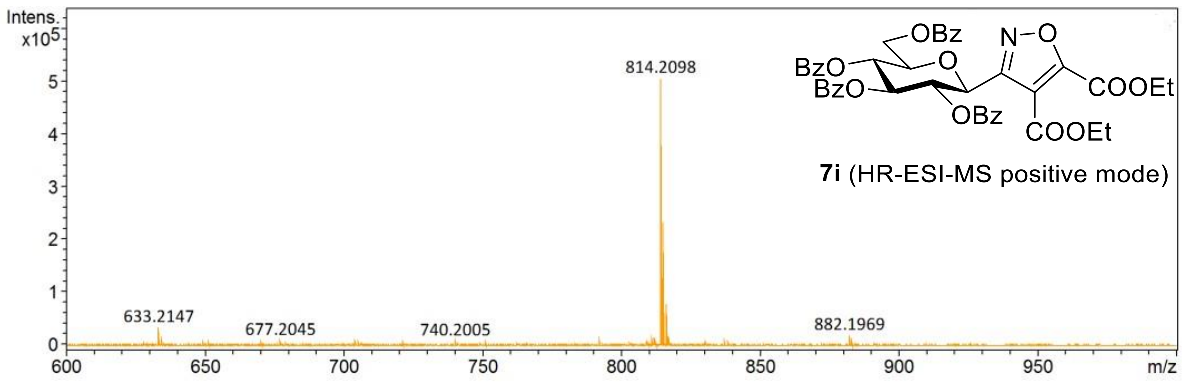

Figure S168. HR-ESI-MS spectrum of **7i**

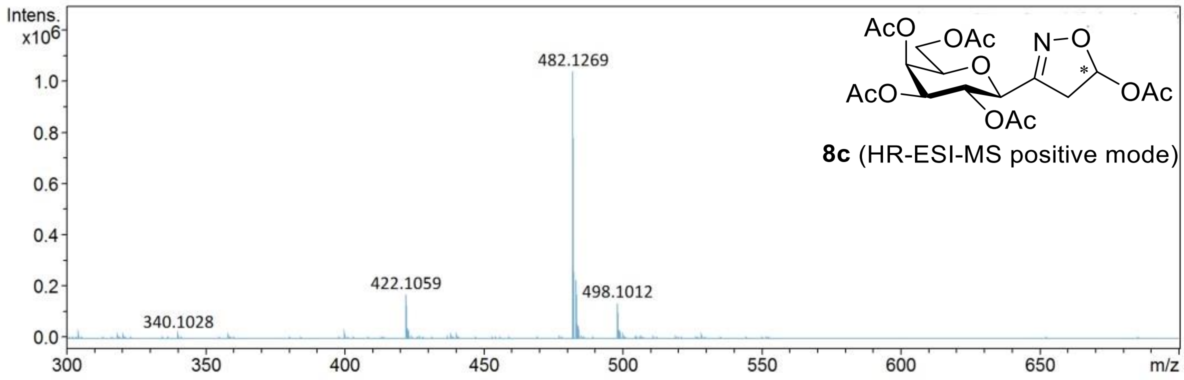

Figure S169. HR-ESI-MS spectrum of **8c**

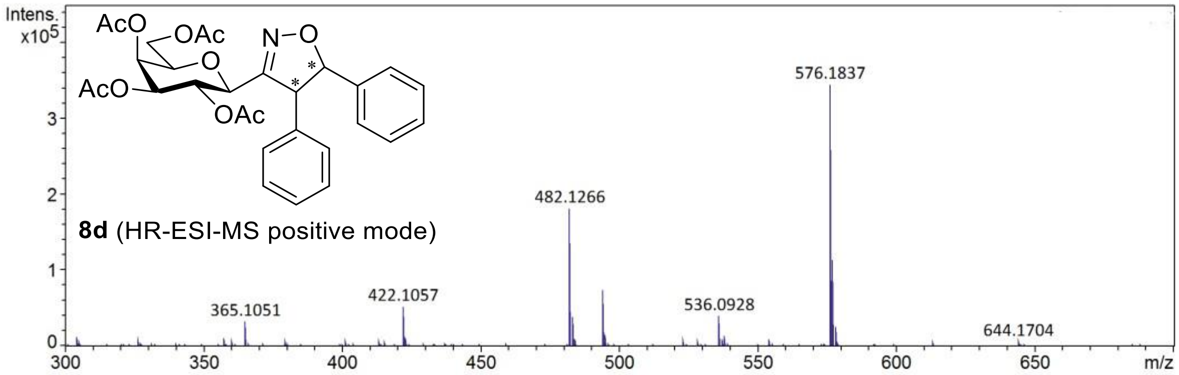

Figure S170. HR-ESI-MS spectrum of **8d**

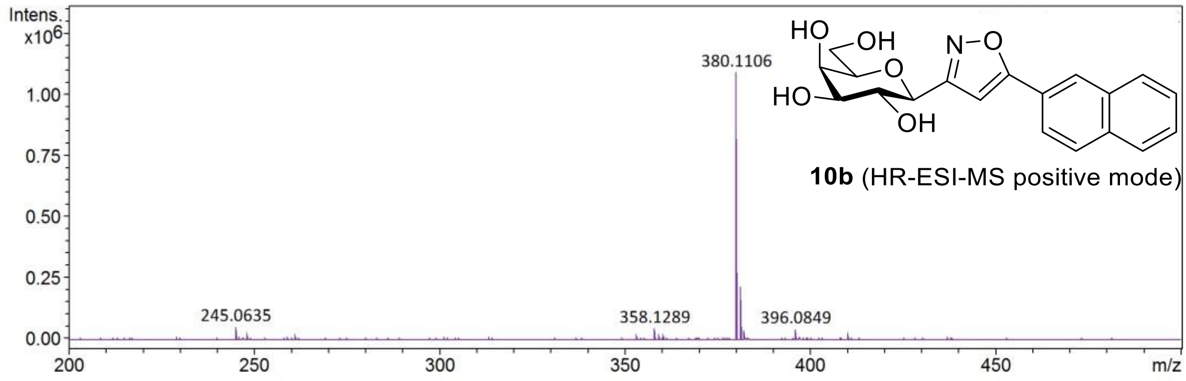

Figure S171. HR-ESI-MS spectrum of **10b**

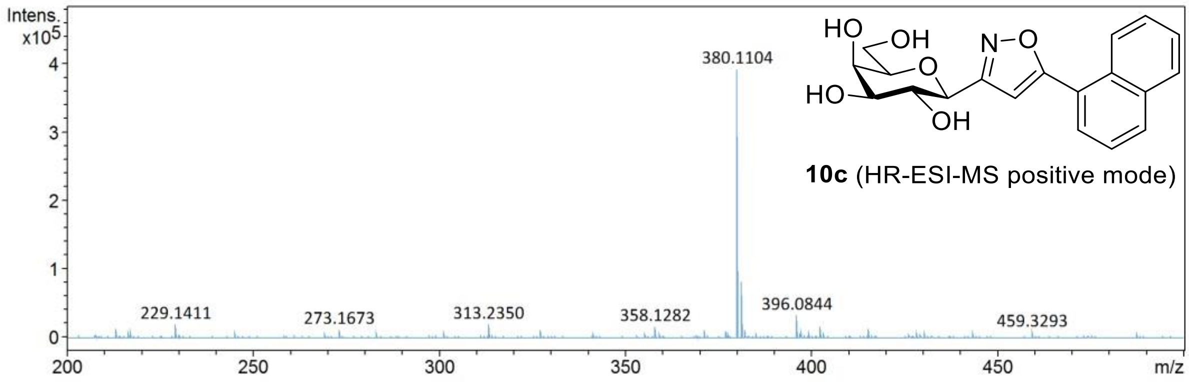

Figure S172. HR-ESI-MS spectrum of **10c**

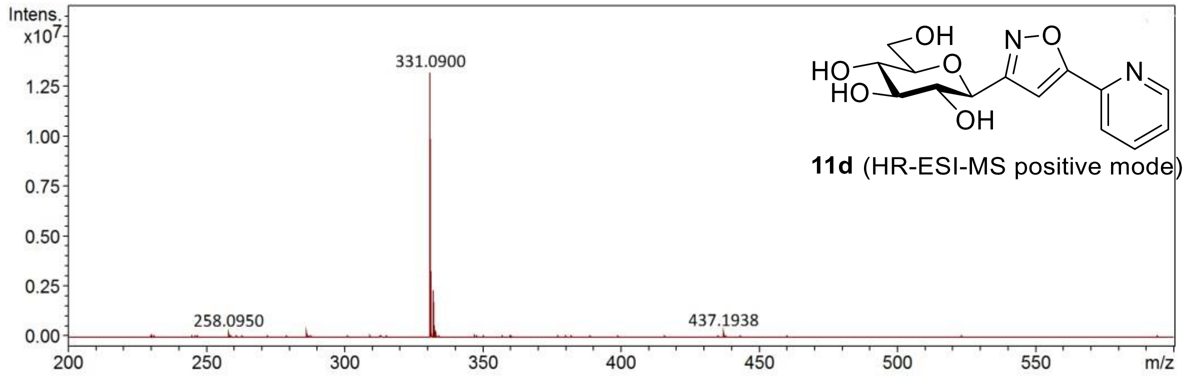

Figure S173. HR-ESI-MS spectrum of **11d**

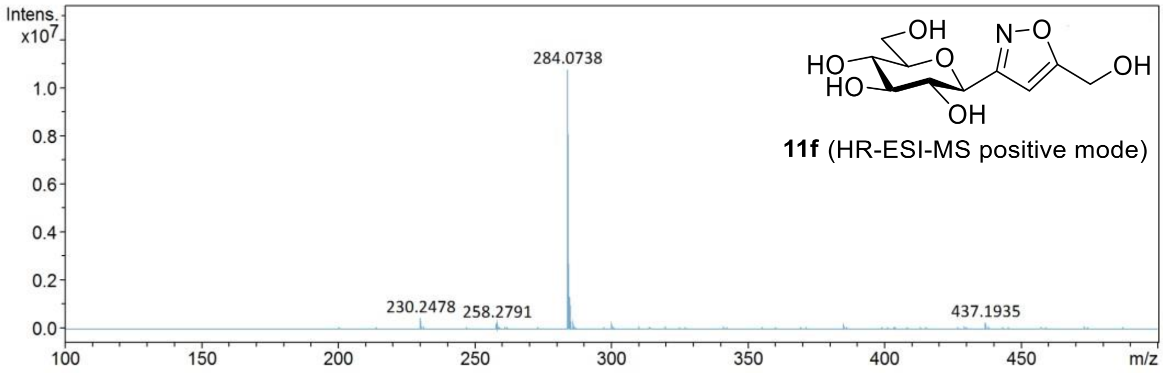

Figure S174. HR-ESI-MS spectrum of **11f**

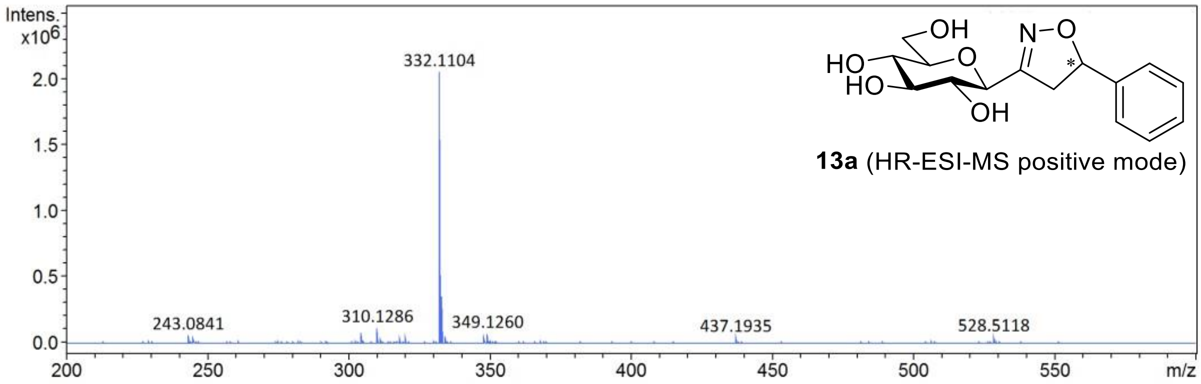

Figure S175. HR-ESI-MS spectrum of **13a**

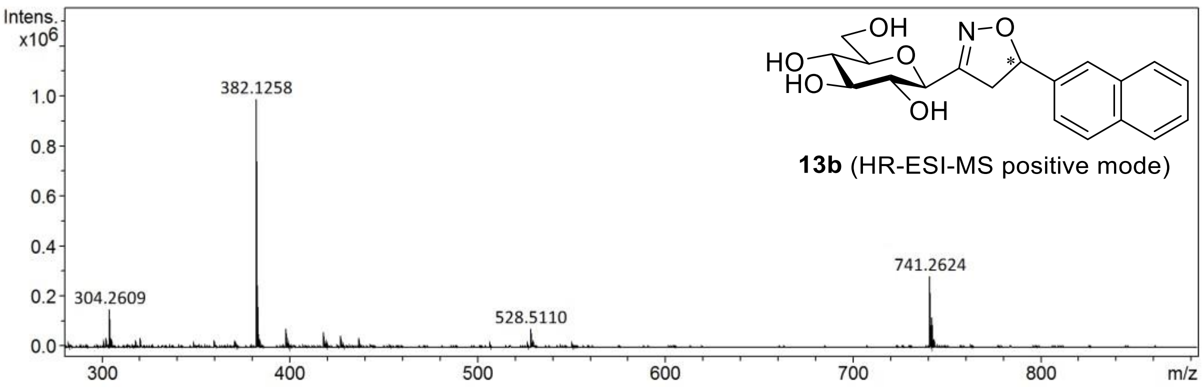

Figure S176. HR-ESI-MS spectrum of **13b**
